# A 3D Printed Device for Low Cost Neural Stimulation in Mice

**DOI:** 10.3389/fnins.2019.00784

**Published:** 2019-07-30

**Authors:** Taylor J. Morrison, Elana Sefton, Melissa Marquez-Chin, Milos R. Popovic, Cindi M. Morshead, Hani E. Naguib

**Affiliations:** ^1^Department of Mechanical and Industrial Engineering, University of Toronto, Toronto, ON, Canada; ^2^Institute of Biomaterials and Biomedical Engineering, University of Toronto, Toronto, ON, Canada; ^3^Department of Engineering, Universidad Iberoamericana, Mexico City, Mexico; ^4^KITE, Toronto Rehabilitation Institute, University Health Network, Toronto, ON, Canada; ^5^Department of Surgery, University of Toronto, Toronto, ON, Canada; ^6^Department of Materials Science & Engineering, University of Toronto, Toronto, ON, Canada

**Keywords:** 3D printing, brain stimulation, neural implantation, manufacturing, platinum wire electrode

## Abstract

Electrical stimulation of the brain through the implantation of electrodes is an effective treatment for certain diseases and the focus of a large body of research investigating new cell mechanisms, neurological phenomena, and treatments. Electrode devices developed for stimulation in rodents vary widely in size, cost, and functionality, with the majority of recent studies presenting complex, multi-functional designs. While some experiments require these added features, others are in greater need of reliable, low cost, and readily available devices that will allow surgeries to be scheduled and completed without delay. In this work, we utilize 3D printing and common electrical hardware to produce an effective 2-channel stimulation device that meets these requirements. Our stimulation electrode has not failed in over 60 consecutive surgeries, costs less than $1 USD, and can be assembled in less than 20 min. 3D printing minimizes the amount of material used in manufacturing the device and enables one to match the curvature of the connector’s base with the curvature of the mouse skull, producing an ultra-lightweight, low size device with improved adhesion to the mouse skull. The range of the stimulation parameters used with the proposed device was: pulse amplitude 1–200 μA, pulse duration 50–500 μs and pulse frequency 1–285 Hz.

## Introduction

Neural stimulation has proven to be an effective method for the treatment of numerous neurological disorders including Parkinson’s disease (Benabid, 2003), epilepsy (Berényi et al., 2012), and depression (Neimat et al., 2008). Currently, researchers are exploring new ways in which neural stimulation techniques can be applied to further advance the medical field. Many of these studies consist of small animal trials with the electrode devices used varying widely in size, functionality, and cost. Multiple groups have reported on wireless battery-powered stimulators that can be mounted on the head or back of rats (Ewing et al., 2013; Feng et al., 2017; Fluri et al., 2017). Hwang et al. (2015) developed a flexible piezoelectric energy harvester capable of deep brain stimulation in mice with only self-generated power. Using photolithography, Kim et al. (2018) created a multi-functional flexible polyimide probe with channels for stimulating, recording, and grounding which can be connected to the external stimulation system through a commercial plastic connector. While these devices offer the benefits of wireless stimulation and flexible probes, which are ideal for experiments with freely moving animals and chronic implantation, respectively, they have complex designs which cannot be manufactured quickly and cheaply for high turnover experiments. Vogler et al. (2017) effectively argue the benefits of simple, low cost devices for neural implantation and demonstrate how this design philosophy can be applied to EEG recordings. Furthermore, many of the stimulation devices described in literature are designed for rats, making them too large to properly function when used with mice. This can be disadvantageous for studies that seek to understand the neurophysiological mechanisms of electrical stimulation, as there is a larger genetic toolbox and the possibility for transgenic mice models that can be used to study these mechanisms (Ellenbroek and Youn, 2016). These tools are largely unavailable in rat models. In this work, we seek to fill the demand for stimulation devices which can be used for exploratory or optimization studies that require a large number of mouse surgeries (Vry et al., 2010; Lim et al., 2015; Podda et al., 2016). To be easily accessible to researchers, the stimulation devices should be manufactured quickly and at minimal cost, while also being suited to the animal and implantation site of interest, in size, geometry, and material biocompatibility.

To meet all these design requirements, we have utilized additive manufacturing and common circuit board components to create an effective 2-channel stimulation device. Additive manufacturing, also known as 3D printing, has already been shown to be a powerful and versatile tool for manufacturing neural devices. Notably, some groups have developed microdrive devices with 3D printed bodies suitable for implantation in mice (Voigts et al., 2013; Freedman et al., 2016). Other works have used 3D printing to produce a headstage (Pinnell et al., 2016), waterproof cap (Pinnell et al., 2018), and microdrive housing (Polo-Castillo et al., 2019) to protect electronic implants in rats. 3D printing has also been utilized in human applications through the fabrication of individualized headsets that can hold a stimulator in position over the scalp with better accuracy and reproducibility than traditional methods (Mansouri et al., 2018). While these examples focus on auxiliary parts, printing has also shown huge potential for the manufacturing of the electronic components themselves. In particular, printed soft electronics are expected to be at the forefront of next generation neural devices (Inzelberg and Hanein, 2019). Despite these works, it is clear that 3D printing has not yet penetrated the field of neural interfacing devices (Szostak et al., 2017). However, there is a need for new manufacturing, packaging, and integration methods that 3D printing could readily meet.

In this work, we present a novel design and manufacturing procedure for a 3D printed device for electric field application (i.e., electrical stimulation) in mice. Each device can be manufactured quickly, and at a minimal cost, using relatively accessible equipment, hardware and techniques. Furthermore, the customizability of 3D printing has been utilized to produce a unique device geometry that conforms to the shape of the mouse skull, improving ease and quality of adhesion. We demonstrate *in vivo* the effectiveness of these devices, as well as provide detailed instructions on how to make them. Overall, these electrodes are expected to reduce delays in testing, and therefore the rate at which results are produced.

## Materials and Methods

### Design and 3D Printing of Electrode Connector

**FIGURE 1:**
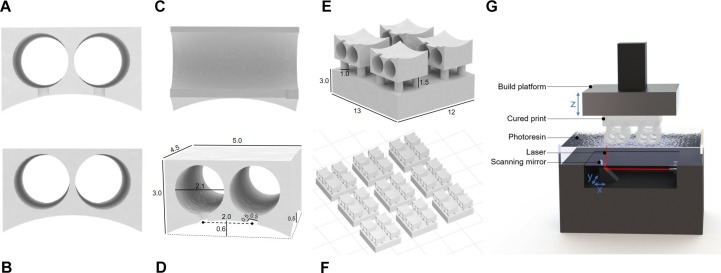
Renderings of connector drawings sent to 3D printer. **(A)** Front view showing grooves for electrode alignment. **(B)** Back view of connection site. **(C)** Side view with interior channels visible. **(D)** Rendering with dimensions indicated. **(E)** Single connector array with supports. **(F)** Electrode base array layout in PreForm for batch printing. **(G)** Stereolithography schematic (not to scale).

The 3D printed connector was designed in SOLIDWORKS 2017 (Figure 1). It consists of a 5 mm by 4.5 mm rectangular base ranging from 3 mm high at the four corners to 2.4 and 2.5 mm high at the bisectors of the front/back and side edges, respectively. The top face of the connector is level, while the bottom is concave as a result of cylindrical arcs cut through the bottom of the front, back, and side faces. This curvature was designed so that the device conforms to the shape of the mouse skull. Two parallel circular holes, each 2.1 mm in diameter, extend through the center of the connector to create open channels between the front and back faces. The center axes of the channels are spaced 2 mm apart. To guide the placement of the wire electrodes, small grooves 0.5 mm wide by 0.5 mm deep extend from the center of each channel to the bottom of the connector on the front face only.

To improve printability, temporary support features that can be removed after printing were added to the design. The connector was oriented so that the level top was facing downward. Small columns 1 mm in diameter were placed on each level corner of the part, extending downward 1.5 mm to meet a 3 mm thick support layer. A support layer must be included to ensure the part adheres to the build platform, as detachment during printing would ruin the part and contaminate the resin tank. The support columns isolate the part to maintain the integrity of the design and must be thick enough to hold the weight of the part while also being thin enough to be easily broken after printing. To facilitate handling during the post-printing process, the support layer encompasses 4 connectors forming a 2 by 2 array within a single continuous part (Figure 1E).

3D printing was performed with the desktop stereolithographic 3D printer Form 2 (Formlabs Inc., United States). An STL file (Supplementary Table S1) containing the previously described design was exported from SOLIDWORKS to PreForm, the Formlabs print preparation software bundled with the Form 2. In a typical print, the 4-connector array was duplicated 8 times and arranged into a 3 by 3 array with 5 mm spacing between pieces, totaling 36 individual connectors per print job. The liquid photopolymer resin “Clear” (FLGPCL04) was selected because of its suitability for higher resolution prints. The resolution was set to the maximum allowable value of 0.025 mm. With the selected settings, the print takes approximately 3 h to complete.

After printing, the parts were removed from the build platform within 30 min of the print’s completion. They were immediately transferred to a bath of isopropanol to remove excess uncured resin. They were soaked for at least 20 min with vigorous manual agitation of the bath at the 10-min mark. After soaking, the electrode bases were spread out on a paper towel to absorb any excess liquid. Finally, the parts were transferred to the Form Cure automated post-processing system (Formlabs Inc., United States) where they were cured under UV light at 60 degrees Celsius for 120 min. The cured support components were removed with wire cutters.

### Device Assembly

**FIGURE 2:**
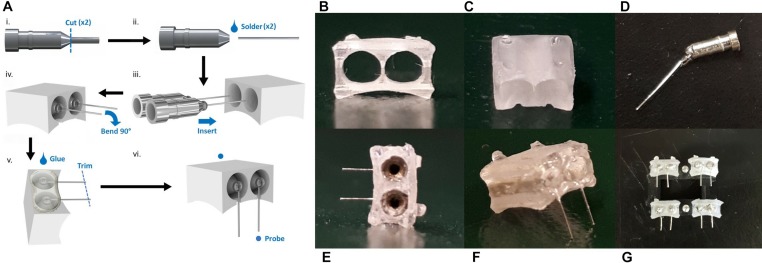
The device assembly process. **(A)** Step-by-step production schematic. **(i)** Remove end of machine pin with wire cutters. **(ii)** Solder Pt wire to pin. **(iii)** Insert pin to each channel of the printed connector. **(iv)** Bend Pt wires 90° downward and align with grooves. **(v)** Apply superglue to electrode face. When dry, trim wires to 2 mm. **(vi)** Probe to confirm conductive pathways are open and there are no shorts between channels. Photos of printed connector from the panel **(B)** eye-level front view, and **(C)** front view showing notches. **(D)** Cut machine pin soldered to Pt wire. **(E)** Back view with machine pins inserted. **(F)** Completed electrode. **(G)** Set of electrodes.

The device assembly steps are illustrated in Figure 2. Approximately 7 mm of hard uncoated platinum wire 0.127 mm in diameter (A-M Systems Inc., United States) was cut from the roll. A standard machine pin was removed from an IC socket by cutting away the plastic holder with wire cutters. Then the long, thin tip of the pin was removed, also with wire cutters. The shortened machine pin and wire were aligned on a flat working surface with the wire extending straight out from the center axis of the pin. While holding the machine pin in place with a pair of tweezers, the pieces were soldered together by carefully transferring a small amount of solder from the end of a hot soldering iron to the connection point at the tip of the pin. This process was repeated a second time to produce 2 pin-wire pieces per connector. The metal pieces were inserted into the connector by feeding the wire end through the back face of the printed channels. The pin end, which the connector has been designed to fit precisely, locks the pin-wire piece in place once it is completely inserted. The extruding wires were bent 90 degrees toward the front face of the connector and aligned with the guide grooves. Spare machine pins were inserted into the connector pins and balanced on the work surface to hold the device with the wires horizontal. A small amount of super glue (LePage Super Glue – Ultra Liquid Control, Henkel Canada Corp., Canada) was spread across the front face of the connector where the pins and wires meet to secure the wires in place and provide insulation on conductive areas that would be exposed to the mouse after implantation. The glue was left to dry between 3 and 12 h. Finally, the platinum wires were cut with small stainless steel scissors to be 2 mm long, excluding the length of wire contained within the arc area. The spare machine pins were removed, and the end-to-end conductivity of each channel was checked with a multimeter for quality control.

### Electrochemical Impedance Spectroscopy

Electrochemical impedance spectroscopy (EIS) was performed using an electrochemical analyzer (Model CHI6054E, CH Instruments, United States) to measure the electrodes’ impedance. A three-electrode configuration was used with a silver/silver chloride (Ag/AgCl) reference electrode (CHI111, CH Instruments, United States), a platinum counter electrode (CHI221, CH Instruments, United States), and the device wires as a working electrode. A 0.9% saline solution at room temperature was used as the electrolyte in a 642.62 cm^3^ (7.1 cm in height, 6.1 cm top radius, and 4.6 bottom radius) plastic cylindrical container. A 500 mV sine wave was applied at a frequency ranging from 50 to 0.01 Hz with 12 measurements per decade. This procedure was performed before implantation of the device as well as post-surgery.

### Tensile and Compression Testing

Tensile and compression tests were performed with the Q800 Dynamic Mechanical Analyzer (TA Instruments Inc., United States) to determine the force required to connect and disconnect an external lead to and from the device. Tensile clamps were used for all tests, and position and mass calibrations were conducted before each test session. The device wires were bent 180 degrees to avoid interference with the clamps. Starting with the tensile test, the device was secured in the bottom clamp with the pin holes facing upward and the edge of the connector leveled with the edge of the clamp. A machine pin with the plastic casing still attached was inserted into one of the device channels. The alignment of the pin was visually checked to be fully vertical before continuing, readjusting the clamped device if necessary. The bottom clamp was raised to the zero position and the top clamp was secured around the machine pin casing. Tensile force was applied at a rate of 0.5 N/min until the pieces were separated, which registered as sample yield by the DMA software, stopping the test. The bottom clamp was then locked in position so that the tip of the machine pin and top of the device were almost touching. Compressive force was applied at a rate of −0.5 N/min until the pin was fully inserted. The test was stopped manually once the DMA software was recording no significant displacement.

### Scanning Electron Microscopy (SEM)

Pieces of platinum wire were cut from the roll as usual with small scissors and soaked in 70% ethanol for 10 min. Each piece was then secured to a SEM pin mount with a strip of tape through the middle of the wire. The sample was imaged with the Jeol JSM-IT100 SEM at a voltage of 10 kV.

### Electrode Implant Surgery

Animal work was approved by the University of Toronto Animal Care Committee in accordance with institutional guidelines (Protocol No. 20011279). Surgeries were performed on C57/BL6 mice aged 7–11 weeks (Charles River).

Animals were placed in a stereotactic apparatus and an incision was made along the scalp’s midline. The skull surface was dried using a Q-tip cotton swab to ensure the electrode would adhere to the bone. Using a dental drill (#77, 0.018, 8177, Kopf), two holes were drilled at anterior +0.8, lateral −0.7, and anterior +0.8, lateral −2.7, relative to Bregma for the electrode leads. The insertion of the electrodes was accomplished with reverse action forceps attached to the stereotaxis apparatus. The electrode was positioned above the two drilled holes and lowered into the brain with small turns of the stereotactic or inserted by hand. Insta-cure+ cyanoacrylate glue (Bob Smith Industries, United States) was used to secure the electrode in place by placing the glue at the bottom of the electrode base. The reverse forceps in the stereotactic were pressed onto the electrode to apply pressure to secure the electrode to the skull. Once the electrodes were in place, the scalp was sutured closed with 4–0 sterile silk sutures.

### Electrical Stimulation Paradigm

Beginning 2 days after electrode implantation, mice received electrical stimulation. Mice were anesthetized with 1.5–2.5% isofluorane, placed in the stereotaxic and the implanted electrode was interfaced with a biphasic electrical stimulator. Mice received stimulation for 1 h using pulse parameters: 1–200 μA amplitude, 50–500 μs pulse width, 1–285 Hz pulse repetition frequency, as previously described by Babona-Pilipos et al. (2015). Following each stimulation session, mice were returned to their home cages for 24 or 72 h. Mice were sacrificed with an overdose of Avertin, transcardially perfused with 4% ice-cold paraformaldehyde and the brains removed. Brains were placed in 4% paraformaldehyde and transferred to 30% sucrose 4 h following perfusion.

### Immunohistochemistry

Brains were sectioned on a cryostat (HM525 NX, Thermo Fisher Scientific, United States) at a thickness of 20 μm. Sections were stained with primary antibodies NeuN, rabbit polyclonal Ab, 1:100 (ABN78, Abcam, United Kingdom), GFAP, mouse polyclonal IgG 1:1000 (63893, Sigma-Aldrich, United States), and Iba1, rabbit polyclonal Ab, 1:500 (019-19741, Wako Chemicals, United States). Secondaries used were goat anti-rabbit IgG 488 (A11036, Invitrogen, United States) for Iba1, and donkey anti-rabbit IgG 647 (A212336, Invitrogen, United States) for NeuN and GFAP, and goat anti-mouse IgG 647 for GFAP (A21236, Invitrogen, United States) all at 1:500 in PBS. Dapi (D1306, Invitrogen, United States) was used for nuclear staining (1:1000) in PBS for 5 min.

### Image Analysis

Fifteen sections from three mice each were analyzed for each condition. Imaging was performed with an Olympus FV1000 laser scanning microscope at 20× and 40× magnification to generate 20 μm-thick z-stacks. Images were taken of the dorsolateral corner of the lateral ventricle subependyma within a 650 μm^2^ region of interest (ROI) within 300 μm rostral and caudal to electrode implant site. The total number of double labeled nuclei (DAPI+), neurons (NeuN+), astrocytes (DCX+), or microglia (Iba1+) in a given ROI were counted and expressed as total number of double positive cell in ROI. Images were analyzed using Fiji Imaging Software (Schindelin et al., 2012).

## Results

### Manufacturing Process and Cost Analysis

The cost of the materials required to manufacture a single device was calculated (Table 1). The platinum wire and resin, which were shipped to our department from their respective suppliers, do not include any additional shipping or handling costs. The pins and glue were simply obtained from the local hardware store. While the excess platinum required to manipulate the wire during manufacturing but trimmed from the final device is included in the cost estimation, the excess resin disposed of during the printing, washing, and clean up process is considered a negligible cost and omitted. However, this estimation does assume that 2 connectors per 36 connector print job will fail during printing (6% fail rate). Therefore, the total material cost of a single device was determined to be $0.97 USD. At less than a dollar each, these electrodes are clearly affordable for researchers and would not be a constraining factor when considering the size and scope of the study.

**TABLE 1:** Bill of materials with costs.

**Component**	**Bulk amount**	**Bulk cost (USD)**	**Amount per device**	**Cost per device (USD)**
Platinum wire, 127 μm dia.	3.048 m	$183	14 mm	$0.84
Formlabs clear resin	1 L	$149	0.153 mL	$0.02
Machine pins	40 pin IC socket	$1.21	2	$0.06
Liquid super glue	4 mL	$7.50	0.018 mL	$0.03

Another factor that must be considered is the manufacturing lead time for each electrode. From start to finish, the process takes 9–18 h depending on the time the glue is allowed to dry. Fortunately, each electrode takes only 17 min to assemble by a practiced person. The rest of the time, which includes printing (3.5 h), washing (20 min), curing (2 h), and letting the glue dry (minimum 3 h from our experience; maximum 12 h for product-guaranteed full cure) does not require active participation by the maker. Printing and curing can also be done ahead of time in bulk quantities so that connectors are readily available. Overall, this manufacturing process has a quick turnaround time with little active time required, freeing the maker to complete other tasks throughout the procedure.

### Impedance

**FIGURE 3:**
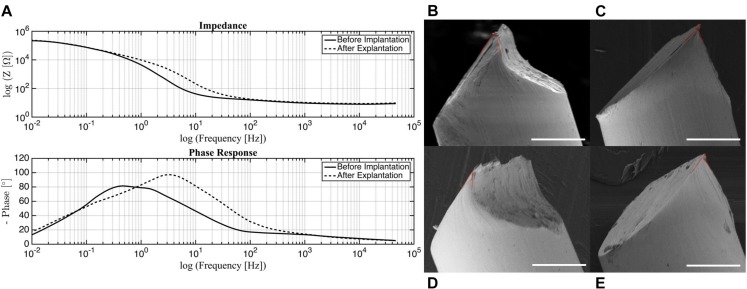
**(A)** Impedance and phase of electrodes before implantation and post-surgery. SEM images of probe tip with **(B)** 50 degree, **(C)** 25 degree (small) and 64 degree (large), **(D)** 29 degree, **(E)** 20 degree (small) and 70 degree (large) opening angles marked in red. Scale bar 50 μm.

The measurement of impedance is often used to characterize the electrical dynamic properties of a system (Geddes et al., 1971; Macdonald and Barsoukov, 2005; Lempka et al., 2009; Miocinovic et al., 2009; Wei and Grill, 2009). Before implantation, the electrodes show a high impedance magnitude (more than 10 kΩ) in the low frequency range from 0.01 to 1 Hz, and a low impedance magnitude (average 14 Ω) in the mid to high frequency ranges from 80 to 50 kHz (Figure 3). Looking at the corresponding phase response, the phase peaks at 80 degrees within the low frequency range and decreases steadily from 20 degrees in the high frequency range.

It can be seen from the impedance plot that the electrodes exhibit linear behavior that remains stable from 80 up to almost 50 kHz. These results suggest that the electrodes will be reliable within this frequency band, with minimal changes in impedance magnitude or phase. For the experiments in which these electrodes were used, the frequency of stimulation (285 Hz) falls within the acceptable range. This validates that the stimulation was delivered as intended without major variations in electrical parameters. For *in vivo* tests, this is of utter importance since any changes in current or voltage due to impedance disparity may cause tissue damage (Wei and Grill, 2009).

Comparing the impedance magnitude and phase response of the electrodes before and after implantation, the morphology of the curves does not change significantly, but there is a shift in both impedance magnitude and phase response post-surgery. The most substantial change takes place in the phase response low frequency range with a peak at 0.8 Hz before implantation that shifts to 4 Hz post-surgery. Although care was taken to clean the used electrodes, it is possible that organic and inorganic residue was still present on the tested samples. Organic residue can increase the impedance of an electrode and contribute to the generation of faradaic currents (Franks et al., 2005; Miocinovic et al., 2009). It is also possible that the electrodes experienced mechanical damage during the removal process because of the force required to detach them from the skull. Some of the post-surgery electrodes that were tested had bent wires and it was necessary to straighten them to their original state. Further investigation should be done to determine the source of this variation; however, the shift is observed mostly in the non-stable regions outside of our frequency range for stimulation, so this phenomenon is not a major concern.

### Probe Tip Geometry

Several platinum wires with tips cut by small stainless steel scissors were examined under SEM. The opening angle of the probe, as defined by the acute angle between edges of the point, was measured (Weltman et al., 2016). These are approximate measurements, as the images do not show a direct cross-section of the cuts. While the overall geometry of the tip was consistent across all samples, the point of the probe was one of two distinct shapes. The final tip was either elongated (Figures 3B,D), or had a small overhang (Figures 3C,E). This variance could be a result of each side of the scissors forming different cut shapes. For the elongated tips, the opening angle was 29 and 50 degrees. This falls within the range of 20–50 degrees identified as being able to penetrate the dura easily, but with dimpling (Edell et al., 1992). For the overhanging tips, the small overhang angle was measured as 20 and 25 degrees, with the angle of the bulk tip measuring at 70 and 64 degrees, respectively. While the smaller angle is ideal, the thinness of that region may cause it to buckle under the force of the dura, making the large angle the effective opening angle. In this case, penetration of the dura may cause more trauma. Overall, acceptable probe tips can be obtained simply with small scissors, but the orientation of the scissors is an important consideration for achieving an optimal opening angle.

### Connection and Disconnection Force

**FIGURE 4:**
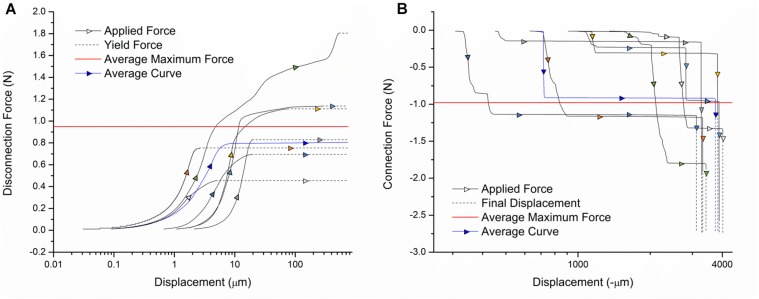
Force-displacement curves for the **(A)** disconnection and **(B)** connection of external leads. Individual sample curves are traced by different colored arrows. A sample curve representative of the average of all trials is shown in blue. The average maximum force exerted on the device during connection/disconnection is shown in red.

The connection and disconnection force-displacement curves resulting from a single pin being inserted and removed from one channel of the device are displayed in Figure 4. During disconnection, there is a small displacement (<10 μm) when force is initially applied. As the force is increased, little displacement continues to be observed until the yield force is reached, at which point the pin is abruptly removed. Therefore, the yield force is the maximum force that would be applied to the mouse skull because of the disconnection of external leads during surgery. Multiple samples were tested with yield forces ranging from 0.45 to 1.8 N, with an average of 0.95 N. During connection, compressive force is steadily applied. This corresponds to little or no displacement until the force required to insert the pin is reached, at which point there is a large increase in displacement. For some samples, this happens in two steps since the pin is tapered, which can result in partial insertion. After this rapid increase, no more displacement is observed, indicating the pin has been fully inserted. Therefore, the final force plateau is the maximum connection force that would be applied to the mouse skull. This force ranges from 0.15 to 1.8 N in magnitude, with an average of 0.98 N. The variation between samples can be attributed to misalignment of the pins from vertical and slight inconsistencies in pin shape. We tested both older samples, for which the lead pin and device pin had been connected/disconnected many times, and samples with new lead and device pins. There was no measurable difference between the two, indicating that wear of the pins is not a significant factor.

Based on existing designs, these are acceptable disconnection/connection force ranges. The commercially available Omnetics Nano Series connectors commonly used for this application require up to 2 N per contact (Hoch et al., 2018). Our design does not exceed this force, with most tested samples falling well below this value. Our procedure also inserts each cable separately, so the applied force does not increase with the number of channels. In literature, connectors utilizing magnetic clamping mechanisms have been designed with the purpose of minimizing the forces exerted on animals during surgery. One magnetic connector for microelectrode arrays required up to 4.9 N for disconnection, which our design is significantly below (Shah et al., 2014). Another has disconnection forces in the several hundred mN range, which is comparable to our device (Hoch et al., 2018). Thus, it can be concluded that external leads can be connected and disconnected from our device with safe levels of force exerted on the mouse.

### *In vivo* Stimulation

**FIGURE 5:**
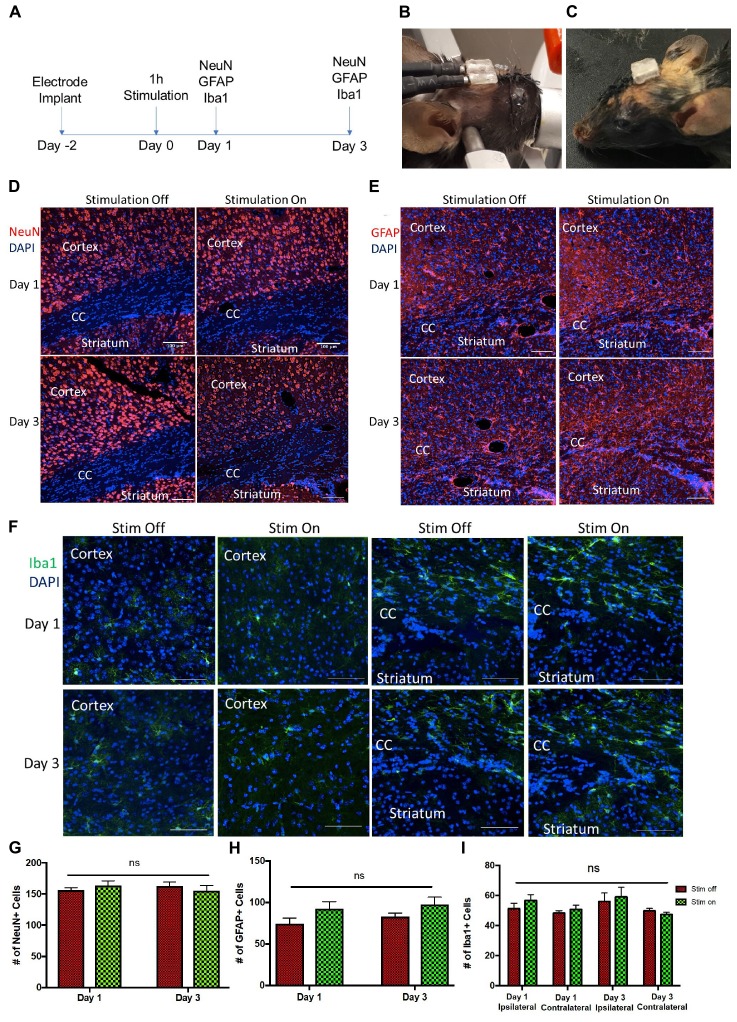
Tissue analysis of electrode implantation and electrical stimulation **(A)** Experimental paradigm for stimulation and tissue analyses. Photos of the implanted device **(B)** during and **(C)** after stimulation. **(D–F)** Representative images of ipsilateral brain hemisection within 300 μm of electrode implantation, showing the cortex, corpus callosum (CC), and striatum at day 1 and day 3 post-stimulation. **(D)** NeuN (red) and DAPI (blue) stained cells. Scale bar = 100 μm **(E)** GFAP (red) and DAPI (blue) stained cells. Scale bar = 100 μm. **(F)** Iba1 (green) and DAPI (blue) stained cells. Scale bar = 50 μm. **(G,H)** Quantification of NeuN+/DAPI+ **(G)** and GFAP+/DAPI+ cells **(H)** per 650 μm^2^ cells in unstimulated (stim off) and stimulated (stim on) groups on day 1 and day 3 post-stimulation. **(I)** Quantification of Iba1+/DAPI+ cells per 250 μm^2^ in stim off and stim on groups on day 1 and day 3 from ipsilateral and contralateral hemispheres relative to electrode implantation. Data presented as mean ± SEM.

During stimulation (Figure 5B), no hindlimb or forelimb movements or muscle twitches were seen, and respiration rate remained steady at comparable rates to unstimulated mice. When mice were returned to their home cage (Figure 5C), they were carefully monitored as per our animal protocol, and no abnormal motor behavior was observed.

We observed no change in the number of mature neurons (NeuN+) following 1 h of stimulation at 24 or 72 h post-stimulation (Figures 5A,D,G). Similarly, the numbers of GFAP+ astrocytes was not changed between the stimulated and unstimulated brains at either day 1 and day 3, or between day 1 and day 3, revealing that astrogliosis was not increased over time or in response to stimulation (Figures 5E,H). We further evaluated the immune response to the electrodes in stimulated and unstimulated groups, and demonstrated no change in Iba1+ cells between stimulated and unstimulated groups or over time (Figures 5F,I). Moreover, we compared the same regions between the implanted (ipsilateral) hemisphere and contralateral (non-implanted) hemisphere and saw no difference in Iba1+ cells (Figure 5I), indicating very little tissue damage from the electrode implant itself. Hence, these findings demonstrate that the presence of the implanted electrodes does not exacerbate the astrogliotic or inflammatory response in the presence or absence of stimulation.

## Discussion

While electrical stimulation is a proven method of treating neurological disorders, researchers are continuously finding new ways of applying this technique to advance the field of neuroscience. A great number of electrode devices have been developed for these experiments, with functionality, size, and cost varying widely among designs. Many of the devices described in literature tend to be complex, with multiple electronic components adding advanced functionality. However, there has been little work toward the improvement of more basic electrode device designs. This is a crucial gap, as complex designs tend to trade affordability, reliability, and accessibility in favor of added functions that may not be necessary for an experiment. In this work we have developed a stimulation device designed for high turnover experiments, such as those that seek to understand new cell mechanisms or optimize parameters. To do this, we have utilized 3D printing combined with off-the-shelf electronic hardware to produce a cost-effective, easily manufactured dual channel stimulating device.

Our manufacturing process was carefully designed for applications with mice over several days. The use of 3D printing allows the shape of the base of the connector to be matched to the curvature of the mouse skull, improving adhesion. The inconsistency between flat components and the curvature of the mouse skull is a frequently encountered issue which researchers have attempted to address in a number of ways, including limiting the size of the flat area (Heo et al., 2016), using flexible materials (Lee et al., 2017), applying multiple adhesive layers (Jeffrey et al., 2013), or cutting a curve with scissors (Bryant et al., 2009). In this case, 3D printing offers an elegant solution, avoiding the addition of steps in the surgical procedure or imprecise adjustment of material shape. For the conductive components, the machine pin and wire are joined through basic soldering, a skill commonly found among engineers and technicians and easily learned by others without prior knowledge. Finally, the liquid glue acts as sealant, so nearly no conductive surfaces are exposed to the mouse, in addition to providing structural support for soldered connections and securing the wires in position. The result is a relatively robust device that can be handled by researchers without fear of damage. Along with the simplicity of our design, this minimizes risk of device failure before or during implantation, saving both time and the need to sacrifice additional animals. Out of the 60+ surgeries performed with this design, we experienced no device failure before the electrodes were detached from the skull. Our device was also designed to be small and extremely lightweight so that animals can move freely and comfortably in between stimulation sessions. Weighing only 143 ± 8 mg, this device is well under the 2 g limit for ultra-light weight designs, and at 3 mm high it can also be considered ultra-low size (Battaglia et al., 2009).

One limitation of our design is the use of hard material for the electrodes. It is commonly understood that the mechanical mismatch between hard implants and soft brain matter can cause tissue damage and trigger an immune response (Liu et al., 2019). Glial cells such as astrocytes react to electrode implantation by forming an encapsulating scar around the probe, compromising stimulation (Salatino et al., 2017). In our electrical stimulation paradigm, there was no difference in glial response with or without stimulation at the anode and cathode. There was also no difference in glial response from day 1 to day 3 after implantation. It can be concluded that the use of our electrodes over a short time period will not trigger an immune response significant enough to impede the experiment. However, a greater glial response can be expected if this device was to be used for chronic implantation.

**FIGURE 6:**
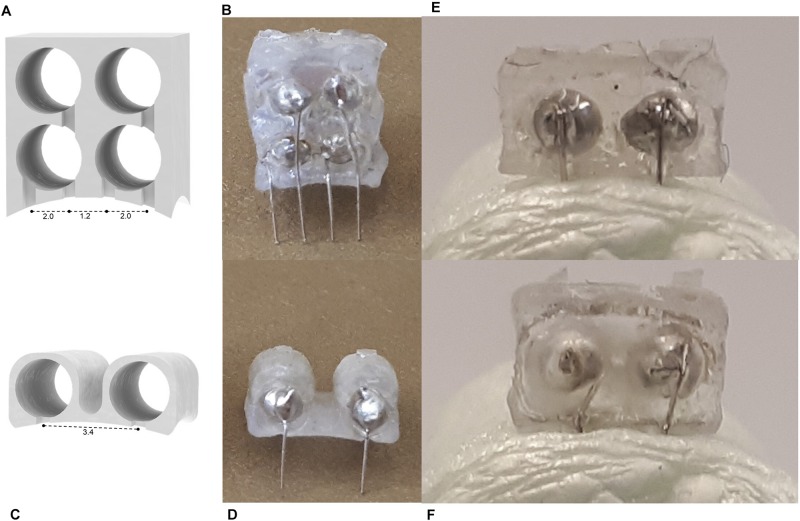
**(A–D)** Alternative device designs manufactured using the method described in this study. A 4-channel **(A)** schematic and **(B)** product, and a wide 2-channel device **(C)** schematic and **(D)** product. Images of **(E)** flat and **(F)** curved devices inserted in Styrofoam modeling the curvature of the mouse skull (radius = 11 mm) (Kim et al., 2016).

One of the primary advantages of 3D printing is the freedom and ease of customization. In Figures 6A–D, we present how this connector design could be easily modified to suit a wide range of experimental parameters. For the first alternative design, more channels were added to accommodate different stimulating or recording paradigms, while the second alternative design has increased distance between wires. The curvature at the base can also be customized to match the topography of the implantation site on the skull. This increases the contact area between the device and skull, improving adhesion (Figures 6E,F). In this study we have demonstrated the smallest design possible given the resolution of the printer and size of the machine pins. However, when increasing the span of the simulation area, or enlarging the device for rats, the design possibilities increase greatly. It should be noted that while the machine pins cannot be placed any closer together than they are in this design due to constraints on how thin a wall the 3D printer could produce, the guiding grooves could be adjusted and the platinum wires bent accordingly to suit experiments with smaller electrode spacing.

## Conclusion

In conclusion, we have a developed a low cost, ultra-light weight, easily manufactured stimulation device using 3D printing. At less than $1 USD and 17 min of assembly time per device, our design is ideal for high turnaround experiments for which electrodes should be readily available. We have verified the electrical and mechanical properties are suitable for the intended application, and the immune response of the brain is not significant over the short term. Finally, we believe that the versatility offered by 3D printing and our assembly process will enable our design to be adapted to a variety of stimulation procedures and facilitate the testing of new electrode materials.

## Data Availability

The raw data supporting the conclusions of this manuscript will be made available by the authors, without undue reservation, to any qualified researcher.

## Ethics Statement

This study was carried out in accordance with the recommendations of the University of Toronto Animal Care Committee (Protocol No. 20011279). The protocol was approved by the University of Toronto Animal Care Committee.

## Author Contributions

TM designed the device. TM and MM-C manufactured the electrodes. TM performed the mechanical testing, SEM imaging, and corresponding analyses. MM-C performed the electrical testing and analysis. ES performed *in vivo* testing and analysis. TM wrote the manuscript. HN, CM, and MP contributed to the planning, implementation, and analysis of research, and supervised the project.

## Conflict of Interest Statement

The authors declare that the research was conducted in the absence of any commercial or financial relationships that could be construed as a potential conflict of interest.
