# Supplementary material for: A 3D Printed Device for Low Cost Neural Stimulation in Mice
Source: Front Neurosci. 2019 Jul 30;13:784. doi: 10.3389/fnins.2019.00784 (PMC6682623; doi:10.3389/fnins.2019.00784)
Supplement: Supplementary file 1 [file Table_1.DOCX]

solid Electrode v19 with support

To convert this file into a printable .STL file, save these instructions and delete this textbox. Save this document as a plain text file [File 🡪 Save As 🡪 Plain text (*.txt)]. Close this file, then re-open it with a basic word processor such as Notepad. To change the file type, open the Save As window [File 🡪 Save As]. Select “All Files” from the “Save as type” drop-down menu. Add **.STL** to the end of the file name and click “Save”.

facet normal 1.161583e-001 0.000000e+000 9.932307e-001

outer loop

vertex 1.078841e+001 4.400000e+000 1.628060e+000

vertex 1.087523e+001 3.000000e+000 1.620464e+000

vertex 1.087523e+001 4.400000e+000 1.620464e+000

endloop

endfacet

facet normal 2.305519e-001 0.000000e+000 9.730600e-001

outer loop

vertex 1.087523e+001 4.400000e+000 1.620464e+000

vertex 1.087523e+001 3.000000e+000 1.620464e+000

vertex 1.095942e+001 3.000000e+000 1.597906e+000

endloop

endfacet

facet normal 2.868663e-001 0.000000e+000 9.579706e-001

outer loop

vertex 1.087523e+001 4.400000e+000 1.620464e+000

vertex 1.095942e+001 3.000000e+000 1.597906e+000

vertex 1.095942e+001 4.400000e+000 1.597906e+000

endloop

endfacet

facet normal 3.960194e-001 0.000000e+000 9.182422e-001

outer loop

vertex 1.095942e+001 4.400000e+000 1.597906e+000

vertex 1.095942e+001 3.000000e+000 1.597906e+000

vertex 1.103841e+001 3.000000e+000 1.561073e+000

endloop

endfacet

facet normal 4.488580e-001 0.000000e+000 8.936031e-001

outer loop

vertex 1.095942e+001 4.400000e+000 1.597906e+000

vertex 1.103841e+001 3.000000e+000 1.561073e+000

vertex 1.103841e+001 4.400000e+000 1.561073e+000

endloop

endfacet

facet normal 5.494540e-001 0.000000e+000 8.355240e-001

outer loop

vertex 1.103841e+001 4.400000e+000 1.561073e+000

vertex 1.103841e+001 3.000000e+000 1.561073e+000

vertex 1.110980e+001 3.000000e+000 1.511082e+000

endloop

endfacet

facet normal 5.972114e-001 0.000000e+000 8.020838e-001

outer loop

vertex 1.103841e+001 4.400000e+000 1.561073e+000

vertex 1.110980e+001 3.000000e+000 1.511082e+000

vertex 1.110980e+001 4.400000e+000 1.511082e+000

endloop

endfacet

facet normal 6.861938e-001 0.000000e+000 7.274188e-001

outer loop

vertex 1.110980e+001 4.400000e+000 1.511082e+000

vertex 1.110980e+001 3.000000e+000 1.511082e+000

vertex 1.117143e+001 3.000000e+000 1.449454e+000

endloop

endfacet

facet normal 7.274188e-001 0.000000e+000 6.861938e-001

outer loop

vertex 1.110980e+001 4.400000e+000 1.511082e+000

vertex 1.117143e+001 3.000000e+000 1.449454e+000

vertex 1.117143e+001 4.400000e+000 1.449454e+000

endloop

endfacet

facet normal 8.020838e-001 0.000000e+000 5.972114e-001

outer loop

vertex 1.117143e+001 4.400000e+000 1.449454e+000

vertex 1.117143e+001 3.000000e+000 1.449454e+000

vertex 1.122142e+001 3.000000e+000 1.378060e+000

endloop

endfacet

facet normal 8.355240e-001 0.000000e+000 5.494540e-001

outer loop

vertex 1.117143e+001 4.400000e+000 1.449454e+000

vertex 1.122142e+001 3.000000e+000 1.378060e+000

vertex 1.122142e+001 4.400000e+000 1.378060e+000

endloop

endfacet

facet normal 8.936031e-001 0.000000e+000 4.488580e-001

outer loop

vertex 1.122142e+001 4.400000e+000 1.378060e+000

vertex 1.122142e+001 3.000000e+000 1.378060e+000

vertex 1.125826e+001 3.000000e+000 1.299070e+000

endloop

endfacet

facet normal 9.182422e-001 0.000000e+000 3.960194e-001

outer loop

vertex 1.122142e+001 4.400000e+000 1.378060e+000

vertex 1.125826e+001 3.000000e+000 1.299070e+000

vertex 1.125826e+001 4.400000e+000 1.299070e+000

endloop

endfacet

facet normal 9.579706e-001 0.000000e+000 2.868663e-001

outer loop

vertex 1.125826e+001 4.400000e+000 1.299070e+000

vertex 1.125826e+001 3.000000e+000 1.299070e+000

vertex 1.128081e+001 3.000000e+000 1.214884e+000

endloop

endfacet

facet normal 9.730600e-001 0.000000e+000 2.305519e-001

outer loop

vertex 1.125826e+001 4.400000e+000 1.299070e+000

vertex 1.128081e+001 3.000000e+000 1.214884e+000

vertex 1.128081e+001 4.400000e+000 1.214884e+000

endloop

endfacet

facet normal 9.932307e-001 0.000000e+000 1.161583e-001

outer loop

vertex 1.128081e+001 4.400000e+000 1.214884e+000

vertex 1.128081e+001 3.000000e+000 1.214884e+000

vertex 1.128841e+001 3.000000e+000 1.128060e+000

endloop

endfacet

facet normal 9.983119e-001 0.000000e+000 5.807913e-002

outer loop

vertex 1.128081e+001 4.400000e+000 1.214884e+000

vertex 1.128841e+001 3.000000e+000 1.128060e+000

vertex 1.128841e+001 4.400000e+000 1.128060e+000

endloop

endfacet

facet normal 9.983119e-001 0.000000e+000 -5.807913e-002

outer loop

vertex 1.128841e+001 4.400000e+000 1.128060e+000

vertex 1.128841e+001 3.000000e+000 1.128060e+000

vertex 1.128081e+001 3.000000e+000 1.041236e+000

endloop

endfacet

facet normal 9.932307e-001 0.000000e+000 -1.161583e-001

outer loop

vertex 1.128841e+001 4.400000e+000 1.128060e+000

vertex 1.128081e+001 3.000000e+000 1.041236e+000

vertex 1.128081e+001 4.400000e+000 1.041236e+000

endloop

endfacet

facet normal 9.730600e-001 0.000000e+000 -2.305519e-001

outer loop

vertex 1.128081e+001 4.400000e+000 1.041236e+000

vertex 1.128081e+001 3.000000e+000 1.041236e+000

vertex 1.125826e+001 3.000000e+000 9.570499e-001

endloop

endfacet

facet normal 9.579706e-001 0.000000e+000 -2.868663e-001

outer loop

vertex 1.128081e+001 4.400000e+000 1.041236e+000

vertex 1.125826e+001 3.000000e+000 9.570499e-001

vertex 1.125826e+001 4.400000e+000 9.570499e-001

endloop

endfacet

facet normal 9.182422e-001 0.000000e+000 -3.960194e-001

outer loop

vertex 1.125826e+001 4.400000e+000 9.570499e-001

vertex 1.125826e+001 3.000000e+000 9.570499e-001

vertex 1.122142e+001 3.000000e+000 8.780602e-001

endloop

endfacet

facet normal 8.936031e-001 0.000000e+000 -4.488580e-001

outer loop

vertex 1.125826e+001 4.400000e+000 9.570499e-001

vertex 1.122142e+001 3.000000e+000 8.780602e-001

vertex 1.122142e+001 4.400000e+000 8.780602e-001

endloop

endfacet

facet normal 8.355240e-001 0.000000e+000 -5.494540e-001

outer loop

vertex 1.122142e+001 4.400000e+000 8.780602e-001

vertex 1.122142e+001 3.000000e+000 8.780602e-001

vertex 1.117143e+001 3.000000e+000 8.066660e-001

endloop

endfacet

facet normal 8.020838e-001 0.000000e+000 -5.972114e-001

outer loop

vertex 1.122142e+001 4.400000e+000 8.780602e-001

vertex 1.117143e+001 3.000000e+000 8.066660e-001

vertex 1.117143e+001 4.400000e+000 8.066660e-001

endloop

endfacet

facet normal 7.274188e-001 0.000000e+000 -6.861938e-001

outer loop

vertex 1.117143e+001 4.400000e+000 8.066660e-001

vertex 1.117143e+001 3.000000e+000 8.066660e-001

vertex 1.110980e+001 3.000000e+000 7.450376e-001

endloop

endfacet

facet normal 6.861938e-001 0.000000e+000 -7.274188e-001

outer loop

vertex 1.117143e+001 4.400000e+000 8.066660e-001

vertex 1.110980e+001 3.000000e+000 7.450376e-001

vertex 1.110980e+001 4.400000e+000 7.450376e-001

endloop

endfacet

facet normal 5.972114e-001 0.000000e+000 -8.020838e-001

outer loop

vertex 1.110980e+001 4.400000e+000 7.450376e-001

vertex 1.110980e+001 3.000000e+000 7.450376e-001

vertex 1.103841e+001 3.000000e+000 6.950474e-001

endloop

endfacet

facet normal 5.494540e-001 0.000000e+000 -8.355240e-001

outer loop

vertex 1.110980e+001 4.400000e+000 7.450376e-001

vertex 1.103841e+001 3.000000e+000 6.950474e-001

vertex 1.103841e+001 4.400000e+000 6.950474e-001

endloop

endfacet

facet normal 4.488580e-001 0.000000e+000 -8.936031e-001

outer loop

vertex 1.103841e+001 4.400000e+000 6.950474e-001

vertex 1.103841e+001 3.000000e+000 6.950474e-001

vertex 1.095942e+001 3.000000e+000 6.582136e-001

endloop

endfacet

facet normal 3.960194e-001 0.000000e+000 -9.182422e-001

outer loop

vertex 1.103841e+001 4.400000e+000 6.950474e-001

vertex 1.095942e+001 3.000000e+000 6.582136e-001

vertex 1.095942e+001 4.400000e+000 6.582136e-001

endloop

endfacet

facet normal 2.868663e-001 0.000000e+000 -9.579706e-001

outer loop

vertex 1.095942e+001 4.400000e+000 6.582136e-001

vertex 1.095942e+001 3.000000e+000 6.582136e-001

vertex 1.087523e+001 3.000000e+000 6.356561e-001

endloop

endfacet

facet normal 2.305519e-001 0.000000e+000 -9.730600e-001

outer loop

vertex 1.095942e+001 4.400000e+000 6.582136e-001

vertex 1.087523e+001 3.000000e+000 6.356561e-001

vertex 1.087523e+001 4.400000e+000 6.356561e-001

endloop

endfacet

facet normal 1.161583e-001 0.000000e+000 -9.932307e-001

outer loop

vertex 1.087523e+001 4.400000e+000 6.356561e-001

vertex 1.087523e+001 3.000000e+000 6.356561e-001

vertex 1.078841e+001 3.000000e+000 6.280602e-001

endloop

endfacet

facet normal 5.807913e-002 0.000000e+000 -9.983119e-001

outer loop

vertex 1.087523e+001 4.400000e+000 6.356561e-001

vertex 1.078841e+001 3.000000e+000 6.280602e-001

vertex 1.078841e+001 4.400000e+000 6.280602e-001

endloop

endfacet

facet normal -5.807913e-002 0.000000e+000 -9.983119e-001

outer loop

vertex 1.078841e+001 4.400000e+000 6.280602e-001

vertex 1.078841e+001 3.000000e+000 6.280602e-001

vertex 1.070159e+001 3.000000e+000 6.356561e-001

endloop

endfacet

facet normal -1.161583e-001 0.000000e+000 -9.932307e-001

outer loop

vertex 1.078841e+001 4.400000e+000 6.280602e-001

vertex 1.070159e+001 3.000000e+000 6.356561e-001

vertex 1.070159e+001 4.400000e+000 6.356561e-001

endloop

endfacet

facet normal -2.305519e-001 0.000000e+000 -9.730600e-001

outer loop

vertex 1.070159e+001 4.400000e+000 6.356561e-001

vertex 1.070159e+001 3.000000e+000 6.356561e-001

vertex 1.061740e+001 3.000000e+000 6.582136e-001

endloop

endfacet

facet normal -2.868663e-001 0.000000e+000 -9.579706e-001

outer loop

vertex 1.070159e+001 4.400000e+000 6.356561e-001

vertex 1.061740e+001 3.000000e+000 6.582136e-001

vertex 1.061740e+001 4.400000e+000 6.582136e-001

endloop

endfacet

facet normal -3.960194e-001 0.000000e+000 -9.182422e-001

outer loop

vertex 1.061740e+001 4.400000e+000 6.582136e-001

vertex 1.061740e+001 3.000000e+000 6.582136e-001

vertex 1.053841e+001 3.000000e+000 6.950474e-001

endloop

endfacet

facet normal -4.488580e-001 0.000000e+000 -8.936031e-001

outer loop

vertex 1.061740e+001 4.400000e+000 6.582136e-001

vertex 1.053841e+001 3.000000e+000 6.950474e-001

vertex 1.053841e+001 4.400000e+000 6.950474e-001

endloop

endfacet

facet normal -5.494540e-001 0.000000e+000 -8.355240e-001

outer loop

vertex 1.053841e+001 4.400000e+000 6.950474e-001

vertex 1.053841e+001 3.000000e+000 6.950474e-001

vertex 1.046702e+001 3.000000e+000 7.450376e-001

endloop

endfacet

facet normal -5.972114e-001 0.000000e+000 -8.020838e-001

outer loop

vertex 1.053841e+001 4.400000e+000 6.950474e-001

vertex 1.046702e+001 3.000000e+000 7.450376e-001

vertex 1.046702e+001 4.400000e+000 7.450376e-001

endloop

endfacet

facet normal -6.861938e-001 0.000000e+000 -7.274188e-001

outer loop

vertex 1.046702e+001 4.400000e+000 7.450376e-001

vertex 1.046702e+001 3.000000e+000 7.450376e-001

vertex 1.040539e+001 3.000000e+000 8.066660e-001

endloop

endfacet

facet normal -7.274188e-001 0.000000e+000 -6.861938e-001

outer loop

vertex 1.046702e+001 4.400000e+000 7.450376e-001

vertex 1.040539e+001 3.000000e+000 8.066660e-001

vertex 1.040539e+001 4.400000e+000 8.066660e-001

endloop

endfacet

facet normal -8.020838e-001 0.000000e+000 -5.972114e-001

outer loop

vertex 1.040539e+001 4.400000e+000 8.066660e-001

vertex 1.040539e+001 3.000000e+000 8.066660e-001

vertex 1.035540e+001 3.000000e+000 8.780602e-001

endloop

endfacet

facet normal -8.355240e-001 0.000000e+000 -5.494540e-001

outer loop

vertex 1.040539e+001 4.400000e+000 8.066660e-001

vertex 1.035540e+001 3.000000e+000 8.780602e-001

vertex 1.035540e+001 4.400000e+000 8.780602e-001

endloop

endfacet

facet normal -8.936031e-001 0.000000e+000 -4.488580e-001

outer loop

vertex 1.035540e+001 4.400000e+000 8.780602e-001

vertex 1.035540e+001 3.000000e+000 8.780602e-001

vertex 1.031856e+001 3.000000e+000 9.570499e-001

endloop

endfacet

facet normal -9.182422e-001 0.000000e+000 -3.960194e-001

outer loop

vertex 1.035540e+001 4.400000e+000 8.780602e-001

vertex 1.031856e+001 3.000000e+000 9.570499e-001

vertex 1.031856e+001 4.400000e+000 9.570499e-001

endloop

endfacet

facet normal -9.579706e-001 0.000000e+000 -2.868663e-001

outer loop

vertex 1.031856e+001 4.400000e+000 9.570499e-001

vertex 1.031856e+001 3.000000e+000 9.570499e-001

vertex 1.029601e+001 3.000000e+000 1.041236e+000

endloop

endfacet

facet normal -9.730600e-001 0.000000e+000 -2.305519e-001

outer loop

vertex 1.031856e+001 4.400000e+000 9.570499e-001

vertex 1.029601e+001 3.000000e+000 1.041236e+000

vertex 1.029601e+001 4.400000e+000 1.041236e+000

endloop

endfacet

facet normal -9.932307e-001 0.000000e+000 -1.161583e-001

outer loop

vertex 1.029601e+001 4.400000e+000 1.041236e+000

vertex 1.029601e+001 3.000000e+000 1.041236e+000

vertex 1.028841e+001 3.000000e+000 1.128060e+000

endloop

endfacet

facet normal -9.983119e-001 0.000000e+000 -5.807913e-002

outer loop

vertex 1.029601e+001 4.400000e+000 1.041236e+000

vertex 1.028841e+001 3.000000e+000 1.128060e+000

vertex 1.028841e+001 4.400000e+000 1.128060e+000

endloop

endfacet

facet normal -9.983119e-001 0.000000e+000 5.807913e-002

outer loop

vertex 1.028841e+001 4.400000e+000 1.128060e+000

vertex 1.028841e+001 3.000000e+000 1.128060e+000

vertex 1.029601e+001 3.000000e+000 1.214884e+000

endloop

endfacet

facet normal -9.932307e-001 0.000000e+000 1.161583e-001

outer loop

vertex 1.028841e+001 4.400000e+000 1.128060e+000

vertex 1.029601e+001 3.000000e+000 1.214884e+000

vertex 1.029601e+001 4.400000e+000 1.214884e+000

endloop

endfacet

facet normal -9.730600e-001 0.000000e+000 2.305519e-001

outer loop

vertex 1.029601e+001 4.400000e+000 1.214884e+000

vertex 1.029601e+001 3.000000e+000 1.214884e+000

vertex 1.031856e+001 3.000000e+000 1.299070e+000

endloop

endfacet

facet normal -9.579706e-001 0.000000e+000 2.868663e-001

outer loop

vertex 1.029601e+001 4.400000e+000 1.214884e+000

vertex 1.031856e+001 3.000000e+000 1.299070e+000

vertex 1.031856e+001 4.400000e+000 1.299070e+000

endloop

endfacet

facet normal -9.182422e-001 0.000000e+000 3.960194e-001

outer loop

vertex 1.031856e+001 4.400000e+000 1.299070e+000

vertex 1.031856e+001 3.000000e+000 1.299070e+000

vertex 1.035540e+001 3.000000e+000 1.378060e+000

endloop

endfacet

facet normal -8.936031e-001 0.000000e+000 4.488580e-001

outer loop

vertex 1.031856e+001 4.400000e+000 1.299070e+000

vertex 1.035540e+001 3.000000e+000 1.378060e+000

vertex 1.035540e+001 4.400000e+000 1.378060e+000

endloop

endfacet

facet normal -8.355240e-001 0.000000e+000 5.494540e-001

outer loop

vertex 1.035540e+001 4.400000e+000 1.378060e+000

vertex 1.035540e+001 3.000000e+000 1.378060e+000

vertex 1.040539e+001 3.000000e+000 1.449454e+000

endloop

endfacet

facet normal -8.020838e-001 0.000000e+000 5.972114e-001

outer loop

vertex 1.035540e+001 4.400000e+000 1.378060e+000

vertex 1.040539e+001 3.000000e+000 1.449454e+000

vertex 1.040539e+001 4.400000e+000 1.449454e+000

endloop

endfacet

facet normal -7.274188e-001 0.000000e+000 6.861938e-001

outer loop

vertex 1.040539e+001 4.400000e+000 1.449454e+000

vertex 1.040539e+001 3.000000e+000 1.449454e+000

vertex 1.046702e+001 3.000000e+000 1.511082e+000

endloop

endfacet

facet normal -6.861938e-001 0.000000e+000 7.274188e-001

outer loop

vertex 1.040539e+001 4.400000e+000 1.449454e+000

vertex 1.046702e+001 3.000000e+000 1.511082e+000

vertex 1.046702e+001 4.400000e+000 1.511082e+000

endloop

endfacet

facet normal -5.972114e-001 0.000000e+000 8.020838e-001

outer loop

vertex 1.046702e+001 4.400000e+000 1.511082e+000

vertex 1.046702e+001 3.000000e+000 1.511082e+000

vertex 1.053841e+001 3.000000e+000 1.561073e+000

endloop

endfacet

facet normal -5.494540e-001 0.000000e+000 8.355240e-001

outer loop

vertex 1.046702e+001 4.400000e+000 1.511082e+000

vertex 1.053841e+001 3.000000e+000 1.561073e+000

vertex 1.053841e+001 4.400000e+000 1.561073e+000

endloop

endfacet

facet normal -4.488580e-001 0.000000e+000 8.936031e-001

outer loop

vertex 1.053841e+001 4.400000e+000 1.561073e+000

vertex 1.053841e+001 3.000000e+000 1.561073e+000

vertex 1.061740e+001 3.000000e+000 1.597906e+000

endloop

endfacet

facet normal -3.960194e-001 0.000000e+000 9.182422e-001

outer loop

vertex 1.053841e+001 4.400000e+000 1.561073e+000

vertex 1.061740e+001 3.000000e+000 1.597906e+000

vertex 1.061740e+001 4.400000e+000 1.597906e+000

endloop

endfacet

facet normal -2.868663e-001 0.000000e+000 9.579706e-001

outer loop

vertex 1.061740e+001 4.400000e+000 1.597906e+000

vertex 1.061740e+001 3.000000e+000 1.597906e+000

vertex 1.070159e+001 3.000000e+000 1.620464e+000

endloop

endfacet

facet normal -2.305519e-001 0.000000e+000 9.730600e-001

outer loop

vertex 1.061740e+001 4.400000e+000 1.597906e+000

vertex 1.070159e+001 3.000000e+000 1.620464e+000

vertex 1.070159e+001 4.400000e+000 1.620464e+000

endloop

endfacet

facet normal -1.161583e-001 0.000000e+000 9.932307e-001

outer loop

vertex 1.070159e+001 4.400000e+000 1.620464e+000

vertex 1.070159e+001 3.000000e+000 1.620464e+000

vertex 1.078841e+001 3.000000e+000 1.628060e+000

endloop

endfacet

facet normal -5.807913e-002 0.000000e+000 9.983119e-001

outer loop

vertex 1.070159e+001 4.400000e+000 1.620464e+000

vertex 1.078841e+001 3.000000e+000 1.628060e+000

vertex 1.078841e+001 4.400000e+000 1.628060e+000

endloop

endfacet

facet normal 5.807913e-002 0.000000e+000 9.983119e-001

outer loop

vertex 1.078841e+001 4.400000e+000 1.628060e+000

vertex 1.078841e+001 3.000000e+000 1.628060e+000

vertex 1.087523e+001 3.000000e+000 1.620464e+000

endloop

endfacet

facet normal -9.983119e-001 0.000000e+000 -5.807913e-002

outer loop

vertex 7.579920e+000 4.400000e+000 1.041236e+000

vertex 7.572323e+000 3.000000e+000 1.128060e+000

vertex 7.572323e+000 4.400000e+000 1.128060e+000

endloop

endfacet

facet normal -9.983119e-001 0.000000e+000 5.807913e-002

outer loop

vertex 7.572323e+000 4.400000e+000 1.128060e+000

vertex 7.572323e+000 3.000000e+000 1.128060e+000

vertex 7.579920e+000 3.000000e+000 1.214884e+000

endloop

endfacet

facet normal -9.932307e-001 0.000000e+000 1.161583e-001

outer loop

vertex 7.572323e+000 4.400000e+000 1.128060e+000

vertex 7.579920e+000 3.000000e+000 1.214884e+000

vertex 7.579920e+000 4.400000e+000 1.214884e+000

endloop

endfacet

facet normal -9.776480e-001 0.000000e+000 2.102483e-001

outer loop

vertex 7.579920e+000 4.400000e+000 1.214884e+000

vertex 7.579920e+000 3.000000e+000 1.214884e+000

vertex 7.594380e+000 3.000000e+000 1.268851e+000

endloop

endfacet

facet normal -9.637589e-001 0.000000e+000 2.667744e-001

outer loop

vertex 7.579920e+000 4.400000e+000 1.214884e+000

vertex 7.594380e+000 3.000000e+000 1.268851e+000

vertex 7.602478e+000 4.400000e+000 1.299070e+000

endloop

endfacet

facet normal -9.466226e-001 0.000000e+000 3.223439e-001

outer loop

vertex 7.594380e+000 3.000000e+000 1.268851e+000

vertex 7.602478e+000 3.000000e+000 1.299070e+000

vertex 7.602478e+000 4.400000e+000 1.299070e+000

endloop

endfacet

facet normal -9.182422e-001 0.000000e+000 3.960194e-001

outer loop

vertex 7.602478e+000 4.400000e+000 1.299070e+000

vertex 7.602478e+000 3.000000e+000 1.299070e+000

vertex 7.639311e+000 3.000000e+000 1.378060e+000

endloop

endfacet

facet normal -8.936031e-001 0.000000e+000 4.488580e-001

outer loop

vertex 7.602478e+000 4.400000e+000 1.299070e+000

vertex 7.639311e+000 3.000000e+000 1.378060e+000

vertex 7.639311e+000 4.400000e+000 1.378060e+000

endloop

endfacet

facet normal -8.355240e-001 0.000000e+000 5.494540e-001

outer loop

vertex 7.639311e+000 4.400000e+000 1.378060e+000

vertex 7.639311e+000 3.000000e+000 1.378060e+000

vertex 7.689301e+000 3.000000e+000 1.449454e+000

endloop

endfacet

facet normal -8.020838e-001 0.000000e+000 5.972114e-001

outer loop

vertex 7.639311e+000 4.400000e+000 1.378060e+000

vertex 7.689301e+000 3.000000e+000 1.449454e+000

vertex 7.689301e+000 4.400000e+000 1.449454e+000

endloop

endfacet

facet normal -7.274188e-001 0.000000e+000 6.861938e-001

outer loop

vertex 7.689301e+000 4.400000e+000 1.449454e+000

vertex 7.689301e+000 3.000000e+000 1.449454e+000

vertex 7.750930e+000 3.000000e+000 1.511082e+000

endloop

endfacet

facet normal -6.861938e-001 0.000000e+000 7.274188e-001

outer loop

vertex 7.689301e+000 4.400000e+000 1.449454e+000

vertex 7.750930e+000 3.000000e+000 1.511082e+000

vertex 7.750930e+000 4.400000e+000 1.511082e+000

endloop

endfacet

facet normal -5.972114e-001 0.000000e+000 8.020838e-001

outer loop

vertex 7.750930e+000 4.400000e+000 1.511082e+000

vertex 7.750930e+000 3.000000e+000 1.511082e+000

vertex 7.822324e+000 3.000000e+000 1.561073e+000

endloop

endfacet

facet normal -5.494540e-001 0.000000e+000 8.355240e-001

outer loop

vertex 7.750930e+000 4.400000e+000 1.511082e+000

vertex 7.822324e+000 3.000000e+000 1.561073e+000

vertex 7.822324e+000 4.400000e+000 1.561073e+000

endloop

endfacet

facet normal -4.488580e-001 0.000000e+000 8.936031e-001

outer loop

vertex 7.822324e+000 4.400000e+000 1.561073e+000

vertex 7.822324e+000 3.000000e+000 1.561073e+000

vertex 7.901314e+000 3.000000e+000 1.597906e+000

endloop

endfacet

facet normal -3.960194e-001 0.000000e+000 9.182422e-001

outer loop

vertex 7.822324e+000 4.400000e+000 1.561073e+000

vertex 7.901314e+000 3.000000e+000 1.597906e+000

vertex 7.901314e+000 4.400000e+000 1.597906e+000

endloop

endfacet

facet normal -2.868663e-001 0.000000e+000 9.579706e-001

outer loop

vertex 7.901314e+000 4.400000e+000 1.597906e+000

vertex 7.901314e+000 3.000000e+000 1.597906e+000

vertex 7.985500e+000 3.000000e+000 1.620464e+000

endloop

endfacet

facet normal -2.305519e-001 0.000000e+000 9.730600e-001

outer loop

vertex 7.901314e+000 4.400000e+000 1.597906e+000

vertex 7.985500e+000 3.000000e+000 1.620464e+000

vertex 7.985500e+000 4.400000e+000 1.620464e+000

endloop

endfacet

facet normal -1.161583e-001 0.000000e+000 9.932307e-001

outer loop

vertex 7.985500e+000 4.400000e+000 1.620464e+000

vertex 7.985500e+000 3.000000e+000 1.620464e+000

vertex 8.072324e+000 3.000000e+000 1.628060e+000

endloop

endfacet

facet normal -5.807913e-002 0.000000e+000 9.983119e-001

outer loop

vertex 7.985500e+000 4.400000e+000 1.620464e+000

vertex 8.072324e+000 3.000000e+000 1.628060e+000

vertex 8.072324e+000 4.400000e+000 1.628060e+000

endloop

endfacet

facet normal 5.807913e-002 0.000000e+000 9.983119e-001

outer loop

vertex 8.072324e+000 4.400000e+000 1.628060e+000

vertex 8.072324e+000 3.000000e+000 1.628060e+000

vertex 8.159147e+000 3.000000e+000 1.620464e+000

endloop

endfacet

facet normal 1.161583e-001 0.000000e+000 9.932307e-001

outer loop

vertex 8.072324e+000 4.400000e+000 1.628060e+000

vertex 8.159147e+000 3.000000e+000 1.620464e+000

vertex 8.159147e+000 4.400000e+000 1.620464e+000

endloop

endfacet

facet normal 2.305519e-001 0.000000e+000 9.730600e-001

outer loop

vertex 8.159147e+000 4.400000e+000 1.620464e+000

vertex 8.159147e+000 3.000000e+000 1.620464e+000

vertex 8.243334e+000 3.000000e+000 1.597906e+000

endloop

endfacet

facet normal 2.868663e-001 0.000000e+000 9.579706e-001

outer loop

vertex 8.159147e+000 4.400000e+000 1.620464e+000

vertex 8.243334e+000 3.000000e+000 1.597906e+000

vertex 8.243334e+000 4.400000e+000 1.597906e+000

endloop

endfacet

facet normal 3.960194e-001 0.000000e+000 9.182422e-001

outer loop

vertex 8.243334e+000 4.400000e+000 1.597906e+000

vertex 8.243334e+000 3.000000e+000 1.597906e+000

vertex 8.322324e+000 3.000000e+000 1.561073e+000

endloop

endfacet

facet normal 4.488580e-001 0.000000e+000 8.936031e-001

outer loop

vertex 8.243334e+000 4.400000e+000 1.597906e+000

vertex 8.322324e+000 3.000000e+000 1.561073e+000

vertex 8.322324e+000 4.400000e+000 1.561073e+000

endloop

endfacet

facet normal 5.494540e-001 0.000000e+000 8.355240e-001

outer loop

vertex 8.322324e+000 4.400000e+000 1.561073e+000

vertex 8.322324e+000 3.000000e+000 1.561073e+000

vertex 8.393718e+000 3.000000e+000 1.511082e+000

endloop

endfacet

facet normal 5.972114e-001 0.000000e+000 8.020838e-001

outer loop

vertex 8.322324e+000 4.400000e+000 1.561073e+000

vertex 8.393718e+000 3.000000e+000 1.511082e+000

vertex 8.393718e+000 4.400000e+000 1.511082e+000

endloop

endfacet

facet normal 6.861938e-001 0.000000e+000 7.274188e-001

outer loop

vertex 8.393718e+000 4.400000e+000 1.511082e+000

vertex 8.393718e+000 3.000000e+000 1.511082e+000

vertex 8.455345e+000 3.000000e+000 1.449454e+000

endloop

endfacet

facet normal 7.274188e-001 0.000000e+000 6.861938e-001

outer loop

vertex 8.393718e+000 4.400000e+000 1.511082e+000

vertex 8.455345e+000 3.000000e+000 1.449454e+000

vertex 8.455345e+000 4.400000e+000 1.449454e+000

endloop

endfacet

facet normal 8.020838e-001 0.000000e+000 5.972114e-001

outer loop

vertex 8.455345e+000 4.400000e+000 1.449454e+000

vertex 8.455345e+000 3.000000e+000 1.449454e+000

vertex 8.505336e+000 3.000000e+000 1.378060e+000

endloop

endfacet

facet normal 8.355240e-001 0.000000e+000 5.494540e-001

outer loop

vertex 8.455345e+000 4.400000e+000 1.449454e+000

vertex 8.505336e+000 3.000000e+000 1.378060e+000

vertex 8.505336e+000 4.400000e+000 1.378060e+000

endloop

endfacet

facet normal 8.936031e-001 0.000000e+000 4.488580e-001

outer loop

vertex 8.505336e+000 4.400000e+000 1.378060e+000

vertex 8.505336e+000 3.000000e+000 1.378060e+000

vertex 8.542170e+000 3.000000e+000 1.299070e+000

endloop

endfacet

facet normal 9.182422e-001 0.000000e+000 3.960194e-001

outer loop

vertex 8.505336e+000 4.400000e+000 1.378060e+000

vertex 8.542170e+000 3.000000e+000 1.299070e+000

vertex 8.542170e+000 4.400000e+000 1.299070e+000

endloop

endfacet

facet normal 9.579706e-001 0.000000e+000 2.868663e-001

outer loop

vertex 8.542170e+000 4.400000e+000 1.299070e+000

vertex 8.542170e+000 3.000000e+000 1.299070e+000

vertex 8.564728e+000 3.000000e+000 1.214884e+000

endloop

endfacet

facet normal 9.730600e-001 0.000000e+000 2.305519e-001

outer loop

vertex 8.542170e+000 4.400000e+000 1.299070e+000

vertex 8.564728e+000 3.000000e+000 1.214884e+000

vertex 8.564728e+000 4.400000e+000 1.214884e+000

endloop

endfacet

facet normal 9.932307e-001 0.000000e+000 1.161583e-001

outer loop

vertex 8.564728e+000 4.400000e+000 1.214884e+000

vertex 8.564728e+000 3.000000e+000 1.214884e+000

vertex 8.572323e+000 3.000000e+000 1.128060e+000

endloop

endfacet

facet normal 9.983119e-001 0.000000e+000 5.807913e-002

outer loop

vertex 8.564728e+000 4.400000e+000 1.214884e+000

vertex 8.572323e+000 3.000000e+000 1.128060e+000

vertex 8.572323e+000 4.400000e+000 1.128060e+000

endloop

endfacet

facet normal 9.983119e-001 0.000000e+000 -5.807913e-002

outer loop

vertex 8.572323e+000 4.400000e+000 1.128060e+000

vertex 8.572323e+000 3.000000e+000 1.128060e+000

vertex 8.564728e+000 3.000000e+000 1.041236e+000

endloop

endfacet

facet normal 9.932307e-001 0.000000e+000 -1.161583e-001

outer loop

vertex 8.572323e+000 4.400000e+000 1.128060e+000

vertex 8.564728e+000 3.000000e+000 1.041236e+000

vertex 8.564728e+000 4.400000e+000 1.041236e+000

endloop

endfacet

facet normal 9.730600e-001 0.000000e+000 -2.305519e-001

outer loop

vertex 8.564728e+000 4.400000e+000 1.041236e+000

vertex 8.564728e+000 3.000000e+000 1.041236e+000

vertex 8.542170e+000 3.000000e+000 9.570499e-001

endloop

endfacet

facet normal 9.579706e-001 0.000000e+000 -2.868663e-001

outer loop

vertex 8.564728e+000 4.400000e+000 1.041236e+000

vertex 8.542170e+000 3.000000e+000 9.570499e-001

vertex 8.542170e+000 4.400000e+000 9.570499e-001

endloop

endfacet

facet normal 9.182422e-001 0.000000e+000 -3.960194e-001

outer loop

vertex 8.542170e+000 4.400000e+000 9.570499e-001

vertex 8.542170e+000 3.000000e+000 9.570499e-001

vertex 8.505336e+000 3.000000e+000 8.780602e-001

endloop

endfacet

facet normal 8.936031e-001 0.000000e+000 -4.488580e-001

outer loop

vertex 8.542170e+000 4.400000e+000 9.570499e-001

vertex 8.505336e+000 3.000000e+000 8.780602e-001

vertex 8.505336e+000 4.400000e+000 8.780602e-001

endloop

endfacet

facet normal 8.355240e-001 0.000000e+000 -5.494540e-001

outer loop

vertex 8.505336e+000 4.400000e+000 8.780602e-001

vertex 8.505336e+000 3.000000e+000 8.780602e-001

vertex 8.455345e+000 3.000000e+000 8.066660e-001

endloop

endfacet

facet normal 8.020838e-001 0.000000e+000 -5.972114e-001

outer loop

vertex 8.505336e+000 4.400000e+000 8.780602e-001

vertex 8.455345e+000 3.000000e+000 8.066660e-001

vertex 8.455345e+000 4.400000e+000 8.066660e-001

endloop

endfacet

facet normal 7.274188e-001 0.000000e+000 -6.861938e-001

outer loop

vertex 8.455345e+000 4.400000e+000 8.066660e-001

vertex 8.455345e+000 3.000000e+000 8.066660e-001

vertex 8.393718e+000 3.000000e+000 7.450376e-001

endloop

endfacet

facet normal 6.861938e-001 0.000000e+000 -7.274188e-001

outer loop

vertex 8.455345e+000 4.400000e+000 8.066660e-001

vertex 8.393718e+000 3.000000e+000 7.450376e-001

vertex 8.393718e+000 4.400000e+000 7.450376e-001

endloop

endfacet

facet normal 5.972114e-001 0.000000e+000 -8.020838e-001

outer loop

vertex 8.393718e+000 4.400000e+000 7.450376e-001

vertex 8.393718e+000 3.000000e+000 7.450376e-001

vertex 8.322324e+000 3.000000e+000 6.950474e-001

endloop

endfacet

facet normal 5.494540e-001 0.000000e+000 -8.355240e-001

outer loop

vertex 8.393718e+000 4.400000e+000 7.450376e-001

vertex 8.322324e+000 3.000000e+000 6.950474e-001

vertex 8.322324e+000 4.400000e+000 6.950474e-001

endloop

endfacet

facet normal 4.488580e-001 0.000000e+000 -8.936031e-001

outer loop

vertex 8.322324e+000 4.400000e+000 6.950474e-001

vertex 8.322324e+000 3.000000e+000 6.950474e-001

vertex 8.243334e+000 3.000000e+000 6.582136e-001

endloop

endfacet

facet normal 3.960194e-001 0.000000e+000 -9.182422e-001

outer loop

vertex 8.322324e+000 4.400000e+000 6.950474e-001

vertex 8.243334e+000 3.000000e+000 6.582136e-001

vertex 8.243334e+000 4.400000e+000 6.582136e-001

endloop

endfacet

facet normal 2.868663e-001 0.000000e+000 -9.579706e-001

outer loop

vertex 8.243334e+000 4.400000e+000 6.582136e-001

vertex 8.243334e+000 3.000000e+000 6.582136e-001

vertex 8.159147e+000 3.000000e+000 6.356561e-001

endloop

endfacet

facet normal 2.305519e-001 0.000000e+000 -9.730600e-001

outer loop

vertex 8.243334e+000 4.400000e+000 6.582136e-001

vertex 8.159147e+000 3.000000e+000 6.356561e-001

vertex 8.159147e+000 4.400000e+000 6.356561e-001

endloop

endfacet

facet normal 1.161583e-001 0.000000e+000 -9.932307e-001

outer loop

vertex 8.159147e+000 4.400000e+000 6.356561e-001

vertex 8.159147e+000 3.000000e+000 6.356561e-001

vertex 8.072324e+000 3.000000e+000 6.280602e-001

endloop

endfacet

facet normal 5.807913e-002 0.000000e+000 -9.983119e-001

outer loop

vertex 8.159147e+000 4.400000e+000 6.356561e-001

vertex 8.072324e+000 3.000000e+000 6.280602e-001

vertex 8.072324e+000 4.400000e+000 6.280602e-001

endloop

endfacet

facet normal -5.807913e-002 0.000000e+000 -9.983119e-001

outer loop

vertex 8.072324e+000 4.400000e+000 6.280602e-001

vertex 8.072324e+000 3.000000e+000 6.280602e-001

vertex 7.985500e+000 3.000000e+000 6.356561e-001

endloop

endfacet

facet normal -1.161583e-001 0.000000e+000 -9.932307e-001

outer loop

vertex 8.072324e+000 4.400000e+000 6.280602e-001

vertex 7.985500e+000 3.000000e+000 6.356561e-001

vertex 7.985500e+000 4.400000e+000 6.356561e-001

endloop

endfacet

facet normal -2.305519e-001 0.000000e+000 -9.730600e-001

outer loop

vertex 7.985500e+000 4.400000e+000 6.356561e-001

vertex 7.985500e+000 3.000000e+000 6.356561e-001

vertex 7.901314e+000 3.000000e+000 6.582136e-001

endloop

endfacet

facet normal -2.868663e-001 0.000000e+000 -9.579706e-001

outer loop

vertex 7.985500e+000 4.400000e+000 6.356561e-001

vertex 7.901314e+000 3.000000e+000 6.582136e-001

vertex 7.901314e+000 4.400000e+000 6.582136e-001

endloop

endfacet

facet normal -3.960194e-001 0.000000e+000 -9.182422e-001

outer loop

vertex 7.901314e+000 4.400000e+000 6.582136e-001

vertex 7.901314e+000 3.000000e+000 6.582136e-001

vertex 7.822324e+000 3.000000e+000 6.950474e-001

endloop

endfacet

facet normal -4.488580e-001 0.000000e+000 -8.936031e-001

outer loop

vertex 7.901314e+000 4.400000e+000 6.582136e-001

vertex 7.822324e+000 3.000000e+000 6.950474e-001

vertex 7.822324e+000 4.400000e+000 6.950474e-001

endloop

endfacet

facet normal -5.494540e-001 0.000000e+000 -8.355240e-001

outer loop

vertex 7.822324e+000 4.400000e+000 6.950474e-001

vertex 7.822324e+000 3.000000e+000 6.950474e-001

vertex 7.750930e+000 3.000000e+000 7.450376e-001

endloop

endfacet

facet normal -5.972114e-001 0.000000e+000 -8.020838e-001

outer loop

vertex 7.822324e+000 4.400000e+000 6.950474e-001

vertex 7.750930e+000 3.000000e+000 7.450376e-001

vertex 7.750930e+000 4.400000e+000 7.450376e-001

endloop

endfacet

facet normal -6.861938e-001 0.000000e+000 -7.274188e-001

outer loop

vertex 7.750930e+000 4.400000e+000 7.450376e-001

vertex 7.750930e+000 3.000000e+000 7.450376e-001

vertex 7.689301e+000 3.000000e+000 8.066660e-001

endloop

endfacet

facet normal -7.274188e-001 0.000000e+000 -6.861938e-001

outer loop

vertex 7.750930e+000 4.400000e+000 7.450376e-001

vertex 7.689301e+000 3.000000e+000 8.066660e-001

vertex 7.689301e+000 4.400000e+000 8.066660e-001

endloop

endfacet

facet normal -8.020838e-001 0.000000e+000 -5.972114e-001

outer loop

vertex 7.689301e+000 4.400000e+000 8.066660e-001

vertex 7.689301e+000 3.000000e+000 8.066660e-001

vertex 7.639311e+000 3.000000e+000 8.780602e-001

endloop

endfacet

facet normal -8.355240e-001 0.000000e+000 -5.494540e-001

outer loop

vertex 7.689301e+000 4.400000e+000 8.066660e-001

vertex 7.639311e+000 3.000000e+000 8.780602e-001

vertex 7.639311e+000 4.400000e+000 8.780602e-001

endloop

endfacet

facet normal -8.936031e-001 0.000000e+000 -4.488580e-001

outer loop

vertex 7.639311e+000 4.400000e+000 8.780602e-001

vertex 7.639311e+000 3.000000e+000 8.780602e-001

vertex 7.602478e+000 3.000000e+000 9.570499e-001

endloop

endfacet

facet normal -9.182422e-001 0.000000e+000 -3.960194e-001

outer loop

vertex 7.639311e+000 4.400000e+000 8.780602e-001

vertex 7.602478e+000 3.000000e+000 9.570499e-001

vertex 7.602478e+000 4.400000e+000 9.570499e-001

endloop

endfacet

facet normal -9.466226e-001 0.000000e+000 -3.223439e-001

outer loop

vertex 7.602478e+000 4.400000e+000 9.570499e-001

vertex 7.602478e+000 3.000000e+000 9.570499e-001

vertex 7.594380e+000 3.000000e+000 9.872695e-001

endloop

endfacet

facet normal -9.637589e-001 0.000000e+000 -2.667744e-001

outer loop

vertex 7.602478e+000 4.400000e+000 9.570499e-001

vertex 7.594380e+000 3.000000e+000 9.872695e-001

vertex 7.579920e+000 4.400000e+000 1.041236e+000

endloop

endfacet

facet normal -9.776480e-001 0.000000e+000 -2.102483e-001

outer loop

vertex 7.579920e+000 4.400000e+000 1.041236e+000

vertex 7.594380e+000 3.000000e+000 9.872695e-001

vertex 7.579920e+000 3.000000e+000 1.041236e+000

endloop

endfacet

facet normal -9.932307e-001 0.000000e+000 -1.161583e-001

outer loop

vertex 7.579920e+000 4.400000e+000 1.041236e+000

vertex 7.579920e+000 3.000000e+000 1.041236e+000

vertex 7.572323e+000 3.000000e+000 1.128060e+000

endloop

endfacet

facet normal 1.161583e-001 0.000000e+000 9.932307e-001

outer loop

vertex 1.078841e+001 4.400000e+000 4.860896e+000

vertex 1.087523e+001 3.000000e+000 4.853300e+000

vertex 1.087523e+001 4.400000e+000 4.853300e+000

endloop

endfacet

facet normal 2.305519e-001 0.000000e+000 9.730600e-001

outer loop

vertex 1.087523e+001 4.400000e+000 4.853300e+000

vertex 1.087523e+001 3.000000e+000 4.853300e+000

vertex 1.095942e+001 3.000000e+000 4.830742e+000

endloop

endfacet

facet normal 2.868663e-001 0.000000e+000 9.579706e-001

outer loop

vertex 1.087523e+001 4.400000e+000 4.853300e+000

vertex 1.095942e+001 3.000000e+000 4.830742e+000

vertex 1.095942e+001 4.400000e+000 4.830742e+000

endloop

endfacet

facet normal 3.960194e-001 0.000000e+000 9.182422e-001

outer loop

vertex 1.095942e+001 4.400000e+000 4.830742e+000

vertex 1.095942e+001 3.000000e+000 4.830742e+000

vertex 1.103841e+001 3.000000e+000 4.793909e+000

endloop

endfacet

facet normal 4.488580e-001 0.000000e+000 8.936031e-001

outer loop

vertex 1.095942e+001 4.400000e+000 4.830742e+000

vertex 1.103841e+001 3.000000e+000 4.793909e+000

vertex 1.103841e+001 4.400000e+000 4.793909e+000

endloop

endfacet

facet normal 5.494540e-001 0.000000e+000 8.355240e-001

outer loop

vertex 1.103841e+001 4.400000e+000 4.793909e+000

vertex 1.103841e+001 3.000000e+000 4.793909e+000

vertex 1.110980e+001 3.000000e+000 4.743918e+000

endloop

endfacet

facet normal 5.972114e-001 0.000000e+000 8.020838e-001

outer loop

vertex 1.103841e+001 4.400000e+000 4.793909e+000

vertex 1.110980e+001 3.000000e+000 4.743918e+000

vertex 1.110980e+001 4.400000e+000 4.743918e+000

endloop

endfacet

facet normal 6.861938e-001 0.000000e+000 7.274188e-001

outer loop

vertex 1.110980e+001 4.400000e+000 4.743918e+000

vertex 1.110980e+001 3.000000e+000 4.743918e+000

vertex 1.117143e+001 3.000000e+000 4.682290e+000

endloop

endfacet

facet normal 7.274188e-001 0.000000e+000 6.861938e-001

outer loop

vertex 1.110980e+001 4.400000e+000 4.743918e+000

vertex 1.117143e+001 3.000000e+000 4.682290e+000

vertex 1.117143e+001 4.400000e+000 4.682290e+000

endloop

endfacet

facet normal 8.020838e-001 0.000000e+000 5.972114e-001

outer loop

vertex 1.117143e+001 4.400000e+000 4.682290e+000

vertex 1.117143e+001 3.000000e+000 4.682290e+000

vertex 1.122142e+001 3.000000e+000 4.610896e+000

endloop

endfacet

facet normal 8.355240e-001 0.000000e+000 5.494540e-001

outer loop

vertex 1.117143e+001 4.400000e+000 4.682290e+000

vertex 1.122142e+001 3.000000e+000 4.610896e+000

vertex 1.122142e+001 4.400000e+000 4.610896e+000

endloop

endfacet

facet normal 8.936031e-001 0.000000e+000 4.488580e-001

outer loop

vertex 1.122142e+001 4.400000e+000 4.610896e+000

vertex 1.122142e+001 3.000000e+000 4.610896e+000

vertex 1.125826e+001 3.000000e+000 4.531906e+000

endloop

endfacet

facet normal 9.182422e-001 0.000000e+000 3.960194e-001

outer loop

vertex 1.122142e+001 4.400000e+000 4.610896e+000

vertex 1.125826e+001 3.000000e+000 4.531906e+000

vertex 1.125826e+001 4.400000e+000 4.531906e+000

endloop

endfacet

facet normal 9.579706e-001 0.000000e+000 2.868663e-001

outer loop

vertex 1.125826e+001 4.400000e+000 4.531906e+000

vertex 1.125826e+001 3.000000e+000 4.531906e+000

vertex 1.128081e+001 3.000000e+000 4.447720e+000

endloop

endfacet

facet normal 9.730600e-001 0.000000e+000 2.305519e-001

outer loop

vertex 1.125826e+001 4.400000e+000 4.531906e+000

vertex 1.128081e+001 3.000000e+000 4.447720e+000

vertex 1.128081e+001 4.400000e+000 4.447720e+000

endloop

endfacet

facet normal 9.932307e-001 0.000000e+000 1.161583e-001

outer loop

vertex 1.128081e+001 4.400000e+000 4.447720e+000

vertex 1.128081e+001 3.000000e+000 4.447720e+000

vertex 1.128841e+001 3.000000e+000 4.360896e+000

endloop

endfacet

facet normal 9.983119e-001 0.000000e+000 5.807913e-002

outer loop

vertex 1.128081e+001 4.400000e+000 4.447720e+000

vertex 1.128841e+001 3.000000e+000 4.360896e+000

vertex 1.128841e+001 4.400000e+000 4.360896e+000

endloop

endfacet

facet normal 9.983119e-001 0.000000e+000 -5.807913e-002

outer loop

vertex 1.128841e+001 4.400000e+000 4.360896e+000

vertex 1.128841e+001 3.000000e+000 4.360896e+000

vertex 1.128081e+001 3.000000e+000 4.274072e+000

endloop

endfacet

facet normal 9.932307e-001 0.000000e+000 -1.161583e-001

outer loop

vertex 1.128841e+001 4.400000e+000 4.360896e+000

vertex 1.128081e+001 3.000000e+000 4.274072e+000

vertex 1.128081e+001 4.400000e+000 4.274072e+000

endloop

endfacet

facet normal 9.730600e-001 0.000000e+000 -2.305519e-001

outer loop

vertex 1.128081e+001 4.400000e+000 4.274072e+000

vertex 1.128081e+001 3.000000e+000 4.274072e+000

vertex 1.125826e+001 3.000000e+000 4.189886e+000

endloop

endfacet

facet normal 9.579706e-001 0.000000e+000 -2.868663e-001

outer loop

vertex 1.128081e+001 4.400000e+000 4.274072e+000

vertex 1.125826e+001 3.000000e+000 4.189886e+000

vertex 1.125826e+001 4.400000e+000 4.189886e+000

endloop

endfacet

facet normal 9.182422e-001 0.000000e+000 -3.960194e-001

outer loop

vertex 1.125826e+001 4.400000e+000 4.189886e+000

vertex 1.125826e+001 3.000000e+000 4.189886e+000

vertex 1.122142e+001 3.000000e+000 4.110896e+000

endloop

endfacet

facet normal 8.936031e-001 0.000000e+000 -4.488580e-001

outer loop

vertex 1.125826e+001 4.400000e+000 4.189886e+000

vertex 1.122142e+001 3.000000e+000 4.110896e+000

vertex 1.122142e+001 4.400000e+000 4.110896e+000

endloop

endfacet

facet normal 8.355240e-001 0.000000e+000 -5.494540e-001

outer loop

vertex 1.122142e+001 4.400000e+000 4.110896e+000

vertex 1.122142e+001 3.000000e+000 4.110896e+000

vertex 1.117143e+001 3.000000e+000 4.039502e+000

endloop

endfacet

facet normal 8.020838e-001 0.000000e+000 -5.972114e-001

outer loop

vertex 1.122142e+001 4.400000e+000 4.110896e+000

vertex 1.117143e+001 3.000000e+000 4.039502e+000

vertex 1.117143e+001 4.400000e+000 4.039502e+000

endloop

endfacet

facet normal 7.274188e-001 0.000000e+000 -6.861938e-001

outer loop

vertex 1.117143e+001 4.400000e+000 4.039502e+000

vertex 1.117143e+001 3.000000e+000 4.039502e+000

vertex 1.110980e+001 3.000000e+000 3.977873e+000

endloop

endfacet

facet normal 6.861938e-001 0.000000e+000 -7.274188e-001

outer loop

vertex 1.117143e+001 4.400000e+000 4.039502e+000

vertex 1.110980e+001 3.000000e+000 3.977873e+000

vertex 1.110980e+001 4.400000e+000 3.977873e+000

endloop

endfacet

facet normal 5.972114e-001 0.000000e+000 -8.020838e-001

outer loop

vertex 1.110980e+001 4.400000e+000 3.977873e+000

vertex 1.110980e+001 3.000000e+000 3.977873e+000

vertex 1.103841e+001 3.000000e+000 3.927883e+000

endloop

endfacet

facet normal 5.494540e-001 0.000000e+000 -8.355240e-001

outer loop

vertex 1.110980e+001 4.400000e+000 3.977873e+000

vertex 1.103841e+001 3.000000e+000 3.927883e+000

vertex 1.103841e+001 4.400000e+000 3.927883e+000

endloop

endfacet

facet normal 4.488580e-001 0.000000e+000 -8.936031e-001

outer loop

vertex 1.103841e+001 4.400000e+000 3.927883e+000

vertex 1.103841e+001 3.000000e+000 3.927883e+000

vertex 1.095942e+001 3.000000e+000 3.891049e+000

endloop

endfacet

facet normal 3.960194e-001 0.000000e+000 -9.182422e-001

outer loop

vertex 1.103841e+001 4.400000e+000 3.927883e+000

vertex 1.095942e+001 3.000000e+000 3.891049e+000

vertex 1.095942e+001 4.400000e+000 3.891049e+000

endloop

endfacet

facet normal 2.868663e-001 0.000000e+000 -9.579706e-001

outer loop

vertex 1.095942e+001 4.400000e+000 3.891049e+000

vertex 1.095942e+001 3.000000e+000 3.891049e+000

vertex 1.087523e+001 3.000000e+000 3.868492e+000

endloop

endfacet

facet normal 2.305519e-001 0.000000e+000 -9.730600e-001

outer loop

vertex 1.095942e+001 4.400000e+000 3.891049e+000

vertex 1.087523e+001 3.000000e+000 3.868492e+000

vertex 1.087523e+001 4.400000e+000 3.868492e+000

endloop

endfacet

facet normal 1.161583e-001 0.000000e+000 -9.932307e-001

outer loop

vertex 1.087523e+001 4.400000e+000 3.868492e+000

vertex 1.087523e+001 3.000000e+000 3.868492e+000

vertex 1.078841e+001 3.000000e+000 3.860896e+000

endloop

endfacet

facet normal 5.807913e-002 0.000000e+000 -9.983119e-001

outer loop

vertex 1.087523e+001 4.400000e+000 3.868492e+000

vertex 1.078841e+001 3.000000e+000 3.860896e+000

vertex 1.078841e+001 4.400000e+000 3.860896e+000

endloop

endfacet

facet normal -5.807913e-002 0.000000e+000 -9.983119e-001

outer loop

vertex 1.078841e+001 4.400000e+000 3.860896e+000

vertex 1.078841e+001 3.000000e+000 3.860896e+000

vertex 1.070159e+001 3.000000e+000 3.868492e+000

endloop

endfacet

facet normal -1.161583e-001 0.000000e+000 -9.932307e-001

outer loop

vertex 1.078841e+001 4.400000e+000 3.860896e+000

vertex 1.070159e+001 3.000000e+000 3.868492e+000

vertex 1.070159e+001 4.400000e+000 3.868492e+000

endloop

endfacet

facet normal -2.305519e-001 0.000000e+000 -9.730600e-001

outer loop

vertex 1.070159e+001 4.400000e+000 3.868492e+000

vertex 1.070159e+001 3.000000e+000 3.868492e+000

vertex 1.061740e+001 3.000000e+000 3.891049e+000

endloop

endfacet

facet normal -2.868663e-001 0.000000e+000 -9.579706e-001

outer loop

vertex 1.070159e+001 4.400000e+000 3.868492e+000

vertex 1.061740e+001 3.000000e+000 3.891049e+000

vertex 1.061740e+001 4.400000e+000 3.891049e+000

endloop

endfacet

facet normal -3.960194e-001 0.000000e+000 -9.182422e-001

outer loop

vertex 1.061740e+001 4.400000e+000 3.891049e+000

vertex 1.061740e+001 3.000000e+000 3.891049e+000

vertex 1.053841e+001 3.000000e+000 3.927883e+000

endloop

endfacet

facet normal -4.488580e-001 0.000000e+000 -8.936031e-001

outer loop

vertex 1.061740e+001 4.400000e+000 3.891049e+000

vertex 1.053841e+001 3.000000e+000 3.927883e+000

vertex 1.053841e+001 4.400000e+000 3.927883e+000

endloop

endfacet

facet normal -5.494540e-001 0.000000e+000 -8.355240e-001

outer loop

vertex 1.053841e+001 4.400000e+000 3.927883e+000

vertex 1.053841e+001 3.000000e+000 3.927883e+000

vertex 1.046702e+001 3.000000e+000 3.977873e+000

endloop

endfacet

facet normal -5.972114e-001 0.000000e+000 -8.020838e-001

outer loop

vertex 1.053841e+001 4.400000e+000 3.927883e+000

vertex 1.046702e+001 3.000000e+000 3.977873e+000

vertex 1.046702e+001 4.400000e+000 3.977873e+000

endloop

endfacet

facet normal -6.861938e-001 0.000000e+000 -7.274188e-001

outer loop

vertex 1.046702e+001 4.400000e+000 3.977873e+000

vertex 1.046702e+001 3.000000e+000 3.977873e+000

vertex 1.040539e+001 3.000000e+000 4.039502e+000

endloop

endfacet

facet normal -7.274188e-001 0.000000e+000 -6.861938e-001

outer loop

vertex 1.046702e+001 4.400000e+000 3.977873e+000

vertex 1.040539e+001 3.000000e+000 4.039502e+000

vertex 1.040539e+001 4.400000e+000 4.039502e+000

endloop

endfacet

facet normal -8.020838e-001 0.000000e+000 -5.972114e-001

outer loop

vertex 1.040539e+001 4.400000e+000 4.039502e+000

vertex 1.040539e+001 3.000000e+000 4.039502e+000

vertex 1.035540e+001 3.000000e+000 4.110896e+000

endloop

endfacet

facet normal -8.355240e-001 0.000000e+000 -5.494540e-001

outer loop

vertex 1.040539e+001 4.400000e+000 4.039502e+000

vertex 1.035540e+001 3.000000e+000 4.110896e+000

vertex 1.035540e+001 4.400000e+000 4.110896e+000

endloop

endfacet

facet normal -8.936031e-001 0.000000e+000 -4.488580e-001

outer loop

vertex 1.035540e+001 4.400000e+000 4.110896e+000

vertex 1.035540e+001 3.000000e+000 4.110896e+000

vertex 1.031856e+001 3.000000e+000 4.189886e+000

endloop

endfacet

facet normal -9.182422e-001 0.000000e+000 -3.960194e-001

outer loop

vertex 1.035540e+001 4.400000e+000 4.110896e+000

vertex 1.031856e+001 3.000000e+000 4.189886e+000

vertex 1.031856e+001 4.400000e+000 4.189886e+000

endloop

endfacet

facet normal -9.579706e-001 0.000000e+000 -2.868663e-001

outer loop

vertex 1.031856e+001 4.400000e+000 4.189886e+000

vertex 1.031856e+001 3.000000e+000 4.189886e+000

vertex 1.029601e+001 3.000000e+000 4.274072e+000

endloop

endfacet

facet normal -9.730600e-001 0.000000e+000 -2.305519e-001

outer loop

vertex 1.031856e+001 4.400000e+000 4.189886e+000

vertex 1.029601e+001 3.000000e+000 4.274072e+000

vertex 1.029601e+001 4.400000e+000 4.274072e+000

endloop

endfacet

facet normal -9.932307e-001 0.000000e+000 -1.161583e-001

outer loop

vertex 1.029601e+001 4.400000e+000 4.274072e+000

vertex 1.029601e+001 3.000000e+000 4.274072e+000

vertex 1.028841e+001 3.000000e+000 4.360896e+000

endloop

endfacet

facet normal -9.983119e-001 0.000000e+000 -5.807913e-002

outer loop

vertex 1.029601e+001 4.400000e+000 4.274072e+000

vertex 1.028841e+001 3.000000e+000 4.360896e+000

vertex 1.028841e+001 4.400000e+000 4.360896e+000

endloop

endfacet

facet normal -9.983119e-001 0.000000e+000 5.807913e-002

outer loop

vertex 1.028841e+001 4.400000e+000 4.360896e+000

vertex 1.028841e+001 3.000000e+000 4.360896e+000

vertex 1.029601e+001 3.000000e+000 4.447720e+000

endloop

endfacet

facet normal -9.932307e-001 0.000000e+000 1.161583e-001

outer loop

vertex 1.028841e+001 4.400000e+000 4.360896e+000

vertex 1.029601e+001 3.000000e+000 4.447720e+000

vertex 1.029601e+001 4.400000e+000 4.447720e+000

endloop

endfacet

facet normal -9.730600e-001 0.000000e+000 2.305519e-001

outer loop

vertex 1.029601e+001 4.400000e+000 4.447720e+000

vertex 1.029601e+001 3.000000e+000 4.447720e+000

vertex 1.031856e+001 3.000000e+000 4.531906e+000

endloop

endfacet

facet normal -9.579706e-001 0.000000e+000 2.868663e-001

outer loop

vertex 1.029601e+001 4.400000e+000 4.447720e+000

vertex 1.031856e+001 3.000000e+000 4.531906e+000

vertex 1.031856e+001 4.400000e+000 4.531906e+000

endloop

endfacet

facet normal -9.182422e-001 0.000000e+000 3.960194e-001

outer loop

vertex 1.031856e+001 4.400000e+000 4.531906e+000

vertex 1.031856e+001 3.000000e+000 4.531906e+000

vertex 1.035540e+001 3.000000e+000 4.610896e+000

endloop

endfacet

facet normal -8.936031e-001 0.000000e+000 4.488580e-001

outer loop

vertex 1.031856e+001 4.400000e+000 4.531906e+000

vertex 1.035540e+001 3.000000e+000 4.610896e+000

vertex 1.035540e+001 4.400000e+000 4.610896e+000

endloop

endfacet

facet normal -8.355240e-001 0.000000e+000 5.494540e-001

outer loop

vertex 1.035540e+001 4.400000e+000 4.610896e+000

vertex 1.035540e+001 3.000000e+000 4.610896e+000

vertex 1.040539e+001 3.000000e+000 4.682290e+000

endloop

endfacet

facet normal -8.020838e-001 0.000000e+000 5.972114e-001

outer loop

vertex 1.035540e+001 4.400000e+000 4.610896e+000

vertex 1.040539e+001 3.000000e+000 4.682290e+000

vertex 1.040539e+001 4.400000e+000 4.682290e+000

endloop

endfacet

facet normal -7.274188e-001 0.000000e+000 6.861938e-001

outer loop

vertex 1.040539e+001 4.400000e+000 4.682290e+000

vertex 1.040539e+001 3.000000e+000 4.682290e+000

vertex 1.046702e+001 3.000000e+000 4.743918e+000

endloop

endfacet

facet normal -6.861938e-001 0.000000e+000 7.274188e-001

outer loop

vertex 1.040539e+001 4.400000e+000 4.682290e+000

vertex 1.046702e+001 3.000000e+000 4.743918e+000

vertex 1.046702e+001 4.400000e+000 4.743918e+000

endloop

endfacet

facet normal -5.972114e-001 0.000000e+000 8.020838e-001

outer loop

vertex 1.046702e+001 4.400000e+000 4.743918e+000

vertex 1.046702e+001 3.000000e+000 4.743918e+000

vertex 1.053841e+001 3.000000e+000 4.793909e+000

endloop

endfacet

facet normal -5.494540e-001 0.000000e+000 8.355240e-001

outer loop

vertex 1.046702e+001 4.400000e+000 4.743918e+000

vertex 1.053841e+001 3.000000e+000 4.793909e+000

vertex 1.053841e+001 4.400000e+000 4.793909e+000

endloop

endfacet

facet normal -4.488580e-001 0.000000e+000 8.936031e-001

outer loop

vertex 1.053841e+001 4.400000e+000 4.793909e+000

vertex 1.053841e+001 3.000000e+000 4.793909e+000

vertex 1.061740e+001 3.000000e+000 4.830742e+000

endloop

endfacet

facet normal -3.960194e-001 0.000000e+000 9.182422e-001

outer loop

vertex 1.053841e+001 4.400000e+000 4.793909e+000

vertex 1.061740e+001 3.000000e+000 4.830742e+000

vertex 1.061740e+001 4.400000e+000 4.830742e+000

endloop

endfacet

facet normal -2.868663e-001 0.000000e+000 9.579706e-001

outer loop

vertex 1.061740e+001 4.400000e+000 4.830742e+000

vertex 1.061740e+001 3.000000e+000 4.830742e+000

vertex 1.070159e+001 3.000000e+000 4.853300e+000

endloop

endfacet

facet normal -2.305519e-001 0.000000e+000 9.730600e-001

outer loop

vertex 1.061740e+001 4.400000e+000 4.830742e+000

vertex 1.070159e+001 3.000000e+000 4.853300e+000

vertex 1.070159e+001 4.400000e+000 4.853300e+000

endloop

endfacet

facet normal -1.161583e-001 0.000000e+000 9.932307e-001

outer loop

vertex 1.070159e+001 4.400000e+000 4.853300e+000

vertex 1.070159e+001 3.000000e+000 4.853300e+000

vertex 1.078841e+001 3.000000e+000 4.860896e+000

endloop

endfacet

facet normal -5.807913e-002 0.000000e+000 9.983119e-001

outer loop

vertex 1.070159e+001 4.400000e+000 4.853300e+000

vertex 1.078841e+001 3.000000e+000 4.860896e+000

vertex 1.078841e+001 4.400000e+000 4.860896e+000

endloop

endfacet

facet normal 5.807913e-002 0.000000e+000 9.983119e-001

outer loop

vertex 1.078841e+001 4.400000e+000 4.860896e+000

vertex 1.078841e+001 3.000000e+000 4.860896e+000

vertex 1.087523e+001 3.000000e+000 4.853300e+000

endloop

endfacet

facet normal 1.161583e-001 0.000000e+000 9.932307e-001

outer loop

vertex 8.116436e+000 4.400000e+000 4.860896e+000

vertex 8.203260e+000 3.000000e+000 4.853300e+000

vertex 8.203260e+000 4.400000e+000 4.853300e+000

endloop

endfacet

facet normal 2.305519e-001 0.000000e+000 9.730600e-001

outer loop

vertex 8.203260e+000 4.400000e+000 4.853300e+000

vertex 8.203260e+000 3.000000e+000 4.853300e+000

vertex 8.287447e+000 3.000000e+000 4.830742e+000

endloop

endfacet

facet normal 2.868663e-001 0.000000e+000 9.579706e-001

outer loop

vertex 8.203260e+000 4.400000e+000 4.853300e+000

vertex 8.287447e+000 3.000000e+000 4.830742e+000

vertex 8.287447e+000 4.400000e+000 4.830742e+000

endloop

endfacet

facet normal 3.960194e-001 0.000000e+000 9.182422e-001

outer loop

vertex 8.287447e+000 4.400000e+000 4.830742e+000

vertex 8.287447e+000 3.000000e+000 4.830742e+000

vertex 8.366437e+000 3.000000e+000 4.793909e+000

endloop

endfacet

facet normal 4.488580e-001 0.000000e+000 8.936031e-001

outer loop

vertex 8.287447e+000 4.400000e+000 4.830742e+000

vertex 8.366437e+000 3.000000e+000 4.793909e+000

vertex 8.366437e+000 4.400000e+000 4.793909e+000

endloop

endfacet

facet normal 5.494540e-001 0.000000e+000 8.355240e-001

outer loop

vertex 8.366437e+000 4.400000e+000 4.793909e+000

vertex 8.366437e+000 3.000000e+000 4.793909e+000

vertex 8.437830e+000 3.000000e+000 4.743918e+000

endloop

endfacet

facet normal 5.972114e-001 0.000000e+000 8.020838e-001

outer loop

vertex 8.366437e+000 4.400000e+000 4.793909e+000

vertex 8.437830e+000 3.000000e+000 4.743918e+000

vertex 8.437830e+000 4.400000e+000 4.743918e+000

endloop

endfacet

facet normal 6.861938e-001 0.000000e+000 7.274188e-001

outer loop

vertex 8.437830e+000 4.400000e+000 4.743918e+000

vertex 8.437830e+000 3.000000e+000 4.743918e+000

vertex 8.499458e+000 3.000000e+000 4.682290e+000

endloop

endfacet

facet normal 7.274188e-001 0.000000e+000 6.861938e-001

outer loop

vertex 8.437830e+000 4.400000e+000 4.743918e+000

vertex 8.499458e+000 3.000000e+000 4.682290e+000

vertex 8.499458e+000 4.400000e+000 4.682290e+000

endloop

endfacet

facet normal 8.020838e-001 0.000000e+000 5.972114e-001

outer loop

vertex 8.499458e+000 4.400000e+000 4.682290e+000

vertex 8.499458e+000 3.000000e+000 4.682290e+000

vertex 8.549449e+000 3.000000e+000 4.610896e+000

endloop

endfacet

facet normal 8.355240e-001 0.000000e+000 5.494540e-001

outer loop

vertex 8.499458e+000 4.400000e+000 4.682290e+000

vertex 8.549449e+000 3.000000e+000 4.610896e+000

vertex 8.549449e+000 4.400000e+000 4.610896e+000

endloop

endfacet

facet normal 8.936031e-001 0.000000e+000 4.488580e-001

outer loop

vertex 8.549449e+000 4.400000e+000 4.610896e+000

vertex 8.549449e+000 3.000000e+000 4.610896e+000

vertex 8.586283e+000 3.000000e+000 4.531906e+000

endloop

endfacet

facet normal 9.182422e-001 0.000000e+000 3.960194e-001

outer loop

vertex 8.549449e+000 4.400000e+000 4.610896e+000

vertex 8.586283e+000 3.000000e+000 4.531906e+000

vertex 8.586283e+000 4.400000e+000 4.531906e+000

endloop

endfacet

facet normal 9.579706e-001 0.000000e+000 2.868663e-001

outer loop

vertex 8.586283e+000 4.400000e+000 4.531906e+000

vertex 8.586283e+000 3.000000e+000 4.531906e+000

vertex 8.608840e+000 3.000000e+000 4.447720e+000

endloop

endfacet

facet normal 9.730600e-001 0.000000e+000 2.305519e-001

outer loop

vertex 8.586283e+000 4.400000e+000 4.531906e+000

vertex 8.608840e+000 3.000000e+000 4.447720e+000

vertex 8.608840e+000 4.400000e+000 4.447720e+000

endloop

endfacet

facet normal 9.932307e-001 0.000000e+000 1.161583e-001

outer loop

vertex 8.608840e+000 4.400000e+000 4.447720e+000

vertex 8.608840e+000 3.000000e+000 4.447720e+000

vertex 8.616436e+000 3.000000e+000 4.360896e+000

endloop

endfacet

facet normal 9.983119e-001 0.000000e+000 5.807913e-002

outer loop

vertex 8.608840e+000 4.400000e+000 4.447720e+000

vertex 8.616436e+000 3.000000e+000 4.360896e+000

vertex 8.616436e+000 4.400000e+000 4.360896e+000

endloop

endfacet

facet normal 9.983119e-001 0.000000e+000 -5.807913e-002

outer loop

vertex 8.616436e+000 4.400000e+000 4.360896e+000

vertex 8.616436e+000 3.000000e+000 4.360896e+000

vertex 8.608840e+000 3.000000e+000 4.274072e+000

endloop

endfacet

facet normal 9.932307e-001 0.000000e+000 -1.161583e-001

outer loop

vertex 8.616436e+000 4.400000e+000 4.360896e+000

vertex 8.608840e+000 3.000000e+000 4.274072e+000

vertex 8.608840e+000 4.400000e+000 4.274072e+000

endloop

endfacet

facet normal 9.730600e-001 0.000000e+000 -2.305519e-001

outer loop

vertex 8.608840e+000 4.400000e+000 4.274072e+000

vertex 8.608840e+000 3.000000e+000 4.274072e+000

vertex 8.586283e+000 3.000000e+000 4.189886e+000

endloop

endfacet

facet normal 9.579706e-001 0.000000e+000 -2.868663e-001

outer loop

vertex 8.608840e+000 4.400000e+000 4.274072e+000

vertex 8.586283e+000 3.000000e+000 4.189886e+000

vertex 8.586283e+000 4.400000e+000 4.189886e+000

endloop

endfacet

facet normal 9.182422e-001 0.000000e+000 -3.960194e-001

outer loop

vertex 8.586283e+000 4.400000e+000 4.189886e+000

vertex 8.586283e+000 3.000000e+000 4.189886e+000

vertex 8.549449e+000 3.000000e+000 4.110896e+000

endloop

endfacet

facet normal 8.936031e-001 0.000000e+000 -4.488580e-001

outer loop

vertex 8.586283e+000 4.400000e+000 4.189886e+000

vertex 8.549449e+000 3.000000e+000 4.110896e+000

vertex 8.549449e+000 4.400000e+000 4.110896e+000

endloop

endfacet

facet normal 8.355240e-001 0.000000e+000 -5.494540e-001

outer loop

vertex 8.549449e+000 4.400000e+000 4.110896e+000

vertex 8.549449e+000 3.000000e+000 4.110896e+000

vertex 8.499458e+000 3.000000e+000 4.039502e+000

endloop

endfacet

facet normal 8.020838e-001 0.000000e+000 -5.972114e-001

outer loop

vertex 8.549449e+000 4.400000e+000 4.110896e+000

vertex 8.499458e+000 3.000000e+000 4.039502e+000

vertex 8.499458e+000 4.400000e+000 4.039502e+000

endloop

endfacet

facet normal 7.274188e-001 0.000000e+000 -6.861938e-001

outer loop

vertex 8.499458e+000 4.400000e+000 4.039502e+000

vertex 8.499458e+000 3.000000e+000 4.039502e+000

vertex 8.437830e+000 3.000000e+000 3.977873e+000

endloop

endfacet

facet normal 6.861938e-001 0.000000e+000 -7.274188e-001

outer loop

vertex 8.499458e+000 4.400000e+000 4.039502e+000

vertex 8.437830e+000 3.000000e+000 3.977873e+000

vertex 8.437830e+000 4.400000e+000 3.977873e+000

endloop

endfacet

facet normal 5.972114e-001 0.000000e+000 -8.020838e-001

outer loop

vertex 8.437830e+000 4.400000e+000 3.977873e+000

vertex 8.437830e+000 3.000000e+000 3.977873e+000

vertex 8.366437e+000 3.000000e+000 3.927883e+000

endloop

endfacet

facet normal 5.494540e-001 0.000000e+000 -8.355240e-001

outer loop

vertex 8.437830e+000 4.400000e+000 3.977873e+000

vertex 8.366437e+000 3.000000e+000 3.927883e+000

vertex 8.366437e+000 4.400000e+000 3.927883e+000

endloop

endfacet

facet normal 4.488580e-001 0.000000e+000 -8.936031e-001

outer loop

vertex 8.366437e+000 4.400000e+000 3.927883e+000

vertex 8.366437e+000 3.000000e+000 3.927883e+000

vertex 8.287447e+000 3.000000e+000 3.891049e+000

endloop

endfacet

facet normal 3.960194e-001 0.000000e+000 -9.182422e-001

outer loop

vertex 8.366437e+000 4.400000e+000 3.927883e+000

vertex 8.287447e+000 3.000000e+000 3.891049e+000

vertex 8.287447e+000 4.400000e+000 3.891049e+000

endloop

endfacet

facet normal 2.868663e-001 0.000000e+000 -9.579706e-001

outer loop

vertex 8.287447e+000 4.400000e+000 3.891049e+000

vertex 8.287447e+000 3.000000e+000 3.891049e+000

vertex 8.203260e+000 3.000000e+000 3.868492e+000

endloop

endfacet

facet normal 2.305519e-001 0.000000e+000 -9.730600e-001

outer loop

vertex 8.287447e+000 4.400000e+000 3.891049e+000

vertex 8.203260e+000 3.000000e+000 3.868492e+000

vertex 8.203260e+000 4.400000e+000 3.868492e+000

endloop

endfacet

facet normal 1.161583e-001 0.000000e+000 -9.932307e-001

outer loop

vertex 8.203260e+000 4.400000e+000 3.868492e+000

vertex 8.203260e+000 3.000000e+000 3.868492e+000

vertex 8.116436e+000 3.000000e+000 3.860896e+000

endloop

endfacet

facet normal 5.807913e-002 0.000000e+000 -9.983119e-001

outer loop

vertex 8.203260e+000 4.400000e+000 3.868492e+000

vertex 8.116436e+000 3.000000e+000 3.860896e+000

vertex 8.116436e+000 4.400000e+000 3.860896e+000

endloop

endfacet

facet normal -5.807913e-002 0.000000e+000 -9.983119e-001

outer loop

vertex 8.116436e+000 4.400000e+000 3.860896e+000

vertex 8.116436e+000 3.000000e+000 3.860896e+000

vertex 8.029613e+000 3.000000e+000 3.868492e+000

endloop

endfacet

facet normal -1.161583e-001 0.000000e+000 -9.932307e-001

outer loop

vertex 8.116436e+000 4.400000e+000 3.860896e+000

vertex 8.029613e+000 3.000000e+000 3.868492e+000

vertex 8.029613e+000 4.400000e+000 3.868492e+000

endloop

endfacet

facet normal -2.305519e-001 0.000000e+000 -9.730600e-001

outer loop

vertex 8.029613e+000 4.400000e+000 3.868492e+000

vertex 8.029613e+000 3.000000e+000 3.868492e+000

vertex 7.945427e+000 3.000000e+000 3.891049e+000

endloop

endfacet

facet normal -2.868663e-001 0.000000e+000 -9.579706e-001

outer loop

vertex 8.029613e+000 4.400000e+000 3.868492e+000

vertex 7.945427e+000 3.000000e+000 3.891049e+000

vertex 7.945427e+000 4.400000e+000 3.891049e+000

endloop

endfacet

facet normal -3.960194e-001 0.000000e+000 -9.182422e-001

outer loop

vertex 7.945427e+000 4.400000e+000 3.891049e+000

vertex 7.945427e+000 3.000000e+000 3.891049e+000

vertex 7.866436e+000 3.000000e+000 3.927883e+000

endloop

endfacet

facet normal -4.488580e-001 0.000000e+000 -8.936031e-001

outer loop

vertex 7.945427e+000 4.400000e+000 3.891049e+000

vertex 7.866436e+000 3.000000e+000 3.927883e+000

vertex 7.866436e+000 4.400000e+000 3.927883e+000

endloop

endfacet

facet normal -5.494540e-001 0.000000e+000 -8.355240e-001

outer loop

vertex 7.866436e+000 4.400000e+000 3.927883e+000

vertex 7.866436e+000 3.000000e+000 3.927883e+000

vertex 7.795043e+000 3.000000e+000 3.977873e+000

endloop

endfacet

facet normal -5.972114e-001 0.000000e+000 -8.020838e-001

outer loop

vertex 7.866436e+000 4.400000e+000 3.927883e+000

vertex 7.795043e+000 3.000000e+000 3.977873e+000

vertex 7.795043e+000 4.400000e+000 3.977873e+000

endloop

endfacet

facet normal -6.861938e-001 0.000000e+000 -7.274188e-001

outer loop

vertex 7.795043e+000 4.400000e+000 3.977873e+000

vertex 7.795043e+000 3.000000e+000 3.977873e+000

vertex 7.733414e+000 3.000000e+000 4.039502e+000

endloop

endfacet

facet normal -7.274188e-001 0.000000e+000 -6.861938e-001

outer loop

vertex 7.795043e+000 4.400000e+000 3.977873e+000

vertex 7.733414e+000 3.000000e+000 4.039502e+000

vertex 7.733414e+000 4.400000e+000 4.039502e+000

endloop

endfacet

facet normal -8.020838e-001 0.000000e+000 -5.972114e-001

outer loop

vertex 7.733414e+000 4.400000e+000 4.039502e+000

vertex 7.733414e+000 3.000000e+000 4.039502e+000

vertex 7.683424e+000 3.000000e+000 4.110896e+000

endloop

endfacet

facet normal -8.355240e-001 0.000000e+000 -5.494540e-001

outer loop

vertex 7.733414e+000 4.400000e+000 4.039502e+000

vertex 7.683424e+000 3.000000e+000 4.110896e+000

vertex 7.683424e+000 4.400000e+000 4.110896e+000

endloop

endfacet

facet normal -8.936031e-001 0.000000e+000 -4.488580e-001

outer loop

vertex 7.683424e+000 4.400000e+000 4.110896e+000

vertex 7.683424e+000 3.000000e+000 4.110896e+000

vertex 7.646590e+000 3.000000e+000 4.189886e+000

endloop

endfacet

facet normal -9.182422e-001 0.000000e+000 -3.960194e-001

outer loop

vertex 7.683424e+000 4.400000e+000 4.110896e+000

vertex 7.646590e+000 3.000000e+000 4.189886e+000

vertex 7.646590e+000 4.400000e+000 4.189886e+000

endloop

endfacet

facet normal -9.579706e-001 0.000000e+000 -2.868663e-001

outer loop

vertex 7.646590e+000 4.400000e+000 4.189886e+000

vertex 7.646590e+000 3.000000e+000 4.189886e+000

vertex 7.624032e+000 3.000000e+000 4.274072e+000

endloop

endfacet

facet normal -9.730600e-001 0.000000e+000 -2.305519e-001

outer loop

vertex 7.646590e+000 4.400000e+000 4.189886e+000

vertex 7.624032e+000 3.000000e+000 4.274072e+000

vertex 7.624032e+000 4.400000e+000 4.274072e+000

endloop

endfacet

facet normal -9.932307e-001 0.000000e+000 -1.161583e-001

outer loop

vertex 7.624032e+000 4.400000e+000 4.274072e+000

vertex 7.624032e+000 3.000000e+000 4.274072e+000

vertex 7.616436e+000 3.000000e+000 4.360896e+000

endloop

endfacet

facet normal -9.983119e-001 0.000000e+000 -5.807913e-002

outer loop

vertex 7.624032e+000 4.400000e+000 4.274072e+000

vertex 7.616436e+000 3.000000e+000 4.360896e+000

vertex 7.616436e+000 4.400000e+000 4.360896e+000

endloop

endfacet

facet normal -9.983119e-001 0.000000e+000 5.807913e-002

outer loop

vertex 7.616436e+000 4.400000e+000 4.360896e+000

vertex 7.616436e+000 3.000000e+000 4.360896e+000

vertex 7.624032e+000 3.000000e+000 4.447720e+000

endloop

endfacet

facet normal -9.932307e-001 0.000000e+000 1.161583e-001

outer loop

vertex 7.616436e+000 4.400000e+000 4.360896e+000

vertex 7.624032e+000 3.000000e+000 4.447720e+000

vertex 7.624032e+000 4.400000e+000 4.447720e+000

endloop

endfacet

facet normal -9.730600e-001 0.000000e+000 2.305519e-001

outer loop

vertex 7.624032e+000 4.400000e+000 4.447720e+000

vertex 7.624032e+000 3.000000e+000 4.447720e+000

vertex 7.646590e+000 3.000000e+000 4.531906e+000

endloop

endfacet

facet normal -9.579706e-001 0.000000e+000 2.868663e-001

outer loop

vertex 7.624032e+000 4.400000e+000 4.447720e+000

vertex 7.646590e+000 3.000000e+000 4.531906e+000

vertex 7.646590e+000 4.400000e+000 4.531906e+000

endloop

endfacet

facet normal -9.182422e-001 0.000000e+000 3.960194e-001

outer loop

vertex 7.646590e+000 4.400000e+000 4.531906e+000

vertex 7.646590e+000 3.000000e+000 4.531906e+000

vertex 7.683424e+000 3.000000e+000 4.610896e+000

endloop

endfacet

facet normal -8.936031e-001 0.000000e+000 4.488580e-001

outer loop

vertex 7.646590e+000 4.400000e+000 4.531906e+000

vertex 7.683424e+000 3.000000e+000 4.610896e+000

vertex 7.683424e+000 4.400000e+000 4.610896e+000

endloop

endfacet

facet normal -8.355240e-001 0.000000e+000 5.494540e-001

outer loop

vertex 7.683424e+000 4.400000e+000 4.610896e+000

vertex 7.683424e+000 3.000000e+000 4.610896e+000

vertex 7.733414e+000 3.000000e+000 4.682290e+000

endloop

endfacet

facet normal -8.020838e-001 0.000000e+000 5.972114e-001

outer loop

vertex 7.683424e+000 4.400000e+000 4.610896e+000

vertex 7.733414e+000 3.000000e+000 4.682290e+000

vertex 7.733414e+000 4.400000e+000 4.682290e+000

endloop

endfacet

facet normal -7.274188e-001 0.000000e+000 6.861938e-001

outer loop

vertex 7.733414e+000 4.400000e+000 4.682290e+000

vertex 7.733414e+000 3.000000e+000 4.682290e+000

vertex 7.795043e+000 3.000000e+000 4.743918e+000

endloop

endfacet

facet normal -6.861938e-001 0.000000e+000 7.274188e-001

outer loop

vertex 7.733414e+000 4.400000e+000 4.682290e+000

vertex 7.795043e+000 3.000000e+000 4.743918e+000

vertex 7.795043e+000 4.400000e+000 4.743918e+000

endloop

endfacet

facet normal -5.972114e-001 0.000000e+000 8.020838e-001

outer loop

vertex 7.795043e+000 4.400000e+000 4.743918e+000

vertex 7.795043e+000 3.000000e+000 4.743918e+000

vertex 7.866436e+000 3.000000e+000 4.793909e+000

endloop

endfacet

facet normal -5.494540e-001 0.000000e+000 8.355240e-001

outer loop

vertex 7.795043e+000 4.400000e+000 4.743918e+000

vertex 7.866436e+000 3.000000e+000 4.793909e+000

vertex 7.866436e+000 4.400000e+000 4.793909e+000

endloop

endfacet

facet normal -4.488580e-001 0.000000e+000 8.936031e-001

outer loop

vertex 7.866436e+000 4.400000e+000 4.793909e+000

vertex 7.866436e+000 3.000000e+000 4.793909e+000

vertex 7.945427e+000 3.000000e+000 4.830742e+000

endloop

endfacet

facet normal -3.960194e-001 0.000000e+000 9.182422e-001

outer loop

vertex 7.866436e+000 4.400000e+000 4.793909e+000

vertex 7.945427e+000 3.000000e+000 4.830742e+000

vertex 7.945427e+000 4.400000e+000 4.830742e+000

endloop

endfacet

facet normal -2.868663e-001 0.000000e+000 9.579706e-001

outer loop

vertex 7.945427e+000 4.400000e+000 4.830742e+000

vertex 7.945427e+000 3.000000e+000 4.830742e+000

vertex 8.029613e+000 3.000000e+000 4.853300e+000

endloop

endfacet

facet normal -2.305519e-001 0.000000e+000 9.730600e-001

outer loop

vertex 7.945427e+000 4.400000e+000 4.830742e+000

vertex 8.029613e+000 3.000000e+000 4.853300e+000

vertex 8.029613e+000 4.400000e+000 4.853300e+000

endloop

endfacet

facet normal -1.161583e-001 0.000000e+000 9.932307e-001

outer loop

vertex 8.029613e+000 4.400000e+000 4.853300e+000

vertex 8.029613e+000 3.000000e+000 4.853300e+000

vertex 8.116436e+000 3.000000e+000 4.860896e+000

endloop

endfacet

facet normal -5.807913e-002 0.000000e+000 9.983119e-001

outer loop

vertex 8.029613e+000 4.400000e+000 4.853300e+000

vertex 8.116436e+000 3.000000e+000 4.860896e+000

vertex 8.116436e+000 4.400000e+000 4.860896e+000

endloop

endfacet

facet normal 5.807913e-002 0.000000e+000 9.983119e-001

outer loop

vertex 8.116436e+000 4.400000e+000 4.860896e+000

vertex 8.116436e+000 3.000000e+000 4.860896e+000

vertex 8.203260e+000 3.000000e+000 4.853300e+000

endloop

endfacet

facet normal -1.000000e+000 0.000000e+000 -2.079452e-016

outer loop

vertex 0.000000e+000 3.000000e+000 6.244478e+000

vertex 0.000000e+000 0.000000e+000 0.000000e+000

vertex 0.000000e+000 3.000000e+000 9.744478e+000

endloop

endfacet

facet normal -1.000000e+000 0.000000e+000 -2.079452e-016

outer loop

vertex 0.000000e+000 3.000000e+000 9.744478e+000

vertex 0.000000e+000 0.000000e+000 0.000000e+000

vertex 0.000000e+000 0.000000e+000 1.251332e+001

endloop

endfacet

facet normal -1.000000e+000 0.000000e+000 -2.079452e-016

outer loop

vertex 0.000000e+000 3.000000e+000 9.744478e+000

vertex 0.000000e+000 0.000000e+000 1.251332e+001

vertex 0.000000e+000 3.000000e+000 1.251332e+001

endloop

endfacet

facet normal -1.000000e+000 0.000000e+000 -2.079452e-016

outer loop

vertex 0.000000e+000 3.000000e+000 6.244478e+000

vertex 0.000000e+000 3.000000e+000 2.744478e+000

vertex 0.000000e+000 0.000000e+000 0.000000e+000

endloop

endfacet

facet normal -1.000000e+000 0.000000e+000 -2.079452e-016

outer loop

vertex 0.000000e+000 0.000000e+000 0.000000e+000

vertex 0.000000e+000 3.000000e+000 2.744478e+000

vertex 0.000000e+000 3.000000e+000 3.140303e-001

endloop

endfacet

facet normal -1.000000e+000 0.000000e+000 -2.079452e-016

outer loop

vertex 0.000000e+000 0.000000e+000 0.000000e+000

vertex 0.000000e+000 3.000000e+000 3.140303e-001

vertex 0.000000e+000 3.000000e+000 0.000000e+000

endloop

endfacet

facet normal 7.278515e-017 0.000000e+000 1.000000e+000

outer loop

vertex 1.191674e+001 3.000000e+000 1.251332e+001

vertex 9.452423e+000 3.000000e+000 1.251332e+001

vertex 1.191674e+001 0.000000e+000 1.251332e+001

endloop

endfacet

facet normal 7.278515e-017 0.000000e+000 1.000000e+000

outer loop

vertex 1.191674e+001 0.000000e+000 1.251332e+001

vertex 9.452423e+000 3.000000e+000 1.251332e+001

vertex 7.594380e+000 3.000000e+000 1.251332e+001

endloop

endfacet

facet normal 7.278515e-017 0.000000e+000 1.000000e+000

outer loop

vertex 1.191674e+001 0.000000e+000 1.251332e+001

vertex 7.594380e+000 3.000000e+000 1.251332e+001

vertex 0.000000e+000 0.000000e+000 1.251332e+001

endloop

endfacet

facet normal 7.278515e-017 0.000000e+000 1.000000e+000

outer loop

vertex 0.000000e+000 0.000000e+000 1.251332e+001

vertex 7.594380e+000 3.000000e+000 1.251332e+001

vertex 5.930367e+000 3.000000e+000 1.251332e+001

endloop

endfacet

facet normal 7.278515e-017 0.000000e+000 1.000000e+000

outer loop

vertex 5.930367e+000 3.000000e+000 1.251332e+001

vertex 2.452423e+000 3.000000e+000 1.251332e+001

vertex 0.000000e+000 0.000000e+000 1.251332e+001

endloop

endfacet

facet normal 7.278515e-017 0.000000e+000 1.000000e+000

outer loop

vertex 0.000000e+000 0.000000e+000 1.251332e+001

vertex 2.452423e+000 3.000000e+000 1.251332e+001

vertex 5.943798e-001 3.000000e+000 1.251332e+001

endloop

endfacet

facet normal 7.278515e-017 0.000000e+000 1.000000e+000

outer loop

vertex 0.000000e+000 0.000000e+000 1.251332e+001

vertex 5.943798e-001 3.000000e+000 1.251332e+001

vertex 0.000000e+000 3.000000e+000 1.251332e+001

endloop

endfacet

facet normal 1.000000e+000 0.000000e+000 0.000000e+000

outer loop

vertex 1.191674e+001 0.000000e+000 0.000000e+000

vertex 1.191674e+001 3.000000e+000 0.000000e+000

vertex 1.191674e+001 3.000000e+000 3.140303e-001

endloop

endfacet

facet normal 1.000000e+000 0.000000e+000 0.000000e+000

outer loop

vertex 1.191674e+001 3.000000e+000 3.140303e-001

vertex 1.191674e+001 3.000000e+000 2.744478e+000

vertex 1.191674e+001 0.000000e+000 0.000000e+000

endloop

endfacet

facet normal 1.000000e+000 0.000000e+000 0.000000e+000

outer loop

vertex 1.191674e+001 0.000000e+000 0.000000e+000

vertex 1.191674e+001 3.000000e+000 2.744478e+000

vertex 1.191674e+001 3.000000e+000 6.244478e+000

endloop

endfacet

facet normal 1.000000e+000 0.000000e+000 0.000000e+000

outer loop

vertex 1.191674e+001 0.000000e+000 0.000000e+000

vertex 1.191674e+001 3.000000e+000 6.244478e+000

vertex 1.191674e+001 0.000000e+000 1.251332e+001

endloop

endfacet

facet normal 1.000000e+000 0.000000e+000 0.000000e+000

outer loop

vertex 1.191674e+001 0.000000e+000 1.251332e+001

vertex 1.191674e+001 3.000000e+000 6.244478e+000

vertex 1.191674e+001 3.000000e+000 9.744478e+000

endloop

endfacet

facet normal 1.000000e+000 0.000000e+000 0.000000e+000

outer loop

vertex 1.191674e+001 0.000000e+000 1.251332e+001

vertex 1.191674e+001 3.000000e+000 9.744478e+000

vertex 1.191674e+001 3.000000e+000 1.251332e+001

endloop

endfacet

facet normal 7.278515e-017 0.000000e+000 -1.000000e+000

outer loop

vertex 9.452423e+000 3.000000e+000 0.000000e+000

vertex 1.191674e+001 3.000000e+000 0.000000e+000

vertex 1.191674e+001 0.000000e+000 0.000000e+000

endloop

endfacet

facet normal 7.278515e-017 0.000000e+000 -1.000000e+000

outer loop

vertex 0.000000e+000 0.000000e+000 0.000000e+000

vertex 0.000000e+000 3.000000e+000 0.000000e+000

vertex 5.943798e-001 3.000000e+000 0.000000e+000

endloop

endfacet

facet normal 7.278515e-017 0.000000e+000 -1.000000e+000

outer loop

vertex 5.943798e-001 3.000000e+000 0.000000e+000

vertex 2.452423e+000 3.000000e+000 0.000000e+000

vertex 0.000000e+000 0.000000e+000 0.000000e+000

endloop

endfacet

facet normal 7.278515e-017 0.000000e+000 -1.000000e+000

outer loop

vertex 0.000000e+000 0.000000e+000 0.000000e+000

vertex 2.452423e+000 3.000000e+000 0.000000e+000

vertex 5.930367e+000 3.000000e+000 0.000000e+000

endloop

endfacet

facet normal 7.278515e-017 0.000000e+000 -1.000000e+000

outer loop

vertex 0.000000e+000 0.000000e+000 0.000000e+000

vertex 5.930367e+000 3.000000e+000 0.000000e+000

vertex 1.191674e+001 0.000000e+000 0.000000e+000

endloop

endfacet

facet normal 7.278515e-017 0.000000e+000 -1.000000e+000

outer loop

vertex 1.191674e+001 0.000000e+000 0.000000e+000

vertex 5.930367e+000 3.000000e+000 0.000000e+000

vertex 7.594380e+000 3.000000e+000 0.000000e+000

endloop

endfacet

facet normal 7.278515e-017 0.000000e+000 -1.000000e+000

outer loop

vertex 1.191674e+001 0.000000e+000 0.000000e+000

vertex 7.594380e+000 3.000000e+000 0.000000e+000

vertex 9.452423e+000 3.000000e+000 0.000000e+000

endloop

endfacet

facet normal 0.000000e+000 1.000000e+000 0.000000e+000

outer loop

vertex 1.087523e+001 3.000000e+000 1.620464e+000

vertex 1.078841e+001 3.000000e+000 1.628060e+000

vertex 1.191674e+001 3.000000e+000 2.744478e+000

endloop

endfacet

facet normal 0.000000e+000 1.000000e+000 0.000000e+000

outer loop

vertex 7.594380e+000 3.000000e+000 3.140303e-001

vertex 7.639311e+000 3.000000e+000 8.780602e-001

vertex 7.689301e+000 3.000000e+000 8.066660e-001

endloop

endfacet

facet normal 0.000000e+000 1.000000e+000 0.000000e+000

outer loop

vertex 7.689301e+000 3.000000e+000 8.066660e-001

vertex 7.750930e+000 3.000000e+000 7.450376e-001

vertex 7.594380e+000 3.000000e+000 3.140303e-001

endloop

endfacet

facet normal 0.000000e+000 1.000000e+000 0.000000e+000

outer loop

vertex 7.594380e+000 3.000000e+000 3.140303e-001

vertex 7.750930e+000 3.000000e+000 7.450376e-001

vertex 7.822324e+000 3.000000e+000 6.950474e-001

endloop

endfacet

facet normal 0.000000e+000 1.000000e+000 0.000000e+000

outer loop

vertex 7.594380e+000 3.000000e+000 3.140303e-001

vertex 7.822324e+000 3.000000e+000 6.950474e-001

vertex 7.901314e+000 3.000000e+000 6.582136e-001

endloop

endfacet

facet normal 0.000000e+000 1.000000e+000 0.000000e+000

outer loop

vertex 8.542170e+000 3.000000e+000 1.299070e+000

vertex 8.505336e+000 3.000000e+000 1.378060e+000

vertex 9.452423e+000 3.000000e+000 2.744478e+000

endloop

endfacet

facet normal 0.000000e+000 1.000000e+000 0.000000e+000

outer loop

vertex 9.452423e+000 3.000000e+000 2.744478e+000

vertex 8.505336e+000 3.000000e+000 1.378060e+000

vertex 8.455345e+000 3.000000e+000 1.449454e+000

endloop

endfacet

facet normal 0.000000e+000 1.000000e+000 0.000000e+000

outer loop

vertex 8.455345e+000 3.000000e+000 1.449454e+000

vertex 8.393718e+000 3.000000e+000 1.511082e+000

vertex 9.452423e+000 3.000000e+000 2.744478e+000

endloop

endfacet

facet normal 0.000000e+000 1.000000e+000 0.000000e+000

outer loop

vertex 9.452423e+000 3.000000e+000 2.744478e+000

vertex 8.393718e+000 3.000000e+000 1.511082e+000

vertex 8.322324e+000 3.000000e+000 1.561073e+000

endloop

endfacet

facet normal 0.000000e+000 1.000000e+000 0.000000e+000

outer loop

vertex 9.452423e+000 3.000000e+000 2.744478e+000

vertex 8.322324e+000 3.000000e+000 1.561073e+000

vertex 8.243334e+000 3.000000e+000 1.597906e+000

endloop

endfacet

facet normal 0.000000e+000 1.000000e+000 0.000000e+000

outer loop

vertex 7.901314e+000 3.000000e+000 6.582136e-001

vertex 7.985500e+000 3.000000e+000 6.356561e-001

vertex 7.594380e+000 3.000000e+000 3.140303e-001

endloop

endfacet

facet normal 0.000000e+000 1.000000e+000 0.000000e+000

outer loop

vertex 7.594380e+000 3.000000e+000 3.140303e-001

vertex 7.985500e+000 3.000000e+000 6.356561e-001

vertex 8.072324e+000 3.000000e+000 6.280602e-001

endloop

endfacet

facet normal 0.000000e+000 1.000000e+000 0.000000e+000

outer loop

vertex 7.594380e+000 3.000000e+000 3.140303e-001

vertex 8.072324e+000 3.000000e+000 6.280602e-001

vertex 9.452423e+000 3.000000e+000 3.140303e-001

endloop

endfacet

facet normal 0.000000e+000 1.000000e+000 0.000000e+000

outer loop

vertex 9.452423e+000 3.000000e+000 3.140303e-001

vertex 8.072324e+000 3.000000e+000 6.280602e-001

vertex 8.159147e+000 3.000000e+000 6.356561e-001

endloop

endfacet

facet normal 0.000000e+000 1.000000e+000 0.000000e+000

outer loop

vertex 8.159147e+000 3.000000e+000 6.356561e-001

vertex 8.243334e+000 3.000000e+000 6.582136e-001

vertex 9.452423e+000 3.000000e+000 3.140303e-001

endloop

endfacet

facet normal 0.000000e+000 1.000000e+000 0.000000e+000

outer loop

vertex 9.452423e+000 3.000000e+000 3.140303e-001

vertex 8.243334e+000 3.000000e+000 6.582136e-001

vertex 8.322324e+000 3.000000e+000 6.950474e-001

endloop

endfacet

facet normal 0.000000e+000 1.000000e+000 0.000000e+000

outer loop

vertex 9.452423e+000 3.000000e+000 3.140303e-001

vertex 8.322324e+000 3.000000e+000 6.950474e-001

vertex 8.393718e+000 3.000000e+000 7.450376e-001

endloop

endfacet

facet normal 0.000000e+000 1.000000e+000 0.000000e+000

outer loop

vertex 8.393718e+000 3.000000e+000 7.450376e-001

vertex 8.455345e+000 3.000000e+000 8.066660e-001

vertex 9.452423e+000 3.000000e+000 3.140303e-001

endloop

endfacet

facet normal 0.000000e+000 1.000000e+000 0.000000e+000

outer loop

vertex 9.452423e+000 3.000000e+000 3.140303e-001

vertex 8.455345e+000 3.000000e+000 8.066660e-001

vertex 8.505336e+000 3.000000e+000 8.780602e-001

endloop

endfacet

facet normal 0.000000e+000 1.000000e+000 0.000000e+000

outer loop

vertex 9.452423e+000 3.000000e+000 3.140303e-001

vertex 8.505336e+000 3.000000e+000 8.780602e-001

vertex 8.542170e+000 3.000000e+000 9.570499e-001

endloop

endfacet

facet normal 0.000000e+000 1.000000e+000 0.000000e+000

outer loop

vertex 8.243334e+000 3.000000e+000 1.597906e+000

vertex 8.159147e+000 3.000000e+000 1.620464e+000

vertex 7.594380e+000 3.000000e+000 2.744478e+000

endloop

endfacet

facet normal 0.000000e+000 1.000000e+000 0.000000e+000

outer loop

vertex 7.594380e+000 3.000000e+000 2.744478e+000

vertex 8.159147e+000 3.000000e+000 1.620464e+000

vertex 8.072324e+000 3.000000e+000 1.628060e+000

endloop

endfacet

facet normal 0.000000e+000 1.000000e+000 0.000000e+000

outer loop

vertex 8.542170e+000 3.000000e+000 9.570499e-001

vertex 8.564728e+000 3.000000e+000 1.041236e+000

vertex 9.452423e+000 3.000000e+000 3.140303e-001

endloop

endfacet

facet normal 0.000000e+000 1.000000e+000 0.000000e+000

outer loop

vertex 9.452423e+000 3.000000e+000 3.140303e-001

vertex 8.564728e+000 3.000000e+000 1.041236e+000

vertex 8.572323e+000 3.000000e+000 1.128060e+000

endloop

endfacet

facet normal 0.000000e+000 1.000000e+000 0.000000e+000

outer loop

vertex 9.452423e+000 3.000000e+000 3.140303e-001

vertex 8.572323e+000 3.000000e+000 1.128060e+000

vertex 9.452423e+000 3.000000e+000 2.744478e+000

endloop

endfacet

facet normal 0.000000e+000 1.000000e+000 0.000000e+000

outer loop

vertex 9.452423e+000 3.000000e+000 2.744478e+000

vertex 8.572323e+000 3.000000e+000 1.128060e+000

vertex 8.564728e+000 3.000000e+000 1.214884e+000

endloop

endfacet

facet normal 0.000000e+000 1.000000e+000 0.000000e+000

outer loop

vertex 9.452423e+000 3.000000e+000 2.744478e+000

vertex 8.564728e+000 3.000000e+000 1.214884e+000

vertex 8.542170e+000 3.000000e+000 1.299070e+000

endloop

endfacet

facet normal 0.000000e+000 1.000000e+000 0.000000e+000

outer loop

vertex 8.072324e+000 3.000000e+000 1.628060e+000

vertex 7.985500e+000 3.000000e+000 1.620464e+000

vertex 7.594380e+000 3.000000e+000 2.744478e+000

endloop

endfacet

facet normal 0.000000e+000 1.000000e+000 0.000000e+000

outer loop

vertex 7.594380e+000 3.000000e+000 2.744478e+000

vertex 7.985500e+000 3.000000e+000 1.620464e+000

vertex 7.901314e+000 3.000000e+000 1.597906e+000

endloop

endfacet

facet normal 0.000000e+000 1.000000e+000 0.000000e+000

outer loop

vertex 7.594380e+000 3.000000e+000 2.744478e+000

vertex 7.901314e+000 3.000000e+000 1.597906e+000

vertex 7.822324e+000 3.000000e+000 1.561073e+000

endloop

endfacet

facet normal 0.000000e+000 1.000000e+000 0.000000e+000

outer loop

vertex 7.822324e+000 3.000000e+000 1.561073e+000

vertex 7.750930e+000 3.000000e+000 1.511082e+000

vertex 7.594380e+000 3.000000e+000 2.744478e+000

endloop

endfacet

facet normal 0.000000e+000 1.000000e+000 0.000000e+000

outer loop

vertex 7.594380e+000 3.000000e+000 2.744478e+000

vertex 7.750930e+000 3.000000e+000 1.511082e+000

vertex 7.689301e+000 3.000000e+000 1.449454e+000

endloop

endfacet

facet normal 0.000000e+000 1.000000e+000 0.000000e+000

outer loop

vertex 7.594380e+000 3.000000e+000 2.744478e+000

vertex 7.689301e+000 3.000000e+000 1.449454e+000

vertex 7.639311e+000 3.000000e+000 1.378060e+000

endloop

endfacet

facet normal 0.000000e+000 1.000000e+000 0.000000e+000

outer loop

vertex 7.639311e+000 3.000000e+000 1.378060e+000

vertex 7.602478e+000 3.000000e+000 1.299070e+000

vertex 7.594380e+000 3.000000e+000 2.744478e+000

endloop

endfacet

facet normal 0.000000e+000 1.000000e+000 0.000000e+000

outer loop

vertex 7.594380e+000 3.000000e+000 2.744478e+000

vertex 7.602478e+000 3.000000e+000 1.299070e+000

vertex 7.594380e+000 3.000000e+000 1.268851e+000

endloop

endfacet

facet normal 0.000000e+000 1.000000e+000 0.000000e+000

outer loop

vertex 7.594380e+000 3.000000e+000 2.744478e+000

vertex 7.594380e+000 3.000000e+000 1.268851e+000

vertex 5.930367e+000 3.000000e+000 2.744478e+000

endloop

endfacet

facet normal 0.000000e+000 1.000000e+000 0.000000e+000

outer loop

vertex 5.930367e+000 3.000000e+000 2.744478e+000

vertex 7.594380e+000 3.000000e+000 1.268851e+000

vertex 7.579920e+000 3.000000e+000 1.214884e+000

endloop

endfacet

facet normal 0.000000e+000 1.000000e+000 0.000000e+000

outer loop

vertex 5.930367e+000 3.000000e+000 2.744478e+000

vertex 7.579920e+000 3.000000e+000 1.214884e+000

vertex 5.930367e+000 3.000000e+000 3.140303e-001

endloop

endfacet

facet normal 0.000000e+000 1.000000e+000 0.000000e+000

outer loop

vertex 5.930367e+000 3.000000e+000 3.140303e-001

vertex 7.579920e+000 3.000000e+000 1.214884e+000

vertex 7.572323e+000 3.000000e+000 1.128060e+000

endloop

endfacet

facet normal 0.000000e+000 1.000000e+000 0.000000e+000

outer loop

vertex 7.572323e+000 3.000000e+000 1.128060e+000

vertex 7.579920e+000 3.000000e+000 1.041236e+000

vertex 5.930367e+000 3.000000e+000 3.140303e-001

endloop

endfacet

facet normal 0.000000e+000 1.000000e+000 0.000000e+000

outer loop

vertex 5.930367e+000 3.000000e+000 3.140303e-001

vertex 7.579920e+000 3.000000e+000 1.041236e+000

vertex 7.594380e+000 3.000000e+000 9.872695e-001

endloop

endfacet

facet normal 0.000000e+000 1.000000e+000 0.000000e+000

outer loop

vertex 5.930367e+000 3.000000e+000 3.140303e-001

vertex 7.594380e+000 3.000000e+000 9.872695e-001

vertex 7.594380e+000 3.000000e+000 3.140303e-001

endloop

endfacet

facet normal 0.000000e+000 1.000000e+000 0.000000e+000

outer loop

vertex 7.594380e+000 3.000000e+000 3.140303e-001

vertex 7.594380e+000 3.000000e+000 9.872695e-001

vertex 7.602478e+000 3.000000e+000 9.570499e-001

endloop

endfacet

facet normal 0.000000e+000 1.000000e+000 0.000000e+000

outer loop

vertex 7.594380e+000 3.000000e+000 3.140303e-001

vertex 7.602478e+000 3.000000e+000 9.570499e-001

vertex 7.639311e+000 3.000000e+000 8.780602e-001

endloop

endfacet

facet normal 0.000000e+000 1.000000e+000 0.000000e+000

outer loop

vertex 6.393110e-001 3.000000e+000 8.780602e-001

vertex 6.893016e-001 3.000000e+000 8.066660e-001

vertex 5.943798e-001 3.000000e+000 3.140303e-001

endloop

endfacet

facet normal 0.000000e+000 1.000000e+000 0.000000e+000

outer loop

vertex 6.893016e-001 3.000000e+000 8.066660e-001

vertex 7.509301e-001 3.000000e+000 7.450376e-001

vertex 5.943798e-001 3.000000e+000 3.140303e-001

endloop

endfacet

facet normal 0.000000e+000 1.000000e+000 0.000000e+000

outer loop

vertex 5.943798e-001 3.000000e+000 3.140303e-001

vertex 7.509301e-001 3.000000e+000 7.450376e-001

vertex 8.223239e-001 3.000000e+000 6.950474e-001

endloop

endfacet

facet normal 0.000000e+000 1.000000e+000 0.000000e+000

outer loop

vertex 5.943798e-001 3.000000e+000 3.140303e-001

vertex 8.223239e-001 3.000000e+000 6.950474e-001

vertex 9.013135e-001 3.000000e+000 6.582136e-001

endloop

endfacet

facet normal 0.000000e+000 1.000000e+000 0.000000e+000

outer loop

vertex 9.013135e-001 3.000000e+000 6.582136e-001

vertex 9.854995e-001 3.000000e+000 6.356561e-001

vertex 5.943798e-001 3.000000e+000 3.140303e-001

endloop

endfacet

facet normal 0.000000e+000 1.000000e+000 0.000000e+000

outer loop

vertex 5.943798e-001 3.000000e+000 3.140303e-001

vertex 9.854995e-001 3.000000e+000 6.356561e-001

vertex 1.072323e+000 3.000000e+000 6.280602e-001

endloop

endfacet

facet normal 0.000000e+000 1.000000e+000 0.000000e+000

outer loop

vertex 5.943798e-001 3.000000e+000 3.140303e-001

vertex 1.072323e+000 3.000000e+000 6.280602e-001

vertex 2.452423e+000 3.000000e+000 3.140303e-001

endloop

endfacet

facet normal 0.000000e+000 1.000000e+000 0.000000e+000

outer loop

vertex 2.452423e+000 3.000000e+000 3.140303e-001

vertex 1.072323e+000 3.000000e+000 6.280602e-001

vertex 1.159148e+000 3.000000e+000 6.356561e-001

endloop

endfacet

facet normal 0.000000e+000 1.000000e+000 0.000000e+000

outer loop

vertex 1.159148e+000 3.000000e+000 6.356561e-001

vertex 1.243334e+000 3.000000e+000 6.582136e-001

vertex 2.452423e+000 3.000000e+000 3.140303e-001

endloop

endfacet

facet normal 0.000000e+000 1.000000e+000 0.000000e+000

outer loop

vertex 2.452423e+000 3.000000e+000 3.140303e-001

vertex 1.243334e+000 3.000000e+000 6.582136e-001

vertex 1.322323e+000 3.000000e+000 6.950474e-001

endloop

endfacet

facet normal 0.000000e+000 1.000000e+000 0.000000e+000

outer loop

vertex 2.452423e+000 3.000000e+000 3.140303e-001

vertex 1.322323e+000 3.000000e+000 6.950474e-001

vertex 1.393717e+000 3.000000e+000 7.450376e-001

endloop

endfacet

facet normal 0.000000e+000 1.000000e+000 0.000000e+000

outer loop

vertex 1.393717e+000 3.000000e+000 7.450376e-001

vertex 1.455346e+000 3.000000e+000 8.066660e-001

vertex 2.452423e+000 3.000000e+000 3.140303e-001

endloop

endfacet

facet normal 0.000000e+000 1.000000e+000 0.000000e+000

outer loop

vertex 2.452423e+000 3.000000e+000 3.140303e-001

vertex 1.455346e+000 3.000000e+000 8.066660e-001

vertex 1.505336e+000 3.000000e+000 8.780602e-001

endloop

endfacet

facet normal 0.000000e+000 1.000000e+000 0.000000e+000

outer loop

vertex 2.452423e+000 3.000000e+000 3.140303e-001

vertex 1.505336e+000 3.000000e+000 8.780602e-001

vertex 1.542170e+000 3.000000e+000 9.570499e-001

endloop

endfacet

facet normal 0.000000e+000 1.000000e+000 0.000000e+000

outer loop

vertex 1.542170e+000 3.000000e+000 9.570499e-001

vertex 1.564728e+000 3.000000e+000 1.041236e+000

vertex 2.452423e+000 3.000000e+000 3.140303e-001

endloop

endfacet

facet normal 0.000000e+000 1.000000e+000 0.000000e+000

outer loop

vertex 2.452423e+000 3.000000e+000 3.140303e-001

vertex 1.564728e+000 3.000000e+000 1.041236e+000

vertex 1.572323e+000 3.000000e+000 1.128060e+000

endloop

endfacet

facet normal 0.000000e+000 1.000000e+000 0.000000e+000

outer loop

vertex 2.452423e+000 3.000000e+000 3.140303e-001

vertex 1.572323e+000 3.000000e+000 1.128060e+000

vertex 2.452423e+000 3.000000e+000 2.744478e+000

endloop

endfacet

facet normal 0.000000e+000 1.000000e+000 0.000000e+000

outer loop

vertex 2.452423e+000 3.000000e+000 2.744478e+000

vertex 1.572323e+000 3.000000e+000 1.128060e+000

vertex 1.564728e+000 3.000000e+000 1.214884e+000

endloop

endfacet

facet normal 0.000000e+000 1.000000e+000 0.000000e+000

outer loop

vertex 2.452423e+000 3.000000e+000 2.744478e+000

vertex 1.564728e+000 3.000000e+000 1.214884e+000

vertex 1.542170e+000 3.000000e+000 1.299070e+000

endloop

endfacet

facet normal 0.000000e+000 1.000000e+000 0.000000e+000

outer loop

vertex 1.542170e+000 3.000000e+000 1.299070e+000

vertex 1.505336e+000 3.000000e+000 1.378060e+000

vertex 2.452423e+000 3.000000e+000 2.744478e+000

endloop

endfacet

facet normal 0.000000e+000 1.000000e+000 0.000000e+000

outer loop

vertex 2.452423e+000 3.000000e+000 2.744478e+000

vertex 1.505336e+000 3.000000e+000 1.378060e+000

vertex 1.455346e+000 3.000000e+000 1.449454e+000

endloop

endfacet

facet normal 0.000000e+000 1.000000e+000 0.000000e+000

outer loop

vertex 2.452423e+000 3.000000e+000 2.744478e+000

vertex 1.455346e+000 3.000000e+000 1.449454e+000

vertex 1.393717e+000 3.000000e+000 1.511082e+000

endloop

endfacet

facet normal 0.000000e+000 1.000000e+000 0.000000e+000

outer loop

vertex 6.893016e-001 3.000000e+000 1.449454e+000

vertex 5.943798e-001 3.000000e+000 2.744478e+000

vertex 7.509301e-001 3.000000e+000 1.511082e+000

endloop

endfacet

facet normal 0.000000e+000 1.000000e+000 0.000000e+000

outer loop

vertex 7.509301e-001 3.000000e+000 1.511082e+000

vertex 5.943798e-001 3.000000e+000 2.744478e+000

vertex 8.223239e-001 3.000000e+000 1.561073e+000

endloop

endfacet

facet normal 0.000000e+000 1.000000e+000 0.000000e+000

outer loop

vertex 6.893016e-001 3.000000e+000 1.449454e+000

vertex 6.393110e-001 3.000000e+000 1.378060e+000

vertex 5.943798e-001 3.000000e+000 2.744478e+000

endloop

endfacet

facet normal 0.000000e+000 1.000000e+000 0.000000e+000

outer loop

vertex 5.943798e-001 3.000000e+000 2.744478e+000

vertex 6.393110e-001 3.000000e+000 1.378060e+000

vertex 6.024773e-001 3.000000e+000 1.299070e+000

endloop

endfacet

facet normal 0.000000e+000 1.000000e+000 0.000000e+000

outer loop

vertex 5.943798e-001 3.000000e+000 2.744478e+000

vertex 6.024773e-001 3.000000e+000 1.299070e+000

vertex 5.943798e-001 3.000000e+000 1.268851e+000

endloop

endfacet

facet normal 0.000000e+000 1.000000e+000 0.000000e+000

outer loop

vertex 1.393717e+000 3.000000e+000 1.511082e+000

vertex 1.322323e+000 3.000000e+000 1.561073e+000

vertex 2.452423e+000 3.000000e+000 2.744478e+000

endloop

endfacet

facet normal 0.000000e+000 1.000000e+000 0.000000e+000

outer loop

vertex 2.452423e+000 3.000000e+000 2.744478e+000

vertex 1.322323e+000 3.000000e+000 1.561073e+000

vertex 1.243334e+000 3.000000e+000 1.597906e+000

endloop

endfacet

facet normal 0.000000e+000 1.000000e+000 0.000000e+000

outer loop

vertex 2.452423e+000 3.000000e+000 2.744478e+000

vertex 1.243334e+000 3.000000e+000 1.597906e+000

vertex 5.943798e-001 3.000000e+000 2.744478e+000

endloop

endfacet

facet normal 0.000000e+000 1.000000e+000 0.000000e+000

outer loop

vertex 5.943798e-001 3.000000e+000 2.744478e+000

vertex 1.243334e+000 3.000000e+000 1.597906e+000

vertex 1.159148e+000 3.000000e+000 1.620464e+000

endloop

endfacet

facet normal 0.000000e+000 1.000000e+000 0.000000e+000

outer loop

vertex 5.943798e-001 3.000000e+000 2.744478e+000

vertex 1.159148e+000 3.000000e+000 1.620464e+000

vertex 1.072323e+000 3.000000e+000 1.628060e+000

endloop

endfacet

facet normal 0.000000e+000 1.000000e+000 0.000000e+000

outer loop

vertex 1.072323e+000 3.000000e+000 1.628060e+000

vertex 9.854995e-001 3.000000e+000 1.620464e+000

vertex 5.943798e-001 3.000000e+000 2.744478e+000

endloop

endfacet

facet normal 0.000000e+000 1.000000e+000 0.000000e+000

outer loop

vertex 5.943798e-001 3.000000e+000 2.744478e+000

vertex 9.854995e-001 3.000000e+000 1.620464e+000

vertex 9.013135e-001 3.000000e+000 1.597906e+000

endloop

endfacet

facet normal 0.000000e+000 1.000000e+000 0.000000e+000

outer loop

vertex 5.943798e-001 3.000000e+000 2.744478e+000

vertex 9.013135e-001 3.000000e+000 1.597906e+000

vertex 8.223239e-001 3.000000e+000 1.561073e+000

endloop

endfacet

facet normal 0.000000e+000 1.000000e+000 0.000000e+000

outer loop

vertex 5.723238e-001 3.000000e+000 1.128060e+000

vertex 0.000000e+000 3.000000e+000 3.140303e-001

vertex 5.799197e-001 3.000000e+000 1.214884e+000

endloop

endfacet

facet normal 0.000000e+000 1.000000e+000 0.000000e+000

outer loop

vertex 5.799197e-001 3.000000e+000 1.214884e+000

vertex 0.000000e+000 3.000000e+000 3.140303e-001

vertex 0.000000e+000 3.000000e+000 2.744478e+000

endloop

endfacet

facet normal 0.000000e+000 1.000000e+000 0.000000e+000

outer loop

vertex 5.799197e-001 3.000000e+000 1.214884e+000

vertex 0.000000e+000 3.000000e+000 2.744478e+000

vertex 5.943798e-001 3.000000e+000 1.268851e+000

endloop

endfacet

facet normal 0.000000e+000 1.000000e+000 0.000000e+000

outer loop

vertex 5.723238e-001 3.000000e+000 1.128060e+000

vertex 5.799197e-001 3.000000e+000 1.041236e+000

vertex 0.000000e+000 3.000000e+000 3.140303e-001

endloop

endfacet

facet normal 0.000000e+000 1.000000e+000 0.000000e+000

outer loop

vertex 0.000000e+000 3.000000e+000 3.140303e-001

vertex 5.799197e-001 3.000000e+000 1.041236e+000

vertex 5.943798e-001 3.000000e+000 9.872695e-001

endloop

endfacet

facet normal 0.000000e+000 1.000000e+000 0.000000e+000

outer loop

vertex 0.000000e+000 3.000000e+000 3.140303e-001

vertex 5.943798e-001 3.000000e+000 9.872695e-001

vertex 5.943798e-001 3.000000e+000 3.140303e-001

endloop

endfacet

facet normal 0.000000e+000 1.000000e+000 0.000000e+000

outer loop

vertex 5.943798e-001 3.000000e+000 3.140303e-001

vertex 5.943798e-001 3.000000e+000 9.872695e-001

vertex 6.024773e-001 3.000000e+000 9.570499e-001

endloop

endfacet

facet normal 0.000000e+000 1.000000e+000 0.000000e+000

outer loop

vertex 5.943798e-001 3.000000e+000 3.140303e-001

vertex 6.024773e-001 3.000000e+000 9.570499e-001

vertex 6.393110e-001 3.000000e+000 8.780602e-001

endloop

endfacet

facet normal 0.000000e+000 1.000000e+000 0.000000e+000

outer loop

vertex 9.452423e+000 3.000000e+000 9.744478e+000

vertex 8.243334e+000 3.000000e+000 8.597907e+000

vertex 7.594380e+000 3.000000e+000 9.744478e+000

endloop

endfacet

facet normal 0.000000e+000 1.000000e+000 0.000000e+000

outer loop

vertex 7.594380e+000 3.000000e+000 9.744478e+000

vertex 8.243334e+000 3.000000e+000 8.597907e+000

vertex 8.159147e+000 3.000000e+000 8.620463e+000

endloop

endfacet

facet normal 0.000000e+000 1.000000e+000 0.000000e+000

outer loop

vertex 7.594380e+000 3.000000e+000 9.744478e+000

vertex 8.159147e+000 3.000000e+000 8.620463e+000

vertex 8.072324e+000 3.000000e+000 8.628060e+000

endloop

endfacet

facet normal 0.000000e+000 1.000000e+000 0.000000e+000

outer loop

vertex 7.639311e+000 3.000000e+000 7.878059e+000

vertex 7.689301e+000 3.000000e+000 7.806666e+000

vertex 7.594380e+000 3.000000e+000 6.244478e+000

endloop

endfacet

facet normal 0.000000e+000 1.000000e+000 0.000000e+000

outer loop

vertex 7.594380e+000 3.000000e+000 6.244478e+000

vertex 7.689301e+000 3.000000e+000 7.806666e+000

vertex 7.750930e+000 3.000000e+000 7.745038e+000

endloop

endfacet

facet normal 0.000000e+000 1.000000e+000 0.000000e+000

outer loop

vertex 8.564728e+000 3.000000e+000 8.214885e+000

vertex 8.542170e+000 3.000000e+000 8.299070e+000

vertex 9.452423e+000 3.000000e+000 9.744478e+000

endloop

endfacet

facet normal 0.000000e+000 1.000000e+000 0.000000e+000

outer loop

vertex 9.452423e+000 3.000000e+000 9.744478e+000

vertex 8.542170e+000 3.000000e+000 8.299070e+000

vertex 8.505336e+000 3.000000e+000 8.378060e+000

endloop

endfacet

facet normal 0.000000e+000 1.000000e+000 0.000000e+000

outer loop

vertex 9.452423e+000 3.000000e+000 9.744478e+000

vertex 8.505336e+000 3.000000e+000 8.378060e+000

vertex 8.455345e+000 3.000000e+000 8.449454e+000

endloop

endfacet

facet normal 0.000000e+000 1.000000e+000 0.000000e+000

outer loop

vertex 7.750930e+000 3.000000e+000 7.745038e+000

vertex 7.822324e+000 3.000000e+000 7.695047e+000

vertex 7.594380e+000 3.000000e+000 6.244478e+000

endloop

endfacet

facet normal 0.000000e+000 1.000000e+000 0.000000e+000

outer loop

vertex 7.594380e+000 3.000000e+000 6.244478e+000

vertex 7.822324e+000 3.000000e+000 7.695047e+000

vertex 7.901314e+000 3.000000e+000 7.658214e+000

endloop

endfacet

facet normal 0.000000e+000 1.000000e+000 0.000000e+000

outer loop

vertex 7.594380e+000 3.000000e+000 6.244478e+000

vertex 7.901314e+000 3.000000e+000 7.658214e+000

vertex 7.985500e+000 3.000000e+000 7.635656e+000

endloop

endfacet

facet normal 0.000000e+000 1.000000e+000 0.000000e+000

outer loop

vertex 8.455345e+000 3.000000e+000 8.449454e+000

vertex 8.393718e+000 3.000000e+000 8.511083e+000

vertex 9.452423e+000 3.000000e+000 9.744478e+000

endloop

endfacet

facet normal 0.000000e+000 1.000000e+000 0.000000e+000

outer loop

vertex 9.452423e+000 3.000000e+000 9.744478e+000

vertex 8.393718e+000 3.000000e+000 8.511083e+000

vertex 8.322324e+000 3.000000e+000 8.561073e+000

endloop

endfacet

facet normal 0.000000e+000 1.000000e+000 0.000000e+000

outer loop

vertex 9.452423e+000 3.000000e+000 9.744478e+000

vertex 8.322324e+000 3.000000e+000 8.561073e+000

vertex 8.243334e+000 3.000000e+000 8.597907e+000

endloop

endfacet

facet normal 0.000000e+000 1.000000e+000 0.000000e+000

outer loop

vertex 8.322324e+000 3.000000e+000 7.695047e+000

vertex 8.393718e+000 3.000000e+000 7.745038e+000

vertex 9.452423e+000 3.000000e+000 6.244478e+000

endloop

endfacet

facet normal 0.000000e+000 1.000000e+000 0.000000e+000

outer loop

vertex 9.452423e+000 3.000000e+000 6.244478e+000

vertex 8.393718e+000 3.000000e+000 7.745038e+000

vertex 8.455345e+000 3.000000e+000 7.806666e+000

endloop

endfacet

facet normal 0.000000e+000 1.000000e+000 0.000000e+000

outer loop

vertex 9.452423e+000 3.000000e+000 6.244478e+000

vertex 8.455345e+000 3.000000e+000 7.806666e+000

vertex 8.505336e+000 3.000000e+000 7.878059e+000

endloop

endfacet

facet normal 0.000000e+000 1.000000e+000 0.000000e+000

outer loop

vertex 8.072324e+000 3.000000e+000 8.628060e+000

vertex 7.985500e+000 3.000000e+000 8.620463e+000

vertex 7.594380e+000 3.000000e+000 9.744478e+000

endloop

endfacet

facet normal 0.000000e+000 1.000000e+000 0.000000e+000

outer loop

vertex 7.594380e+000 3.000000e+000 9.744478e+000

vertex 7.985500e+000 3.000000e+000 8.620463e+000

vertex 7.901314e+000 3.000000e+000 8.597907e+000

endloop

endfacet

facet normal 0.000000e+000 1.000000e+000 0.000000e+000

outer loop

vertex 7.594380e+000 3.000000e+000 9.744478e+000

vertex 7.901314e+000 3.000000e+000 8.597907e+000

vertex 7.822324e+000 3.000000e+000 8.561073e+000

endloop

endfacet

facet normal 0.000000e+000 1.000000e+000 0.000000e+000

outer loop

vertex 7.985500e+000 3.000000e+000 7.635656e+000

vertex 8.072324e+000 3.000000e+000 7.628060e+000

vertex 7.594380e+000 3.000000e+000 6.244478e+000

endloop

endfacet

facet normal 0.000000e+000 1.000000e+000 0.000000e+000

outer loop

vertex 7.594380e+000 3.000000e+000 6.244478e+000

vertex 8.072324e+000 3.000000e+000 7.628060e+000

vertex 8.159147e+000 3.000000e+000 7.635656e+000

endloop

endfacet

facet normal 0.000000e+000 1.000000e+000 0.000000e+000

outer loop

vertex 7.594380e+000 3.000000e+000 6.244478e+000

vertex 8.159147e+000 3.000000e+000 7.635656e+000

vertex 9.452423e+000 3.000000e+000 6.244478e+000

endloop

endfacet

facet normal 0.000000e+000 1.000000e+000 0.000000e+000

outer loop

vertex 9.452423e+000 3.000000e+000 6.244478e+000

vertex 8.159147e+000 3.000000e+000 7.635656e+000

vertex 8.243334e+000 3.000000e+000 7.658214e+000

endloop

endfacet

facet normal 0.000000e+000 1.000000e+000 0.000000e+000

outer loop

vertex 9.452423e+000 3.000000e+000 6.244478e+000

vertex 8.243334e+000 3.000000e+000 7.658214e+000

vertex 8.322324e+000 3.000000e+000 7.695047e+000

endloop

endfacet

facet normal 0.000000e+000 1.000000e+000 0.000000e+000

outer loop

vertex 7.822324e+000 3.000000e+000 8.561073e+000

vertex 7.750930e+000 3.000000e+000 8.511083e+000

vertex 7.594380e+000 3.000000e+000 9.744478e+000

endloop

endfacet

facet normal 0.000000e+000 1.000000e+000 0.000000e+000

outer loop

vertex 7.594380e+000 3.000000e+000 9.744478e+000

vertex 7.750930e+000 3.000000e+000 8.511083e+000

vertex 7.689301e+000 3.000000e+000 8.449454e+000

endloop

endfacet

facet normal 0.000000e+000 1.000000e+000 0.000000e+000

outer loop

vertex 7.594380e+000 3.000000e+000 9.744478e+000

vertex 7.689301e+000 3.000000e+000 8.449454e+000

vertex 7.639311e+000 3.000000e+000 8.378060e+000

endloop

endfacet

facet normal 0.000000e+000 1.000000e+000 0.000000e+000

outer loop

vertex 8.505336e+000 3.000000e+000 7.878059e+000

vertex 8.542170e+000 3.000000e+000 7.957050e+000

vertex 9.452423e+000 3.000000e+000 6.244478e+000

endloop

endfacet

facet normal 0.000000e+000 1.000000e+000 0.000000e+000

outer loop

vertex 9.452423e+000 3.000000e+000 6.244478e+000

vertex 8.542170e+000 3.000000e+000 7.957050e+000

vertex 8.564728e+000 3.000000e+000 8.041236e+000

endloop

endfacet

facet normal 0.000000e+000 1.000000e+000 0.000000e+000

outer loop

vertex 9.452423e+000 3.000000e+000 6.244478e+000

vertex 8.564728e+000 3.000000e+000 8.041236e+000

vertex 9.452423e+000 3.000000e+000 9.744478e+000

endloop

endfacet

facet normal 0.000000e+000 1.000000e+000 0.000000e+000

outer loop

vertex 9.452423e+000 3.000000e+000 9.744478e+000

vertex 8.564728e+000 3.000000e+000 8.041236e+000

vertex 8.572323e+000 3.000000e+000 8.128060e+000

endloop

endfacet

facet normal 0.000000e+000 1.000000e+000 0.000000e+000

outer loop

vertex 9.452423e+000 3.000000e+000 9.744478e+000

vertex 8.572323e+000 3.000000e+000 8.128060e+000

vertex 8.564728e+000 3.000000e+000 8.214885e+000

endloop

endfacet

facet normal 0.000000e+000 1.000000e+000 0.000000e+000

outer loop

vertex 7.639311e+000 3.000000e+000 8.378060e+000

vertex 7.602478e+000 3.000000e+000 8.299070e+000

vertex 7.594380e+000 3.000000e+000 9.744478e+000

endloop

endfacet

facet normal 0.000000e+000 1.000000e+000 0.000000e+000

outer loop

vertex 7.594380e+000 3.000000e+000 9.744478e+000

vertex 7.602478e+000 3.000000e+000 8.299070e+000

vertex 7.594380e+000 3.000000e+000 8.268851e+000

endloop

endfacet

facet normal 0.000000e+000 1.000000e+000 0.000000e+000

outer loop

vertex 7.594380e+000 3.000000e+000 9.744478e+000

vertex 7.594380e+000 3.000000e+000 8.268851e+000

vertex 5.930367e+000 3.000000e+000 9.744478e+000

endloop

endfacet

facet normal 0.000000e+000 1.000000e+000 0.000000e+000

outer loop

vertex 7.594380e+000 3.000000e+000 8.268851e+000

vertex 7.579920e+000 3.000000e+000 8.214885e+000

vertex 5.930367e+000 3.000000e+000 9.744478e+000

endloop

endfacet

facet normal 0.000000e+000 1.000000e+000 0.000000e+000

outer loop

vertex 5.930367e+000 3.000000e+000 9.744478e+000

vertex 7.579920e+000 3.000000e+000 8.214885e+000

vertex 7.572323e+000 3.000000e+000 8.128060e+000

endloop

endfacet

facet normal 0.000000e+000 1.000000e+000 0.000000e+000

outer loop

vertex 5.930367e+000 3.000000e+000 9.744478e+000

vertex 7.572323e+000 3.000000e+000 8.128060e+000

vertex 5.930367e+000 3.000000e+000 6.244478e+000

endloop

endfacet

facet normal 0.000000e+000 1.000000e+000 0.000000e+000

outer loop

vertex 7.572323e+000 3.000000e+000 8.128060e+000

vertex 7.579920e+000 3.000000e+000 8.041236e+000

vertex 5.930367e+000 3.000000e+000 6.244478e+000

endloop

endfacet

facet normal 0.000000e+000 1.000000e+000 0.000000e+000

outer loop

vertex 5.930367e+000 3.000000e+000 6.244478e+000

vertex 7.579920e+000 3.000000e+000 8.041236e+000

vertex 7.594380e+000 3.000000e+000 7.987269e+000

endloop

endfacet

facet normal 0.000000e+000 1.000000e+000 0.000000e+000

outer loop

vertex 5.930367e+000 3.000000e+000 6.244478e+000

vertex 7.594380e+000 3.000000e+000 7.987269e+000

vertex 7.594380e+000 3.000000e+000 6.244478e+000

endloop

endfacet

facet normal 0.000000e+000 1.000000e+000 0.000000e+000

outer loop

vertex 7.594380e+000 3.000000e+000 6.244478e+000

vertex 7.594380e+000 3.000000e+000 7.987269e+000

vertex 7.602478e+000 3.000000e+000 7.957050e+000

endloop

endfacet

facet normal 0.000000e+000 1.000000e+000 0.000000e+000

outer loop

vertex 7.594380e+000 3.000000e+000 6.244478e+000

vertex 7.602478e+000 3.000000e+000 7.957050e+000

vertex 7.639311e+000 3.000000e+000 7.878059e+000

endloop

endfacet

facet normal 0.000000e+000 1.000000e+000 0.000000e+000

outer loop

vertex 6.024773e-001 3.000000e+000 7.957050e+000

vertex 6.393110e-001 3.000000e+000 7.878059e+000

vertex 5.943798e-001 3.000000e+000 6.244478e+000

endloop

endfacet

facet normal 0.000000e+000 1.000000e+000 0.000000e+000

outer loop

vertex 5.943798e-001 3.000000e+000 6.244478e+000

vertex 6.393110e-001 3.000000e+000 7.878059e+000

vertex 6.893016e-001 3.000000e+000 7.806666e+000

endloop

endfacet

facet normal 0.000000e+000 1.000000e+000 0.000000e+000

outer loop

vertex 5.943798e-001 3.000000e+000 6.244478e+000

vertex 6.893016e-001 3.000000e+000 7.806666e+000

vertex 7.509301e-001 3.000000e+000 7.745038e+000

endloop

endfacet

facet normal 0.000000e+000 1.000000e+000 0.000000e+000

outer loop

vertex 7.509301e-001 3.000000e+000 7.745038e+000

vertex 8.223239e-001 3.000000e+000 7.695047e+000

vertex 5.943798e-001 3.000000e+000 6.244478e+000

endloop

endfacet

facet normal 0.000000e+000 1.000000e+000 0.000000e+000

outer loop

vertex 5.943798e-001 3.000000e+000 6.244478e+000

vertex 8.223239e-001 3.000000e+000 7.695047e+000

vertex 9.013135e-001 3.000000e+000 7.658214e+000

endloop

endfacet

facet normal 0.000000e+000 1.000000e+000 0.000000e+000

outer loop

vertex 5.943798e-001 3.000000e+000 6.244478e+000

vertex 9.013135e-001 3.000000e+000 7.658214e+000

vertex 9.854995e-001 3.000000e+000 7.635656e+000

endloop

endfacet

facet normal 0.000000e+000 1.000000e+000 0.000000e+000

outer loop

vertex 9.854995e-001 3.000000e+000 7.635656e+000

vertex 1.072323e+000 3.000000e+000 7.628060e+000

vertex 5.943798e-001 3.000000e+000 6.244478e+000

endloop

endfacet

facet normal 0.000000e+000 1.000000e+000 0.000000e+000

outer loop

vertex 5.943798e-001 3.000000e+000 6.244478e+000

vertex 1.072323e+000 3.000000e+000 7.628060e+000

vertex 1.159148e+000 3.000000e+000 7.635656e+000

endloop

endfacet

facet normal 0.000000e+000 1.000000e+000 0.000000e+000

outer loop

vertex 5.943798e-001 3.000000e+000 6.244478e+000

vertex 1.159148e+000 3.000000e+000 7.635656e+000

vertex 2.452423e+000 3.000000e+000 6.244478e+000

endloop

endfacet

facet normal 0.000000e+000 1.000000e+000 0.000000e+000

outer loop

vertex 2.452423e+000 3.000000e+000 6.244478e+000

vertex 1.159148e+000 3.000000e+000 7.635656e+000

vertex 1.243334e+000 3.000000e+000 7.658214e+000

endloop

endfacet

facet normal 0.000000e+000 1.000000e+000 0.000000e+000

outer loop

vertex 2.452423e+000 3.000000e+000 6.244478e+000

vertex 1.243334e+000 3.000000e+000 7.658214e+000

vertex 1.322323e+000 3.000000e+000 7.695047e+000

endloop

endfacet

facet normal 0.000000e+000 1.000000e+000 0.000000e+000

outer loop

vertex 1.322323e+000 3.000000e+000 7.695047e+000

vertex 1.393717e+000 3.000000e+000 7.745038e+000

vertex 2.452423e+000 3.000000e+000 6.244478e+000

endloop

endfacet

facet normal 0.000000e+000 1.000000e+000 0.000000e+000

outer loop

vertex 2.452423e+000 3.000000e+000 6.244478e+000

vertex 1.393717e+000 3.000000e+000 7.745038e+000

vertex 1.455346e+000 3.000000e+000 7.806666e+000

endloop

endfacet

facet normal 0.000000e+000 1.000000e+000 0.000000e+000

outer loop

vertex 2.452423e+000 3.000000e+000 6.244478e+000

vertex 1.455346e+000 3.000000e+000 7.806666e+000

vertex 1.505336e+000 3.000000e+000 7.878059e+000

endloop

endfacet

facet normal 0.000000e+000 1.000000e+000 0.000000e+000

outer loop

vertex 1.564728e+000 3.000000e+000 8.214885e+000

vertex 1.542170e+000 3.000000e+000 8.299070e+000

vertex 2.452423e+000 3.000000e+000 9.744478e+000

endloop

endfacet

facet normal 0.000000e+000 1.000000e+000 0.000000e+000

outer loop

vertex 2.452423e+000 3.000000e+000 9.744478e+000

vertex 1.542170e+000 3.000000e+000 8.299070e+000

vertex 1.505336e+000 3.000000e+000 8.378060e+000

endloop

endfacet

facet normal 0.000000e+000 1.000000e+000 0.000000e+000

outer loop

vertex 1.505336e+000 3.000000e+000 7.878059e+000

vertex 1.542170e+000 3.000000e+000 7.957050e+000

vertex 2.452423e+000 3.000000e+000 6.244478e+000

endloop

endfacet

facet normal 0.000000e+000 1.000000e+000 0.000000e+000

outer loop

vertex 2.452423e+000 3.000000e+000 6.244478e+000

vertex 1.542170e+000 3.000000e+000 7.957050e+000

vertex 1.564728e+000 3.000000e+000 8.041236e+000

endloop

endfacet

facet normal 0.000000e+000 1.000000e+000 0.000000e+000

outer loop

vertex 2.452423e+000 3.000000e+000 6.244478e+000

vertex 1.564728e+000 3.000000e+000 8.041236e+000

vertex 2.452423e+000 3.000000e+000 9.744478e+000

endloop

endfacet

facet normal 0.000000e+000 1.000000e+000 0.000000e+000

outer loop

vertex 2.452423e+000 3.000000e+000 9.744478e+000

vertex 1.564728e+000 3.000000e+000 8.041236e+000

vertex 1.572323e+000 3.000000e+000 8.128060e+000

endloop

endfacet

facet normal 0.000000e+000 1.000000e+000 0.000000e+000

outer loop

vertex 2.452423e+000 3.000000e+000 9.744478e+000

vertex 1.572323e+000 3.000000e+000 8.128060e+000

vertex 1.564728e+000 3.000000e+000 8.214885e+000

endloop

endfacet

facet normal 0.000000e+000 1.000000e+000 0.000000e+000

outer loop

vertex 1.505336e+000 3.000000e+000 8.378060e+000

vertex 1.455346e+000 3.000000e+000 8.449454e+000

vertex 2.452423e+000 3.000000e+000 9.744478e+000

endloop

endfacet

facet normal 0.000000e+000 1.000000e+000 0.000000e+000

outer loop

vertex 2.452423e+000 3.000000e+000 9.744478e+000

vertex 1.455346e+000 3.000000e+000 8.449454e+000

vertex 1.393717e+000 3.000000e+000 8.511083e+000

endloop

endfacet

facet normal 0.000000e+000 1.000000e+000 0.000000e+000

outer loop

vertex 2.452423e+000 3.000000e+000 9.744478e+000

vertex 1.393717e+000 3.000000e+000 8.511083e+000

vertex 1.322323e+000 3.000000e+000 8.561073e+000

endloop

endfacet

facet normal 0.000000e+000 1.000000e+000 0.000000e+000

outer loop

vertex 1.243334e+000 3.000000e+000 8.597907e+000

vertex 1.159148e+000 3.000000e+000 8.620463e+000

vertex 5.943798e-001 3.000000e+000 9.744478e+000

endloop

endfacet

facet normal 0.000000e+000 1.000000e+000 0.000000e+000

outer loop

vertex 5.943798e-001 3.000000e+000 9.744478e+000

vertex 1.159148e+000 3.000000e+000 8.620463e+000

vertex 1.072323e+000 3.000000e+000 8.628060e+000

endloop

endfacet

facet normal 0.000000e+000 1.000000e+000 0.000000e+000

outer loop

vertex 8.223239e-001 3.000000e+000 8.561073e+000

vertex 7.509301e-001 3.000000e+000 8.511083e+000

vertex 5.943798e-001 3.000000e+000 9.744478e+000

endloop

endfacet

facet normal 0.000000e+000 1.000000e+000 0.000000e+000

outer loop

vertex 5.943798e-001 3.000000e+000 9.744478e+000

vertex 7.509301e-001 3.000000e+000 8.511083e+000

vertex 6.893016e-001 3.000000e+000 8.449454e+000

endloop

endfacet

facet normal 0.000000e+000 1.000000e+000 0.000000e+000

outer loop

vertex 5.943798e-001 3.000000e+000 9.744478e+000

vertex 6.893016e-001 3.000000e+000 8.449454e+000

vertex 6.393110e-001 3.000000e+000 8.378060e+000

endloop

endfacet

facet normal 0.000000e+000 1.000000e+000 0.000000e+000

outer loop

vertex 1.072323e+000 3.000000e+000 8.628060e+000

vertex 9.854995e-001 3.000000e+000 8.620463e+000

vertex 5.943798e-001 3.000000e+000 9.744478e+000

endloop

endfacet

facet normal 0.000000e+000 1.000000e+000 0.000000e+000

outer loop

vertex 5.943798e-001 3.000000e+000 9.744478e+000

vertex 9.854995e-001 3.000000e+000 8.620463e+000

vertex 9.013135e-001 3.000000e+000 8.597907e+000

endloop

endfacet

facet normal 0.000000e+000 1.000000e+000 0.000000e+000

outer loop

vertex 5.943798e-001 3.000000e+000 9.744478e+000

vertex 9.013135e-001 3.000000e+000 8.597907e+000

vertex 8.223239e-001 3.000000e+000 8.561073e+000

endloop

endfacet

facet normal 0.000000e+000 1.000000e+000 0.000000e+000

outer loop

vertex 6.393110e-001 3.000000e+000 8.378060e+000

vertex 6.024773e-001 3.000000e+000 8.299070e+000

vertex 5.943798e-001 3.000000e+000 9.744478e+000

endloop

endfacet

facet normal 0.000000e+000 1.000000e+000 0.000000e+000

outer loop

vertex 5.943798e-001 3.000000e+000 9.744478e+000

vertex 6.024773e-001 3.000000e+000 8.299070e+000

vertex 5.943798e-001 3.000000e+000 8.268851e+000

endloop

endfacet

facet normal 0.000000e+000 1.000000e+000 0.000000e+000

outer loop

vertex 5.943798e-001 3.000000e+000 9.744478e+000

vertex 5.943798e-001 3.000000e+000 8.268851e+000

vertex 0.000000e+000 3.000000e+000 9.744478e+000

endloop

endfacet

facet normal 0.000000e+000 1.000000e+000 0.000000e+000

outer loop

vertex 5.943798e-001 3.000000e+000 8.268851e+000

vertex 5.799197e-001 3.000000e+000 8.214885e+000

vertex 0.000000e+000 3.000000e+000 9.744478e+000

endloop

endfacet

facet normal 0.000000e+000 1.000000e+000 0.000000e+000

outer loop

vertex 0.000000e+000 3.000000e+000 9.744478e+000

vertex 5.799197e-001 3.000000e+000 8.214885e+000

vertex 5.723238e-001 3.000000e+000 8.128060e+000

endloop

endfacet

facet normal 0.000000e+000 1.000000e+000 0.000000e+000

outer loop

vertex 0.000000e+000 3.000000e+000 9.744478e+000

vertex 5.723238e-001 3.000000e+000 8.128060e+000

vertex 0.000000e+000 3.000000e+000 6.244478e+000

endloop

endfacet

facet normal 0.000000e+000 1.000000e+000 0.000000e+000

outer loop

vertex 0.000000e+000 3.000000e+000 6.244478e+000

vertex 5.723238e-001 3.000000e+000 8.128060e+000

vertex 5.799197e-001 3.000000e+000 8.041236e+000

endloop

endfacet

facet normal 0.000000e+000 1.000000e+000 0.000000e+000

outer loop

vertex 0.000000e+000 3.000000e+000 6.244478e+000

vertex 5.799197e-001 3.000000e+000 8.041236e+000

vertex 5.943798e-001 3.000000e+000 7.987269e+000

endloop

endfacet

facet normal 0.000000e+000 1.000000e+000 0.000000e+000

outer loop

vertex 1.031856e+001 3.000000e+000 1.118989e+001

vertex 1.035540e+001 3.000000e+000 1.111090e+001

vertex 9.452423e+000 3.000000e+000 9.744478e+000

endloop

endfacet

facet normal 0.000000e+000 1.000000e+000 0.000000e+000

outer loop

vertex 1.095942e+001 3.000000e+000 1.089105e+001

vertex 1.103841e+001 3.000000e+000 1.092788e+001

vertex 1.191674e+001 3.000000e+000 9.744478e+000

endloop

endfacet

facet normal 0.000000e+000 1.000000e+000 0.000000e+000

outer loop

vertex 1.061740e+001 3.000000e+000 1.183074e+001

vertex 1.053841e+001 3.000000e+000 1.179391e+001

vertex 9.452423e+000 3.000000e+000 1.251332e+001

endloop

endfacet

facet normal 0.000000e+000 1.000000e+000 0.000000e+000

outer loop

vertex 9.452423e+000 3.000000e+000 1.251332e+001

vertex 1.053841e+001 3.000000e+000 1.179391e+001

vertex 1.046702e+001 3.000000e+000 1.174392e+001

endloop

endfacet

facet normal 0.000000e+000 1.000000e+000 0.000000e+000

outer loop

vertex 1.046702e+001 3.000000e+000 1.174392e+001

vertex 1.040539e+001 3.000000e+000 1.168229e+001

vertex 9.452423e+000 3.000000e+000 1.251332e+001

endloop

endfacet

facet normal 0.000000e+000 1.000000e+000 0.000000e+000

outer loop

vertex 9.452423e+000 3.000000e+000 1.251332e+001

vertex 1.040539e+001 3.000000e+000 1.168229e+001

vertex 1.035540e+001 3.000000e+000 1.161090e+001

endloop

endfacet

facet normal 0.000000e+000 1.000000e+000 0.000000e+000

outer loop

vertex 9.452423e+000 3.000000e+000 1.251332e+001

vertex 1.035540e+001 3.000000e+000 1.161090e+001

vertex 1.031856e+001 3.000000e+000 1.153191e+001

endloop

endfacet

facet normal 0.000000e+000 1.000000e+000 0.000000e+000

outer loop

vertex 1.035540e+001 3.000000e+000 1.111090e+001

vertex 1.040539e+001 3.000000e+000 1.103950e+001

vertex 9.452423e+000 3.000000e+000 9.744478e+000

endloop

endfacet

facet normal 0.000000e+000 1.000000e+000 0.000000e+000

outer loop

vertex 9.452423e+000 3.000000e+000 9.744478e+000

vertex 1.040539e+001 3.000000e+000 1.103950e+001

vertex 1.046702e+001 3.000000e+000 1.097787e+001

endloop

endfacet

facet normal 0.000000e+000 1.000000e+000 0.000000e+000

outer loop

vertex 9.452423e+000 3.000000e+000 9.744478e+000

vertex 1.046702e+001 3.000000e+000 1.097787e+001

vertex 1.053841e+001 3.000000e+000 1.092788e+001

endloop

endfacet

facet normal 0.000000e+000 1.000000e+000 0.000000e+000

outer loop

vertex 1.095942e+001 3.000000e+000 1.089105e+001

vertex 1.191674e+001 3.000000e+000 9.744478e+000

vertex 1.087523e+001 3.000000e+000 1.086849e+001

endloop

endfacet

facet normal 0.000000e+000 1.000000e+000 0.000000e+000

outer loop

vertex 1.103841e+001 3.000000e+000 1.092788e+001

vertex 1.110980e+001 3.000000e+000 1.097787e+001

vertex 1.191674e+001 3.000000e+000 9.744478e+000

endloop

endfacet

facet normal 0.000000e+000 1.000000e+000 0.000000e+000

outer loop

vertex 1.191674e+001 3.000000e+000 9.744478e+000

vertex 1.110980e+001 3.000000e+000 1.097787e+001

vertex 1.117143e+001 3.000000e+000 1.103950e+001

endloop

endfacet

facet normal 0.000000e+000 1.000000e+000 0.000000e+000

outer loop

vertex 1.191674e+001 3.000000e+000 9.744478e+000

vertex 1.117143e+001 3.000000e+000 1.103950e+001

vertex 1.122142e+001 3.000000e+000 1.111090e+001

endloop

endfacet

facet normal 0.000000e+000 1.000000e+000 0.000000e+000

outer loop

vertex 1.031856e+001 3.000000e+000 1.153191e+001

vertex 1.029601e+001 3.000000e+000 1.144772e+001

vertex 9.452423e+000 3.000000e+000 1.251332e+001

endloop

endfacet

facet normal 0.000000e+000 1.000000e+000 0.000000e+000

outer loop

vertex 9.452423e+000 3.000000e+000 1.251332e+001

vertex 1.029601e+001 3.000000e+000 1.144772e+001

vertex 1.028841e+001 3.000000e+000 1.136090e+001

endloop

endfacet

facet normal 0.000000e+000 1.000000e+000 0.000000e+000

outer loop

vertex 9.452423e+000 3.000000e+000 1.251332e+001

vertex 1.028841e+001 3.000000e+000 1.136090e+001

vertex 9.452423e+000 3.000000e+000 9.744478e+000

endloop

endfacet

facet normal 0.000000e+000 1.000000e+000 0.000000e+000

outer loop

vertex 9.452423e+000 3.000000e+000 9.744478e+000

vertex 1.028841e+001 3.000000e+000 1.136090e+001

vertex 1.029601e+001 3.000000e+000 1.127407e+001

endloop

endfacet

facet normal 0.000000e+000 1.000000e+000 0.000000e+000

outer loop

vertex 9.452423e+000 3.000000e+000 9.744478e+000

vertex 1.029601e+001 3.000000e+000 1.127407e+001

vertex 1.031856e+001 3.000000e+000 1.118989e+001

endloop

endfacet

facet normal 0.000000e+000 1.000000e+000 0.000000e+000

outer loop

vertex 1.128081e+001 3.000000e+000 1.144772e+001

vertex 1.125826e+001 3.000000e+000 1.153191e+001

vertex 1.191674e+001 3.000000e+000 1.251332e+001

endloop

endfacet

facet normal 0.000000e+000 1.000000e+000 0.000000e+000

outer loop

vertex 1.191674e+001 3.000000e+000 1.251332e+001

vertex 1.125826e+001 3.000000e+000 1.153191e+001

vertex 1.122142e+001 3.000000e+000 1.161090e+001

endloop

endfacet

facet normal 0.000000e+000 1.000000e+000 0.000000e+000

outer loop

vertex 1.191674e+001 3.000000e+000 1.251332e+001

vertex 1.122142e+001 3.000000e+000 1.161090e+001

vertex 1.117143e+001 3.000000e+000 1.168229e+001

endloop

endfacet

facet normal 0.000000e+000 1.000000e+000 0.000000e+000

outer loop

vertex 1.053841e+001 3.000000e+000 1.092788e+001

vertex 1.061740e+001 3.000000e+000 1.089105e+001

vertex 9.452423e+000 3.000000e+000 9.744478e+000

endloop

endfacet

facet normal 0.000000e+000 1.000000e+000 0.000000e+000

outer loop

vertex 9.452423e+000 3.000000e+000 9.744478e+000

vertex 1.061740e+001 3.000000e+000 1.089105e+001

vertex 1.070159e+001 3.000000e+000 1.086849e+001

endloop

endfacet

facet normal 0.000000e+000 1.000000e+000 0.000000e+000

outer loop

vertex 9.452423e+000 3.000000e+000 9.744478e+000

vertex 1.070159e+001 3.000000e+000 1.086849e+001

vertex 1.191674e+001 3.000000e+000 9.744478e+000

endloop

endfacet

facet normal 0.000000e+000 1.000000e+000 0.000000e+000

outer loop

vertex 1.191674e+001 3.000000e+000 9.744478e+000

vertex 1.070159e+001 3.000000e+000 1.086849e+001

vertex 1.078841e+001 3.000000e+000 1.086090e+001

endloop

endfacet

facet normal 0.000000e+000 1.000000e+000 0.000000e+000

outer loop

vertex 1.191674e+001 3.000000e+000 9.744478e+000

vertex 1.078841e+001 3.000000e+000 1.086090e+001

vertex 1.087523e+001 3.000000e+000 1.086849e+001

endloop

endfacet

facet normal 0.000000e+000 1.000000e+000 0.000000e+000

outer loop

vertex 1.122142e+001 3.000000e+000 1.111090e+001

vertex 1.125826e+001 3.000000e+000 1.118989e+001

vertex 1.191674e+001 3.000000e+000 9.744478e+000

endloop

endfacet

facet normal 0.000000e+000 1.000000e+000 0.000000e+000

outer loop

vertex 1.191674e+001 3.000000e+000 9.744478e+000

vertex 1.125826e+001 3.000000e+000 1.118989e+001

vertex 1.128081e+001 3.000000e+000 1.127407e+001

endloop

endfacet

facet normal 0.000000e+000 1.000000e+000 0.000000e+000

outer loop

vertex 1.191674e+001 3.000000e+000 9.744478e+000

vertex 1.128081e+001 3.000000e+000 1.127407e+001

vertex 1.191674e+001 3.000000e+000 1.251332e+001

endloop

endfacet

facet normal 0.000000e+000 1.000000e+000 0.000000e+000

outer loop

vertex 1.191674e+001 3.000000e+000 1.251332e+001

vertex 1.128081e+001 3.000000e+000 1.127407e+001

vertex 1.128841e+001 3.000000e+000 1.136090e+001

endloop

endfacet

facet normal 0.000000e+000 1.000000e+000 0.000000e+000

outer loop

vertex 1.191674e+001 3.000000e+000 1.251332e+001

vertex 1.128841e+001 3.000000e+000 1.136090e+001

vertex 1.128081e+001 3.000000e+000 1.144772e+001

endloop

endfacet

facet normal 0.000000e+000 1.000000e+000 0.000000e+000

outer loop

vertex 1.095942e+001 3.000000e+000 1.183074e+001

vertex 1.087523e+001 3.000000e+000 1.185330e+001

vertex 1.191674e+001 3.000000e+000 1.251332e+001

endloop

endfacet

facet normal 0.000000e+000 1.000000e+000 0.000000e+000

outer loop

vertex 1.191674e+001 3.000000e+000 1.251332e+001

vertex 1.087523e+001 3.000000e+000 1.185330e+001

vertex 1.078841e+001 3.000000e+000 1.186090e+001

endloop

endfacet

facet normal 0.000000e+000 1.000000e+000 0.000000e+000

outer loop

vertex 1.191674e+001 3.000000e+000 1.251332e+001

vertex 1.078841e+001 3.000000e+000 1.186090e+001

vertex 9.452423e+000 3.000000e+000 1.251332e+001

endloop

endfacet

facet normal 0.000000e+000 1.000000e+000 0.000000e+000

outer loop

vertex 9.452423e+000 3.000000e+000 1.251332e+001

vertex 1.078841e+001 3.000000e+000 1.186090e+001

vertex 1.070159e+001 3.000000e+000 1.185330e+001

endloop

endfacet

facet normal 0.000000e+000 1.000000e+000 0.000000e+000

outer loop

vertex 9.452423e+000 3.000000e+000 1.251332e+001

vertex 1.070159e+001 3.000000e+000 1.185330e+001

vertex 1.061740e+001 3.000000e+000 1.183074e+001

endloop

endfacet

facet normal 0.000000e+000 1.000000e+000 0.000000e+000

outer loop

vertex 1.117143e+001 3.000000e+000 1.168229e+001

vertex 1.110980e+001 3.000000e+000 1.174392e+001

vertex 1.191674e+001 3.000000e+000 1.251332e+001

endloop

endfacet

facet normal 0.000000e+000 1.000000e+000 0.000000e+000

outer loop

vertex 1.191674e+001 3.000000e+000 1.251332e+001

vertex 1.110980e+001 3.000000e+000 1.174392e+001

vertex 1.103841e+001 3.000000e+000 1.179391e+001

endloop

endfacet

facet normal 0.000000e+000 1.000000e+000 0.000000e+000

outer loop

vertex 1.191674e+001 3.000000e+000 1.251332e+001

vertex 1.103841e+001 3.000000e+000 1.179391e+001

vertex 1.095942e+001 3.000000e+000 1.183074e+001

endloop

endfacet

facet normal 0.000000e+000 1.000000e+000 0.000000e+000

outer loop

vertex 1.128081e+001 3.000000e+000 8.214885e+000

vertex 1.191674e+001 3.000000e+000 9.744478e+000

vertex 1.128841e+001 3.000000e+000 8.128060e+000

endloop

endfacet

facet normal 0.000000e+000 1.000000e+000 0.000000e+000

outer loop

vertex 1.128841e+001 3.000000e+000 8.128060e+000

vertex 1.191674e+001 3.000000e+000 9.744478e+000

vertex 1.191674e+001 3.000000e+000 6.244478e+000

endloop

endfacet

facet normal 0.000000e+000 1.000000e+000 0.000000e+000

outer loop

vertex 1.128081e+001 3.000000e+000 8.214885e+000

vertex 1.125826e+001 3.000000e+000 8.299070e+000

vertex 1.191674e+001 3.000000e+000 9.744478e+000

endloop

endfacet

facet normal 0.000000e+000 1.000000e+000 0.000000e+000

outer loop

vertex 1.191674e+001 3.000000e+000 9.744478e+000

vertex 1.125826e+001 3.000000e+000 8.299070e+000

vertex 1.122142e+001 3.000000e+000 8.378060e+000

endloop

endfacet

facet normal 0.000000e+000 1.000000e+000 0.000000e+000

outer loop

vertex 1.191674e+001 3.000000e+000 9.744478e+000

vertex 1.122142e+001 3.000000e+000 8.378060e+000

vertex 1.117143e+001 3.000000e+000 8.449454e+000

endloop

endfacet

facet normal 0.000000e+000 1.000000e+000 0.000000e+000

outer loop

vertex 1.070159e+001 3.000000e+000 7.635656e+000

vertex 1.078841e+001 3.000000e+000 7.628060e+000

vertex 1.191674e+001 3.000000e+000 6.244478e+000

endloop

endfacet

facet normal 0.000000e+000 1.000000e+000 0.000000e+000

outer loop

vertex 1.046702e+001 3.000000e+000 8.511083e+000

vertex 9.452423e+000 3.000000e+000 9.744478e+000

vertex 1.053841e+001 3.000000e+000 8.561073e+000

endloop

endfacet

facet normal 0.000000e+000 1.000000e+000 0.000000e+000

outer loop

vertex 1.053841e+001 3.000000e+000 8.561073e+000

vertex 9.452423e+000 3.000000e+000 9.744478e+000

vertex 1.061740e+001 3.000000e+000 8.597907e+000

endloop

endfacet

facet normal 0.000000e+000 1.000000e+000 0.000000e+000

outer loop

vertex 1.046702e+001 3.000000e+000 8.511083e+000

vertex 1.040539e+001 3.000000e+000 8.449454e+000

vertex 9.452423e+000 3.000000e+000 9.744478e+000

endloop

endfacet

facet normal 0.000000e+000 1.000000e+000 0.000000e+000

outer loop

vertex 9.452423e+000 3.000000e+000 9.744478e+000

vertex 1.040539e+001 3.000000e+000 8.449454e+000

vertex 1.035540e+001 3.000000e+000 8.378060e+000

endloop

endfacet

facet normal 0.000000e+000 1.000000e+000 0.000000e+000

outer loop

vertex 9.452423e+000 3.000000e+000 9.744478e+000

vertex 1.035540e+001 3.000000e+000 8.378060e+000

vertex 1.031856e+001 3.000000e+000 8.299070e+000

endloop

endfacet

facet normal 0.000000e+000 1.000000e+000 0.000000e+000

outer loop

vertex 1.031856e+001 3.000000e+000 7.957050e+000

vertex 1.035540e+001 3.000000e+000 7.878059e+000

vertex 9.452423e+000 3.000000e+000 6.244478e+000

endloop

endfacet

facet normal 0.000000e+000 1.000000e+000 0.000000e+000

outer loop

vertex 9.452423e+000 3.000000e+000 6.244478e+000

vertex 1.035540e+001 3.000000e+000 7.878059e+000

vertex 1.040539e+001 3.000000e+000 7.806666e+000

endloop

endfacet

facet normal 0.000000e+000 1.000000e+000 0.000000e+000

outer loop

vertex 9.452423e+000 3.000000e+000 6.244478e+000

vertex 1.040539e+001 3.000000e+000 7.806666e+000

vertex 1.046702e+001 3.000000e+000 7.745038e+000

endloop

endfacet

facet normal 0.000000e+000 1.000000e+000 0.000000e+000

outer loop

vertex 1.046702e+001 3.000000e+000 7.745038e+000

vertex 1.053841e+001 3.000000e+000 7.695047e+000

vertex 9.452423e+000 3.000000e+000 6.244478e+000

endloop

endfacet

facet normal 0.000000e+000 1.000000e+000 0.000000e+000

outer loop

vertex 9.452423e+000 3.000000e+000 6.244478e+000

vertex 1.053841e+001 3.000000e+000 7.695047e+000

vertex 1.061740e+001 3.000000e+000 7.658214e+000

endloop

endfacet

facet normal 0.000000e+000 1.000000e+000 0.000000e+000

outer loop

vertex 9.452423e+000 3.000000e+000 6.244478e+000

vertex 1.061740e+001 3.000000e+000 7.658214e+000

vertex 1.070159e+001 3.000000e+000 7.635656e+000

endloop

endfacet

facet normal 0.000000e+000 1.000000e+000 0.000000e+000

outer loop

vertex 1.078841e+001 3.000000e+000 7.628060e+000

vertex 1.087523e+001 3.000000e+000 7.635656e+000

vertex 1.191674e+001 3.000000e+000 6.244478e+000

endloop

endfacet

facet normal 0.000000e+000 1.000000e+000 0.000000e+000

outer loop

vertex 1.191674e+001 3.000000e+000 6.244478e+000

vertex 1.087523e+001 3.000000e+000 7.635656e+000

vertex 1.095942e+001 3.000000e+000 7.658214e+000

endloop

endfacet

facet normal 0.000000e+000 1.000000e+000 0.000000e+000

outer loop

vertex 1.191674e+001 3.000000e+000 6.244478e+000

vertex 1.095942e+001 3.000000e+000 7.658214e+000

vertex 1.103841e+001 3.000000e+000 7.695047e+000

endloop

endfacet

facet normal 0.000000e+000 1.000000e+000 0.000000e+000

outer loop

vertex 1.103841e+001 3.000000e+000 7.695047e+000

vertex 1.110980e+001 3.000000e+000 7.745038e+000

vertex 1.191674e+001 3.000000e+000 6.244478e+000

endloop

endfacet

facet normal 0.000000e+000 1.000000e+000 0.000000e+000

outer loop

vertex 1.191674e+001 3.000000e+000 6.244478e+000

vertex 1.110980e+001 3.000000e+000 7.745038e+000

vertex 1.117143e+001 3.000000e+000 7.806666e+000

endloop

endfacet

facet normal 0.000000e+000 1.000000e+000 0.000000e+000

outer loop

vertex 1.191674e+001 3.000000e+000 6.244478e+000

vertex 1.117143e+001 3.000000e+000 7.806666e+000

vertex 1.122142e+001 3.000000e+000 7.878059e+000

endloop

endfacet

facet normal 0.000000e+000 1.000000e+000 0.000000e+000

outer loop

vertex 1.122142e+001 3.000000e+000 7.878059e+000

vertex 1.125826e+001 3.000000e+000 7.957050e+000

vertex 1.191674e+001 3.000000e+000 6.244478e+000

endloop

endfacet

facet normal 0.000000e+000 1.000000e+000 0.000000e+000

outer loop

vertex 1.191674e+001 3.000000e+000 6.244478e+000

vertex 1.125826e+001 3.000000e+000 7.957050e+000

vertex 1.128081e+001 3.000000e+000 8.041236e+000

endloop

endfacet

facet normal 0.000000e+000 1.000000e+000 0.000000e+000

outer loop

vertex 1.191674e+001 3.000000e+000 6.244478e+000

vertex 1.128081e+001 3.000000e+000 8.041236e+000

vertex 1.128841e+001 3.000000e+000 8.128060e+000

endloop

endfacet

facet normal 0.000000e+000 1.000000e+000 0.000000e+000

outer loop

vertex 1.117143e+001 3.000000e+000 8.449454e+000

vertex 1.110980e+001 3.000000e+000 8.511083e+000

vertex 1.191674e+001 3.000000e+000 9.744478e+000

endloop

endfacet

facet normal 0.000000e+000 1.000000e+000 0.000000e+000

outer loop

vertex 1.191674e+001 3.000000e+000 9.744478e+000

vertex 1.110980e+001 3.000000e+000 8.511083e+000

vertex 1.103841e+001 3.000000e+000 8.561073e+000

endloop

endfacet

facet normal 0.000000e+000 1.000000e+000 0.000000e+000

outer loop

vertex 1.191674e+001 3.000000e+000 9.744478e+000

vertex 1.103841e+001 3.000000e+000 8.561073e+000

vertex 1.095942e+001 3.000000e+000 8.597907e+000

endloop

endfacet

facet normal 0.000000e+000 1.000000e+000 0.000000e+000

outer loop

vertex 1.095942e+001 3.000000e+000 8.597907e+000

vertex 1.087523e+001 3.000000e+000 8.620463e+000

vertex 1.191674e+001 3.000000e+000 9.744478e+000

endloop

endfacet

facet normal 0.000000e+000 1.000000e+000 0.000000e+000

outer loop

vertex 1.191674e+001 3.000000e+000 9.744478e+000

vertex 1.087523e+001 3.000000e+000 8.620463e+000

vertex 1.078841e+001 3.000000e+000 8.628060e+000

endloop

endfacet

facet normal 0.000000e+000 1.000000e+000 0.000000e+000

outer loop

vertex 1.191674e+001 3.000000e+000 9.744478e+000

vertex 1.078841e+001 3.000000e+000 8.628060e+000

vertex 9.452423e+000 3.000000e+000 9.744478e+000

endloop

endfacet

facet normal 0.000000e+000 1.000000e+000 0.000000e+000

outer loop

vertex 9.452423e+000 3.000000e+000 9.744478e+000

vertex 1.078841e+001 3.000000e+000 8.628060e+000

vertex 1.070159e+001 3.000000e+000 8.620463e+000

endloop

endfacet

facet normal 0.000000e+000 1.000000e+000 0.000000e+000

outer loop

vertex 9.452423e+000 3.000000e+000 9.744478e+000

vertex 1.070159e+001 3.000000e+000 8.620463e+000

vertex 1.061740e+001 3.000000e+000 8.597907e+000

endloop

endfacet

facet normal 0.000000e+000 1.000000e+000 0.000000e+000

outer loop

vertex 1.031856e+001 3.000000e+000 8.299070e+000

vertex 1.029601e+001 3.000000e+000 8.214885e+000

vertex 9.452423e+000 3.000000e+000 9.744478e+000

endloop

endfacet

facet normal 0.000000e+000 1.000000e+000 0.000000e+000

outer loop

vertex 9.452423e+000 3.000000e+000 9.744478e+000

vertex 1.029601e+001 3.000000e+000 8.214885e+000

vertex 1.028841e+001 3.000000e+000 8.128060e+000

endloop

endfacet

facet normal 0.000000e+000 1.000000e+000 0.000000e+000

outer loop

vertex 9.452423e+000 3.000000e+000 9.744478e+000

vertex 1.028841e+001 3.000000e+000 8.128060e+000

vertex 9.452423e+000 3.000000e+000 6.244478e+000

endloop

endfacet

facet normal 0.000000e+000 1.000000e+000 0.000000e+000

outer loop

vertex 9.452423e+000 3.000000e+000 6.244478e+000

vertex 1.028841e+001 3.000000e+000 8.128060e+000

vertex 1.029601e+001 3.000000e+000 8.041236e+000

endloop

endfacet

facet normal 0.000000e+000 1.000000e+000 0.000000e+000

outer loop

vertex 9.452423e+000 3.000000e+000 6.244478e+000

vertex 1.029601e+001 3.000000e+000 8.041236e+000

vertex 1.031856e+001 3.000000e+000 7.957050e+000

endloop

endfacet

facet normal 0.000000e+000 1.000000e+000 0.000000e+000

outer loop

vertex 1.070159e+001 3.000000e+000 4.853300e+000

vertex 1.061740e+001 3.000000e+000 4.830742e+000

vertex 9.452423e+000 3.000000e+000 6.244478e+000

endloop

endfacet

facet normal 0.000000e+000 1.000000e+000 0.000000e+000

outer loop

vertex 9.452423e+000 3.000000e+000 6.244478e+000

vertex 1.061740e+001 3.000000e+000 4.830742e+000

vertex 1.053841e+001 3.000000e+000 4.793909e+000

endloop

endfacet

facet normal 0.000000e+000 1.000000e+000 0.000000e+000

outer loop

vertex 1.070159e+001 3.000000e+000 7.635656e+000

vertex 1.191674e+001 3.000000e+000 6.244478e+000

vertex 9.452423e+000 3.000000e+000 6.244478e+000

endloop

endfacet

facet normal 0.000000e+000 1.000000e+000 0.000000e+000

outer loop

vertex 9.452423e+000 3.000000e+000 6.244478e+000

vertex 1.191674e+001 3.000000e+000 6.244478e+000

vertex 1.078841e+001 3.000000e+000 4.860896e+000

endloop

endfacet

facet normal 0.000000e+000 1.000000e+000 0.000000e+000

outer loop

vertex 9.452423e+000 3.000000e+000 6.244478e+000

vertex 1.078841e+001 3.000000e+000 4.860896e+000

vertex 1.070159e+001 3.000000e+000 4.853300e+000

endloop

endfacet

facet normal 0.000000e+000 1.000000e+000 0.000000e+000

outer loop

vertex 1.053841e+001 3.000000e+000 4.793909e+000

vertex 1.046702e+001 3.000000e+000 4.743918e+000

vertex 9.452423e+000 3.000000e+000 6.244478e+000

endloop

endfacet

facet normal 0.000000e+000 1.000000e+000 0.000000e+000

outer loop

vertex 9.452423e+000 3.000000e+000 6.244478e+000

vertex 1.046702e+001 3.000000e+000 4.743918e+000

vertex 1.040539e+001 3.000000e+000 4.682290e+000

endloop

endfacet

facet normal 0.000000e+000 1.000000e+000 0.000000e+000

outer loop

vertex 9.452423e+000 3.000000e+000 6.244478e+000

vertex 1.040539e+001 3.000000e+000 4.682290e+000

vertex 1.035540e+001 3.000000e+000 4.610896e+000

endloop

endfacet

facet normal 0.000000e+000 1.000000e+000 0.000000e+000

outer loop

vertex 1.035540e+001 3.000000e+000 4.110896e+000

vertex 9.452423e+000 3.000000e+000 2.744478e+000

vertex 1.031856e+001 3.000000e+000 4.189886e+000

endloop

endfacet

facet normal 0.000000e+000 1.000000e+000 0.000000e+000

outer loop

vertex 1.031856e+001 3.000000e+000 4.189886e+000

vertex 9.452423e+000 3.000000e+000 2.744478e+000

vertex 1.029601e+001 3.000000e+000 4.274072e+000

endloop

endfacet

facet normal 0.000000e+000 1.000000e+000 0.000000e+000

outer loop

vertex 1.035540e+001 3.000000e+000 4.110896e+000

vertex 1.040539e+001 3.000000e+000 4.039502e+000

vertex 9.452423e+000 3.000000e+000 2.744478e+000

endloop

endfacet

facet normal 0.000000e+000 1.000000e+000 0.000000e+000

outer loop

vertex 9.452423e+000 3.000000e+000 2.744478e+000

vertex 1.040539e+001 3.000000e+000 4.039502e+000

vertex 1.046702e+001 3.000000e+000 3.977873e+000

endloop

endfacet

facet normal 0.000000e+000 1.000000e+000 0.000000e+000

outer loop

vertex 9.452423e+000 3.000000e+000 2.744478e+000

vertex 1.046702e+001 3.000000e+000 3.977873e+000

vertex 1.053841e+001 3.000000e+000 3.927883e+000

endloop

endfacet

facet normal 0.000000e+000 1.000000e+000 0.000000e+000

outer loop

vertex 1.087523e+001 3.000000e+000 3.868492e+000

vertex 1.095942e+001 3.000000e+000 3.891049e+000

vertex 1.191674e+001 3.000000e+000 2.744478e+000

endloop

endfacet

facet normal 0.000000e+000 1.000000e+000 0.000000e+000

outer loop

vertex 1.191674e+001 3.000000e+000 2.744478e+000

vertex 1.095942e+001 3.000000e+000 3.891049e+000

vertex 1.103841e+001 3.000000e+000 3.927883e+000

endloop

endfacet

facet normal 0.000000e+000 1.000000e+000 0.000000e+000

outer loop

vertex 1.191674e+001 3.000000e+000 2.744478e+000

vertex 1.103841e+001 3.000000e+000 3.927883e+000

vertex 1.110980e+001 3.000000e+000 3.977873e+000

endloop

endfacet

facet normal 0.000000e+000 1.000000e+000 0.000000e+000

outer loop

vertex 1.035540e+001 3.000000e+000 4.610896e+000

vertex 1.031856e+001 3.000000e+000 4.531906e+000

vertex 9.452423e+000 3.000000e+000 6.244478e+000

endloop

endfacet

facet normal 0.000000e+000 1.000000e+000 0.000000e+000

outer loop

vertex 9.452423e+000 3.000000e+000 6.244478e+000

vertex 1.031856e+001 3.000000e+000 4.531906e+000

vertex 1.029601e+001 3.000000e+000 4.447720e+000

endloop

endfacet

facet normal 0.000000e+000 1.000000e+000 0.000000e+000

outer loop

vertex 9.452423e+000 3.000000e+000 6.244478e+000

vertex 1.029601e+001 3.000000e+000 4.447720e+000

vertex 9.452423e+000 3.000000e+000 2.744478e+000

endloop

endfacet

facet normal 0.000000e+000 1.000000e+000 0.000000e+000

outer loop

vertex 9.452423e+000 3.000000e+000 2.744478e+000

vertex 1.029601e+001 3.000000e+000 4.447720e+000

vertex 1.028841e+001 3.000000e+000 4.360896e+000

endloop

endfacet

facet normal 0.000000e+000 1.000000e+000 0.000000e+000

outer loop

vertex 9.452423e+000 3.000000e+000 2.744478e+000

vertex 1.028841e+001 3.000000e+000 4.360896e+000

vertex 1.029601e+001 3.000000e+000 4.274072e+000

endloop

endfacet

facet normal 0.000000e+000 1.000000e+000 0.000000e+000

outer loop

vertex 1.053841e+001 3.000000e+000 3.927883e+000

vertex 1.061740e+001 3.000000e+000 3.891049e+000

vertex 9.452423e+000 3.000000e+000 2.744478e+000

endloop

endfacet

facet normal 0.000000e+000 1.000000e+000 0.000000e+000

outer loop

vertex 9.452423e+000 3.000000e+000 2.744478e+000

vertex 1.061740e+001 3.000000e+000 3.891049e+000

vertex 1.070159e+001 3.000000e+000 3.868492e+000

endloop

endfacet

facet normal 0.000000e+000 1.000000e+000 0.000000e+000

outer loop

vertex 9.452423e+000 3.000000e+000 2.744478e+000

vertex 1.070159e+001 3.000000e+000 3.868492e+000

vertex 1.191674e+001 3.000000e+000 2.744478e+000

endloop

endfacet

facet normal 0.000000e+000 1.000000e+000 0.000000e+000

outer loop

vertex 1.191674e+001 3.000000e+000 2.744478e+000

vertex 1.070159e+001 3.000000e+000 3.868492e+000

vertex 1.078841e+001 3.000000e+000 3.860896e+000

endloop

endfacet

facet normal 0.000000e+000 1.000000e+000 0.000000e+000

outer loop

vertex 1.191674e+001 3.000000e+000 2.744478e+000

vertex 1.078841e+001 3.000000e+000 3.860896e+000

vertex 1.087523e+001 3.000000e+000 3.868492e+000

endloop

endfacet

facet normal 0.000000e+000 1.000000e+000 0.000000e+000

outer loop

vertex 1.110980e+001 3.000000e+000 3.977873e+000

vertex 1.117143e+001 3.000000e+000 4.039502e+000

vertex 1.191674e+001 3.000000e+000 2.744478e+000

endloop

endfacet

facet normal 0.000000e+000 1.000000e+000 0.000000e+000

outer loop

vertex 1.191674e+001 3.000000e+000 2.744478e+000

vertex 1.117143e+001 3.000000e+000 4.039502e+000

vertex 1.122142e+001 3.000000e+000 4.110896e+000

endloop

endfacet

facet normal 0.000000e+000 1.000000e+000 0.000000e+000

outer loop

vertex 1.191674e+001 3.000000e+000 2.744478e+000

vertex 1.122142e+001 3.000000e+000 4.110896e+000

vertex 1.125826e+001 3.000000e+000 4.189886e+000

endloop

endfacet

facet normal 0.000000e+000 1.000000e+000 0.000000e+000

outer loop

vertex 1.191674e+001 3.000000e+000 6.244478e+000

vertex 1.125826e+001 3.000000e+000 4.531906e+000

vertex 1.122142e+001 3.000000e+000 4.610896e+000

endloop

endfacet

facet normal 0.000000e+000 1.000000e+000 0.000000e+000

outer loop

vertex 1.122142e+001 3.000000e+000 4.610896e+000

vertex 1.117143e+001 3.000000e+000 4.682290e+000

vertex 1.191674e+001 3.000000e+000 6.244478e+000

endloop

endfacet

facet normal 0.000000e+000 1.000000e+000 0.000000e+000

outer loop

vertex 1.191674e+001 3.000000e+000 6.244478e+000

vertex 1.117143e+001 3.000000e+000 4.682290e+000

vertex 1.110980e+001 3.000000e+000 4.743918e+000

endloop

endfacet

facet normal 0.000000e+000 1.000000e+000 0.000000e+000

outer loop

vertex 1.191674e+001 3.000000e+000 6.244478e+000

vertex 1.110980e+001 3.000000e+000 4.743918e+000

vertex 1.103841e+001 3.000000e+000 4.793909e+000

endloop

endfacet

facet normal 0.000000e+000 1.000000e+000 0.000000e+000

outer loop

vertex 1.125826e+001 3.000000e+000 4.189886e+000

vertex 1.128081e+001 3.000000e+000 4.274072e+000

vertex 1.191674e+001 3.000000e+000 2.744478e+000

endloop

endfacet

facet normal 0.000000e+000 1.000000e+000 0.000000e+000

outer loop

vertex 1.191674e+001 3.000000e+000 2.744478e+000

vertex 1.128081e+001 3.000000e+000 4.274072e+000

vertex 1.128841e+001 3.000000e+000 4.360896e+000

endloop

endfacet

facet normal 0.000000e+000 1.000000e+000 0.000000e+000

outer loop

vertex 1.191674e+001 3.000000e+000 2.744478e+000

vertex 1.128841e+001 3.000000e+000 4.360896e+000

vertex 1.191674e+001 3.000000e+000 6.244478e+000

endloop

endfacet

facet normal 0.000000e+000 1.000000e+000 0.000000e+000

outer loop

vertex 1.191674e+001 3.000000e+000 6.244478e+000

vertex 1.128841e+001 3.000000e+000 4.360896e+000

vertex 1.128081e+001 3.000000e+000 4.447720e+000

endloop

endfacet

facet normal 0.000000e+000 1.000000e+000 0.000000e+000

outer loop

vertex 1.191674e+001 3.000000e+000 6.244478e+000

vertex 1.128081e+001 3.000000e+000 4.447720e+000

vertex 1.125826e+001 3.000000e+000 4.531906e+000

endloop

endfacet

facet normal 0.000000e+000 1.000000e+000 0.000000e+000

outer loop

vertex 1.103841e+001 3.000000e+000 4.793909e+000

vertex 1.095942e+001 3.000000e+000 4.830742e+000

vertex 1.191674e+001 3.000000e+000 6.244478e+000

endloop

endfacet

facet normal 0.000000e+000 1.000000e+000 0.000000e+000

outer loop

vertex 1.191674e+001 3.000000e+000 6.244478e+000

vertex 1.095942e+001 3.000000e+000 4.830742e+000

vertex 1.087523e+001 3.000000e+000 4.853300e+000

endloop

endfacet

facet normal 0.000000e+000 1.000000e+000 0.000000e+000

outer loop

vertex 1.191674e+001 3.000000e+000 6.244478e+000

vertex 1.087523e+001 3.000000e+000 4.853300e+000

vertex 1.078841e+001 3.000000e+000 4.860896e+000

endloop

endfacet

facet normal 0.000000e+000 1.000000e+000 0.000000e+000

outer loop

vertex 1.040539e+001 3.000000e+000 1.449454e+000

vertex 1.035540e+001 3.000000e+000 1.378060e+000

vertex 9.452423e+000 3.000000e+000 2.744478e+000

endloop

endfacet

facet normal 0.000000e+000 1.000000e+000 0.000000e+000

outer loop

vertex 1.191674e+001 3.000000e+000 3.140303e-001

vertex 1.128841e+001 3.000000e+000 1.128060e+000

vertex 1.191674e+001 3.000000e+000 2.744478e+000

endloop

endfacet

facet normal 0.000000e+000 1.000000e+000 0.000000e+000

outer loop

vertex 1.191674e+001 3.000000e+000 2.744478e+000

vertex 1.128841e+001 3.000000e+000 1.128060e+000

vertex 1.128081e+001 3.000000e+000 1.214884e+000

endloop

endfacet

facet normal 0.000000e+000 1.000000e+000 0.000000e+000

outer loop

vertex 1.191674e+001 3.000000e+000 2.744478e+000

vertex 1.128081e+001 3.000000e+000 1.214884e+000

vertex 1.125826e+001 3.000000e+000 1.299070e+000

endloop

endfacet

facet normal 0.000000e+000 1.000000e+000 0.000000e+000

outer loop

vertex 1.061740e+001 3.000000e+000 1.597906e+000

vertex 1.053841e+001 3.000000e+000 1.561073e+000

vertex 9.452423e+000 3.000000e+000 2.744478e+000

endloop

endfacet

facet normal 0.000000e+000 1.000000e+000 0.000000e+000

outer loop

vertex 9.452423e+000 3.000000e+000 2.744478e+000

vertex 1.053841e+001 3.000000e+000 1.561073e+000

vertex 1.046702e+001 3.000000e+000 1.511082e+000

endloop

endfacet

facet normal 0.000000e+000 1.000000e+000 0.000000e+000

outer loop

vertex 9.452423e+000 3.000000e+000 2.744478e+000

vertex 1.046702e+001 3.000000e+000 1.511082e+000

vertex 1.040539e+001 3.000000e+000 1.449454e+000

endloop

endfacet

facet normal 0.000000e+000 1.000000e+000 0.000000e+000

outer loop

vertex 1.191674e+001 3.000000e+000 3.140303e-001

vertex 1.095942e+001 3.000000e+000 6.582136e-001

vertex 1.103841e+001 3.000000e+000 6.950474e-001

endloop

endfacet

facet normal 0.000000e+000 1.000000e+000 0.000000e+000

outer loop

vertex 1.125826e+001 3.000000e+000 1.299070e+000

vertex 1.122142e+001 3.000000e+000 1.378060e+000

vertex 1.191674e+001 3.000000e+000 2.744478e+000

endloop

endfacet

facet normal 0.000000e+000 1.000000e+000 0.000000e+000

outer loop

vertex 1.191674e+001 3.000000e+000 2.744478e+000

vertex 1.122142e+001 3.000000e+000 1.378060e+000

vertex 1.117143e+001 3.000000e+000 1.449454e+000

endloop

endfacet

facet normal 0.000000e+000 1.000000e+000 0.000000e+000

outer loop

vertex 1.191674e+001 3.000000e+000 2.744478e+000

vertex 1.117143e+001 3.000000e+000 1.449454e+000

vertex 1.110980e+001 3.000000e+000 1.511082e+000

endloop

endfacet

facet normal 0.000000e+000 1.000000e+000 0.000000e+000

outer loop

vertex 1.191674e+001 3.000000e+000 2.744478e+000

vertex 1.078841e+001 3.000000e+000 1.628060e+000

vertex 9.452423e+000 3.000000e+000 2.744478e+000

endloop

endfacet

facet normal 0.000000e+000 1.000000e+000 0.000000e+000

outer loop

vertex 9.452423e+000 3.000000e+000 2.744478e+000

vertex 1.078841e+001 3.000000e+000 1.628060e+000

vertex 1.070159e+001 3.000000e+000 1.620464e+000

endloop

endfacet

facet normal 0.000000e+000 1.000000e+000 0.000000e+000

outer loop

vertex 9.452423e+000 3.000000e+000 2.744478e+000

vertex 1.070159e+001 3.000000e+000 1.620464e+000

vertex 1.061740e+001 3.000000e+000 1.597906e+000

endloop

endfacet

facet normal 0.000000e+000 1.000000e+000 0.000000e+000

outer loop

vertex 1.029601e+001 3.000000e+000 1.041236e+000

vertex 1.031856e+001 3.000000e+000 9.570499e-001

vertex 9.452423e+000 3.000000e+000 3.140303e-001

endloop

endfacet

facet normal 0.000000e+000 1.000000e+000 0.000000e+000

outer loop

vertex 9.452423e+000 3.000000e+000 3.140303e-001

vertex 1.031856e+001 3.000000e+000 9.570499e-001

vertex 1.035540e+001 3.000000e+000 8.780602e-001

endloop

endfacet

facet normal 0.000000e+000 1.000000e+000 0.000000e+000

outer loop

vertex 9.452423e+000 3.000000e+000 3.140303e-001

vertex 1.035540e+001 3.000000e+000 8.780602e-001

vertex 1.040539e+001 3.000000e+000 8.066660e-001

endloop

endfacet

facet normal 0.000000e+000 1.000000e+000 0.000000e+000

outer loop

vertex 1.103841e+001 3.000000e+000 6.950474e-001

vertex 1.110980e+001 3.000000e+000 7.450376e-001

vertex 1.191674e+001 3.000000e+000 3.140303e-001

endloop

endfacet

facet normal 0.000000e+000 1.000000e+000 0.000000e+000

outer loop

vertex 1.191674e+001 3.000000e+000 3.140303e-001

vertex 1.110980e+001 3.000000e+000 7.450376e-001

vertex 1.117143e+001 3.000000e+000 8.066660e-001

endloop

endfacet

facet normal 0.000000e+000 1.000000e+000 0.000000e+000

outer loop

vertex 1.191674e+001 3.000000e+000 3.140303e-001

vertex 1.117143e+001 3.000000e+000 8.066660e-001

vertex 1.122142e+001 3.000000e+000 8.780602e-001

endloop

endfacet

facet normal 0.000000e+000 1.000000e+000 0.000000e+000

outer loop

vertex 1.122142e+001 3.000000e+000 8.780602e-001

vertex 1.125826e+001 3.000000e+000 9.570499e-001

vertex 1.191674e+001 3.000000e+000 3.140303e-001

endloop

endfacet

facet normal 0.000000e+000 1.000000e+000 0.000000e+000

outer loop

vertex 1.191674e+001 3.000000e+000 3.140303e-001

vertex 1.125826e+001 3.000000e+000 9.570499e-001

vertex 1.128081e+001 3.000000e+000 1.041236e+000

endloop

endfacet

facet normal 0.000000e+000 1.000000e+000 0.000000e+000

outer loop

vertex 1.191674e+001 3.000000e+000 3.140303e-001

vertex 1.128081e+001 3.000000e+000 1.041236e+000

vertex 1.128841e+001 3.000000e+000 1.128060e+000

endloop

endfacet

facet normal 0.000000e+000 1.000000e+000 0.000000e+000

outer loop

vertex 1.110980e+001 3.000000e+000 1.511082e+000

vertex 1.103841e+001 3.000000e+000 1.561073e+000

vertex 1.191674e+001 3.000000e+000 2.744478e+000

endloop

endfacet

facet normal 0.000000e+000 1.000000e+000 0.000000e+000

outer loop

vertex 1.191674e+001 3.000000e+000 2.744478e+000

vertex 1.103841e+001 3.000000e+000 1.561073e+000

vertex 1.095942e+001 3.000000e+000 1.597906e+000

endloop

endfacet

facet normal 0.000000e+000 1.000000e+000 0.000000e+000

outer loop

vertex 1.191674e+001 3.000000e+000 2.744478e+000

vertex 1.095942e+001 3.000000e+000 1.597906e+000

vertex 1.087523e+001 3.000000e+000 1.620464e+000

endloop

endfacet

facet normal 0.000000e+000 1.000000e+000 0.000000e+000

outer loop

vertex 1.040539e+001 3.000000e+000 8.066660e-001

vertex 1.046702e+001 3.000000e+000 7.450376e-001

vertex 9.452423e+000 3.000000e+000 3.140303e-001

endloop

endfacet

facet normal 0.000000e+000 1.000000e+000 0.000000e+000

outer loop

vertex 9.452423e+000 3.000000e+000 3.140303e-001

vertex 1.046702e+001 3.000000e+000 7.450376e-001

vertex 1.053841e+001 3.000000e+000 6.950474e-001

endloop

endfacet

facet normal 0.000000e+000 1.000000e+000 0.000000e+000

outer loop

vertex 9.452423e+000 3.000000e+000 3.140303e-001

vertex 1.053841e+001 3.000000e+000 6.950474e-001

vertex 1.061740e+001 3.000000e+000 6.582136e-001

endloop

endfacet

facet normal 0.000000e+000 1.000000e+000 0.000000e+000

outer loop

vertex 1.035540e+001 3.000000e+000 1.378060e+000

vertex 1.031856e+001 3.000000e+000 1.299070e+000

vertex 9.452423e+000 3.000000e+000 2.744478e+000

endloop

endfacet

facet normal 0.000000e+000 1.000000e+000 0.000000e+000

outer loop

vertex 9.452423e+000 3.000000e+000 2.744478e+000

vertex 1.031856e+001 3.000000e+000 1.299070e+000

vertex 1.029601e+001 3.000000e+000 1.214884e+000

endloop

endfacet

facet normal 0.000000e+000 1.000000e+000 0.000000e+000

outer loop

vertex 9.452423e+000 3.000000e+000 2.744478e+000

vertex 1.029601e+001 3.000000e+000 1.214884e+000

vertex 9.452423e+000 3.000000e+000 3.140303e-001

endloop

endfacet

facet normal 0.000000e+000 1.000000e+000 0.000000e+000

outer loop

vertex 9.452423e+000 3.000000e+000 3.140303e-001

vertex 1.029601e+001 3.000000e+000 1.214884e+000

vertex 1.028841e+001 3.000000e+000 1.128060e+000

endloop

endfacet

facet normal 0.000000e+000 1.000000e+000 0.000000e+000

outer loop

vertex 9.452423e+000 3.000000e+000 3.140303e-001

vertex 1.028841e+001 3.000000e+000 1.128060e+000

vertex 1.029601e+001 3.000000e+000 1.041236e+000

endloop

endfacet

facet normal 0.000000e+000 1.000000e+000 0.000000e+000

outer loop

vertex 1.061740e+001 3.000000e+000 6.582136e-001

vertex 1.070159e+001 3.000000e+000 6.356561e-001

vertex 9.452423e+000 3.000000e+000 3.140303e-001

endloop

endfacet

facet normal 0.000000e+000 1.000000e+000 0.000000e+000

outer loop

vertex 9.452423e+000 3.000000e+000 3.140303e-001

vertex 1.070159e+001 3.000000e+000 6.356561e-001

vertex 1.078841e+001 3.000000e+000 6.280602e-001

endloop

endfacet

facet normal 0.000000e+000 1.000000e+000 0.000000e+000

outer loop

vertex 9.452423e+000 3.000000e+000 3.140303e-001

vertex 1.078841e+001 3.000000e+000 6.280602e-001

vertex 1.191674e+001 3.000000e+000 3.140303e-001

endloop

endfacet

facet normal 0.000000e+000 1.000000e+000 0.000000e+000

outer loop

vertex 1.191674e+001 3.000000e+000 3.140303e-001

vertex 1.078841e+001 3.000000e+000 6.280602e-001

vertex 1.087523e+001 3.000000e+000 6.356561e-001

endloop

endfacet

facet normal 0.000000e+000 1.000000e+000 0.000000e+000

outer loop

vertex 1.191674e+001 3.000000e+000 3.140303e-001

vertex 1.087523e+001 3.000000e+000 6.356561e-001

vertex 1.095942e+001 3.000000e+000 6.582136e-001

endloop

endfacet

facet normal 0.000000e+000 1.000000e+000 0.000000e+000

outer loop

vertex 7.683424e+000 3.000000e+000 1.111090e+001

vertex 7.733414e+000 3.000000e+000 1.103950e+001

vertex 7.594380e+000 3.000000e+000 9.744478e+000

endloop

endfacet

facet normal 0.000000e+000 1.000000e+000 0.000000e+000

outer loop

vertex 7.594380e+000 3.000000e+000 1.251332e+001

vertex 8.029613e+000 3.000000e+000 1.185330e+001

vertex 7.945427e+000 3.000000e+000 1.183074e+001

endloop

endfacet

facet normal 0.000000e+000 1.000000e+000 0.000000e+000

outer loop

vertex 8.287447e+000 3.000000e+000 1.089105e+001

vertex 8.366437e+000 3.000000e+000 1.092788e+001

vertex 9.452423e+000 3.000000e+000 9.744478e+000

endloop

endfacet

facet normal 0.000000e+000 1.000000e+000 0.000000e+000

outer loop

vertex 7.733414e+000 3.000000e+000 1.103950e+001

vertex 7.795043e+000 3.000000e+000 1.097787e+001

vertex 7.594380e+000 3.000000e+000 9.744478e+000

endloop

endfacet

facet normal 0.000000e+000 1.000000e+000 0.000000e+000

outer loop

vertex 7.594380e+000 3.000000e+000 9.744478e+000

vertex 7.795043e+000 3.000000e+000 1.097787e+001

vertex 7.866436e+000 3.000000e+000 1.092788e+001

endloop

endfacet

facet normal 0.000000e+000 1.000000e+000 0.000000e+000

outer loop

vertex 7.594380e+000 3.000000e+000 9.744478e+000

vertex 7.866436e+000 3.000000e+000 1.092788e+001

vertex 7.945427e+000 3.000000e+000 1.089105e+001

endloop

endfacet

facet normal 0.000000e+000 1.000000e+000 0.000000e+000

outer loop

vertex 7.945427e+000 3.000000e+000 1.183074e+001

vertex 7.866436e+000 3.000000e+000 1.179391e+001

vertex 7.594380e+000 3.000000e+000 1.251332e+001

endloop

endfacet

facet normal 0.000000e+000 1.000000e+000 0.000000e+000

outer loop

vertex 7.594380e+000 3.000000e+000 1.251332e+001

vertex 7.866436e+000 3.000000e+000 1.179391e+001

vertex 7.795043e+000 3.000000e+000 1.174392e+001

endloop

endfacet

facet normal 0.000000e+000 1.000000e+000 0.000000e+000

outer loop

vertex 7.594380e+000 3.000000e+000 1.251332e+001

vertex 7.795043e+000 3.000000e+000 1.174392e+001

vertex 7.733414e+000 3.000000e+000 1.168229e+001

endloop

endfacet

facet normal 0.000000e+000 1.000000e+000 0.000000e+000

outer loop

vertex 7.733414e+000 3.000000e+000 1.168229e+001

vertex 7.683424e+000 3.000000e+000 1.161090e+001

vertex 7.594380e+000 3.000000e+000 1.251332e+001

endloop

endfacet

facet normal 0.000000e+000 1.000000e+000 0.000000e+000

outer loop

vertex 7.594380e+000 3.000000e+000 1.251332e+001

vertex 7.683424e+000 3.000000e+000 1.161090e+001

vertex 7.646590e+000 3.000000e+000 1.153191e+001

endloop

endfacet

facet normal 0.000000e+000 1.000000e+000 0.000000e+000

outer loop

vertex 7.594380e+000 3.000000e+000 1.251332e+001

vertex 7.646590e+000 3.000000e+000 1.153191e+001

vertex 7.624032e+000 3.000000e+000 1.144772e+001

endloop

endfacet

facet normal 0.000000e+000 1.000000e+000 0.000000e+000

outer loop

vertex 7.616436e+000 3.000000e+000 1.136090e+001

vertex 7.624032e+000 3.000000e+000 1.127407e+001

vertex 7.594380e+000 3.000000e+000 9.744478e+000

endloop

endfacet

facet normal 0.000000e+000 1.000000e+000 0.000000e+000

outer loop

vertex 7.594380e+000 3.000000e+000 9.744478e+000

vertex 7.624032e+000 3.000000e+000 1.127407e+001

vertex 7.646590e+000 3.000000e+000 1.118989e+001

endloop

endfacet

facet normal 0.000000e+000 1.000000e+000 0.000000e+000

outer loop

vertex 7.594380e+000 3.000000e+000 9.744478e+000

vertex 7.646590e+000 3.000000e+000 1.118989e+001

vertex 7.683424e+000 3.000000e+000 1.111090e+001

endloop

endfacet

facet normal 0.000000e+000 1.000000e+000 0.000000e+000

outer loop

vertex 8.366437e+000 3.000000e+000 1.092788e+001

vertex 8.437830e+000 3.000000e+000 1.097787e+001

vertex 9.452423e+000 3.000000e+000 9.744478e+000

endloop

endfacet

facet normal 0.000000e+000 1.000000e+000 0.000000e+000

outer loop

vertex 9.452423e+000 3.000000e+000 9.744478e+000

vertex 8.437830e+000 3.000000e+000 1.097787e+001

vertex 8.499458e+000 3.000000e+000 1.103950e+001

endloop

endfacet

facet normal 0.000000e+000 1.000000e+000 0.000000e+000

outer loop

vertex 9.452423e+000 3.000000e+000 9.744478e+000

vertex 8.499458e+000 3.000000e+000 1.103950e+001

vertex 8.549449e+000 3.000000e+000 1.111090e+001

endloop

endfacet

facet normal 0.000000e+000 1.000000e+000 0.000000e+000

outer loop

vertex 8.608840e+000 3.000000e+000 1.144772e+001

vertex 8.586283e+000 3.000000e+000 1.153191e+001

vertex 9.452423e+000 3.000000e+000 1.251332e+001

endloop

endfacet

facet normal 0.000000e+000 1.000000e+000 0.000000e+000

outer loop

vertex 9.452423e+000 3.000000e+000 1.251332e+001

vertex 8.586283e+000 3.000000e+000 1.153191e+001

vertex 8.549449e+000 3.000000e+000 1.161090e+001

endloop

endfacet

facet normal 0.000000e+000 1.000000e+000 0.000000e+000

outer loop

vertex 8.549449e+000 3.000000e+000 1.161090e+001

vertex 8.499458e+000 3.000000e+000 1.168229e+001

vertex 9.452423e+000 3.000000e+000 1.251332e+001

endloop

endfacet

facet normal 0.000000e+000 1.000000e+000 0.000000e+000

outer loop

vertex 9.452423e+000 3.000000e+000 1.251332e+001

vertex 8.499458e+000 3.000000e+000 1.168229e+001

vertex 8.437830e+000 3.000000e+000 1.174392e+001

endloop

endfacet

facet normal 0.000000e+000 1.000000e+000 0.000000e+000

outer loop

vertex 9.452423e+000 3.000000e+000 1.251332e+001

vertex 8.437830e+000 3.000000e+000 1.174392e+001

vertex 8.366437e+000 3.000000e+000 1.179391e+001

endloop

endfacet

facet normal 0.000000e+000 1.000000e+000 0.000000e+000

outer loop

vertex 8.366437e+000 3.000000e+000 1.179391e+001

vertex 8.287447e+000 3.000000e+000 1.183074e+001

vertex 9.452423e+000 3.000000e+000 1.251332e+001

endloop

endfacet

facet normal 0.000000e+000 1.000000e+000 0.000000e+000

outer loop

vertex 9.452423e+000 3.000000e+000 1.251332e+001

vertex 8.287447e+000 3.000000e+000 1.183074e+001

vertex 8.203260e+000 3.000000e+000 1.185330e+001

endloop

endfacet

facet normal 0.000000e+000 1.000000e+000 0.000000e+000

outer loop

vertex 9.452423e+000 3.000000e+000 1.251332e+001

vertex 8.203260e+000 3.000000e+000 1.185330e+001

vertex 7.594380e+000 3.000000e+000 1.251332e+001

endloop

endfacet

facet normal 0.000000e+000 1.000000e+000 0.000000e+000

outer loop

vertex 7.594380e+000 3.000000e+000 1.251332e+001

vertex 8.203260e+000 3.000000e+000 1.185330e+001

vertex 8.116436e+000 3.000000e+000 1.186090e+001

endloop

endfacet

facet normal 0.000000e+000 1.000000e+000 0.000000e+000

outer loop

vertex 7.594380e+000 3.000000e+000 1.251332e+001

vertex 8.116436e+000 3.000000e+000 1.186090e+001

vertex 8.029613e+000 3.000000e+000 1.185330e+001

endloop

endfacet

facet normal 0.000000e+000 1.000000e+000 0.000000e+000

outer loop

vertex 7.945427e+000 3.000000e+000 1.089105e+001

vertex 8.029613e+000 3.000000e+000 1.086849e+001

vertex 7.594380e+000 3.000000e+000 9.744478e+000

endloop

endfacet

facet normal 0.000000e+000 1.000000e+000 0.000000e+000

outer loop

vertex 7.594380e+000 3.000000e+000 9.744478e+000

vertex 8.029613e+000 3.000000e+000 1.086849e+001

vertex 8.116436e+000 3.000000e+000 1.086090e+001

endloop

endfacet

facet normal 0.000000e+000 1.000000e+000 0.000000e+000

outer loop

vertex 7.594380e+000 3.000000e+000 9.744478e+000

vertex 8.116436e+000 3.000000e+000 1.086090e+001

vertex 9.452423e+000 3.000000e+000 9.744478e+000

endloop

endfacet

facet normal 0.000000e+000 1.000000e+000 0.000000e+000

outer loop

vertex 9.452423e+000 3.000000e+000 9.744478e+000

vertex 8.116436e+000 3.000000e+000 1.086090e+001

vertex 8.203260e+000 3.000000e+000 1.086849e+001

endloop

endfacet

facet normal 0.000000e+000 1.000000e+000 0.000000e+000

outer loop

vertex 9.452423e+000 3.000000e+000 9.744478e+000

vertex 8.203260e+000 3.000000e+000 1.086849e+001

vertex 8.287447e+000 3.000000e+000 1.089105e+001

endloop

endfacet

facet normal 0.000000e+000 1.000000e+000 0.000000e+000

outer loop

vertex 8.549449e+000 3.000000e+000 1.111090e+001

vertex 8.586283e+000 3.000000e+000 1.118989e+001

vertex 9.452423e+000 3.000000e+000 9.744478e+000

endloop

endfacet

facet normal 0.000000e+000 1.000000e+000 0.000000e+000

outer loop

vertex 9.452423e+000 3.000000e+000 9.744478e+000

vertex 8.586283e+000 3.000000e+000 1.118989e+001

vertex 8.608840e+000 3.000000e+000 1.127407e+001

endloop

endfacet

facet normal 0.000000e+000 1.000000e+000 0.000000e+000

outer loop

vertex 9.452423e+000 3.000000e+000 9.744478e+000

vertex 8.608840e+000 3.000000e+000 1.127407e+001

vertex 9.452423e+000 3.000000e+000 1.251332e+001

endloop

endfacet

facet normal 0.000000e+000 1.000000e+000 0.000000e+000

outer loop

vertex 9.452423e+000 3.000000e+000 1.251332e+001

vertex 8.608840e+000 3.000000e+000 1.127407e+001

vertex 8.616436e+000 3.000000e+000 1.136090e+001

endloop

endfacet

facet normal 0.000000e+000 1.000000e+000 0.000000e+000

outer loop

vertex 9.452423e+000 3.000000e+000 1.251332e+001

vertex 8.616436e+000 3.000000e+000 1.136090e+001

vertex 8.608840e+000 3.000000e+000 1.144772e+001

endloop

endfacet

facet normal 0.000000e+000 1.000000e+000 0.000000e+000

outer loop

vertex 8.029613e+000 3.000000e+000 4.853300e+000

vertex 7.945427e+000 3.000000e+000 4.830742e+000

vertex 7.594380e+000 3.000000e+000 6.244478e+000

endloop

endfacet

facet normal 0.000000e+000 1.000000e+000 0.000000e+000

outer loop

vertex 8.203260e+000 3.000000e+000 3.868492e+000

vertex 8.287447e+000 3.000000e+000 3.891049e+000

vertex 9.452423e+000 3.000000e+000 2.744478e+000

endloop

endfacet

facet normal 0.000000e+000 1.000000e+000 0.000000e+000

outer loop

vertex 9.452423e+000 3.000000e+000 2.744478e+000

vertex 8.287447e+000 3.000000e+000 3.891049e+000

vertex 8.366437e+000 3.000000e+000 3.927883e+000

endloop

endfacet

facet normal 0.000000e+000 1.000000e+000 0.000000e+000

outer loop

vertex 9.452423e+000 3.000000e+000 2.744478e+000

vertex 8.366437e+000 3.000000e+000 3.927883e+000

vertex 8.437830e+000 3.000000e+000 3.977873e+000

endloop

endfacet

facet normal 0.000000e+000 1.000000e+000 0.000000e+000

outer loop

vertex 8.586283e+000 3.000000e+000 4.531906e+000

vertex 8.549449e+000 3.000000e+000 4.610896e+000

vertex 9.452423e+000 3.000000e+000 6.244478e+000

endloop

endfacet

facet normal 0.000000e+000 1.000000e+000 0.000000e+000

outer loop

vertex 7.683424e+000 3.000000e+000 4.110896e+000

vertex 7.733414e+000 3.000000e+000 4.039502e+000

vertex 7.594380e+000 3.000000e+000 2.744478e+000

endloop

endfacet

facet normal 0.000000e+000 1.000000e+000 0.000000e+000

outer loop

vertex 7.594380e+000 3.000000e+000 2.744478e+000

vertex 7.733414e+000 3.000000e+000 4.039502e+000

vertex 7.795043e+000 3.000000e+000 3.977873e+000

endloop

endfacet

facet normal 0.000000e+000 1.000000e+000 0.000000e+000

outer loop

vertex 7.594380e+000 3.000000e+000 2.744478e+000

vertex 7.795043e+000 3.000000e+000 3.977873e+000

vertex 7.866436e+000 3.000000e+000 3.927883e+000

endloop

endfacet

facet normal 0.000000e+000 1.000000e+000 0.000000e+000

outer loop

vertex 8.243334e+000 3.000000e+000 1.597906e+000

vertex 7.594380e+000 3.000000e+000 2.744478e+000

vertex 9.452423e+000 3.000000e+000 2.744478e+000

endloop

endfacet

facet normal 0.000000e+000 1.000000e+000 0.000000e+000

outer loop

vertex 9.452423e+000 3.000000e+000 2.744478e+000

vertex 7.594380e+000 3.000000e+000 2.744478e+000

vertex 8.116436e+000 3.000000e+000 3.860896e+000

endloop

endfacet

facet normal 0.000000e+000 1.000000e+000 0.000000e+000

outer loop

vertex 9.452423e+000 3.000000e+000 2.744478e+000

vertex 8.116436e+000 3.000000e+000 3.860896e+000

vertex 8.203260e+000 3.000000e+000 3.868492e+000

endloop

endfacet

facet normal 0.000000e+000 1.000000e+000 0.000000e+000

outer loop

vertex 7.616436e+000 3.000000e+000 4.360896e+000

vertex 7.624032e+000 3.000000e+000 4.274072e+000

vertex 7.594380e+000 3.000000e+000 2.744478e+000

endloop

endfacet

facet normal 0.000000e+000 1.000000e+000 0.000000e+000

outer loop

vertex 7.594380e+000 3.000000e+000 2.744478e+000

vertex 7.624032e+000 3.000000e+000 4.274072e+000

vertex 7.646590e+000 3.000000e+000 4.189886e+000

endloop

endfacet

facet normal 0.000000e+000 1.000000e+000 0.000000e+000

outer loop

vertex 7.594380e+000 3.000000e+000 2.744478e+000

vertex 7.646590e+000 3.000000e+000 4.189886e+000

vertex 7.683424e+000 3.000000e+000 4.110896e+000

endloop

endfacet

facet normal 0.000000e+000 1.000000e+000 0.000000e+000

outer loop

vertex 7.866436e+000 3.000000e+000 3.927883e+000

vertex 7.945427e+000 3.000000e+000 3.891049e+000

vertex 7.594380e+000 3.000000e+000 2.744478e+000

endloop

endfacet

facet normal 0.000000e+000 1.000000e+000 0.000000e+000

outer loop

vertex 7.594380e+000 3.000000e+000 2.744478e+000

vertex 7.945427e+000 3.000000e+000 3.891049e+000

vertex 8.029613e+000 3.000000e+000 3.868492e+000

endloop

endfacet

facet normal 0.000000e+000 1.000000e+000 0.000000e+000

outer loop

vertex 7.594380e+000 3.000000e+000 2.744478e+000

vertex 8.029613e+000 3.000000e+000 3.868492e+000

vertex 8.116436e+000 3.000000e+000 3.860896e+000

endloop

endfacet

facet normal 0.000000e+000 1.000000e+000 0.000000e+000

outer loop

vertex 7.945427e+000 3.000000e+000 4.830742e+000

vertex 7.866436e+000 3.000000e+000 4.793909e+000

vertex 7.594380e+000 3.000000e+000 6.244478e+000

endloop

endfacet

facet normal 0.000000e+000 1.000000e+000 0.000000e+000

outer loop

vertex 7.594380e+000 3.000000e+000 6.244478e+000

vertex 7.866436e+000 3.000000e+000 4.793909e+000

vertex 7.795043e+000 3.000000e+000 4.743918e+000

endloop

endfacet

facet normal 0.000000e+000 1.000000e+000 0.000000e+000

outer loop

vertex 7.594380e+000 3.000000e+000 6.244478e+000

vertex 7.795043e+000 3.000000e+000 4.743918e+000

vertex 7.733414e+000 3.000000e+000 4.682290e+000

endloop

endfacet

facet normal 0.000000e+000 1.000000e+000 0.000000e+000

outer loop

vertex 7.733414e+000 3.000000e+000 4.682290e+000

vertex 7.683424e+000 3.000000e+000 4.610896e+000

vertex 7.594380e+000 3.000000e+000 6.244478e+000

endloop

endfacet

facet normal 0.000000e+000 1.000000e+000 0.000000e+000

outer loop

vertex 7.594380e+000 3.000000e+000 6.244478e+000

vertex 7.683424e+000 3.000000e+000 4.610896e+000

vertex 7.646590e+000 3.000000e+000 4.531906e+000

endloop

endfacet

facet normal 0.000000e+000 1.000000e+000 0.000000e+000

outer loop

vertex 7.594380e+000 3.000000e+000 6.244478e+000

vertex 7.646590e+000 3.000000e+000 4.531906e+000

vertex 7.624032e+000 3.000000e+000 4.447720e+000

endloop

endfacet

facet normal 0.000000e+000 1.000000e+000 0.000000e+000

outer loop

vertex 8.437830e+000 3.000000e+000 3.977873e+000

vertex 8.499458e+000 3.000000e+000 4.039502e+000

vertex 9.452423e+000 3.000000e+000 2.744478e+000

endloop

endfacet

facet normal 0.000000e+000 1.000000e+000 0.000000e+000

outer loop

vertex 9.452423e+000 3.000000e+000 2.744478e+000

vertex 8.499458e+000 3.000000e+000 4.039502e+000

vertex 8.549449e+000 3.000000e+000 4.110896e+000

endloop

endfacet

facet normal 0.000000e+000 1.000000e+000 0.000000e+000

outer loop

vertex 9.452423e+000 3.000000e+000 2.744478e+000

vertex 8.549449e+000 3.000000e+000 4.110896e+000

vertex 8.586283e+000 3.000000e+000 4.189886e+000

endloop

endfacet

facet normal 0.000000e+000 1.000000e+000 0.000000e+000

outer loop

vertex 8.549449e+000 3.000000e+000 4.610896e+000

vertex 8.499458e+000 3.000000e+000 4.682290e+000

vertex 9.452423e+000 3.000000e+000 6.244478e+000

endloop

endfacet

facet normal 0.000000e+000 1.000000e+000 0.000000e+000

outer loop

vertex 9.452423e+000 3.000000e+000 6.244478e+000

vertex 8.499458e+000 3.000000e+000 4.682290e+000

vertex 8.437830e+000 3.000000e+000 4.743918e+000

endloop

endfacet

facet normal 0.000000e+000 1.000000e+000 0.000000e+000

outer loop

vertex 9.452423e+000 3.000000e+000 6.244478e+000

vertex 8.437830e+000 3.000000e+000 4.743918e+000

vertex 8.366437e+000 3.000000e+000 4.793909e+000

endloop

endfacet

facet normal 0.000000e+000 1.000000e+000 0.000000e+000

outer loop

vertex 8.366437e+000 3.000000e+000 4.793909e+000

vertex 8.287447e+000 3.000000e+000 4.830742e+000

vertex 9.452423e+000 3.000000e+000 6.244478e+000

endloop

endfacet

facet normal 0.000000e+000 1.000000e+000 0.000000e+000

outer loop

vertex 9.452423e+000 3.000000e+000 6.244478e+000

vertex 8.287447e+000 3.000000e+000 4.830742e+000

vertex 8.203260e+000 3.000000e+000 4.853300e+000

endloop

endfacet

facet normal 0.000000e+000 1.000000e+000 0.000000e+000

outer loop

vertex 9.452423e+000 3.000000e+000 6.244478e+000

vertex 8.203260e+000 3.000000e+000 4.853300e+000

vertex 7.594380e+000 3.000000e+000 6.244478e+000

endloop

endfacet

facet normal 0.000000e+000 1.000000e+000 0.000000e+000

outer loop

vertex 7.594380e+000 3.000000e+000 6.244478e+000

vertex 8.203260e+000 3.000000e+000 4.853300e+000

vertex 8.116436e+000 3.000000e+000 4.860896e+000

endloop

endfacet

facet normal 0.000000e+000 1.000000e+000 0.000000e+000

outer loop

vertex 7.594380e+000 3.000000e+000 6.244478e+000

vertex 8.116436e+000 3.000000e+000 4.860896e+000

vertex 8.029613e+000 3.000000e+000 4.853300e+000

endloop

endfacet

facet normal 0.000000e+000 1.000000e+000 0.000000e+000

outer loop

vertex 8.586283e+000 3.000000e+000 4.189886e+000

vertex 8.608840e+000 3.000000e+000 4.274072e+000

vertex 9.452423e+000 3.000000e+000 2.744478e+000

endloop

endfacet

facet normal 0.000000e+000 1.000000e+000 0.000000e+000

outer loop

vertex 9.452423e+000 3.000000e+000 2.744478e+000

vertex 8.608840e+000 3.000000e+000 4.274072e+000

vertex 8.616436e+000 3.000000e+000 4.360896e+000

endloop

endfacet

facet normal 0.000000e+000 1.000000e+000 0.000000e+000

outer loop

vertex 9.452423e+000 3.000000e+000 2.744478e+000

vertex 8.616436e+000 3.000000e+000 4.360896e+000

vertex 9.452423e+000 3.000000e+000 6.244478e+000

endloop

endfacet

facet normal 0.000000e+000 1.000000e+000 0.000000e+000

outer loop

vertex 9.452423e+000 3.000000e+000 6.244478e+000

vertex 8.616436e+000 3.000000e+000 4.360896e+000

vertex 8.608840e+000 3.000000e+000 4.447720e+000

endloop

endfacet

facet normal 0.000000e+000 1.000000e+000 0.000000e+000

outer loop

vertex 9.452423e+000 3.000000e+000 6.244478e+000

vertex 8.608840e+000 3.000000e+000 4.447720e+000

vertex 8.586283e+000 3.000000e+000 4.531906e+000

endloop

endfacet

facet normal 0.000000e+000 1.000000e+000 0.000000e+000

outer loop

vertex 7.624032e+000 3.000000e+000 1.144772e+001

vertex 7.616436e+000 3.000000e+000 1.136090e+001

vertex 7.594380e+000 3.000000e+000 1.251332e+001

endloop

endfacet

facet normal 0.000000e+000 1.000000e+000 0.000000e+000

outer loop

vertex 7.594380e+000 3.000000e+000 1.251332e+001

vertex 7.616436e+000 3.000000e+000 1.136090e+001

vertex 7.594380e+000 3.000000e+000 9.744478e+000

endloop

endfacet

facet normal 0.000000e+000 1.000000e+000 0.000000e+000

outer loop

vertex 7.594380e+000 3.000000e+000 1.251332e+001

vertex 7.594380e+000 3.000000e+000 9.744478e+000

vertex 5.930367e+000 3.000000e+000 1.251332e+001

endloop

endfacet

facet normal 0.000000e+000 1.000000e+000 0.000000e+000

outer loop

vertex 5.930367e+000 3.000000e+000 1.251332e+001

vertex 7.594380e+000 3.000000e+000 9.744478e+000

vertex 5.930367e+000 3.000000e+000 9.744478e+000

endloop

endfacet

facet normal 0.000000e+000 1.000000e+000 0.000000e+000

outer loop

vertex 3.617400e+000 3.000000e+000 1.183074e+001

vertex 2.452423e+000 3.000000e+000 1.251332e+001

vertex 3.701586e+000 3.000000e+000 1.185330e+001

endloop

endfacet

facet normal 0.000000e+000 1.000000e+000 0.000000e+000

outer loop

vertex 3.701586e+000 3.000000e+000 1.185330e+001

vertex 2.452423e+000 3.000000e+000 1.251332e+001

vertex 3.788410e+000 3.000000e+000 1.186090e+001

endloop

endfacet

facet normal 0.000000e+000 1.000000e+000 0.000000e+000

outer loop

vertex 3.617400e+000 3.000000e+000 1.183074e+001

vertex 3.538410e+000 3.000000e+000 1.179391e+001

vertex 2.452423e+000 3.000000e+000 1.251332e+001

endloop

endfacet

facet normal 0.000000e+000 1.000000e+000 0.000000e+000

outer loop

vertex 2.452423e+000 3.000000e+000 1.251332e+001

vertex 3.538410e+000 3.000000e+000 1.179391e+001

vertex 3.467016e+000 3.000000e+000 1.174392e+001

endloop

endfacet

facet normal 0.000000e+000 1.000000e+000 0.000000e+000

outer loop

vertex 2.452423e+000 3.000000e+000 1.251332e+001

vertex 3.467016e+000 3.000000e+000 1.174392e+001

vertex 3.405388e+000 3.000000e+000 1.168229e+001

endloop

endfacet

facet normal 0.000000e+000 1.000000e+000 0.000000e+000

outer loop

vertex 3.405388e+000 3.000000e+000 1.168229e+001

vertex 3.355397e+000 3.000000e+000 1.161090e+001

vertex 2.452423e+000 3.000000e+000 1.251332e+001

endloop

endfacet

facet normal 0.000000e+000 1.000000e+000 0.000000e+000

outer loop

vertex 2.452423e+000 3.000000e+000 1.251332e+001

vertex 3.355397e+000 3.000000e+000 1.161090e+001

vertex 3.318563e+000 3.000000e+000 1.153191e+001

endloop

endfacet

facet normal 0.000000e+000 1.000000e+000 0.000000e+000

outer loop

vertex 2.452423e+000 3.000000e+000 1.251332e+001

vertex 3.318563e+000 3.000000e+000 1.153191e+001

vertex 2.452423e+000 3.000000e+000 9.744478e+000

endloop

endfacet

facet normal 0.000000e+000 1.000000e+000 0.000000e+000

outer loop

vertex 2.452423e+000 3.000000e+000 9.744478e+000

vertex 3.318563e+000 3.000000e+000 1.153191e+001

vertex 3.296006e+000 3.000000e+000 1.144772e+001

endloop

endfacet

facet normal 0.000000e+000 1.000000e+000 0.000000e+000

outer loop

vertex 2.452423e+000 3.000000e+000 9.744478e+000

vertex 3.296006e+000 3.000000e+000 1.144772e+001

vertex 3.288410e+000 3.000000e+000 1.136090e+001

endloop

endfacet

facet normal 0.000000e+000 1.000000e+000 0.000000e+000

outer loop

vertex 3.355397e+000 3.000000e+000 1.111090e+001

vertex 3.405388e+000 3.000000e+000 1.103950e+001

vertex 2.452423e+000 3.000000e+000 9.744478e+000

endloop

endfacet

facet normal 0.000000e+000 1.000000e+000 0.000000e+000

outer loop

vertex 2.452423e+000 3.000000e+000 9.744478e+000

vertex 3.405388e+000 3.000000e+000 1.103950e+001

vertex 3.467016e+000 3.000000e+000 1.097787e+001

endloop

endfacet

facet normal 0.000000e+000 1.000000e+000 0.000000e+000

outer loop

vertex 2.452423e+000 3.000000e+000 9.744478e+000

vertex 3.467016e+000 3.000000e+000 1.097787e+001

vertex 3.538410e+000 3.000000e+000 1.092788e+001

endloop

endfacet

facet normal 0.000000e+000 1.000000e+000 0.000000e+000

outer loop

vertex 3.288410e+000 3.000000e+000 1.136090e+001

vertex 3.296006e+000 3.000000e+000 1.127407e+001

vertex 2.452423e+000 3.000000e+000 9.744478e+000

endloop

endfacet

facet normal 0.000000e+000 1.000000e+000 0.000000e+000

outer loop

vertex 2.452423e+000 3.000000e+000 9.744478e+000

vertex 3.296006e+000 3.000000e+000 1.127407e+001

vertex 3.318563e+000 3.000000e+000 1.118989e+001

endloop

endfacet

facet normal 0.000000e+000 1.000000e+000 0.000000e+000

outer loop

vertex 2.452423e+000 3.000000e+000 9.744478e+000

vertex 3.318563e+000 3.000000e+000 1.118989e+001

vertex 3.355397e+000 3.000000e+000 1.111090e+001

endloop

endfacet

facet normal 0.000000e+000 1.000000e+000 0.000000e+000

outer loop

vertex 3.538410e+000 3.000000e+000 1.092788e+001

vertex 3.617400e+000 3.000000e+000 1.089105e+001

vertex 2.452423e+000 3.000000e+000 9.744478e+000

endloop

endfacet

facet normal 0.000000e+000 1.000000e+000 0.000000e+000

outer loop

vertex 2.452423e+000 3.000000e+000 9.744478e+000

vertex 3.617400e+000 3.000000e+000 1.089105e+001

vertex 3.701586e+000 3.000000e+000 1.086849e+001

endloop

endfacet

facet normal 0.000000e+000 1.000000e+000 0.000000e+000

outer loop

vertex 2.452423e+000 3.000000e+000 9.744478e+000

vertex 3.701586e+000 3.000000e+000 1.086849e+001

vertex 3.788410e+000 3.000000e+000 1.086090e+001

endloop

endfacet

facet normal 0.000000e+000 1.000000e+000 0.000000e+000

outer loop

vertex 3.788410e+000 3.000000e+000 1.186090e+001

vertex 2.452423e+000 3.000000e+000 1.251332e+001

vertex 3.875234e+000 3.000000e+000 1.185330e+001

endloop

endfacet

facet normal 0.000000e+000 1.000000e+000 0.000000e+000

outer loop

vertex 3.875234e+000 3.000000e+000 1.185330e+001

vertex 2.452423e+000 3.000000e+000 1.251332e+001

vertex 5.930367e+000 3.000000e+000 1.251332e+001

endloop

endfacet

facet normal 0.000000e+000 1.000000e+000 0.000000e+000

outer loop

vertex 3.875234e+000 3.000000e+000 1.185330e+001

vertex 5.930367e+000 3.000000e+000 1.251332e+001

vertex 3.959420e+000 3.000000e+000 1.183074e+001

endloop

endfacet

facet normal 0.000000e+000 1.000000e+000 0.000000e+000

outer loop

vertex 3.788410e+000 3.000000e+000 1.086090e+001

vertex 3.875234e+000 3.000000e+000 1.086849e+001

vertex 5.930367e+000 3.000000e+000 9.744478e+000

endloop

endfacet

facet normal 0.000000e+000 1.000000e+000 0.000000e+000

outer loop

vertex 5.930367e+000 3.000000e+000 9.744478e+000

vertex 3.875234e+000 3.000000e+000 1.086849e+001

vertex 3.959420e+000 3.000000e+000 1.089105e+001

endloop

endfacet

facet normal 0.000000e+000 1.000000e+000 0.000000e+000

outer loop

vertex 5.930367e+000 3.000000e+000 9.744478e+000

vertex 3.959420e+000 3.000000e+000 1.089105e+001

vertex 4.038410e+000 3.000000e+000 1.092788e+001

endloop

endfacet

facet normal 0.000000e+000 1.000000e+000 0.000000e+000

outer loop

vertex 4.038410e+000 3.000000e+000 1.092788e+001

vertex 4.109804e+000 3.000000e+000 1.097787e+001

vertex 5.930367e+000 3.000000e+000 9.744478e+000

endloop

endfacet

facet normal 0.000000e+000 1.000000e+000 0.000000e+000

outer loop

vertex 5.930367e+000 3.000000e+000 9.744478e+000

vertex 4.109804e+000 3.000000e+000 1.097787e+001

vertex 4.171432e+000 3.000000e+000 1.103950e+001

endloop

endfacet

facet normal 0.000000e+000 1.000000e+000 0.000000e+000

outer loop

vertex 5.930367e+000 3.000000e+000 9.744478e+000

vertex 4.171432e+000 3.000000e+000 1.103950e+001

vertex 4.221423e+000 3.000000e+000 1.111090e+001

endloop

endfacet

facet normal 0.000000e+000 1.000000e+000 0.000000e+000

outer loop

vertex 4.280814e+000 3.000000e+000 1.144772e+001

vertex 4.258256e+000 3.000000e+000 1.153191e+001

vertex 5.930367e+000 3.000000e+000 1.251332e+001

endloop

endfacet

facet normal 0.000000e+000 1.000000e+000 0.000000e+000

outer loop

vertex 5.930367e+000 3.000000e+000 1.251332e+001

vertex 4.258256e+000 3.000000e+000 1.153191e+001

vertex 4.221423e+000 3.000000e+000 1.161090e+001

endloop

endfacet

facet normal 0.000000e+000 1.000000e+000 0.000000e+000

outer loop

vertex 5.930367e+000 3.000000e+000 1.251332e+001

vertex 4.221423e+000 3.000000e+000 1.161090e+001

vertex 4.171432e+000 3.000000e+000 1.168229e+001

endloop

endfacet

facet normal 0.000000e+000 1.000000e+000 0.000000e+000

outer loop

vertex 4.171432e+000 3.000000e+000 1.168229e+001

vertex 4.109804e+000 3.000000e+000 1.174392e+001

vertex 5.930367e+000 3.000000e+000 1.251332e+001

endloop

endfacet

facet normal 0.000000e+000 1.000000e+000 0.000000e+000

outer loop

vertex 5.930367e+000 3.000000e+000 1.251332e+001

vertex 4.109804e+000 3.000000e+000 1.174392e+001

vertex 4.038410e+000 3.000000e+000 1.179391e+001

endloop

endfacet

facet normal 0.000000e+000 1.000000e+000 0.000000e+000

outer loop

vertex 5.930367e+000 3.000000e+000 1.251332e+001

vertex 4.038410e+000 3.000000e+000 1.179391e+001

vertex 3.959420e+000 3.000000e+000 1.183074e+001

endloop

endfacet

facet normal 0.000000e+000 1.000000e+000 0.000000e+000

outer loop

vertex 4.221423e+000 3.000000e+000 1.111090e+001

vertex 4.258256e+000 3.000000e+000 1.118989e+001

vertex 5.930367e+000 3.000000e+000 9.744478e+000

endloop

endfacet

facet normal 0.000000e+000 1.000000e+000 0.000000e+000

outer loop

vertex 5.930367e+000 3.000000e+000 9.744478e+000

vertex 4.258256e+000 3.000000e+000 1.118989e+001

vertex 4.280814e+000 3.000000e+000 1.127407e+001

endloop

endfacet

facet normal 0.000000e+000 1.000000e+000 0.000000e+000

outer loop

vertex 5.930367e+000 3.000000e+000 9.744478e+000

vertex 4.280814e+000 3.000000e+000 1.127407e+001

vertex 5.930367e+000 3.000000e+000 1.251332e+001

endloop

endfacet

facet normal 0.000000e+000 1.000000e+000 0.000000e+000

outer loop

vertex 5.930367e+000 3.000000e+000 1.251332e+001

vertex 4.280814e+000 3.000000e+000 1.127407e+001

vertex 4.288410e+000 3.000000e+000 1.136090e+001

endloop

endfacet

facet normal 0.000000e+000 1.000000e+000 0.000000e+000

outer loop

vertex 5.930367e+000 3.000000e+000 1.251332e+001

vertex 4.288410e+000 3.000000e+000 1.136090e+001

vertex 4.280814e+000 3.000000e+000 1.144772e+001

endloop

endfacet

facet normal 0.000000e+000 1.000000e+000 0.000000e+000

outer loop

vertex 3.405388e+000 3.000000e+000 8.449454e+000

vertex 3.355397e+000 3.000000e+000 8.378060e+000

vertex 2.452423e+000 3.000000e+000 9.744478e+000

endloop

endfacet

facet normal 0.000000e+000 1.000000e+000 0.000000e+000

outer loop

vertex 2.452423e+000 3.000000e+000 9.744478e+000

vertex 3.355397e+000 3.000000e+000 8.378060e+000

vertex 3.318563e+000 3.000000e+000 8.299070e+000

endloop

endfacet

facet normal 0.000000e+000 1.000000e+000 0.000000e+000

outer loop

vertex 3.617400e+000 3.000000e+000 8.597907e+000

vertex 2.452423e+000 3.000000e+000 9.744478e+000

vertex 3.701586e+000 3.000000e+000 8.620463e+000

endloop

endfacet

facet normal 0.000000e+000 1.000000e+000 0.000000e+000

outer loop

vertex 3.701586e+000 3.000000e+000 8.620463e+000

vertex 2.452423e+000 3.000000e+000 9.744478e+000

vertex 3.788410e+000 3.000000e+000 8.628060e+000

endloop

endfacet

facet normal 0.000000e+000 1.000000e+000 0.000000e+000

outer loop

vertex 3.617400e+000 3.000000e+000 8.597907e+000

vertex 3.538410e+000 3.000000e+000 8.561073e+000

vertex 2.452423e+000 3.000000e+000 9.744478e+000

endloop

endfacet

facet normal 0.000000e+000 1.000000e+000 0.000000e+000

outer loop

vertex 2.452423e+000 3.000000e+000 9.744478e+000

vertex 3.538410e+000 3.000000e+000 8.561073e+000

vertex 3.467016e+000 3.000000e+000 8.511083e+000

endloop

endfacet

facet normal 0.000000e+000 1.000000e+000 0.000000e+000

outer loop

vertex 2.452423e+000 3.000000e+000 9.744478e+000

vertex 3.467016e+000 3.000000e+000 8.511083e+000

vertex 3.405388e+000 3.000000e+000 8.449454e+000

endloop

endfacet

facet normal 0.000000e+000 1.000000e+000 0.000000e+000

outer loop

vertex 3.318563e+000 3.000000e+000 7.957050e+000

vertex 3.355397e+000 3.000000e+000 7.878059e+000

vertex 2.452423e+000 3.000000e+000 6.244478e+000

endloop

endfacet

facet normal 0.000000e+000 1.000000e+000 0.000000e+000

outer loop

vertex 2.452423e+000 3.000000e+000 6.244478e+000

vertex 3.355397e+000 3.000000e+000 7.878059e+000

vertex 3.405388e+000 3.000000e+000 7.806666e+000

endloop

endfacet

facet normal 0.000000e+000 1.000000e+000 0.000000e+000

outer loop

vertex 3.405388e+000 3.000000e+000 7.806666e+000

vertex 3.467016e+000 3.000000e+000 7.745038e+000

vertex 2.452423e+000 3.000000e+000 6.244478e+000

endloop

endfacet

facet normal 0.000000e+000 1.000000e+000 0.000000e+000

outer loop

vertex 2.452423e+000 3.000000e+000 6.244478e+000

vertex 3.467016e+000 3.000000e+000 7.745038e+000

vertex 3.538410e+000 3.000000e+000 7.695047e+000

endloop

endfacet

facet normal 0.000000e+000 1.000000e+000 0.000000e+000

outer loop

vertex 2.452423e+000 3.000000e+000 6.244478e+000

vertex 3.538410e+000 3.000000e+000 7.695047e+000

vertex 3.617400e+000 3.000000e+000 7.658214e+000

endloop

endfacet

facet normal 0.000000e+000 1.000000e+000 0.000000e+000

outer loop

vertex 5.930367e+000 3.000000e+000 6.244478e+000

vertex 3.959420e+000 3.000000e+000 7.658214e+000

vertex 4.038410e+000 3.000000e+000 7.695047e+000

endloop

endfacet

facet normal 0.000000e+000 1.000000e+000 0.000000e+000

outer loop

vertex 4.280814e+000 3.000000e+000 8.214885e+000

vertex 4.258256e+000 3.000000e+000 8.299070e+000

vertex 5.930367e+000 3.000000e+000 9.744478e+000

endloop

endfacet

facet normal 0.000000e+000 1.000000e+000 0.000000e+000

outer loop

vertex 3.318563e+000 3.000000e+000 8.299070e+000

vertex 3.296006e+000 3.000000e+000 8.214885e+000

vertex 2.452423e+000 3.000000e+000 9.744478e+000

endloop

endfacet

facet normal 0.000000e+000 1.000000e+000 0.000000e+000

outer loop

vertex 2.452423e+000 3.000000e+000 9.744478e+000

vertex 3.296006e+000 3.000000e+000 8.214885e+000

vertex 3.288410e+000 3.000000e+000 8.128060e+000

endloop

endfacet

facet normal 0.000000e+000 1.000000e+000 0.000000e+000

outer loop

vertex 2.452423e+000 3.000000e+000 9.744478e+000

vertex 3.288410e+000 3.000000e+000 8.128060e+000

vertex 2.452423e+000 3.000000e+000 6.244478e+000

endloop

endfacet

facet normal 0.000000e+000 1.000000e+000 0.000000e+000

outer loop

vertex 2.452423e+000 3.000000e+000 6.244478e+000

vertex 3.288410e+000 3.000000e+000 8.128060e+000

vertex 3.296006e+000 3.000000e+000 8.041236e+000

endloop

endfacet

facet normal 0.000000e+000 1.000000e+000 0.000000e+000

outer loop

vertex 2.452423e+000 3.000000e+000 6.244478e+000

vertex 3.296006e+000 3.000000e+000 8.041236e+000

vertex 3.318563e+000 3.000000e+000 7.957050e+000

endloop

endfacet

facet normal 0.000000e+000 1.000000e+000 0.000000e+000

outer loop

vertex 3.617400e+000 3.000000e+000 7.658214e+000

vertex 3.701586e+000 3.000000e+000 7.635656e+000

vertex 2.452423e+000 3.000000e+000 6.244478e+000

endloop

endfacet

facet normal 0.000000e+000 1.000000e+000 0.000000e+000

outer loop

vertex 2.452423e+000 3.000000e+000 6.244478e+000

vertex 3.701586e+000 3.000000e+000 7.635656e+000

vertex 3.788410e+000 3.000000e+000 7.628060e+000

endloop

endfacet

facet normal 0.000000e+000 1.000000e+000 0.000000e+000

outer loop

vertex 2.452423e+000 3.000000e+000 6.244478e+000

vertex 3.788410e+000 3.000000e+000 7.628060e+000

vertex 5.930367e+000 3.000000e+000 6.244478e+000

endloop

endfacet

facet normal 0.000000e+000 1.000000e+000 0.000000e+000

outer loop

vertex 5.930367e+000 3.000000e+000 6.244478e+000

vertex 3.788410e+000 3.000000e+000 7.628060e+000

vertex 3.875234e+000 3.000000e+000 7.635656e+000

endloop

endfacet

facet normal 0.000000e+000 1.000000e+000 0.000000e+000

outer loop

vertex 5.930367e+000 3.000000e+000 6.244478e+000

vertex 3.875234e+000 3.000000e+000 7.635656e+000

vertex 3.959420e+000 3.000000e+000 7.658214e+000

endloop

endfacet

facet normal 0.000000e+000 1.000000e+000 0.000000e+000

outer loop

vertex 4.038410e+000 3.000000e+000 7.695047e+000

vertex 4.109804e+000 3.000000e+000 7.745038e+000

vertex 5.930367e+000 3.000000e+000 6.244478e+000

endloop

endfacet

facet normal 0.000000e+000 1.000000e+000 0.000000e+000

outer loop

vertex 5.930367e+000 3.000000e+000 6.244478e+000

vertex 4.109804e+000 3.000000e+000 7.745038e+000

vertex 4.171432e+000 3.000000e+000 7.806666e+000

endloop

endfacet

facet normal 0.000000e+000 1.000000e+000 0.000000e+000

outer loop

vertex 5.930367e+000 3.000000e+000 6.244478e+000

vertex 4.171432e+000 3.000000e+000 7.806666e+000

vertex 4.221423e+000 3.000000e+000 7.878059e+000

endloop

endfacet

facet normal 0.000000e+000 1.000000e+000 0.000000e+000

outer loop

vertex 3.788410e+000 3.000000e+000 1.086090e+001

vertex 5.930367e+000 3.000000e+000 9.744478e+000

vertex 2.452423e+000 3.000000e+000 9.744478e+000

endloop

endfacet

facet normal 0.000000e+000 1.000000e+000 0.000000e+000

outer loop

vertex 2.452423e+000 3.000000e+000 9.744478e+000

vertex 5.930367e+000 3.000000e+000 9.744478e+000

vertex 3.875234e+000 3.000000e+000 8.620463e+000

endloop

endfacet

facet normal 0.000000e+000 1.000000e+000 0.000000e+000

outer loop

vertex 2.452423e+000 3.000000e+000 9.744478e+000

vertex 3.875234e+000 3.000000e+000 8.620463e+000

vertex 3.788410e+000 3.000000e+000 8.628060e+000

endloop

endfacet

facet normal 0.000000e+000 1.000000e+000 0.000000e+000

outer loop

vertex 4.258256e+000 3.000000e+000 8.299070e+000

vertex 4.221423e+000 3.000000e+000 8.378060e+000

vertex 5.930367e+000 3.000000e+000 9.744478e+000

endloop

endfacet

facet normal 0.000000e+000 1.000000e+000 0.000000e+000

outer loop

vertex 5.930367e+000 3.000000e+000 9.744478e+000

vertex 4.221423e+000 3.000000e+000 8.378060e+000

vertex 4.171432e+000 3.000000e+000 8.449454e+000

endloop

endfacet

facet normal 0.000000e+000 1.000000e+000 0.000000e+000

outer loop

vertex 5.930367e+000 3.000000e+000 9.744478e+000

vertex 4.171432e+000 3.000000e+000 8.449454e+000

vertex 4.109804e+000 3.000000e+000 8.511083e+000

endloop

endfacet

facet normal 0.000000e+000 1.000000e+000 0.000000e+000

outer loop

vertex 4.221423e+000 3.000000e+000 7.878059e+000

vertex 4.258256e+000 3.000000e+000 7.957050e+000

vertex 5.930367e+000 3.000000e+000 6.244478e+000

endloop

endfacet

facet normal 0.000000e+000 1.000000e+000 0.000000e+000

outer loop

vertex 5.930367e+000 3.000000e+000 6.244478e+000

vertex 4.258256e+000 3.000000e+000 7.957050e+000

vertex 4.280814e+000 3.000000e+000 8.041236e+000

endloop

endfacet

facet normal 0.000000e+000 1.000000e+000 0.000000e+000

outer loop

vertex 5.930367e+000 3.000000e+000 6.244478e+000

vertex 4.280814e+000 3.000000e+000 8.041236e+000

vertex 5.930367e+000 3.000000e+000 9.744478e+000

endloop

endfacet

facet normal 0.000000e+000 1.000000e+000 0.000000e+000

outer loop

vertex 5.930367e+000 3.000000e+000 9.744478e+000

vertex 4.280814e+000 3.000000e+000 8.041236e+000

vertex 4.288410e+000 3.000000e+000 8.128060e+000

endloop

endfacet

facet normal 0.000000e+000 1.000000e+000 0.000000e+000

outer loop

vertex 5.930367e+000 3.000000e+000 9.744478e+000

vertex 4.288410e+000 3.000000e+000 8.128060e+000

vertex 4.280814e+000 3.000000e+000 8.214885e+000

endloop

endfacet

facet normal 0.000000e+000 1.000000e+000 0.000000e+000

outer loop

vertex 4.109804e+000 3.000000e+000 8.511083e+000

vertex 4.038410e+000 3.000000e+000 8.561073e+000

vertex 5.930367e+000 3.000000e+000 9.744478e+000

endloop

endfacet

facet normal 0.000000e+000 1.000000e+000 0.000000e+000

outer loop

vertex 5.930367e+000 3.000000e+000 9.744478e+000

vertex 4.038410e+000 3.000000e+000 8.561073e+000

vertex 3.959420e+000 3.000000e+000 8.597907e+000

endloop

endfacet

facet normal 0.000000e+000 1.000000e+000 0.000000e+000

outer loop

vertex 5.930367e+000 3.000000e+000 9.744478e+000

vertex 3.959420e+000 3.000000e+000 8.597907e+000

vertex 3.875234e+000 3.000000e+000 8.620463e+000

endloop

endfacet

facet normal 0.000000e+000 1.000000e+000 0.000000e+000

outer loop

vertex 3.701586e+000 3.000000e+000 4.853300e+000

vertex 3.617400e+000 3.000000e+000 4.830742e+000

vertex 2.452423e+000 3.000000e+000 6.244478e+000

endloop

endfacet

facet normal 0.000000e+000 1.000000e+000 0.000000e+000

outer loop

vertex 2.452423e+000 3.000000e+000 6.244478e+000

vertex 3.617400e+000 3.000000e+000 4.830742e+000

vertex 3.538410e+000 3.000000e+000 4.793909e+000

endloop

endfacet

facet normal 0.000000e+000 1.000000e+000 0.000000e+000

outer loop

vertex 3.788410e+000 3.000000e+000 3.860896e+000

vertex 3.875234e+000 3.000000e+000 3.868492e+000

vertex 5.930367e+000 3.000000e+000 2.744478e+000

endloop

endfacet

facet normal 0.000000e+000 1.000000e+000 0.000000e+000

outer loop

vertex 4.258256e+000 3.000000e+000 4.189886e+000

vertex 4.280814e+000 3.000000e+000 4.274072e+000

vertex 5.930367e+000 3.000000e+000 2.744478e+000

endloop

endfacet

facet normal 0.000000e+000 1.000000e+000 0.000000e+000

outer loop

vertex 3.538410e+000 3.000000e+000 4.793909e+000

vertex 3.467016e+000 3.000000e+000 4.743918e+000

vertex 2.452423e+000 3.000000e+000 6.244478e+000

endloop

endfacet

facet normal 0.000000e+000 1.000000e+000 0.000000e+000

outer loop

vertex 2.452423e+000 3.000000e+000 6.244478e+000

vertex 3.467016e+000 3.000000e+000 4.743918e+000

vertex 3.405388e+000 3.000000e+000 4.682290e+000

endloop

endfacet

facet normal 0.000000e+000 1.000000e+000 0.000000e+000

outer loop

vertex 2.452423e+000 3.000000e+000 6.244478e+000

vertex 3.405388e+000 3.000000e+000 4.682290e+000

vertex 3.355397e+000 3.000000e+000 4.610896e+000

endloop

endfacet

facet normal 0.000000e+000 1.000000e+000 0.000000e+000

outer loop

vertex 3.296006e+000 3.000000e+000 4.274072e+000

vertex 3.318563e+000 3.000000e+000 4.189886e+000

vertex 2.452423e+000 3.000000e+000 2.744478e+000

endloop

endfacet

facet normal 0.000000e+000 1.000000e+000 0.000000e+000

outer loop

vertex 2.452423e+000 3.000000e+000 2.744478e+000

vertex 3.318563e+000 3.000000e+000 4.189886e+000

vertex 3.355397e+000 3.000000e+000 4.110896e+000

endloop

endfacet

facet normal 0.000000e+000 1.000000e+000 0.000000e+000

outer loop

vertex 3.355397e+000 3.000000e+000 4.110896e+000

vertex 3.405388e+000 3.000000e+000 4.039502e+000

vertex 2.452423e+000 3.000000e+000 2.744478e+000

endloop

endfacet

facet normal 0.000000e+000 1.000000e+000 0.000000e+000

outer loop

vertex 2.452423e+000 3.000000e+000 2.744478e+000

vertex 3.405388e+000 3.000000e+000 4.039502e+000

vertex 3.467016e+000 3.000000e+000 3.977873e+000

endloop

endfacet

facet normal 0.000000e+000 1.000000e+000 0.000000e+000

outer loop

vertex 2.452423e+000 3.000000e+000 2.744478e+000

vertex 3.467016e+000 3.000000e+000 3.977873e+000

vertex 3.538410e+000 3.000000e+000 3.927883e+000

endloop

endfacet

facet normal 0.000000e+000 1.000000e+000 0.000000e+000

outer loop

vertex 4.109804e+000 3.000000e+000 3.977873e+000

vertex 4.171432e+000 3.000000e+000 4.039502e+000

vertex 5.930367e+000 3.000000e+000 2.744478e+000

endloop

endfacet

facet normal 0.000000e+000 1.000000e+000 0.000000e+000

outer loop

vertex 5.930367e+000 3.000000e+000 2.744478e+000

vertex 4.171432e+000 3.000000e+000 4.039502e+000

vertex 4.221423e+000 3.000000e+000 4.110896e+000

endloop

endfacet

facet normal 0.000000e+000 1.000000e+000 0.000000e+000

outer loop

vertex 5.930367e+000 3.000000e+000 2.744478e+000

vertex 4.221423e+000 3.000000e+000 4.110896e+000

vertex 4.258256e+000 3.000000e+000 4.189886e+000

endloop

endfacet

facet normal 0.000000e+000 1.000000e+000 0.000000e+000

outer loop

vertex 4.288410e+000 3.000000e+000 4.360896e+000

vertex 4.280814e+000 3.000000e+000 4.447720e+000

vertex 5.930367e+000 3.000000e+000 6.244478e+000

endloop

endfacet

facet normal 0.000000e+000 1.000000e+000 0.000000e+000

outer loop

vertex 5.930367e+000 3.000000e+000 6.244478e+000

vertex 4.280814e+000 3.000000e+000 4.447720e+000

vertex 4.258256e+000 3.000000e+000 4.531906e+000

endloop

endfacet

facet normal 0.000000e+000 1.000000e+000 0.000000e+000

outer loop

vertex 5.930367e+000 3.000000e+000 6.244478e+000

vertex 4.258256e+000 3.000000e+000 4.531906e+000

vertex 4.221423e+000 3.000000e+000 4.610896e+000

endloop

endfacet

facet normal 0.000000e+000 1.000000e+000 0.000000e+000

outer loop

vertex 4.221423e+000 3.000000e+000 4.610896e+000

vertex 4.171432e+000 3.000000e+000 4.682290e+000

vertex 5.930367e+000 3.000000e+000 6.244478e+000

endloop

endfacet

facet normal 0.000000e+000 1.000000e+000 0.000000e+000

outer loop

vertex 5.930367e+000 3.000000e+000 6.244478e+000

vertex 4.171432e+000 3.000000e+000 4.682290e+000

vertex 4.109804e+000 3.000000e+000 4.743918e+000

endloop

endfacet

facet normal 0.000000e+000 1.000000e+000 0.000000e+000

outer loop

vertex 5.930367e+000 3.000000e+000 6.244478e+000

vertex 4.109804e+000 3.000000e+000 4.743918e+000

vertex 4.038410e+000 3.000000e+000 4.793909e+000

endloop

endfacet

facet normal 0.000000e+000 1.000000e+000 0.000000e+000

outer loop

vertex 3.355397e+000 3.000000e+000 4.610896e+000

vertex 3.318563e+000 3.000000e+000 4.531906e+000

vertex 2.452423e+000 3.000000e+000 6.244478e+000

endloop

endfacet

facet normal 0.000000e+000 1.000000e+000 0.000000e+000

outer loop

vertex 2.452423e+000 3.000000e+000 6.244478e+000

vertex 3.318563e+000 3.000000e+000 4.531906e+000

vertex 3.296006e+000 3.000000e+000 4.447720e+000

endloop

endfacet

facet normal 0.000000e+000 1.000000e+000 0.000000e+000

outer loop

vertex 2.452423e+000 3.000000e+000 6.244478e+000

vertex 3.296006e+000 3.000000e+000 4.447720e+000

vertex 2.452423e+000 3.000000e+000 2.744478e+000

endloop

endfacet

facet normal 0.000000e+000 1.000000e+000 0.000000e+000

outer loop

vertex 2.452423e+000 3.000000e+000 2.744478e+000

vertex 3.296006e+000 3.000000e+000 4.447720e+000

vertex 3.288410e+000 3.000000e+000 4.360896e+000

endloop

endfacet

facet normal 0.000000e+000 1.000000e+000 0.000000e+000

outer loop

vertex 2.452423e+000 3.000000e+000 2.744478e+000

vertex 3.288410e+000 3.000000e+000 4.360896e+000

vertex 3.296006e+000 3.000000e+000 4.274072e+000

endloop

endfacet

facet normal 0.000000e+000 1.000000e+000 0.000000e+000

outer loop

vertex 3.538410e+000 3.000000e+000 3.927883e+000

vertex 3.617400e+000 3.000000e+000 3.891049e+000

vertex 2.452423e+000 3.000000e+000 2.744478e+000

endloop

endfacet

facet normal 0.000000e+000 1.000000e+000 0.000000e+000

outer loop

vertex 2.452423e+000 3.000000e+000 2.744478e+000

vertex 3.617400e+000 3.000000e+000 3.891049e+000

vertex 3.701586e+000 3.000000e+000 3.868492e+000

endloop

endfacet

facet normal 0.000000e+000 1.000000e+000 0.000000e+000

outer loop

vertex 2.452423e+000 3.000000e+000 2.744478e+000

vertex 3.701586e+000 3.000000e+000 3.868492e+000

vertex 3.788410e+000 3.000000e+000 3.860896e+000

endloop

endfacet

facet normal 0.000000e+000 1.000000e+000 0.000000e+000

outer loop

vertex 3.875234e+000 3.000000e+000 3.868492e+000

vertex 3.959420e+000 3.000000e+000 3.891049e+000

vertex 5.930367e+000 3.000000e+000 2.744478e+000

endloop

endfacet

facet normal 0.000000e+000 1.000000e+000 0.000000e+000

outer loop

vertex 5.930367e+000 3.000000e+000 2.744478e+000

vertex 3.959420e+000 3.000000e+000 3.891049e+000

vertex 4.038410e+000 3.000000e+000 3.927883e+000

endloop

endfacet

facet normal 0.000000e+000 1.000000e+000 0.000000e+000

outer loop

vertex 5.930367e+000 3.000000e+000 2.744478e+000

vertex 4.038410e+000 3.000000e+000 3.927883e+000

vertex 4.109804e+000 3.000000e+000 3.977873e+000

endloop

endfacet

facet normal 0.000000e+000 1.000000e+000 0.000000e+000

outer loop

vertex 4.280814e+000 3.000000e+000 4.274072e+000

vertex 4.288410e+000 3.000000e+000 4.360896e+000

vertex 5.930367e+000 3.000000e+000 2.744478e+000

endloop

endfacet

facet normal 0.000000e+000 1.000000e+000 0.000000e+000

outer loop

vertex 5.930367e+000 3.000000e+000 2.744478e+000

vertex 4.288410e+000 3.000000e+000 4.360896e+000

vertex 5.930367e+000 3.000000e+000 6.244478e+000

endloop

endfacet

facet normal 0.000000e+000 1.000000e+000 0.000000e+000

outer loop

vertex 5.930367e+000 3.000000e+000 2.744478e+000

vertex 5.930367e+000 3.000000e+000 6.244478e+000

vertex 7.594380e+000 3.000000e+000 2.744478e+000

endloop

endfacet

facet normal 0.000000e+000 1.000000e+000 0.000000e+000

outer loop

vertex 7.594380e+000 3.000000e+000 2.744478e+000

vertex 5.930367e+000 3.000000e+000 6.244478e+000

vertex 7.594380e+000 3.000000e+000 6.244478e+000

endloop

endfacet

facet normal 0.000000e+000 1.000000e+000 0.000000e+000

outer loop

vertex 7.594380e+000 3.000000e+000 2.744478e+000

vertex 7.594380e+000 3.000000e+000 6.244478e+000

vertex 7.616436e+000 3.000000e+000 4.360896e+000

endloop

endfacet

facet normal 0.000000e+000 1.000000e+000 0.000000e+000

outer loop

vertex 7.616436e+000 3.000000e+000 4.360896e+000

vertex 7.594380e+000 3.000000e+000 6.244478e+000

vertex 7.624032e+000 3.000000e+000 4.447720e+000

endloop

endfacet

facet normal 0.000000e+000 1.000000e+000 0.000000e+000

outer loop

vertex 4.038410e+000 3.000000e+000 4.793909e+000

vertex 3.959420e+000 3.000000e+000 4.830742e+000

vertex 5.930367e+000 3.000000e+000 6.244478e+000

endloop

endfacet

facet normal 0.000000e+000 1.000000e+000 0.000000e+000

outer loop

vertex 5.930367e+000 3.000000e+000 6.244478e+000

vertex 3.959420e+000 3.000000e+000 4.830742e+000

vertex 3.875234e+000 3.000000e+000 4.853300e+000

endloop

endfacet

facet normal 0.000000e+000 1.000000e+000 0.000000e+000

outer loop

vertex 5.930367e+000 3.000000e+000 6.244478e+000

vertex 3.875234e+000 3.000000e+000 4.853300e+000

vertex 2.452423e+000 3.000000e+000 6.244478e+000

endloop

endfacet

facet normal 0.000000e+000 1.000000e+000 0.000000e+000

outer loop

vertex 2.452423e+000 3.000000e+000 6.244478e+000

vertex 3.875234e+000 3.000000e+000 4.853300e+000

vertex 3.788410e+000 3.000000e+000 4.860896e+000

endloop

endfacet

facet normal 0.000000e+000 1.000000e+000 0.000000e+000

outer loop

vertex 2.452423e+000 3.000000e+000 6.244478e+000

vertex 3.788410e+000 3.000000e+000 4.860896e+000

vertex 3.701586e+000 3.000000e+000 4.853300e+000

endloop

endfacet

facet normal 0.000000e+000 1.000000e+000 0.000000e+000

outer loop

vertex 3.405388e+000 3.000000e+000 1.449454e+000

vertex 3.355397e+000 3.000000e+000 1.378060e+000

vertex 2.452423e+000 3.000000e+000 2.744478e+000

endloop

endfacet

facet normal 0.000000e+000 1.000000e+000 0.000000e+000

outer loop

vertex 3.617400e+000 3.000000e+000 1.597906e+000

vertex 2.452423e+000 3.000000e+000 2.744478e+000

vertex 3.701586e+000 3.000000e+000 1.620464e+000

endloop

endfacet

facet normal 0.000000e+000 1.000000e+000 0.000000e+000

outer loop

vertex 3.701586e+000 3.000000e+000 1.620464e+000

vertex 2.452423e+000 3.000000e+000 2.744478e+000

vertex 3.788410e+000 3.000000e+000 1.628060e+000

endloop

endfacet

facet normal 0.000000e+000 1.000000e+000 0.000000e+000

outer loop

vertex 3.617400e+000 3.000000e+000 1.597906e+000

vertex 3.538410e+000 3.000000e+000 1.561073e+000

vertex 2.452423e+000 3.000000e+000 2.744478e+000

endloop

endfacet

facet normal 0.000000e+000 1.000000e+000 0.000000e+000

outer loop

vertex 2.452423e+000 3.000000e+000 2.744478e+000

vertex 3.538410e+000 3.000000e+000 1.561073e+000

vertex 3.467016e+000 3.000000e+000 1.511082e+000

endloop

endfacet

facet normal 0.000000e+000 1.000000e+000 0.000000e+000

outer loop

vertex 2.452423e+000 3.000000e+000 2.744478e+000

vertex 3.467016e+000 3.000000e+000 1.511082e+000

vertex 3.405388e+000 3.000000e+000 1.449454e+000

endloop

endfacet

facet normal 0.000000e+000 1.000000e+000 0.000000e+000

outer loop

vertex 3.296006e+000 3.000000e+000 1.041236e+000

vertex 3.318563e+000 3.000000e+000 9.570499e-001

vertex 2.452423e+000 3.000000e+000 3.140303e-001

endloop

endfacet

facet normal 0.000000e+000 1.000000e+000 0.000000e+000

outer loop

vertex 2.452423e+000 3.000000e+000 3.140303e-001

vertex 3.318563e+000 3.000000e+000 9.570499e-001

vertex 3.355397e+000 3.000000e+000 8.780602e-001

endloop

endfacet

facet normal 0.000000e+000 1.000000e+000 0.000000e+000

outer loop

vertex 2.452423e+000 3.000000e+000 3.140303e-001

vertex 3.355397e+000 3.000000e+000 8.780602e-001

vertex 3.405388e+000 3.000000e+000 8.066660e-001

endloop

endfacet

facet normal 0.000000e+000 1.000000e+000 0.000000e+000

outer loop

vertex 3.405388e+000 3.000000e+000 8.066660e-001

vertex 3.467016e+000 3.000000e+000 7.450376e-001

vertex 2.452423e+000 3.000000e+000 3.140303e-001

endloop

endfacet

facet normal 0.000000e+000 1.000000e+000 0.000000e+000

outer loop

vertex 2.452423e+000 3.000000e+000 3.140303e-001

vertex 3.467016e+000 3.000000e+000 7.450376e-001

vertex 3.538410e+000 3.000000e+000 6.950474e-001

endloop

endfacet

facet normal 0.000000e+000 1.000000e+000 0.000000e+000

outer loop

vertex 2.452423e+000 3.000000e+000 3.140303e-001

vertex 3.538410e+000 3.000000e+000 6.950474e-001

vertex 3.617400e+000 3.000000e+000 6.582136e-001

endloop

endfacet

facet normal 0.000000e+000 1.000000e+000 0.000000e+000

outer loop

vertex 5.930367e+000 3.000000e+000 3.140303e-001

vertex 3.959420e+000 3.000000e+000 6.582136e-001

vertex 4.038410e+000 3.000000e+000 6.950474e-001

endloop

endfacet

facet normal 0.000000e+000 1.000000e+000 0.000000e+000

outer loop

vertex 5.930367e+000 3.000000e+000 3.140303e-001

vertex 4.221423e+000 3.000000e+000 8.780602e-001

vertex 4.258256e+000 3.000000e+000 9.570499e-001

endloop

endfacet

facet normal 0.000000e+000 1.000000e+000 0.000000e+000

outer loop

vertex 3.788410e+000 3.000000e+000 3.860896e+000

vertex 5.930367e+000 3.000000e+000 2.744478e+000

vertex 2.452423e+000 3.000000e+000 2.744478e+000

endloop

endfacet

facet normal 0.000000e+000 1.000000e+000 0.000000e+000

outer loop

vertex 2.452423e+000 3.000000e+000 2.744478e+000

vertex 5.930367e+000 3.000000e+000 2.744478e+000

vertex 3.875234e+000 3.000000e+000 1.620464e+000

endloop

endfacet

facet normal 0.000000e+000 1.000000e+000 0.000000e+000

outer loop

vertex 2.452423e+000 3.000000e+000 2.744478e+000

vertex 3.875234e+000 3.000000e+000 1.620464e+000

vertex 3.788410e+000 3.000000e+000 1.628060e+000

endloop

endfacet

facet normal 0.000000e+000 1.000000e+000 0.000000e+000

outer loop

vertex 3.355397e+000 3.000000e+000 1.378060e+000

vertex 3.318563e+000 3.000000e+000 1.299070e+000

vertex 2.452423e+000 3.000000e+000 2.744478e+000

endloop

endfacet

facet normal 0.000000e+000 1.000000e+000 0.000000e+000

outer loop

vertex 2.452423e+000 3.000000e+000 2.744478e+000

vertex 3.318563e+000 3.000000e+000 1.299070e+000

vertex 3.296006e+000 3.000000e+000 1.214884e+000

endloop

endfacet

facet normal 0.000000e+000 1.000000e+000 0.000000e+000

outer loop

vertex 2.452423e+000 3.000000e+000 2.744478e+000

vertex 3.296006e+000 3.000000e+000 1.214884e+000

vertex 2.452423e+000 3.000000e+000 3.140303e-001

endloop

endfacet

facet normal 0.000000e+000 1.000000e+000 0.000000e+000

outer loop

vertex 2.452423e+000 3.000000e+000 3.140303e-001

vertex 3.296006e+000 3.000000e+000 1.214884e+000

vertex 3.288410e+000 3.000000e+000 1.128060e+000

endloop

endfacet

facet normal 0.000000e+000 1.000000e+000 0.000000e+000

outer loop

vertex 2.452423e+000 3.000000e+000 3.140303e-001

vertex 3.288410e+000 3.000000e+000 1.128060e+000

vertex 3.296006e+000 3.000000e+000 1.041236e+000

endloop

endfacet

facet normal 0.000000e+000 1.000000e+000 0.000000e+000

outer loop

vertex 4.038410e+000 3.000000e+000 6.950474e-001

vertex 4.109804e+000 3.000000e+000 7.450376e-001

vertex 5.930367e+000 3.000000e+000 3.140303e-001

endloop

endfacet

facet normal 0.000000e+000 1.000000e+000 0.000000e+000

outer loop

vertex 5.930367e+000 3.000000e+000 3.140303e-001

vertex 4.109804e+000 3.000000e+000 7.450376e-001

vertex 4.171432e+000 3.000000e+000 8.066660e-001

endloop

endfacet

facet normal 0.000000e+000 1.000000e+000 0.000000e+000

outer loop

vertex 5.930367e+000 3.000000e+000 3.140303e-001

vertex 4.171432e+000 3.000000e+000 8.066660e-001

vertex 4.221423e+000 3.000000e+000 8.780602e-001

endloop

endfacet

facet normal 0.000000e+000 1.000000e+000 0.000000e+000

outer loop

vertex 4.258256e+000 3.000000e+000 1.299070e+000

vertex 4.221423e+000 3.000000e+000 1.378060e+000

vertex 5.930367e+000 3.000000e+000 2.744478e+000

endloop

endfacet

facet normal 0.000000e+000 1.000000e+000 0.000000e+000

outer loop

vertex 5.930367e+000 3.000000e+000 2.744478e+000

vertex 4.221423e+000 3.000000e+000 1.378060e+000

vertex 4.171432e+000 3.000000e+000 1.449454e+000

endloop

endfacet

facet normal 0.000000e+000 1.000000e+000 0.000000e+000

outer loop

vertex 5.930367e+000 3.000000e+000 2.744478e+000

vertex 4.171432e+000 3.000000e+000 1.449454e+000

vertex 4.109804e+000 3.000000e+000 1.511082e+000

endloop

endfacet

facet normal 0.000000e+000 1.000000e+000 0.000000e+000

outer loop

vertex 3.617400e+000 3.000000e+000 6.582136e-001

vertex 3.701586e+000 3.000000e+000 6.356561e-001

vertex 2.452423e+000 3.000000e+000 3.140303e-001

endloop

endfacet

facet normal 0.000000e+000 1.000000e+000 0.000000e+000

outer loop

vertex 2.452423e+000 3.000000e+000 3.140303e-001

vertex 3.701586e+000 3.000000e+000 6.356561e-001

vertex 3.788410e+000 3.000000e+000 6.280602e-001

endloop

endfacet

facet normal 0.000000e+000 1.000000e+000 0.000000e+000

outer loop

vertex 2.452423e+000 3.000000e+000 3.140303e-001

vertex 3.788410e+000 3.000000e+000 6.280602e-001

vertex 5.930367e+000 3.000000e+000 3.140303e-001

endloop

endfacet

facet normal 0.000000e+000 1.000000e+000 0.000000e+000

outer loop

vertex 5.930367e+000 3.000000e+000 3.140303e-001

vertex 3.788410e+000 3.000000e+000 6.280602e-001

vertex 3.875234e+000 3.000000e+000 6.356561e-001

endloop

endfacet

facet normal 0.000000e+000 1.000000e+000 0.000000e+000

outer loop

vertex 5.930367e+000 3.000000e+000 3.140303e-001

vertex 3.875234e+000 3.000000e+000 6.356561e-001

vertex 3.959420e+000 3.000000e+000 6.582136e-001

endloop

endfacet

facet normal 0.000000e+000 1.000000e+000 0.000000e+000

outer loop

vertex 4.258256e+000 3.000000e+000 9.570499e-001

vertex 4.280814e+000 3.000000e+000 1.041236e+000

vertex 5.930367e+000 3.000000e+000 3.140303e-001

endloop

endfacet

facet normal 0.000000e+000 1.000000e+000 0.000000e+000

outer loop

vertex 5.930367e+000 3.000000e+000 3.140303e-001

vertex 4.280814e+000 3.000000e+000 1.041236e+000

vertex 4.288410e+000 3.000000e+000 1.128060e+000

endloop

endfacet

facet normal 0.000000e+000 1.000000e+000 0.000000e+000

outer loop

vertex 5.930367e+000 3.000000e+000 3.140303e-001

vertex 4.288410e+000 3.000000e+000 1.128060e+000

vertex 5.930367e+000 3.000000e+000 2.744478e+000

endloop

endfacet

facet normal 0.000000e+000 1.000000e+000 0.000000e+000

outer loop

vertex 5.930367e+000 3.000000e+000 2.744478e+000

vertex 4.288410e+000 3.000000e+000 1.128060e+000

vertex 4.280814e+000 3.000000e+000 1.214884e+000

endloop

endfacet

facet normal 0.000000e+000 1.000000e+000 0.000000e+000

outer loop

vertex 5.930367e+000 3.000000e+000 2.744478e+000

vertex 4.280814e+000 3.000000e+000 1.214884e+000

vertex 4.258256e+000 3.000000e+000 1.299070e+000

endloop

endfacet

facet normal 0.000000e+000 1.000000e+000 0.000000e+000

outer loop

vertex 4.109804e+000 3.000000e+000 1.511082e+000

vertex 4.038410e+000 3.000000e+000 1.561073e+000

vertex 5.930367e+000 3.000000e+000 2.744478e+000

endloop

endfacet

facet normal 0.000000e+000 1.000000e+000 0.000000e+000

outer loop

vertex 5.930367e+000 3.000000e+000 2.744478e+000

vertex 4.038410e+000 3.000000e+000 1.561073e+000

vertex 3.959420e+000 3.000000e+000 1.597906e+000

endloop

endfacet

facet normal 0.000000e+000 1.000000e+000 0.000000e+000

outer loop

vertex 5.930367e+000 3.000000e+000 2.744478e+000

vertex 3.959420e+000 3.000000e+000 1.597906e+000

vertex 3.875234e+000 3.000000e+000 1.620464e+000

endloop

endfacet

facet normal 0.000000e+000 1.000000e+000 0.000000e+000

outer loop

vertex 6.164364e-001 3.000000e+000 1.136090e+001

vertex 6.240327e-001 3.000000e+000 1.127407e+001

vertex 5.943798e-001 3.000000e+000 9.744478e+000

endloop

endfacet

facet normal 0.000000e+000 1.000000e+000 0.000000e+000

outer loop

vertex 5.943798e-001 3.000000e+000 9.744478e+000

vertex 6.240327e-001 3.000000e+000 1.127407e+001

vertex 6.465902e-001 3.000000e+000 1.118989e+001

endloop

endfacet

facet normal 0.000000e+000 1.000000e+000 0.000000e+000

outer loop

vertex 9.454265e-001 3.000000e+000 1.183074e+001

vertex 5.943798e-001 3.000000e+000 1.251332e+001

vertex 1.029613e+000 3.000000e+000 1.185330e+001

endloop

endfacet

facet normal 0.000000e+000 1.000000e+000 0.000000e+000

outer loop

vertex 1.029613e+000 3.000000e+000 1.185330e+001

vertex 5.943798e-001 3.000000e+000 1.251332e+001

vertex 1.116436e+000 3.000000e+000 1.186090e+001

endloop

endfacet

facet normal 0.000000e+000 1.000000e+000 0.000000e+000

outer loop

vertex 6.465902e-001 3.000000e+000 1.118989e+001

vertex 6.834236e-001 3.000000e+000 1.111090e+001

vertex 5.943798e-001 3.000000e+000 9.744478e+000

endloop

endfacet

facet normal 0.000000e+000 1.000000e+000 0.000000e+000

outer loop

vertex 5.943798e-001 3.000000e+000 9.744478e+000

vertex 6.834236e-001 3.000000e+000 1.111090e+001

vertex 7.334142e-001 3.000000e+000 1.103950e+001

endloop

endfacet

facet normal 0.000000e+000 1.000000e+000 0.000000e+000

outer loop

vertex 5.943798e-001 3.000000e+000 9.744478e+000

vertex 7.334142e-001 3.000000e+000 1.103950e+001

vertex 7.950426e-001 3.000000e+000 1.097787e+001

endloop

endfacet

facet normal 0.000000e+000 1.000000e+000 0.000000e+000

outer loop

vertex 1.116436e+000 3.000000e+000 1.186090e+001

vertex 5.943798e-001 3.000000e+000 1.251332e+001

vertex 1.203260e+000 3.000000e+000 1.185330e+001

endloop

endfacet

facet normal 0.000000e+000 1.000000e+000 0.000000e+000

outer loop

vertex 1.203260e+000 3.000000e+000 1.185330e+001

vertex 5.943798e-001 3.000000e+000 1.251332e+001

vertex 2.452423e+000 3.000000e+000 1.251332e+001

endloop

endfacet

facet normal 0.000000e+000 1.000000e+000 0.000000e+000

outer loop

vertex 1.203260e+000 3.000000e+000 1.185330e+001

vertex 2.452423e+000 3.000000e+000 1.251332e+001

vertex 1.287446e+000 3.000000e+000 1.183074e+001

endloop

endfacet

facet normal 0.000000e+000 1.000000e+000 0.000000e+000

outer loop

vertex 9.454265e-001 3.000000e+000 1.183074e+001

vertex 8.664364e-001 3.000000e+000 1.179391e+001

vertex 5.943798e-001 3.000000e+000 1.251332e+001

endloop

endfacet

facet normal 0.000000e+000 1.000000e+000 0.000000e+000

outer loop

vertex 5.943798e-001 3.000000e+000 1.251332e+001

vertex 8.664364e-001 3.000000e+000 1.179391e+001

vertex 7.950426e-001 3.000000e+000 1.174392e+001

endloop

endfacet

facet normal 0.000000e+000 1.000000e+000 0.000000e+000

outer loop

vertex 5.943798e-001 3.000000e+000 1.251332e+001

vertex 7.950426e-001 3.000000e+000 1.174392e+001

vertex 7.334142e-001 3.000000e+000 1.168229e+001

endloop

endfacet

facet normal 0.000000e+000 1.000000e+000 0.000000e+000

outer loop

vertex 1.608840e+000 3.000000e+000 1.144772e+001

vertex 1.586283e+000 3.000000e+000 1.153191e+001

vertex 2.452423e+000 3.000000e+000 1.251332e+001

endloop

endfacet

facet normal 0.000000e+000 1.000000e+000 0.000000e+000

outer loop

vertex 2.452423e+000 3.000000e+000 1.251332e+001

vertex 1.586283e+000 3.000000e+000 1.153191e+001

vertex 1.549449e+000 3.000000e+000 1.161090e+001

endloop

endfacet

facet normal 0.000000e+000 1.000000e+000 0.000000e+000

outer loop

vertex 2.452423e+000 3.000000e+000 1.251332e+001

vertex 1.549449e+000 3.000000e+000 1.161090e+001

vertex 1.499459e+000 3.000000e+000 1.168229e+001

endloop

endfacet

facet normal 0.000000e+000 1.000000e+000 0.000000e+000

outer loop

vertex 1.499459e+000 3.000000e+000 1.168229e+001

vertex 1.437830e+000 3.000000e+000 1.174392e+001

vertex 2.452423e+000 3.000000e+000 1.251332e+001

endloop

endfacet

facet normal 0.000000e+000 1.000000e+000 0.000000e+000

outer loop

vertex 2.452423e+000 3.000000e+000 1.251332e+001

vertex 1.437830e+000 3.000000e+000 1.174392e+001

vertex 1.366436e+000 3.000000e+000 1.179391e+001

endloop

endfacet

facet normal 0.000000e+000 1.000000e+000 0.000000e+000

outer loop

vertex 2.452423e+000 3.000000e+000 1.251332e+001

vertex 1.366436e+000 3.000000e+000 1.179391e+001

vertex 1.287446e+000 3.000000e+000 1.183074e+001

endloop

endfacet

facet normal 0.000000e+000 1.000000e+000 0.000000e+000

outer loop

vertex 7.950426e-001 3.000000e+000 1.097787e+001

vertex 8.664364e-001 3.000000e+000 1.092788e+001

vertex 5.943798e-001 3.000000e+000 9.744478e+000

endloop

endfacet

facet normal 0.000000e+000 1.000000e+000 0.000000e+000

outer loop

vertex 5.943798e-001 3.000000e+000 9.744478e+000

vertex 8.664364e-001 3.000000e+000 1.092788e+001

vertex 9.454265e-001 3.000000e+000 1.089105e+001

endloop

endfacet

facet normal 0.000000e+000 1.000000e+000 0.000000e+000

outer loop

vertex 5.943798e-001 3.000000e+000 9.744478e+000

vertex 9.454265e-001 3.000000e+000 1.089105e+001

vertex 1.029613e+000 3.000000e+000 1.086849e+001

endloop

endfacet

facet normal 0.000000e+000 1.000000e+000 0.000000e+000

outer loop

vertex 1.029613e+000 3.000000e+000 1.086849e+001

vertex 1.116436e+000 3.000000e+000 1.086090e+001

vertex 5.943798e-001 3.000000e+000 9.744478e+000

endloop

endfacet

facet normal 0.000000e+000 1.000000e+000 0.000000e+000

outer loop

vertex 5.943798e-001 3.000000e+000 9.744478e+000

vertex 1.116436e+000 3.000000e+000 1.086090e+001

vertex 2.452423e+000 3.000000e+000 9.744478e+000

endloop

endfacet

facet normal 0.000000e+000 1.000000e+000 0.000000e+000

outer loop

vertex 5.943798e-001 3.000000e+000 9.744478e+000

vertex 2.452423e+000 3.000000e+000 9.744478e+000

vertex 1.243334e+000 3.000000e+000 8.597907e+000

endloop

endfacet

facet normal 0.000000e+000 1.000000e+000 0.000000e+000

outer loop

vertex 1.243334e+000 3.000000e+000 8.597907e+000

vertex 2.452423e+000 3.000000e+000 9.744478e+000

vertex 1.322323e+000 3.000000e+000 8.561073e+000

endloop

endfacet

facet normal 0.000000e+000 1.000000e+000 0.000000e+000

outer loop

vertex 1.116436e+000 3.000000e+000 1.086090e+001

vertex 1.203260e+000 3.000000e+000 1.086849e+001

vertex 2.452423e+000 3.000000e+000 9.744478e+000

endloop

endfacet

facet normal 0.000000e+000 1.000000e+000 0.000000e+000

outer loop

vertex 2.452423e+000 3.000000e+000 9.744478e+000

vertex 1.203260e+000 3.000000e+000 1.086849e+001

vertex 1.287446e+000 3.000000e+000 1.089105e+001

endloop

endfacet

facet normal 0.000000e+000 1.000000e+000 0.000000e+000

outer loop

vertex 2.452423e+000 3.000000e+000 9.744478e+000

vertex 1.287446e+000 3.000000e+000 1.089105e+001

vertex 1.366436e+000 3.000000e+000 1.092788e+001

endloop

endfacet

facet normal 0.000000e+000 1.000000e+000 0.000000e+000

outer loop

vertex 1.366436e+000 3.000000e+000 1.092788e+001

vertex 1.437830e+000 3.000000e+000 1.097787e+001

vertex 2.452423e+000 3.000000e+000 9.744478e+000

endloop

endfacet

facet normal 0.000000e+000 1.000000e+000 0.000000e+000

outer loop

vertex 2.452423e+000 3.000000e+000 9.744478e+000

vertex 1.437830e+000 3.000000e+000 1.097787e+001

vertex 1.499459e+000 3.000000e+000 1.103950e+001

endloop

endfacet

facet normal 0.000000e+000 1.000000e+000 0.000000e+000

outer loop

vertex 2.452423e+000 3.000000e+000 9.744478e+000

vertex 1.499459e+000 3.000000e+000 1.103950e+001

vertex 1.549449e+000 3.000000e+000 1.111090e+001

endloop

endfacet

facet normal 0.000000e+000 1.000000e+000 0.000000e+000

outer loop

vertex 7.334142e-001 3.000000e+000 1.168229e+001

vertex 6.834236e-001 3.000000e+000 1.161090e+001

vertex 5.943798e-001 3.000000e+000 1.251332e+001

endloop

endfacet

facet normal 0.000000e+000 1.000000e+000 0.000000e+000

outer loop

vertex 5.943798e-001 3.000000e+000 1.251332e+001

vertex 6.834236e-001 3.000000e+000 1.161090e+001

vertex 6.465902e-001 3.000000e+000 1.153191e+001

endloop

endfacet

facet normal 0.000000e+000 1.000000e+000 0.000000e+000

outer loop

vertex 5.943798e-001 3.000000e+000 1.251332e+001

vertex 6.465902e-001 3.000000e+000 1.153191e+001

vertex 6.240327e-001 3.000000e+000 1.144772e+001

endloop

endfacet

facet normal 0.000000e+000 1.000000e+000 0.000000e+000

outer loop

vertex 1.549449e+000 3.000000e+000 1.111090e+001

vertex 1.586283e+000 3.000000e+000 1.118989e+001

vertex 2.452423e+000 3.000000e+000 9.744478e+000

endloop

endfacet

facet normal 0.000000e+000 1.000000e+000 0.000000e+000

outer loop

vertex 2.452423e+000 3.000000e+000 9.744478e+000

vertex 1.586283e+000 3.000000e+000 1.118989e+001

vertex 1.608840e+000 3.000000e+000 1.127407e+001

endloop

endfacet

facet normal 0.000000e+000 1.000000e+000 0.000000e+000

outer loop

vertex 2.452423e+000 3.000000e+000 9.744478e+000

vertex 1.608840e+000 3.000000e+000 1.127407e+001

vertex 2.452423e+000 3.000000e+000 1.251332e+001

endloop

endfacet

facet normal 0.000000e+000 1.000000e+000 0.000000e+000

outer loop

vertex 2.452423e+000 3.000000e+000 1.251332e+001

vertex 1.608840e+000 3.000000e+000 1.127407e+001

vertex 1.616436e+000 3.000000e+000 1.136090e+001

endloop

endfacet

facet normal 0.000000e+000 1.000000e+000 0.000000e+000

outer loop

vertex 2.452423e+000 3.000000e+000 1.251332e+001

vertex 1.616436e+000 3.000000e+000 1.136090e+001

vertex 1.608840e+000 3.000000e+000 1.144772e+001

endloop

endfacet

facet normal 0.000000e+000 1.000000e+000 0.000000e+000

outer loop

vertex 1.029613e+000 3.000000e+000 4.853300e+000

vertex 9.454265e-001 3.000000e+000 4.830742e+000

vertex 5.943798e-001 3.000000e+000 6.244478e+000

endloop

endfacet

facet normal 0.000000e+000 1.000000e+000 0.000000e+000

outer loop

vertex 5.943798e-001 3.000000e+000 6.244478e+000

vertex 9.454265e-001 3.000000e+000 4.830742e+000

vertex 8.664364e-001 3.000000e+000 4.793909e+000

endloop

endfacet

facet normal 0.000000e+000 1.000000e+000 0.000000e+000

outer loop

vertex 5.943798e-001 3.000000e+000 6.244478e+000

vertex 8.664364e-001 3.000000e+000 4.793909e+000

vertex 7.950426e-001 3.000000e+000 4.743918e+000

endloop

endfacet

facet normal 0.000000e+000 1.000000e+000 0.000000e+000

outer loop

vertex 2.452423e+000 3.000000e+000 6.244478e+000

vertex 1.586283e+000 3.000000e+000 4.531906e+000

vertex 1.549449e+000 3.000000e+000 4.610896e+000

endloop

endfacet

facet normal 0.000000e+000 1.000000e+000 0.000000e+000

outer loop

vertex 7.950426e-001 3.000000e+000 4.743918e+000

vertex 7.334142e-001 3.000000e+000 4.682290e+000

vertex 5.943798e-001 3.000000e+000 6.244478e+000

endloop

endfacet

facet normal 0.000000e+000 1.000000e+000 0.000000e+000

outer loop

vertex 5.943798e-001 3.000000e+000 6.244478e+000

vertex 7.334142e-001 3.000000e+000 4.682290e+000

vertex 6.834236e-001 3.000000e+000 4.610896e+000

endloop

endfacet

facet normal 0.000000e+000 1.000000e+000 0.000000e+000

outer loop

vertex 5.943798e-001 3.000000e+000 6.244478e+000

vertex 6.834236e-001 3.000000e+000 4.610896e+000

vertex 6.465902e-001 3.000000e+000 4.531906e+000

endloop

endfacet

facet normal 0.000000e+000 1.000000e+000 0.000000e+000

outer loop

vertex 1.549449e+000 3.000000e+000 4.610896e+000

vertex 1.499459e+000 3.000000e+000 4.682290e+000

vertex 2.452423e+000 3.000000e+000 6.244478e+000

endloop

endfacet

facet normal 0.000000e+000 1.000000e+000 0.000000e+000

outer loop

vertex 2.452423e+000 3.000000e+000 6.244478e+000

vertex 1.499459e+000 3.000000e+000 4.682290e+000

vertex 1.437830e+000 3.000000e+000 4.743918e+000

endloop

endfacet

facet normal 0.000000e+000 1.000000e+000 0.000000e+000

outer loop

vertex 2.452423e+000 3.000000e+000 6.244478e+000

vertex 1.437830e+000 3.000000e+000 4.743918e+000

vertex 1.366436e+000 3.000000e+000 4.793909e+000

endloop

endfacet

facet normal 0.000000e+000 1.000000e+000 0.000000e+000

outer loop

vertex 1.366436e+000 3.000000e+000 4.793909e+000

vertex 1.287446e+000 3.000000e+000 4.830742e+000

vertex 2.452423e+000 3.000000e+000 6.244478e+000

endloop

endfacet

facet normal 0.000000e+000 1.000000e+000 0.000000e+000

outer loop

vertex 2.452423e+000 3.000000e+000 6.244478e+000

vertex 1.287446e+000 3.000000e+000 4.830742e+000

vertex 1.203260e+000 3.000000e+000 4.853300e+000

endloop

endfacet

facet normal 0.000000e+000 1.000000e+000 0.000000e+000

outer loop

vertex 2.452423e+000 3.000000e+000 6.244478e+000

vertex 1.203260e+000 3.000000e+000 4.853300e+000

vertex 5.943798e-001 3.000000e+000 6.244478e+000

endloop

endfacet

facet normal 0.000000e+000 1.000000e+000 0.000000e+000

outer loop

vertex 5.943798e-001 3.000000e+000 6.244478e+000

vertex 1.203260e+000 3.000000e+000 4.853300e+000

vertex 1.116436e+000 3.000000e+000 4.860896e+000

endloop

endfacet

facet normal 0.000000e+000 1.000000e+000 0.000000e+000

outer loop

vertex 5.943798e-001 3.000000e+000 6.244478e+000

vertex 1.116436e+000 3.000000e+000 4.860896e+000

vertex 1.029613e+000 3.000000e+000 4.853300e+000

endloop

endfacet

facet normal 0.000000e+000 1.000000e+000 0.000000e+000

outer loop

vertex 1.287446e+000 3.000000e+000 3.891049e+000

vertex 1.366436e+000 3.000000e+000 3.927883e+000

vertex 2.452423e+000 3.000000e+000 2.744478e+000

endloop

endfacet

facet normal 0.000000e+000 1.000000e+000 0.000000e+000

outer loop

vertex 2.452423e+000 3.000000e+000 2.744478e+000

vertex 1.366436e+000 3.000000e+000 3.927883e+000

vertex 1.437830e+000 3.000000e+000 3.977873e+000

endloop

endfacet

facet normal 0.000000e+000 1.000000e+000 0.000000e+000

outer loop

vertex 6.465902e-001 3.000000e+000 4.531906e+000

vertex 6.240327e-001 3.000000e+000 4.447720e+000

vertex 5.943798e-001 3.000000e+000 6.244478e+000

endloop

endfacet

facet normal 0.000000e+000 1.000000e+000 0.000000e+000

outer loop

vertex 5.943798e-001 3.000000e+000 6.244478e+000

vertex 6.240327e-001 3.000000e+000 4.447720e+000

vertex 6.164364e-001 3.000000e+000 4.360896e+000

endloop

endfacet

facet normal 0.000000e+000 1.000000e+000 0.000000e+000

outer loop

vertex 5.943798e-001 3.000000e+000 6.244478e+000

vertex 6.164364e-001 3.000000e+000 4.360896e+000

vertex 5.943798e-001 3.000000e+000 2.744478e+000

endloop

endfacet

facet normal 0.000000e+000 1.000000e+000 0.000000e+000

outer loop

vertex 5.943798e-001 3.000000e+000 2.744478e+000

vertex 6.164364e-001 3.000000e+000 4.360896e+000

vertex 6.240327e-001 3.000000e+000 4.274072e+000

endloop

endfacet

facet normal 0.000000e+000 1.000000e+000 0.000000e+000

outer loop

vertex 6.240327e-001 3.000000e+000 4.274072e+000

vertex 6.465902e-001 3.000000e+000 4.189886e+000

vertex 5.943798e-001 3.000000e+000 2.744478e+000

endloop

endfacet

facet normal 0.000000e+000 1.000000e+000 0.000000e+000

outer loop

vertex 5.943798e-001 3.000000e+000 2.744478e+000

vertex 6.465902e-001 3.000000e+000 4.189886e+000

vertex 6.834236e-001 3.000000e+000 4.110896e+000

endloop

endfacet

facet normal 0.000000e+000 1.000000e+000 0.000000e+000

outer loop

vertex 5.943798e-001 3.000000e+000 2.744478e+000

vertex 6.834236e-001 3.000000e+000 4.110896e+000

vertex 7.334142e-001 3.000000e+000 4.039502e+000

endloop

endfacet

facet normal 0.000000e+000 1.000000e+000 0.000000e+000

outer loop

vertex 7.334142e-001 3.000000e+000 4.039502e+000

vertex 7.950426e-001 3.000000e+000 3.977873e+000

vertex 5.943798e-001 3.000000e+000 2.744478e+000

endloop

endfacet

facet normal 0.000000e+000 1.000000e+000 0.000000e+000

outer loop

vertex 5.943798e-001 3.000000e+000 2.744478e+000

vertex 7.950426e-001 3.000000e+000 3.977873e+000

vertex 8.664364e-001 3.000000e+000 3.927883e+000

endloop

endfacet

facet normal 0.000000e+000 1.000000e+000 0.000000e+000

outer loop

vertex 5.943798e-001 3.000000e+000 2.744478e+000

vertex 8.664364e-001 3.000000e+000 3.927883e+000

vertex 9.454265e-001 3.000000e+000 3.891049e+000

endloop

endfacet

facet normal 0.000000e+000 1.000000e+000 0.000000e+000

outer loop

vertex 9.454265e-001 3.000000e+000 3.891049e+000

vertex 1.029613e+000 3.000000e+000 3.868492e+000

vertex 5.943798e-001 3.000000e+000 2.744478e+000

endloop

endfacet

facet normal 0.000000e+000 1.000000e+000 0.000000e+000

outer loop

vertex 5.943798e-001 3.000000e+000 2.744478e+000

vertex 1.029613e+000 3.000000e+000 3.868492e+000

vertex 1.116436e+000 3.000000e+000 3.860896e+000

endloop

endfacet

facet normal 0.000000e+000 1.000000e+000 0.000000e+000

outer loop

vertex 5.943798e-001 3.000000e+000 2.744478e+000

vertex 1.116436e+000 3.000000e+000 3.860896e+000

vertex 2.452423e+000 3.000000e+000 2.744478e+000

endloop

endfacet

facet normal 0.000000e+000 1.000000e+000 0.000000e+000

outer loop

vertex 2.452423e+000 3.000000e+000 2.744478e+000

vertex 1.116436e+000 3.000000e+000 3.860896e+000

vertex 1.203260e+000 3.000000e+000 3.868492e+000

endloop

endfacet

facet normal 0.000000e+000 1.000000e+000 0.000000e+000

outer loop

vertex 2.452423e+000 3.000000e+000 2.744478e+000

vertex 1.203260e+000 3.000000e+000 3.868492e+000

vertex 1.287446e+000 3.000000e+000 3.891049e+000

endloop

endfacet

facet normal 0.000000e+000 1.000000e+000 0.000000e+000

outer loop

vertex 1.437830e+000 3.000000e+000 3.977873e+000

vertex 1.499459e+000 3.000000e+000 4.039502e+000

vertex 2.452423e+000 3.000000e+000 2.744478e+000

endloop

endfacet

facet normal 0.000000e+000 1.000000e+000 0.000000e+000

outer loop

vertex 2.452423e+000 3.000000e+000 2.744478e+000

vertex 1.499459e+000 3.000000e+000 4.039502e+000

vertex 1.549449e+000 3.000000e+000 4.110896e+000

endloop

endfacet

facet normal 0.000000e+000 1.000000e+000 0.000000e+000

outer loop

vertex 2.452423e+000 3.000000e+000 2.744478e+000

vertex 1.549449e+000 3.000000e+000 4.110896e+000

vertex 1.586283e+000 3.000000e+000 4.189886e+000

endloop

endfacet

facet normal 0.000000e+000 1.000000e+000 0.000000e+000

outer loop

vertex 1.586283e+000 3.000000e+000 4.189886e+000

vertex 1.608840e+000 3.000000e+000 4.274072e+000

vertex 2.452423e+000 3.000000e+000 2.744478e+000

endloop

endfacet

facet normal 0.000000e+000 1.000000e+000 0.000000e+000

outer loop

vertex 2.452423e+000 3.000000e+000 2.744478e+000

vertex 1.608840e+000 3.000000e+000 4.274072e+000

vertex 1.616436e+000 3.000000e+000 4.360896e+000

endloop

endfacet

facet normal 0.000000e+000 1.000000e+000 0.000000e+000

outer loop

vertex 2.452423e+000 3.000000e+000 2.744478e+000

vertex 1.616436e+000 3.000000e+000 4.360896e+000

vertex 2.452423e+000 3.000000e+000 6.244478e+000

endloop

endfacet

facet normal 0.000000e+000 1.000000e+000 0.000000e+000

outer loop

vertex 2.452423e+000 3.000000e+000 6.244478e+000

vertex 1.616436e+000 3.000000e+000 4.360896e+000

vertex 1.608840e+000 3.000000e+000 4.447720e+000

endloop

endfacet

facet normal 0.000000e+000 1.000000e+000 0.000000e+000

outer loop

vertex 2.452423e+000 3.000000e+000 6.244478e+000

vertex 1.608840e+000 3.000000e+000 4.447720e+000

vertex 1.586283e+000 3.000000e+000 4.531906e+000

endloop

endfacet

facet normal 0.000000e+000 1.000000e+000 0.000000e+000

outer loop

vertex 6.240327e-001 3.000000e+000 1.144772e+001

vertex 6.164364e-001 3.000000e+000 1.136090e+001

vertex 5.943798e-001 3.000000e+000 1.251332e+001

endloop

endfacet

facet normal 0.000000e+000 1.000000e+000 0.000000e+000

outer loop

vertex 5.943798e-001 3.000000e+000 1.251332e+001

vertex 6.164364e-001 3.000000e+000 1.136090e+001

vertex 5.943798e-001 3.000000e+000 9.744478e+000

endloop

endfacet

facet normal 0.000000e+000 1.000000e+000 0.000000e+000

outer loop

vertex 5.943798e-001 3.000000e+000 1.251332e+001

vertex 5.943798e-001 3.000000e+000 9.744478e+000

vertex 0.000000e+000 3.000000e+000 1.251332e+001

endloop

endfacet

facet normal 0.000000e+000 1.000000e+000 0.000000e+000

outer loop

vertex 0.000000e+000 3.000000e+000 1.251332e+001

vertex 5.943798e-001 3.000000e+000 9.744478e+000

vertex 0.000000e+000 3.000000e+000 9.744478e+000

endloop

endfacet

facet normal 0.000000e+000 1.000000e+000 0.000000e+000

outer loop

vertex 5.943798e-001 3.000000e+000 1.268851e+000

vertex 0.000000e+000 3.000000e+000 2.744478e+000

vertex 5.943798e-001 3.000000e+000 2.744478e+000

endloop

endfacet

facet normal 0.000000e+000 1.000000e+000 0.000000e+000

outer loop

vertex 5.943798e-001 3.000000e+000 2.744478e+000

vertex 0.000000e+000 3.000000e+000 2.744478e+000

vertex 0.000000e+000 3.000000e+000 6.244478e+000

endloop

endfacet

facet normal 0.000000e+000 1.000000e+000 0.000000e+000

outer loop

vertex 5.943798e-001 3.000000e+000 2.744478e+000

vertex 0.000000e+000 3.000000e+000 6.244478e+000

vertex 5.943798e-001 3.000000e+000 6.244478e+000

endloop

endfacet

facet normal 0.000000e+000 1.000000e+000 0.000000e+000

outer loop

vertex 5.943798e-001 3.000000e+000 6.244478e+000

vertex 0.000000e+000 3.000000e+000 6.244478e+000

vertex 5.943798e-001 3.000000e+000 7.987269e+000

endloop

endfacet

facet normal 0.000000e+000 1.000000e+000 0.000000e+000

outer loop

vertex 5.943798e-001 3.000000e+000 6.244478e+000

vertex 5.943798e-001 3.000000e+000 7.987269e+000

vertex 6.024773e-001 3.000000e+000 7.957050e+000

endloop

endfacet

facet normal 0.000000e+000 1.000000e+000 0.000000e+000

outer loop

vertex 1.191674e+001 3.000000e+000 3.140303e-001

vertex 1.191674e+001 3.000000e+000 0.000000e+000

vertex 9.452423e+000 3.000000e+000 3.140303e-001

endloop

endfacet

facet normal 0.000000e+000 1.000000e+000 0.000000e+000

outer loop

vertex 9.452423e+000 3.000000e+000 3.140303e-001

vertex 1.191674e+001 3.000000e+000 0.000000e+000

vertex 9.452423e+000 3.000000e+000 0.000000e+000

endloop

endfacet

facet normal 0.000000e+000 1.000000e+000 0.000000e+000

outer loop

vertex 9.452423e+000 3.000000e+000 3.140303e-001

vertex 9.452423e+000 3.000000e+000 0.000000e+000

vertex 7.594380e+000 3.000000e+000 3.140303e-001

endloop

endfacet

facet normal 0.000000e+000 1.000000e+000 0.000000e+000

outer loop

vertex 7.594380e+000 3.000000e+000 3.140303e-001

vertex 9.452423e+000 3.000000e+000 0.000000e+000

vertex 7.594380e+000 3.000000e+000 0.000000e+000

endloop

endfacet

facet normal 0.000000e+000 1.000000e+000 0.000000e+000

outer loop

vertex 7.594380e+000 3.000000e+000 3.140303e-001

vertex 7.594380e+000 3.000000e+000 0.000000e+000

vertex 5.930367e+000 3.000000e+000 3.140303e-001

endloop

endfacet

facet normal 0.000000e+000 1.000000e+000 0.000000e+000

outer loop

vertex 5.930367e+000 3.000000e+000 3.140303e-001

vertex 7.594380e+000 3.000000e+000 0.000000e+000

vertex 5.930367e+000 3.000000e+000 0.000000e+000

endloop

endfacet

facet normal 0.000000e+000 1.000000e+000 0.000000e+000

outer loop

vertex 5.930367e+000 3.000000e+000 3.140303e-001

vertex 5.930367e+000 3.000000e+000 0.000000e+000

vertex 2.452423e+000 3.000000e+000 3.140303e-001

endloop

endfacet

facet normal 0.000000e+000 1.000000e+000 0.000000e+000

outer loop

vertex 2.452423e+000 3.000000e+000 3.140303e-001

vertex 5.930367e+000 3.000000e+000 0.000000e+000

vertex 2.452423e+000 3.000000e+000 0.000000e+000

endloop

endfacet

facet normal 0.000000e+000 1.000000e+000 0.000000e+000

outer loop

vertex 2.452423e+000 3.000000e+000 3.140303e-001

vertex 2.452423e+000 3.000000e+000 0.000000e+000

vertex 5.943798e-001 3.000000e+000 3.140303e-001

endloop

endfacet

facet normal 0.000000e+000 1.000000e+000 0.000000e+000

outer loop

vertex 5.943798e-001 3.000000e+000 3.140303e-001

vertex 2.452423e+000 3.000000e+000 0.000000e+000

vertex 5.943798e-001 3.000000e+000 0.000000e+000

endloop

endfacet

facet normal 0.000000e+000 1.000000e+000 0.000000e+000

outer loop

vertex 5.943798e-001 3.000000e+000 3.140303e-001

vertex 5.943798e-001 3.000000e+000 0.000000e+000

vertex 0.000000e+000 3.000000e+000 3.140303e-001

endloop

endfacet

facet normal 0.000000e+000 1.000000e+000 0.000000e+000

outer loop

vertex 0.000000e+000 3.000000e+000 3.140303e-001

vertex 5.943798e-001 3.000000e+000 0.000000e+000

vertex 0.000000e+000 3.000000e+000 0.000000e+000

endloop

endfacet

facet normal 0.000000e+000 -1.000000e+000 0.000000e+000

outer loop

vertex 1.191674e+001 0.000000e+000 0.000000e+000

vertex 1.191674e+001 0.000000e+000 1.251332e+001

vertex 0.000000e+000 0.000000e+000 0.000000e+000

endloop

endfacet

facet normal 0.000000e+000 -1.000000e+000 0.000000e+000

outer loop

vertex 0.000000e+000 0.000000e+000 0.000000e+000

vertex 1.191674e+001 0.000000e+000 1.251332e+001

vertex 0.000000e+000 0.000000e+000 1.251332e+001

endloop

endfacet

facet normal 1.161583e-001 0.000000e+000 9.932307e-001

outer loop

vertex 3.788410e+000 4.400000e+000 1.628060e+000

vertex 3.875234e+000 3.000000e+000 1.620464e+000

vertex 3.875234e+000 4.400000e+000 1.620464e+000

endloop

endfacet

facet normal 2.305519e-001 0.000000e+000 9.730600e-001

outer loop

vertex 3.875234e+000 4.400000e+000 1.620464e+000

vertex 3.875234e+000 3.000000e+000 1.620464e+000

vertex 3.959420e+000 3.000000e+000 1.597906e+000

endloop

endfacet

facet normal 2.868663e-001 0.000000e+000 9.579706e-001

outer loop

vertex 3.875234e+000 4.400000e+000 1.620464e+000

vertex 3.959420e+000 3.000000e+000 1.597906e+000

vertex 3.959420e+000 4.400000e+000 1.597906e+000

endloop

endfacet

facet normal 3.960194e-001 0.000000e+000 9.182422e-001

outer loop

vertex 3.959420e+000 4.400000e+000 1.597906e+000

vertex 3.959420e+000 3.000000e+000 1.597906e+000

vertex 4.038410e+000 3.000000e+000 1.561073e+000

endloop

endfacet

facet normal 4.488580e-001 0.000000e+000 8.936031e-001

outer loop

vertex 3.959420e+000 4.400000e+000 1.597906e+000

vertex 4.038410e+000 3.000000e+000 1.561073e+000

vertex 4.038410e+000 4.400000e+000 1.561073e+000

endloop

endfacet

facet normal 5.494540e-001 0.000000e+000 8.355240e-001

outer loop

vertex 4.038410e+000 4.400000e+000 1.561073e+000

vertex 4.038410e+000 3.000000e+000 1.561073e+000

vertex 4.109804e+000 3.000000e+000 1.511082e+000

endloop

endfacet

facet normal 5.972114e-001 0.000000e+000 8.020838e-001

outer loop

vertex 4.038410e+000 4.400000e+000 1.561073e+000

vertex 4.109804e+000 3.000000e+000 1.511082e+000

vertex 4.109804e+000 4.400000e+000 1.511082e+000

endloop

endfacet

facet normal 6.861938e-001 0.000000e+000 7.274188e-001

outer loop

vertex 4.109804e+000 4.400000e+000 1.511082e+000

vertex 4.109804e+000 3.000000e+000 1.511082e+000

vertex 4.171432e+000 3.000000e+000 1.449454e+000

endloop

endfacet

facet normal 7.274188e-001 0.000000e+000 6.861938e-001

outer loop

vertex 4.109804e+000 4.400000e+000 1.511082e+000

vertex 4.171432e+000 3.000000e+000 1.449454e+000

vertex 4.171432e+000 4.400000e+000 1.449454e+000

endloop

endfacet

facet normal 8.020838e-001 0.000000e+000 5.972114e-001

outer loop

vertex 4.171432e+000 4.400000e+000 1.449454e+000

vertex 4.171432e+000 3.000000e+000 1.449454e+000

vertex 4.221423e+000 3.000000e+000 1.378060e+000

endloop

endfacet

facet normal 8.355240e-001 0.000000e+000 5.494540e-001

outer loop

vertex 4.171432e+000 4.400000e+000 1.449454e+000

vertex 4.221423e+000 3.000000e+000 1.378060e+000

vertex 4.221423e+000 4.400000e+000 1.378060e+000

endloop

endfacet

facet normal 8.936031e-001 0.000000e+000 4.488580e-001

outer loop

vertex 4.221423e+000 4.400000e+000 1.378060e+000

vertex 4.221423e+000 3.000000e+000 1.378060e+000

vertex 4.258256e+000 3.000000e+000 1.299070e+000

endloop

endfacet

facet normal 9.182422e-001 0.000000e+000 3.960194e-001

outer loop

vertex 4.221423e+000 4.400000e+000 1.378060e+000

vertex 4.258256e+000 3.000000e+000 1.299070e+000

vertex 4.258256e+000 4.400000e+000 1.299070e+000

endloop

endfacet

facet normal 9.579706e-001 0.000000e+000 2.868663e-001

outer loop

vertex 4.258256e+000 4.400000e+000 1.299070e+000

vertex 4.258256e+000 3.000000e+000 1.299070e+000

vertex 4.280814e+000 3.000000e+000 1.214884e+000

endloop

endfacet

facet normal 9.730600e-001 0.000000e+000 2.305519e-001

outer loop

vertex 4.258256e+000 4.400000e+000 1.299070e+000

vertex 4.280814e+000 3.000000e+000 1.214884e+000

vertex 4.280814e+000 4.400000e+000 1.214884e+000

endloop

endfacet

facet normal 9.932307e-001 0.000000e+000 1.161583e-001

outer loop

vertex 4.280814e+000 4.400000e+000 1.214884e+000

vertex 4.280814e+000 3.000000e+000 1.214884e+000

vertex 4.288410e+000 3.000000e+000 1.128060e+000

endloop

endfacet

facet normal 9.983119e-001 0.000000e+000 5.807913e-002

outer loop

vertex 4.280814e+000 4.400000e+000 1.214884e+000

vertex 4.288410e+000 3.000000e+000 1.128060e+000

vertex 4.288410e+000 4.400000e+000 1.128060e+000

endloop

endfacet

facet normal 9.983119e-001 0.000000e+000 -5.807913e-002

outer loop

vertex 4.288410e+000 4.400000e+000 1.128060e+000

vertex 4.288410e+000 3.000000e+000 1.128060e+000

vertex 4.280814e+000 3.000000e+000 1.041236e+000

endloop

endfacet

facet normal 9.932307e-001 0.000000e+000 -1.161583e-001

outer loop

vertex 4.288410e+000 4.400000e+000 1.128060e+000

vertex 4.280814e+000 3.000000e+000 1.041236e+000

vertex 4.280814e+000 4.400000e+000 1.041236e+000

endloop

endfacet

facet normal 9.730600e-001 0.000000e+000 -2.305519e-001

outer loop

vertex 4.280814e+000 4.400000e+000 1.041236e+000

vertex 4.280814e+000 3.000000e+000 1.041236e+000

vertex 4.258256e+000 3.000000e+000 9.570499e-001

endloop

endfacet

facet normal 9.579706e-001 0.000000e+000 -2.868663e-001

outer loop

vertex 4.280814e+000 4.400000e+000 1.041236e+000

vertex 4.258256e+000 3.000000e+000 9.570499e-001

vertex 4.258256e+000 4.400000e+000 9.570499e-001

endloop

endfacet

facet normal 9.182422e-001 0.000000e+000 -3.960194e-001

outer loop

vertex 4.258256e+000 4.400000e+000 9.570499e-001

vertex 4.258256e+000 3.000000e+000 9.570499e-001

vertex 4.221423e+000 3.000000e+000 8.780602e-001

endloop

endfacet

facet normal 8.936031e-001 0.000000e+000 -4.488580e-001

outer loop

vertex 4.258256e+000 4.400000e+000 9.570499e-001

vertex 4.221423e+000 3.000000e+000 8.780602e-001

vertex 4.221423e+000 4.400000e+000 8.780602e-001

endloop

endfacet

facet normal 8.355240e-001 0.000000e+000 -5.494540e-001

outer loop

vertex 4.221423e+000 4.400000e+000 8.780602e-001

vertex 4.221423e+000 3.000000e+000 8.780602e-001

vertex 4.171432e+000 3.000000e+000 8.066660e-001

endloop

endfacet

facet normal 8.020838e-001 0.000000e+000 -5.972114e-001

outer loop

vertex 4.221423e+000 4.400000e+000 8.780602e-001

vertex 4.171432e+000 3.000000e+000 8.066660e-001

vertex 4.171432e+000 4.400000e+000 8.066660e-001

endloop

endfacet

facet normal 7.274188e-001 0.000000e+000 -6.861938e-001

outer loop

vertex 4.171432e+000 4.400000e+000 8.066660e-001

vertex 4.171432e+000 3.000000e+000 8.066660e-001

vertex 4.109804e+000 3.000000e+000 7.450376e-001

endloop

endfacet

facet normal 6.861938e-001 0.000000e+000 -7.274188e-001

outer loop

vertex 4.171432e+000 4.400000e+000 8.066660e-001

vertex 4.109804e+000 3.000000e+000 7.450376e-001

vertex 4.109804e+000 4.400000e+000 7.450376e-001

endloop

endfacet

facet normal 5.972114e-001 0.000000e+000 -8.020838e-001

outer loop

vertex 4.109804e+000 4.400000e+000 7.450376e-001

vertex 4.109804e+000 3.000000e+000 7.450376e-001

vertex 4.038410e+000 3.000000e+000 6.950474e-001

endloop

endfacet

facet normal 5.494540e-001 0.000000e+000 -8.355240e-001

outer loop

vertex 4.109804e+000 4.400000e+000 7.450376e-001

vertex 4.038410e+000 3.000000e+000 6.950474e-001

vertex 4.038410e+000 4.400000e+000 6.950474e-001

endloop

endfacet

facet normal 4.488580e-001 0.000000e+000 -8.936031e-001

outer loop

vertex 4.038410e+000 4.400000e+000 6.950474e-001

vertex 4.038410e+000 3.000000e+000 6.950474e-001

vertex 3.959420e+000 3.000000e+000 6.582136e-001

endloop

endfacet

facet normal 3.960194e-001 0.000000e+000 -9.182422e-001

outer loop

vertex 4.038410e+000 4.400000e+000 6.950474e-001

vertex 3.959420e+000 3.000000e+000 6.582136e-001

vertex 3.959420e+000 4.400000e+000 6.582136e-001

endloop

endfacet

facet normal 2.868663e-001 0.000000e+000 -9.579706e-001

outer loop

vertex 3.959420e+000 4.400000e+000 6.582136e-001

vertex 3.959420e+000 3.000000e+000 6.582136e-001

vertex 3.875234e+000 3.000000e+000 6.356561e-001

endloop

endfacet

facet normal 2.305519e-001 0.000000e+000 -9.730600e-001

outer loop

vertex 3.959420e+000 4.400000e+000 6.582136e-001

vertex 3.875234e+000 3.000000e+000 6.356561e-001

vertex 3.875234e+000 4.400000e+000 6.356561e-001

endloop

endfacet

facet normal 1.161583e-001 0.000000e+000 -9.932307e-001

outer loop

vertex 3.875234e+000 4.400000e+000 6.356561e-001

vertex 3.875234e+000 3.000000e+000 6.356561e-001

vertex 3.788410e+000 3.000000e+000 6.280602e-001

endloop

endfacet

facet normal 5.807913e-002 0.000000e+000 -9.983119e-001

outer loop

vertex 3.875234e+000 4.400000e+000 6.356561e-001

vertex 3.788410e+000 3.000000e+000 6.280602e-001

vertex 3.788410e+000 4.400000e+000 6.280602e-001

endloop

endfacet

facet normal -5.807913e-002 0.000000e+000 -9.983119e-001

outer loop

vertex 3.788410e+000 4.400000e+000 6.280602e-001

vertex 3.788410e+000 3.000000e+000 6.280602e-001

vertex 3.701586e+000 3.000000e+000 6.356561e-001

endloop

endfacet

facet normal -1.161583e-001 0.000000e+000 -9.932307e-001

outer loop

vertex 3.788410e+000 4.400000e+000 6.280602e-001

vertex 3.701586e+000 3.000000e+000 6.356561e-001

vertex 3.701586e+000 4.400000e+000 6.356561e-001

endloop

endfacet

facet normal -2.305519e-001 0.000000e+000 -9.730600e-001

outer loop

vertex 3.701586e+000 4.400000e+000 6.356561e-001

vertex 3.701586e+000 3.000000e+000 6.356561e-001

vertex 3.617400e+000 3.000000e+000 6.582136e-001

endloop

endfacet

facet normal -2.868663e-001 0.000000e+000 -9.579706e-001

outer loop

vertex 3.701586e+000 4.400000e+000 6.356561e-001

vertex 3.617400e+000 3.000000e+000 6.582136e-001

vertex 3.617400e+000 4.400000e+000 6.582136e-001

endloop

endfacet

facet normal -3.960194e-001 0.000000e+000 -9.182422e-001

outer loop

vertex 3.617400e+000 4.400000e+000 6.582136e-001

vertex 3.617400e+000 3.000000e+000 6.582136e-001

vertex 3.538410e+000 3.000000e+000 6.950474e-001

endloop

endfacet

facet normal -4.488580e-001 0.000000e+000 -8.936031e-001

outer loop

vertex 3.617400e+000 4.400000e+000 6.582136e-001

vertex 3.538410e+000 3.000000e+000 6.950474e-001

vertex 3.538410e+000 4.400000e+000 6.950474e-001

endloop

endfacet

facet normal -5.494540e-001 0.000000e+000 -8.355240e-001

outer loop

vertex 3.538410e+000 4.400000e+000 6.950474e-001

vertex 3.538410e+000 3.000000e+000 6.950474e-001

vertex 3.467016e+000 3.000000e+000 7.450376e-001

endloop

endfacet

facet normal -5.972114e-001 0.000000e+000 -8.020838e-001

outer loop

vertex 3.538410e+000 4.400000e+000 6.950474e-001

vertex 3.467016e+000 3.000000e+000 7.450376e-001

vertex 3.467016e+000 4.400000e+000 7.450376e-001

endloop

endfacet

facet normal -6.861938e-001 0.000000e+000 -7.274188e-001

outer loop

vertex 3.467016e+000 4.400000e+000 7.450376e-001

vertex 3.467016e+000 3.000000e+000 7.450376e-001

vertex 3.405388e+000 3.000000e+000 8.066660e-001

endloop

endfacet

facet normal -7.274188e-001 0.000000e+000 -6.861938e-001

outer loop

vertex 3.467016e+000 4.400000e+000 7.450376e-001

vertex 3.405388e+000 3.000000e+000 8.066660e-001

vertex 3.405388e+000 4.400000e+000 8.066660e-001

endloop

endfacet

facet normal -8.020838e-001 0.000000e+000 -5.972114e-001

outer loop

vertex 3.405388e+000 4.400000e+000 8.066660e-001

vertex 3.405388e+000 3.000000e+000 8.066660e-001

vertex 3.355397e+000 3.000000e+000 8.780602e-001

endloop

endfacet

facet normal -8.355240e-001 0.000000e+000 -5.494540e-001

outer loop

vertex 3.405388e+000 4.400000e+000 8.066660e-001

vertex 3.355397e+000 3.000000e+000 8.780602e-001

vertex 3.355397e+000 4.400000e+000 8.780602e-001

endloop

endfacet

facet normal -8.936031e-001 0.000000e+000 -4.488580e-001

outer loop

vertex 3.355397e+000 4.400000e+000 8.780602e-001

vertex 3.355397e+000 3.000000e+000 8.780602e-001

vertex 3.318563e+000 3.000000e+000 9.570499e-001

endloop

endfacet

facet normal -9.182422e-001 0.000000e+000 -3.960194e-001

outer loop

vertex 3.355397e+000 4.400000e+000 8.780602e-001

vertex 3.318563e+000 3.000000e+000 9.570499e-001

vertex 3.318563e+000 4.400000e+000 9.570499e-001

endloop

endfacet

facet normal -9.579706e-001 0.000000e+000 -2.868663e-001

outer loop

vertex 3.318563e+000 4.400000e+000 9.570499e-001

vertex 3.318563e+000 3.000000e+000 9.570499e-001

vertex 3.296006e+000 3.000000e+000 1.041236e+000

endloop

endfacet

facet normal -9.730600e-001 0.000000e+000 -2.305519e-001

outer loop

vertex 3.318563e+000 4.400000e+000 9.570499e-001

vertex 3.296006e+000 3.000000e+000 1.041236e+000

vertex 3.296006e+000 4.400000e+000 1.041236e+000

endloop

endfacet

facet normal -9.932307e-001 0.000000e+000 -1.161583e-001

outer loop

vertex 3.296006e+000 4.400000e+000 1.041236e+000

vertex 3.296006e+000 3.000000e+000 1.041236e+000

vertex 3.288410e+000 3.000000e+000 1.128060e+000

endloop

endfacet

facet normal -9.983119e-001 0.000000e+000 -5.807913e-002

outer loop

vertex 3.296006e+000 4.400000e+000 1.041236e+000

vertex 3.288410e+000 3.000000e+000 1.128060e+000

vertex 3.288410e+000 4.400000e+000 1.128060e+000

endloop

endfacet

facet normal -9.983119e-001 0.000000e+000 5.807913e-002

outer loop

vertex 3.288410e+000 4.400000e+000 1.128060e+000

vertex 3.288410e+000 3.000000e+000 1.128060e+000

vertex 3.296006e+000 3.000000e+000 1.214884e+000

endloop

endfacet

facet normal -9.932307e-001 0.000000e+000 1.161583e-001

outer loop

vertex 3.288410e+000 4.400000e+000 1.128060e+000

vertex 3.296006e+000 3.000000e+000 1.214884e+000

vertex 3.296006e+000 4.400000e+000 1.214884e+000

endloop

endfacet

facet normal -9.730600e-001 0.000000e+000 2.305519e-001

outer loop

vertex 3.296006e+000 4.400000e+000 1.214884e+000

vertex 3.296006e+000 3.000000e+000 1.214884e+000

vertex 3.318563e+000 3.000000e+000 1.299070e+000

endloop

endfacet

facet normal -9.579706e-001 0.000000e+000 2.868663e-001

outer loop

vertex 3.296006e+000 4.400000e+000 1.214884e+000

vertex 3.318563e+000 3.000000e+000 1.299070e+000

vertex 3.318563e+000 4.400000e+000 1.299070e+000

endloop

endfacet

facet normal -9.182422e-001 0.000000e+000 3.960194e-001

outer loop

vertex 3.318563e+000 4.400000e+000 1.299070e+000

vertex 3.318563e+000 3.000000e+000 1.299070e+000

vertex 3.355397e+000 3.000000e+000 1.378060e+000

endloop

endfacet

facet normal -8.936031e-001 0.000000e+000 4.488580e-001

outer loop

vertex 3.318563e+000 4.400000e+000 1.299070e+000

vertex 3.355397e+000 3.000000e+000 1.378060e+000

vertex 3.355397e+000 4.400000e+000 1.378060e+000

endloop

endfacet

facet normal -8.355240e-001 0.000000e+000 5.494540e-001

outer loop

vertex 3.355397e+000 4.400000e+000 1.378060e+000

vertex 3.355397e+000 3.000000e+000 1.378060e+000

vertex 3.405388e+000 3.000000e+000 1.449454e+000

endloop

endfacet

facet normal -8.020838e-001 0.000000e+000 5.972114e-001

outer loop

vertex 3.355397e+000 4.400000e+000 1.378060e+000

vertex 3.405388e+000 3.000000e+000 1.449454e+000

vertex 3.405388e+000 4.400000e+000 1.449454e+000

endloop

endfacet

facet normal -7.274188e-001 0.000000e+000 6.861938e-001

outer loop

vertex 3.405388e+000 4.400000e+000 1.449454e+000

vertex 3.405388e+000 3.000000e+000 1.449454e+000

vertex 3.467016e+000 3.000000e+000 1.511082e+000

endloop

endfacet

facet normal -6.861938e-001 0.000000e+000 7.274188e-001

outer loop

vertex 3.405388e+000 4.400000e+000 1.449454e+000

vertex 3.467016e+000 3.000000e+000 1.511082e+000

vertex 3.467016e+000 4.400000e+000 1.511082e+000

endloop

endfacet

facet normal -5.972114e-001 0.000000e+000 8.020838e-001

outer loop

vertex 3.467016e+000 4.400000e+000 1.511082e+000

vertex 3.467016e+000 3.000000e+000 1.511082e+000

vertex 3.538410e+000 3.000000e+000 1.561073e+000

endloop

endfacet

facet normal -5.494540e-001 0.000000e+000 8.355240e-001

outer loop

vertex 3.467016e+000 4.400000e+000 1.511082e+000

vertex 3.538410e+000 3.000000e+000 1.561073e+000

vertex 3.538410e+000 4.400000e+000 1.561073e+000

endloop

endfacet

facet normal -4.488580e-001 0.000000e+000 8.936031e-001

outer loop

vertex 3.538410e+000 4.400000e+000 1.561073e+000

vertex 3.538410e+000 3.000000e+000 1.561073e+000

vertex 3.617400e+000 3.000000e+000 1.597906e+000

endloop

endfacet

facet normal -3.960194e-001 0.000000e+000 9.182422e-001

outer loop

vertex 3.538410e+000 4.400000e+000 1.561073e+000

vertex 3.617400e+000 3.000000e+000 1.597906e+000

vertex 3.617400e+000 4.400000e+000 1.597906e+000

endloop

endfacet

facet normal -2.868663e-001 0.000000e+000 9.579706e-001

outer loop

vertex 3.617400e+000 4.400000e+000 1.597906e+000

vertex 3.617400e+000 3.000000e+000 1.597906e+000

vertex 3.701586e+000 3.000000e+000 1.620464e+000

endloop

endfacet

facet normal -2.305519e-001 0.000000e+000 9.730600e-001

outer loop

vertex 3.617400e+000 4.400000e+000 1.597906e+000

vertex 3.701586e+000 3.000000e+000 1.620464e+000

vertex 3.701586e+000 4.400000e+000 1.620464e+000

endloop

endfacet

facet normal -1.161583e-001 0.000000e+000 9.932307e-001

outer loop

vertex 3.701586e+000 4.400000e+000 1.620464e+000

vertex 3.701586e+000 3.000000e+000 1.620464e+000

vertex 3.788410e+000 3.000000e+000 1.628060e+000

endloop

endfacet

facet normal -5.807913e-002 0.000000e+000 9.983119e-001

outer loop

vertex 3.701586e+000 4.400000e+000 1.620464e+000

vertex 3.788410e+000 3.000000e+000 1.628060e+000

vertex 3.788410e+000 4.400000e+000 1.628060e+000

endloop

endfacet

facet normal 5.807913e-002 0.000000e+000 9.983119e-001

outer loop

vertex 3.788410e+000 4.400000e+000 1.628060e+000

vertex 3.788410e+000 3.000000e+000 1.628060e+000

vertex 3.875234e+000 3.000000e+000 1.620464e+000

endloop

endfacet

facet normal -9.983119e-001 0.000000e+000 -5.807913e-002

outer loop

vertex 5.799197e-001 4.400000e+000 1.041236e+000

vertex 5.723238e-001 3.000000e+000 1.128060e+000

vertex 5.723238e-001 4.400000e+000 1.128060e+000

endloop

endfacet

facet normal -9.983119e-001 0.000000e+000 5.807913e-002

outer loop

vertex 5.723238e-001 4.400000e+000 1.128060e+000

vertex 5.723238e-001 3.000000e+000 1.128060e+000

vertex 5.799197e-001 3.000000e+000 1.214884e+000

endloop

endfacet

facet normal -9.932307e-001 0.000000e+000 1.161583e-001

outer loop

vertex 5.723238e-001 4.400000e+000 1.128060e+000

vertex 5.799197e-001 3.000000e+000 1.214884e+000

vertex 5.799197e-001 4.400000e+000 1.214884e+000

endloop

endfacet

facet normal -9.776480e-001 0.000000e+000 2.102483e-001

outer loop

vertex 5.799197e-001 4.400000e+000 1.214884e+000

vertex 5.799197e-001 3.000000e+000 1.214884e+000

vertex 5.943798e-001 3.000000e+000 1.268851e+000

endloop

endfacet

facet normal -9.637589e-001 0.000000e+000 2.667744e-001

outer loop

vertex 5.799197e-001 4.400000e+000 1.214884e+000

vertex 5.943798e-001 3.000000e+000 1.268851e+000

vertex 6.024773e-001 4.400000e+000 1.299070e+000

endloop

endfacet

facet normal -9.466226e-001 0.000000e+000 3.223439e-001

outer loop

vertex 5.943798e-001 3.000000e+000 1.268851e+000

vertex 6.024773e-001 3.000000e+000 1.299070e+000

vertex 6.024773e-001 4.400000e+000 1.299070e+000

endloop

endfacet

facet normal -9.182422e-001 0.000000e+000 3.960194e-001

outer loop

vertex 6.024773e-001 4.400000e+000 1.299070e+000

vertex 6.024773e-001 3.000000e+000 1.299070e+000

vertex 6.393110e-001 3.000000e+000 1.378060e+000

endloop

endfacet

facet normal -8.936031e-001 0.000000e+000 4.488580e-001

outer loop

vertex 6.024773e-001 4.400000e+000 1.299070e+000

vertex 6.393110e-001 3.000000e+000 1.378060e+000

vertex 6.393110e-001 4.400000e+000 1.378060e+000

endloop

endfacet

facet normal -8.355240e-001 0.000000e+000 5.494540e-001

outer loop

vertex 6.393110e-001 4.400000e+000 1.378060e+000

vertex 6.393110e-001 3.000000e+000 1.378060e+000

vertex 6.893016e-001 3.000000e+000 1.449454e+000

endloop

endfacet

facet normal -8.020838e-001 0.000000e+000 5.972114e-001

outer loop

vertex 6.393110e-001 4.400000e+000 1.378060e+000

vertex 6.893016e-001 3.000000e+000 1.449454e+000

vertex 6.893016e-001 4.400000e+000 1.449454e+000

endloop

endfacet

facet normal -7.274188e-001 0.000000e+000 6.861938e-001

outer loop

vertex 6.893016e-001 4.400000e+000 1.449454e+000

vertex 6.893016e-001 3.000000e+000 1.449454e+000

vertex 7.509301e-001 3.000000e+000 1.511082e+000

endloop

endfacet

facet normal -6.861938e-001 0.000000e+000 7.274188e-001

outer loop

vertex 6.893016e-001 4.400000e+000 1.449454e+000

vertex 7.509301e-001 3.000000e+000 1.511082e+000

vertex 7.509301e-001 4.400000e+000 1.511082e+000

endloop

endfacet

facet normal -5.972114e-001 0.000000e+000 8.020838e-001

outer loop

vertex 7.509301e-001 4.400000e+000 1.511082e+000

vertex 7.509301e-001 3.000000e+000 1.511082e+000

vertex 8.223239e-001 3.000000e+000 1.561073e+000

endloop

endfacet

facet normal -5.494540e-001 0.000000e+000 8.355240e-001

outer loop

vertex 7.509301e-001 4.400000e+000 1.511082e+000

vertex 8.223239e-001 3.000000e+000 1.561073e+000

vertex 8.223239e-001 4.400000e+000 1.561073e+000

endloop

endfacet

facet normal -4.488580e-001 0.000000e+000 8.936031e-001

outer loop

vertex 8.223239e-001 4.400000e+000 1.561073e+000

vertex 8.223239e-001 3.000000e+000 1.561073e+000

vertex 9.013135e-001 3.000000e+000 1.597906e+000

endloop

endfacet

facet normal -3.960194e-001 0.000000e+000 9.182422e-001

outer loop

vertex 8.223239e-001 4.400000e+000 1.561073e+000

vertex 9.013135e-001 3.000000e+000 1.597906e+000

vertex 9.013135e-001 4.400000e+000 1.597906e+000

endloop

endfacet

facet normal -2.868663e-001 0.000000e+000 9.579706e-001

outer loop

vertex 9.013135e-001 4.400000e+000 1.597906e+000

vertex 9.013135e-001 3.000000e+000 1.597906e+000

vertex 9.854995e-001 3.000000e+000 1.620464e+000

endloop

endfacet

facet normal -2.305519e-001 0.000000e+000 9.730600e-001

outer loop

vertex 9.013135e-001 4.400000e+000 1.597906e+000

vertex 9.854995e-001 3.000000e+000 1.620464e+000

vertex 9.854995e-001 4.400000e+000 1.620464e+000

endloop

endfacet

facet normal -1.161583e-001 0.000000e+000 9.932307e-001

outer loop

vertex 9.854995e-001 4.400000e+000 1.620464e+000

vertex 9.854995e-001 3.000000e+000 1.620464e+000

vertex 1.072323e+000 3.000000e+000 1.628060e+000

endloop

endfacet

facet normal -5.807913e-002 0.000000e+000 9.983119e-001

outer loop

vertex 9.854995e-001 4.400000e+000 1.620464e+000

vertex 1.072323e+000 3.000000e+000 1.628060e+000

vertex 1.072323e+000 4.400000e+000 1.628060e+000

endloop

endfacet

facet normal 5.807913e-002 0.000000e+000 9.983119e-001

outer loop

vertex 1.072323e+000 4.400000e+000 1.628060e+000

vertex 1.072323e+000 3.000000e+000 1.628060e+000

vertex 1.159148e+000 3.000000e+000 1.620464e+000

endloop

endfacet

facet normal 1.161583e-001 0.000000e+000 9.932307e-001

outer loop

vertex 1.072323e+000 4.400000e+000 1.628060e+000

vertex 1.159148e+000 3.000000e+000 1.620464e+000

vertex 1.159148e+000 4.400000e+000 1.620464e+000

endloop

endfacet

facet normal 2.305519e-001 0.000000e+000 9.730600e-001

outer loop

vertex 1.159148e+000 4.400000e+000 1.620464e+000

vertex 1.159148e+000 3.000000e+000 1.620464e+000

vertex 1.243334e+000 3.000000e+000 1.597906e+000

endloop

endfacet

facet normal 2.868663e-001 0.000000e+000 9.579706e-001

outer loop

vertex 1.159148e+000 4.400000e+000 1.620464e+000

vertex 1.243334e+000 3.000000e+000 1.597906e+000

vertex 1.243334e+000 4.400000e+000 1.597906e+000

endloop

endfacet

facet normal 3.960194e-001 0.000000e+000 9.182422e-001

outer loop

vertex 1.243334e+000 4.400000e+000 1.597906e+000

vertex 1.243334e+000 3.000000e+000 1.597906e+000

vertex 1.322323e+000 3.000000e+000 1.561073e+000

endloop

endfacet

facet normal 4.488580e-001 0.000000e+000 8.936031e-001

outer loop

vertex 1.243334e+000 4.400000e+000 1.597906e+000

vertex 1.322323e+000 3.000000e+000 1.561073e+000

vertex 1.322323e+000 4.400000e+000 1.561073e+000

endloop

endfacet

facet normal 5.494540e-001 0.000000e+000 8.355240e-001

outer loop

vertex 1.322323e+000 4.400000e+000 1.561073e+000

vertex 1.322323e+000 3.000000e+000 1.561073e+000

vertex 1.393717e+000 3.000000e+000 1.511082e+000

endloop

endfacet

facet normal 5.972114e-001 0.000000e+000 8.020838e-001

outer loop

vertex 1.322323e+000 4.400000e+000 1.561073e+000

vertex 1.393717e+000 3.000000e+000 1.511082e+000

vertex 1.393717e+000 4.400000e+000 1.511082e+000

endloop

endfacet

facet normal 6.861938e-001 0.000000e+000 7.274188e-001

outer loop

vertex 1.393717e+000 4.400000e+000 1.511082e+000

vertex 1.393717e+000 3.000000e+000 1.511082e+000

vertex 1.455346e+000 3.000000e+000 1.449454e+000

endloop

endfacet

facet normal 7.274188e-001 0.000000e+000 6.861938e-001

outer loop

vertex 1.393717e+000 4.400000e+000 1.511082e+000

vertex 1.455346e+000 3.000000e+000 1.449454e+000

vertex 1.455346e+000 4.400000e+000 1.449454e+000

endloop

endfacet

facet normal 8.020838e-001 0.000000e+000 5.972114e-001

outer loop

vertex 1.455346e+000 4.400000e+000 1.449454e+000

vertex 1.455346e+000 3.000000e+000 1.449454e+000

vertex 1.505336e+000 3.000000e+000 1.378060e+000

endloop

endfacet

facet normal 8.355240e-001 0.000000e+000 5.494540e-001

outer loop

vertex 1.455346e+000 4.400000e+000 1.449454e+000

vertex 1.505336e+000 3.000000e+000 1.378060e+000

vertex 1.505336e+000 4.400000e+000 1.378060e+000

endloop

endfacet

facet normal 8.936031e-001 0.000000e+000 4.488580e-001

outer loop

vertex 1.505336e+000 4.400000e+000 1.378060e+000

vertex 1.505336e+000 3.000000e+000 1.378060e+000

vertex 1.542170e+000 3.000000e+000 1.299070e+000

endloop

endfacet

facet normal 9.182422e-001 0.000000e+000 3.960194e-001

outer loop

vertex 1.505336e+000 4.400000e+000 1.378060e+000

vertex 1.542170e+000 3.000000e+000 1.299070e+000

vertex 1.542170e+000 4.400000e+000 1.299070e+000

endloop

endfacet

facet normal 9.579706e-001 0.000000e+000 2.868663e-001

outer loop

vertex 1.542170e+000 4.400000e+000 1.299070e+000

vertex 1.542170e+000 3.000000e+000 1.299070e+000

vertex 1.564728e+000 3.000000e+000 1.214884e+000

endloop

endfacet

facet normal 9.730600e-001 0.000000e+000 2.305519e-001

outer loop

vertex 1.542170e+000 4.400000e+000 1.299070e+000

vertex 1.564728e+000 3.000000e+000 1.214884e+000

vertex 1.564728e+000 4.400000e+000 1.214884e+000

endloop

endfacet

facet normal 9.932307e-001 0.000000e+000 1.161583e-001

outer loop

vertex 1.564728e+000 4.400000e+000 1.214884e+000

vertex 1.564728e+000 3.000000e+000 1.214884e+000

vertex 1.572323e+000 3.000000e+000 1.128060e+000

endloop

endfacet

facet normal 9.983119e-001 0.000000e+000 5.807913e-002

outer loop

vertex 1.564728e+000 4.400000e+000 1.214884e+000

vertex 1.572323e+000 3.000000e+000 1.128060e+000

vertex 1.572323e+000 4.400000e+000 1.128060e+000

endloop

endfacet

facet normal 9.983119e-001 0.000000e+000 -5.807913e-002

outer loop

vertex 1.572323e+000 4.400000e+000 1.128060e+000

vertex 1.572323e+000 3.000000e+000 1.128060e+000

vertex 1.564728e+000 3.000000e+000 1.041236e+000

endloop

endfacet

facet normal 9.932307e-001 0.000000e+000 -1.161583e-001

outer loop

vertex 1.572323e+000 4.400000e+000 1.128060e+000

vertex 1.564728e+000 3.000000e+000 1.041236e+000

vertex 1.564728e+000 4.400000e+000 1.041236e+000

endloop

endfacet

facet normal 9.730600e-001 0.000000e+000 -2.305519e-001

outer loop

vertex 1.564728e+000 4.400000e+000 1.041236e+000

vertex 1.564728e+000 3.000000e+000 1.041236e+000

vertex 1.542170e+000 3.000000e+000 9.570499e-001

endloop

endfacet

facet normal 9.579706e-001 0.000000e+000 -2.868663e-001

outer loop

vertex 1.564728e+000 4.400000e+000 1.041236e+000

vertex 1.542170e+000 3.000000e+000 9.570499e-001

vertex 1.542170e+000 4.400000e+000 9.570499e-001

endloop

endfacet

facet normal 9.182422e-001 0.000000e+000 -3.960194e-001

outer loop

vertex 1.542170e+000 4.400000e+000 9.570499e-001

vertex 1.542170e+000 3.000000e+000 9.570499e-001

vertex 1.505336e+000 3.000000e+000 8.780602e-001

endloop

endfacet

facet normal 8.936031e-001 0.000000e+000 -4.488580e-001

outer loop

vertex 1.542170e+000 4.400000e+000 9.570499e-001

vertex 1.505336e+000 3.000000e+000 8.780602e-001

vertex 1.505336e+000 4.400000e+000 8.780602e-001

endloop

endfacet

facet normal 8.355240e-001 0.000000e+000 -5.494540e-001

outer loop

vertex 1.505336e+000 4.400000e+000 8.780602e-001

vertex 1.505336e+000 3.000000e+000 8.780602e-001

vertex 1.455346e+000 3.000000e+000 8.066660e-001

endloop

endfacet

facet normal 8.020838e-001 0.000000e+000 -5.972114e-001

outer loop

vertex 1.505336e+000 4.400000e+000 8.780602e-001

vertex 1.455346e+000 3.000000e+000 8.066660e-001

vertex 1.455346e+000 4.400000e+000 8.066660e-001

endloop

endfacet

facet normal 7.274188e-001 0.000000e+000 -6.861938e-001

outer loop

vertex 1.455346e+000 4.400000e+000 8.066660e-001

vertex 1.455346e+000 3.000000e+000 8.066660e-001

vertex 1.393717e+000 3.000000e+000 7.450376e-001

endloop

endfacet

facet normal 6.861938e-001 0.000000e+000 -7.274188e-001

outer loop

vertex 1.455346e+000 4.400000e+000 8.066660e-001

vertex 1.393717e+000 3.000000e+000 7.450376e-001

vertex 1.393717e+000 4.400000e+000 7.450376e-001

endloop

endfacet

facet normal 5.972114e-001 0.000000e+000 -8.020838e-001

outer loop

vertex 1.393717e+000 4.400000e+000 7.450376e-001

vertex 1.393717e+000 3.000000e+000 7.450376e-001

vertex 1.322323e+000 3.000000e+000 6.950474e-001

endloop

endfacet

facet normal 5.494540e-001 0.000000e+000 -8.355240e-001

outer loop

vertex 1.393717e+000 4.400000e+000 7.450376e-001

vertex 1.322323e+000 3.000000e+000 6.950474e-001

vertex 1.322323e+000 4.400000e+000 6.950474e-001

endloop

endfacet

facet normal 4.488580e-001 0.000000e+000 -8.936031e-001

outer loop

vertex 1.322323e+000 4.400000e+000 6.950474e-001

vertex 1.322323e+000 3.000000e+000 6.950474e-001

vertex 1.243334e+000 3.000000e+000 6.582136e-001

endloop

endfacet

facet normal 3.960194e-001 0.000000e+000 -9.182422e-001

outer loop

vertex 1.322323e+000 4.400000e+000 6.950474e-001

vertex 1.243334e+000 3.000000e+000 6.582136e-001

vertex 1.243334e+000 4.400000e+000 6.582136e-001

endloop

endfacet

facet normal 2.868663e-001 0.000000e+000 -9.579706e-001

outer loop

vertex 1.243334e+000 4.400000e+000 6.582136e-001

vertex 1.243334e+000 3.000000e+000 6.582136e-001

vertex 1.159148e+000 3.000000e+000 6.356561e-001

endloop

endfacet

facet normal 2.305519e-001 0.000000e+000 -9.730600e-001

outer loop

vertex 1.243334e+000 4.400000e+000 6.582136e-001

vertex 1.159148e+000 3.000000e+000 6.356561e-001

vertex 1.159148e+000 4.400000e+000 6.356561e-001

endloop

endfacet

facet normal 1.161583e-001 0.000000e+000 -9.932307e-001

outer loop

vertex 1.159148e+000 4.400000e+000 6.356561e-001

vertex 1.159148e+000 3.000000e+000 6.356561e-001

vertex 1.072323e+000 3.000000e+000 6.280602e-001

endloop

endfacet

facet normal 5.807913e-002 0.000000e+000 -9.983119e-001

outer loop

vertex 1.159148e+000 4.400000e+000 6.356561e-001

vertex 1.072323e+000 3.000000e+000 6.280602e-001

vertex 1.072323e+000 4.400000e+000 6.280602e-001

endloop

endfacet

facet normal -5.807913e-002 0.000000e+000 -9.983119e-001

outer loop

vertex 1.072323e+000 4.400000e+000 6.280602e-001

vertex 1.072323e+000 3.000000e+000 6.280602e-001

vertex 9.854995e-001 3.000000e+000 6.356561e-001

endloop

endfacet

facet normal -1.161583e-001 0.000000e+000 -9.932307e-001

outer loop

vertex 1.072323e+000 4.400000e+000 6.280602e-001

vertex 9.854995e-001 3.000000e+000 6.356561e-001

vertex 9.854995e-001 4.400000e+000 6.356561e-001

endloop

endfacet

facet normal -2.305519e-001 0.000000e+000 -9.730600e-001

outer loop

vertex 9.854995e-001 4.400000e+000 6.356561e-001

vertex 9.854995e-001 3.000000e+000 6.356561e-001

vertex 9.013135e-001 3.000000e+000 6.582136e-001

endloop

endfacet

facet normal -2.868663e-001 0.000000e+000 -9.579706e-001

outer loop

vertex 9.854995e-001 4.400000e+000 6.356561e-001

vertex 9.013135e-001 3.000000e+000 6.582136e-001

vertex 9.013135e-001 4.400000e+000 6.582136e-001

endloop

endfacet

facet normal -3.960194e-001 0.000000e+000 -9.182422e-001

outer loop

vertex 9.013135e-001 4.400000e+000 6.582136e-001

vertex 9.013135e-001 3.000000e+000 6.582136e-001

vertex 8.223239e-001 3.000000e+000 6.950474e-001

endloop

endfacet

facet normal -4.488580e-001 0.000000e+000 -8.936031e-001

outer loop

vertex 9.013135e-001 4.400000e+000 6.582136e-001

vertex 8.223239e-001 3.000000e+000 6.950474e-001

vertex 8.223239e-001 4.400000e+000 6.950474e-001

endloop

endfacet

facet normal -5.494540e-001 0.000000e+000 -8.355240e-001

outer loop

vertex 8.223239e-001 4.400000e+000 6.950474e-001

vertex 8.223239e-001 3.000000e+000 6.950474e-001

vertex 7.509301e-001 3.000000e+000 7.450376e-001

endloop

endfacet

facet normal -5.972114e-001 0.000000e+000 -8.020838e-001

outer loop

vertex 8.223239e-001 4.400000e+000 6.950474e-001

vertex 7.509301e-001 3.000000e+000 7.450376e-001

vertex 7.509301e-001 4.400000e+000 7.450376e-001

endloop

endfacet

facet normal -6.861938e-001 0.000000e+000 -7.274188e-001

outer loop

vertex 7.509301e-001 4.400000e+000 7.450376e-001

vertex 7.509301e-001 3.000000e+000 7.450376e-001

vertex 6.893016e-001 3.000000e+000 8.066660e-001

endloop

endfacet

facet normal -7.274188e-001 0.000000e+000 -6.861938e-001

outer loop

vertex 7.509301e-001 4.400000e+000 7.450376e-001

vertex 6.893016e-001 3.000000e+000 8.066660e-001

vertex 6.893016e-001 4.400000e+000 8.066660e-001

endloop

endfacet

facet normal -8.020838e-001 0.000000e+000 -5.972114e-001

outer loop

vertex 6.893016e-001 4.400000e+000 8.066660e-001

vertex 6.893016e-001 3.000000e+000 8.066660e-001

vertex 6.393110e-001 3.000000e+000 8.780602e-001

endloop

endfacet

facet normal -8.355240e-001 0.000000e+000 -5.494540e-001

outer loop

vertex 6.893016e-001 4.400000e+000 8.066660e-001

vertex 6.393110e-001 3.000000e+000 8.780602e-001

vertex 6.393110e-001 4.400000e+000 8.780602e-001

endloop

endfacet

facet normal -8.936031e-001 0.000000e+000 -4.488580e-001

outer loop

vertex 6.393110e-001 4.400000e+000 8.780602e-001

vertex 6.393110e-001 3.000000e+000 8.780602e-001

vertex 6.024773e-001 3.000000e+000 9.570499e-001

endloop

endfacet

facet normal -9.182422e-001 0.000000e+000 -3.960194e-001

outer loop

vertex 6.393110e-001 4.400000e+000 8.780602e-001

vertex 6.024773e-001 3.000000e+000 9.570499e-001

vertex 6.024773e-001 4.400000e+000 9.570499e-001

endloop

endfacet

facet normal -9.466226e-001 0.000000e+000 -3.223439e-001

outer loop

vertex 6.024773e-001 4.400000e+000 9.570499e-001

vertex 6.024773e-001 3.000000e+000 9.570499e-001

vertex 5.943798e-001 3.000000e+000 9.872695e-001

endloop

endfacet

facet normal -9.637589e-001 0.000000e+000 -2.667744e-001

outer loop

vertex 6.024773e-001 4.400000e+000 9.570499e-001

vertex 5.943798e-001 3.000000e+000 9.872695e-001

vertex 5.799197e-001 4.400000e+000 1.041236e+000

endloop

endfacet

facet normal -9.776480e-001 0.000000e+000 -2.102483e-001

outer loop

vertex 5.799197e-001 4.400000e+000 1.041236e+000

vertex 5.943798e-001 3.000000e+000 9.872695e-001

vertex 5.799197e-001 3.000000e+000 1.041236e+000

endloop

endfacet

facet normal -9.932307e-001 0.000000e+000 -1.161583e-001

outer loop

vertex 5.799197e-001 4.400000e+000 1.041236e+000

vertex 5.799197e-001 3.000000e+000 1.041236e+000

vertex 5.723238e-001 3.000000e+000 1.128060e+000

endloop

endfacet

facet normal 1.161583e-001 0.000000e+000 9.932307e-001

outer loop

vertex 3.788410e+000 4.400000e+000 4.860896e+000

vertex 3.875234e+000 3.000000e+000 4.853300e+000

vertex 3.875234e+000 4.400000e+000 4.853300e+000

endloop

endfacet

facet normal 2.305519e-001 0.000000e+000 9.730600e-001

outer loop

vertex 3.875234e+000 4.400000e+000 4.853300e+000

vertex 3.875234e+000 3.000000e+000 4.853300e+000

vertex 3.959420e+000 3.000000e+000 4.830742e+000

endloop

endfacet

facet normal 2.868663e-001 0.000000e+000 9.579706e-001

outer loop

vertex 3.875234e+000 4.400000e+000 4.853300e+000

vertex 3.959420e+000 3.000000e+000 4.830742e+000

vertex 3.959420e+000 4.400000e+000 4.830742e+000

endloop

endfacet

facet normal 3.960194e-001 0.000000e+000 9.182422e-001

outer loop

vertex 3.959420e+000 4.400000e+000 4.830742e+000

vertex 3.959420e+000 3.000000e+000 4.830742e+000

vertex 4.038410e+000 3.000000e+000 4.793909e+000

endloop

endfacet

facet normal 4.488580e-001 0.000000e+000 8.936031e-001

outer loop

vertex 3.959420e+000 4.400000e+000 4.830742e+000

vertex 4.038410e+000 3.000000e+000 4.793909e+000

vertex 4.038410e+000 4.400000e+000 4.793909e+000

endloop

endfacet

facet normal 5.494540e-001 0.000000e+000 8.355240e-001

outer loop

vertex 4.038410e+000 4.400000e+000 4.793909e+000

vertex 4.038410e+000 3.000000e+000 4.793909e+000

vertex 4.109804e+000 3.000000e+000 4.743918e+000

endloop

endfacet

facet normal 5.972114e-001 0.000000e+000 8.020838e-001

outer loop

vertex 4.038410e+000 4.400000e+000 4.793909e+000

vertex 4.109804e+000 3.000000e+000 4.743918e+000

vertex 4.109804e+000 4.400000e+000 4.743918e+000

endloop

endfacet

facet normal 6.861938e-001 0.000000e+000 7.274188e-001

outer loop

vertex 4.109804e+000 4.400000e+000 4.743918e+000

vertex 4.109804e+000 3.000000e+000 4.743918e+000

vertex 4.171432e+000 3.000000e+000 4.682290e+000

endloop

endfacet

facet normal 7.274188e-001 0.000000e+000 6.861938e-001

outer loop

vertex 4.109804e+000 4.400000e+000 4.743918e+000

vertex 4.171432e+000 3.000000e+000 4.682290e+000

vertex 4.171432e+000 4.400000e+000 4.682290e+000

endloop

endfacet

facet normal 8.020838e-001 0.000000e+000 5.972114e-001

outer loop

vertex 4.171432e+000 4.400000e+000 4.682290e+000

vertex 4.171432e+000 3.000000e+000 4.682290e+000

vertex 4.221423e+000 3.000000e+000 4.610896e+000

endloop

endfacet

facet normal 8.355240e-001 0.000000e+000 5.494540e-001

outer loop

vertex 4.171432e+000 4.400000e+000 4.682290e+000

vertex 4.221423e+000 3.000000e+000 4.610896e+000

vertex 4.221423e+000 4.400000e+000 4.610896e+000

endloop

endfacet

facet normal 8.936031e-001 0.000000e+000 4.488580e-001

outer loop

vertex 4.221423e+000 4.400000e+000 4.610896e+000

vertex 4.221423e+000 3.000000e+000 4.610896e+000

vertex 4.258256e+000 3.000000e+000 4.531906e+000

endloop

endfacet

facet normal 9.182422e-001 0.000000e+000 3.960194e-001

outer loop

vertex 4.221423e+000 4.400000e+000 4.610896e+000

vertex 4.258256e+000 3.000000e+000 4.531906e+000

vertex 4.258256e+000 4.400000e+000 4.531906e+000

endloop

endfacet

facet normal 9.579706e-001 0.000000e+000 2.868663e-001

outer loop

vertex 4.258256e+000 4.400000e+000 4.531906e+000

vertex 4.258256e+000 3.000000e+000 4.531906e+000

vertex 4.280814e+000 3.000000e+000 4.447720e+000

endloop

endfacet

facet normal 9.730600e-001 0.000000e+000 2.305519e-001

outer loop

vertex 4.258256e+000 4.400000e+000 4.531906e+000

vertex 4.280814e+000 3.000000e+000 4.447720e+000

vertex 4.280814e+000 4.400000e+000 4.447720e+000

endloop

endfacet

facet normal 9.932307e-001 0.000000e+000 1.161583e-001

outer loop

vertex 4.280814e+000 4.400000e+000 4.447720e+000

vertex 4.280814e+000 3.000000e+000 4.447720e+000

vertex 4.288410e+000 3.000000e+000 4.360896e+000

endloop

endfacet

facet normal 9.983119e-001 0.000000e+000 5.807913e-002

outer loop

vertex 4.280814e+000 4.400000e+000 4.447720e+000

vertex 4.288410e+000 3.000000e+000 4.360896e+000

vertex 4.288410e+000 4.400000e+000 4.360896e+000

endloop

endfacet

facet normal 9.983119e-001 0.000000e+000 -5.807913e-002

outer loop

vertex 4.288410e+000 4.400000e+000 4.360896e+000

vertex 4.288410e+000 3.000000e+000 4.360896e+000

vertex 4.280814e+000 3.000000e+000 4.274072e+000

endloop

endfacet

facet normal 9.932307e-001 0.000000e+000 -1.161583e-001

outer loop

vertex 4.288410e+000 4.400000e+000 4.360896e+000

vertex 4.280814e+000 3.000000e+000 4.274072e+000

vertex 4.280814e+000 4.400000e+000 4.274072e+000

endloop

endfacet

facet normal 9.730600e-001 0.000000e+000 -2.305519e-001

outer loop

vertex 4.280814e+000 4.400000e+000 4.274072e+000

vertex 4.280814e+000 3.000000e+000 4.274072e+000

vertex 4.258256e+000 3.000000e+000 4.189886e+000

endloop

endfacet

facet normal 9.579706e-001 0.000000e+000 -2.868663e-001

outer loop

vertex 4.280814e+000 4.400000e+000 4.274072e+000

vertex 4.258256e+000 3.000000e+000 4.189886e+000

vertex 4.258256e+000 4.400000e+000 4.189886e+000

endloop

endfacet

facet normal 9.182422e-001 0.000000e+000 -3.960194e-001

outer loop

vertex 4.258256e+000 4.400000e+000 4.189886e+000

vertex 4.258256e+000 3.000000e+000 4.189886e+000

vertex 4.221423e+000 3.000000e+000 4.110896e+000

endloop

endfacet

facet normal 8.936031e-001 0.000000e+000 -4.488580e-001

outer loop

vertex 4.258256e+000 4.400000e+000 4.189886e+000

vertex 4.221423e+000 3.000000e+000 4.110896e+000

vertex 4.221423e+000 4.400000e+000 4.110896e+000

endloop

endfacet

facet normal 8.355240e-001 0.000000e+000 -5.494540e-001

outer loop

vertex 4.221423e+000 4.400000e+000 4.110896e+000

vertex 4.221423e+000 3.000000e+000 4.110896e+000

vertex 4.171432e+000 3.000000e+000 4.039502e+000

endloop

endfacet

facet normal 8.020838e-001 0.000000e+000 -5.972114e-001

outer loop

vertex 4.221423e+000 4.400000e+000 4.110896e+000

vertex 4.171432e+000 3.000000e+000 4.039502e+000

vertex 4.171432e+000 4.400000e+000 4.039502e+000

endloop

endfacet

facet normal 7.274188e-001 0.000000e+000 -6.861938e-001

outer loop

vertex 4.171432e+000 4.400000e+000 4.039502e+000

vertex 4.171432e+000 3.000000e+000 4.039502e+000

vertex 4.109804e+000 3.000000e+000 3.977873e+000

endloop

endfacet

facet normal 6.861938e-001 0.000000e+000 -7.274188e-001

outer loop

vertex 4.171432e+000 4.400000e+000 4.039502e+000

vertex 4.109804e+000 3.000000e+000 3.977873e+000

vertex 4.109804e+000 4.400000e+000 3.977873e+000

endloop

endfacet

facet normal 5.972114e-001 0.000000e+000 -8.020838e-001

outer loop

vertex 4.109804e+000 4.400000e+000 3.977873e+000

vertex 4.109804e+000 3.000000e+000 3.977873e+000

vertex 4.038410e+000 3.000000e+000 3.927883e+000

endloop

endfacet

facet normal 5.494540e-001 0.000000e+000 -8.355240e-001

outer loop

vertex 4.109804e+000 4.400000e+000 3.977873e+000

vertex 4.038410e+000 3.000000e+000 3.927883e+000

vertex 4.038410e+000 4.400000e+000 3.927883e+000

endloop

endfacet

facet normal 4.488580e-001 0.000000e+000 -8.936031e-001

outer loop

vertex 4.038410e+000 4.400000e+000 3.927883e+000

vertex 4.038410e+000 3.000000e+000 3.927883e+000

vertex 3.959420e+000 3.000000e+000 3.891049e+000

endloop

endfacet

facet normal 3.960194e-001 0.000000e+000 -9.182422e-001

outer loop

vertex 4.038410e+000 4.400000e+000 3.927883e+000

vertex 3.959420e+000 3.000000e+000 3.891049e+000

vertex 3.959420e+000 4.400000e+000 3.891049e+000

endloop

endfacet

facet normal 2.868663e-001 0.000000e+000 -9.579706e-001

outer loop

vertex 3.959420e+000 4.400000e+000 3.891049e+000

vertex 3.959420e+000 3.000000e+000 3.891049e+000

vertex 3.875234e+000 3.000000e+000 3.868492e+000

endloop

endfacet

facet normal 2.305519e-001 0.000000e+000 -9.730600e-001

outer loop

vertex 3.959420e+000 4.400000e+000 3.891049e+000

vertex 3.875234e+000 3.000000e+000 3.868492e+000

vertex 3.875234e+000 4.400000e+000 3.868492e+000

endloop

endfacet

facet normal 1.161583e-001 0.000000e+000 -9.932307e-001

outer loop

vertex 3.875234e+000 4.400000e+000 3.868492e+000

vertex 3.875234e+000 3.000000e+000 3.868492e+000

vertex 3.788410e+000 3.000000e+000 3.860896e+000

endloop

endfacet

facet normal 5.807913e-002 0.000000e+000 -9.983119e-001

outer loop

vertex 3.875234e+000 4.400000e+000 3.868492e+000

vertex 3.788410e+000 3.000000e+000 3.860896e+000

vertex 3.788410e+000 4.400000e+000 3.860896e+000

endloop

endfacet

facet normal -5.807913e-002 0.000000e+000 -9.983119e-001

outer loop

vertex 3.788410e+000 4.400000e+000 3.860896e+000

vertex 3.788410e+000 3.000000e+000 3.860896e+000

vertex 3.701586e+000 3.000000e+000 3.868492e+000

endloop

endfacet

facet normal -1.161583e-001 0.000000e+000 -9.932307e-001

outer loop

vertex 3.788410e+000 4.400000e+000 3.860896e+000

vertex 3.701586e+000 3.000000e+000 3.868492e+000

vertex 3.701586e+000 4.400000e+000 3.868492e+000

endloop

endfacet

facet normal -2.305519e-001 0.000000e+000 -9.730600e-001

outer loop

vertex 3.701586e+000 4.400000e+000 3.868492e+000

vertex 3.701586e+000 3.000000e+000 3.868492e+000

vertex 3.617400e+000 3.000000e+000 3.891049e+000

endloop

endfacet

facet normal -2.868663e-001 0.000000e+000 -9.579706e-001

outer loop

vertex 3.701586e+000 4.400000e+000 3.868492e+000

vertex 3.617400e+000 3.000000e+000 3.891049e+000

vertex 3.617400e+000 4.400000e+000 3.891049e+000

endloop

endfacet

facet normal -3.960194e-001 0.000000e+000 -9.182422e-001

outer loop

vertex 3.617400e+000 4.400000e+000 3.891049e+000

vertex 3.617400e+000 3.000000e+000 3.891049e+000

vertex 3.538410e+000 3.000000e+000 3.927883e+000

endloop

endfacet

facet normal -4.488580e-001 0.000000e+000 -8.936031e-001

outer loop

vertex 3.617400e+000 4.400000e+000 3.891049e+000

vertex 3.538410e+000 3.000000e+000 3.927883e+000

vertex 3.538410e+000 4.400000e+000 3.927883e+000

endloop

endfacet

facet normal -5.494540e-001 0.000000e+000 -8.355240e-001

outer loop

vertex 3.538410e+000 4.400000e+000 3.927883e+000

vertex 3.538410e+000 3.000000e+000 3.927883e+000

vertex 3.467016e+000 3.000000e+000 3.977873e+000

endloop

endfacet

facet normal -5.972114e-001 0.000000e+000 -8.020838e-001

outer loop

vertex 3.538410e+000 4.400000e+000 3.927883e+000

vertex 3.467016e+000 3.000000e+000 3.977873e+000

vertex 3.467016e+000 4.400000e+000 3.977873e+000

endloop

endfacet

facet normal -6.861938e-001 0.000000e+000 -7.274188e-001

outer loop

vertex 3.467016e+000 4.400000e+000 3.977873e+000

vertex 3.467016e+000 3.000000e+000 3.977873e+000

vertex 3.405388e+000 3.000000e+000 4.039502e+000

endloop

endfacet

facet normal -7.274188e-001 0.000000e+000 -6.861938e-001

outer loop

vertex 3.467016e+000 4.400000e+000 3.977873e+000

vertex 3.405388e+000 3.000000e+000 4.039502e+000

vertex 3.405388e+000 4.400000e+000 4.039502e+000

endloop

endfacet

facet normal -8.020838e-001 0.000000e+000 -5.972114e-001

outer loop

vertex 3.405388e+000 4.400000e+000 4.039502e+000

vertex 3.405388e+000 3.000000e+000 4.039502e+000

vertex 3.355397e+000 3.000000e+000 4.110896e+000

endloop

endfacet

facet normal -8.355240e-001 0.000000e+000 -5.494540e-001

outer loop

vertex 3.405388e+000 4.400000e+000 4.039502e+000

vertex 3.355397e+000 3.000000e+000 4.110896e+000

vertex 3.355397e+000 4.400000e+000 4.110896e+000

endloop

endfacet

facet normal -8.936031e-001 0.000000e+000 -4.488580e-001

outer loop

vertex 3.355397e+000 4.400000e+000 4.110896e+000

vertex 3.355397e+000 3.000000e+000 4.110896e+000

vertex 3.318563e+000 3.000000e+000 4.189886e+000

endloop

endfacet

facet normal -9.182422e-001 0.000000e+000 -3.960194e-001

outer loop

vertex 3.355397e+000 4.400000e+000 4.110896e+000

vertex 3.318563e+000 3.000000e+000 4.189886e+000

vertex 3.318563e+000 4.400000e+000 4.189886e+000

endloop

endfacet

facet normal -9.579706e-001 0.000000e+000 -2.868663e-001

outer loop

vertex 3.318563e+000 4.400000e+000 4.189886e+000

vertex 3.318563e+000 3.000000e+000 4.189886e+000

vertex 3.296006e+000 3.000000e+000 4.274072e+000

endloop

endfacet

facet normal -9.730600e-001 0.000000e+000 -2.305519e-001

outer loop

vertex 3.318563e+000 4.400000e+000 4.189886e+000

vertex 3.296006e+000 3.000000e+000 4.274072e+000

vertex 3.296006e+000 4.400000e+000 4.274072e+000

endloop

endfacet

facet normal -9.932307e-001 0.000000e+000 -1.161583e-001

outer loop

vertex 3.296006e+000 4.400000e+000 4.274072e+000

vertex 3.296006e+000 3.000000e+000 4.274072e+000

vertex 3.288410e+000 3.000000e+000 4.360896e+000

endloop

endfacet

facet normal -9.983119e-001 0.000000e+000 -5.807913e-002

outer loop

vertex 3.296006e+000 4.400000e+000 4.274072e+000

vertex 3.288410e+000 3.000000e+000 4.360896e+000

vertex 3.288410e+000 4.400000e+000 4.360896e+000

endloop

endfacet

facet normal -9.983119e-001 0.000000e+000 5.807913e-002

outer loop

vertex 3.288410e+000 4.400000e+000 4.360896e+000

vertex 3.288410e+000 3.000000e+000 4.360896e+000

vertex 3.296006e+000 3.000000e+000 4.447720e+000

endloop

endfacet

facet normal -9.932307e-001 0.000000e+000 1.161583e-001

outer loop

vertex 3.288410e+000 4.400000e+000 4.360896e+000

vertex 3.296006e+000 3.000000e+000 4.447720e+000

vertex 3.296006e+000 4.400000e+000 4.447720e+000

endloop

endfacet

facet normal -9.730600e-001 0.000000e+000 2.305519e-001

outer loop

vertex 3.296006e+000 4.400000e+000 4.447720e+000

vertex 3.296006e+000 3.000000e+000 4.447720e+000

vertex 3.318563e+000 3.000000e+000 4.531906e+000

endloop

endfacet

facet normal -9.579706e-001 0.000000e+000 2.868663e-001

outer loop

vertex 3.296006e+000 4.400000e+000 4.447720e+000

vertex 3.318563e+000 3.000000e+000 4.531906e+000

vertex 3.318563e+000 4.400000e+000 4.531906e+000

endloop

endfacet

facet normal -9.182422e-001 0.000000e+000 3.960194e-001

outer loop

vertex 3.318563e+000 4.400000e+000 4.531906e+000

vertex 3.318563e+000 3.000000e+000 4.531906e+000

vertex 3.355397e+000 3.000000e+000 4.610896e+000

endloop

endfacet

facet normal -8.936031e-001 0.000000e+000 4.488580e-001

outer loop

vertex 3.318563e+000 4.400000e+000 4.531906e+000

vertex 3.355397e+000 3.000000e+000 4.610896e+000

vertex 3.355397e+000 4.400000e+000 4.610896e+000

endloop

endfacet

facet normal -8.355240e-001 0.000000e+000 5.494540e-001

outer loop

vertex 3.355397e+000 4.400000e+000 4.610896e+000

vertex 3.355397e+000 3.000000e+000 4.610896e+000

vertex 3.405388e+000 3.000000e+000 4.682290e+000

endloop

endfacet

facet normal -8.020838e-001 0.000000e+000 5.972114e-001

outer loop

vertex 3.355397e+000 4.400000e+000 4.610896e+000

vertex 3.405388e+000 3.000000e+000 4.682290e+000

vertex 3.405388e+000 4.400000e+000 4.682290e+000

endloop

endfacet

facet normal -7.274188e-001 0.000000e+000 6.861938e-001

outer loop

vertex 3.405388e+000 4.400000e+000 4.682290e+000

vertex 3.405388e+000 3.000000e+000 4.682290e+000

vertex 3.467016e+000 3.000000e+000 4.743918e+000

endloop

endfacet

facet normal -6.861938e-001 0.000000e+000 7.274188e-001

outer loop

vertex 3.405388e+000 4.400000e+000 4.682290e+000

vertex 3.467016e+000 3.000000e+000 4.743918e+000

vertex 3.467016e+000 4.400000e+000 4.743918e+000

endloop

endfacet

facet normal -5.972114e-001 0.000000e+000 8.020838e-001

outer loop

vertex 3.467016e+000 4.400000e+000 4.743918e+000

vertex 3.467016e+000 3.000000e+000 4.743918e+000

vertex 3.538410e+000 3.000000e+000 4.793909e+000

endloop

endfacet

facet normal -5.494540e-001 0.000000e+000 8.355240e-001

outer loop

vertex 3.467016e+000 4.400000e+000 4.743918e+000

vertex 3.538410e+000 3.000000e+000 4.793909e+000

vertex 3.538410e+000 4.400000e+000 4.793909e+000

endloop

endfacet

facet normal -4.488580e-001 0.000000e+000 8.936031e-001

outer loop

vertex 3.538410e+000 4.400000e+000 4.793909e+000

vertex 3.538410e+000 3.000000e+000 4.793909e+000

vertex 3.617400e+000 3.000000e+000 4.830742e+000

endloop

endfacet

facet normal -3.960194e-001 0.000000e+000 9.182422e-001

outer loop

vertex 3.538410e+000 4.400000e+000 4.793909e+000

vertex 3.617400e+000 3.000000e+000 4.830742e+000

vertex 3.617400e+000 4.400000e+000 4.830742e+000

endloop

endfacet

facet normal -2.868663e-001 0.000000e+000 9.579706e-001

outer loop

vertex 3.617400e+000 4.400000e+000 4.830742e+000

vertex 3.617400e+000 3.000000e+000 4.830742e+000

vertex 3.701586e+000 3.000000e+000 4.853300e+000

endloop

endfacet

facet normal -2.305519e-001 0.000000e+000 9.730600e-001

outer loop

vertex 3.617400e+000 4.400000e+000 4.830742e+000

vertex 3.701586e+000 3.000000e+000 4.853300e+000

vertex 3.701586e+000 4.400000e+000 4.853300e+000

endloop

endfacet

facet normal -1.161583e-001 0.000000e+000 9.932307e-001

outer loop

vertex 3.701586e+000 4.400000e+000 4.853300e+000

vertex 3.701586e+000 3.000000e+000 4.853300e+000

vertex 3.788410e+000 3.000000e+000 4.860896e+000

endloop

endfacet

facet normal -5.807913e-002 0.000000e+000 9.983119e-001

outer loop

vertex 3.701586e+000 4.400000e+000 4.853300e+000

vertex 3.788410e+000 3.000000e+000 4.860896e+000

vertex 3.788410e+000 4.400000e+000 4.860896e+000

endloop

endfacet

facet normal 5.807913e-002 0.000000e+000 9.983119e-001

outer loop

vertex 3.788410e+000 4.400000e+000 4.860896e+000

vertex 3.788410e+000 3.000000e+000 4.860896e+000

vertex 3.875234e+000 3.000000e+000 4.853300e+000

endloop

endfacet

facet normal 1.161583e-001 0.000000e+000 9.932307e-001

outer loop

vertex 1.116436e+000 4.400000e+000 4.860896e+000

vertex 1.203260e+000 3.000000e+000 4.853300e+000

vertex 1.203260e+000 4.400000e+000 4.853300e+000

endloop

endfacet

facet normal 2.305519e-001 0.000000e+000 9.730600e-001

outer loop

vertex 1.203260e+000 4.400000e+000 4.853300e+000

vertex 1.203260e+000 3.000000e+000 4.853300e+000

vertex 1.287446e+000 3.000000e+000 4.830742e+000

endloop

endfacet

facet normal 2.868663e-001 0.000000e+000 9.579706e-001

outer loop

vertex 1.203260e+000 4.400000e+000 4.853300e+000

vertex 1.287446e+000 3.000000e+000 4.830742e+000

vertex 1.287446e+000 4.400000e+000 4.830742e+000

endloop

endfacet

facet normal 3.960194e-001 0.000000e+000 9.182422e-001

outer loop

vertex 1.287446e+000 4.400000e+000 4.830742e+000

vertex 1.287446e+000 3.000000e+000 4.830742e+000

vertex 1.366436e+000 3.000000e+000 4.793909e+000

endloop

endfacet

facet normal 4.488580e-001 0.000000e+000 8.936031e-001

outer loop

vertex 1.287446e+000 4.400000e+000 4.830742e+000

vertex 1.366436e+000 3.000000e+000 4.793909e+000

vertex 1.366436e+000 4.400000e+000 4.793909e+000

endloop

endfacet

facet normal 5.494540e-001 0.000000e+000 8.355240e-001

outer loop

vertex 1.366436e+000 4.400000e+000 4.793909e+000

vertex 1.366436e+000 3.000000e+000 4.793909e+000

vertex 1.437830e+000 3.000000e+000 4.743918e+000

endloop

endfacet

facet normal 5.972114e-001 0.000000e+000 8.020838e-001

outer loop

vertex 1.366436e+000 4.400000e+000 4.793909e+000

vertex 1.437830e+000 3.000000e+000 4.743918e+000

vertex 1.437830e+000 4.400000e+000 4.743918e+000

endloop

endfacet

facet normal 6.861938e-001 0.000000e+000 7.274188e-001

outer loop

vertex 1.437830e+000 4.400000e+000 4.743918e+000

vertex 1.437830e+000 3.000000e+000 4.743918e+000

vertex 1.499459e+000 3.000000e+000 4.682290e+000

endloop

endfacet

facet normal 7.274188e-001 0.000000e+000 6.861938e-001

outer loop

vertex 1.437830e+000 4.400000e+000 4.743918e+000

vertex 1.499459e+000 3.000000e+000 4.682290e+000

vertex 1.499459e+000 4.400000e+000 4.682290e+000

endloop

endfacet

facet normal 8.020838e-001 0.000000e+000 5.972114e-001

outer loop

vertex 1.499459e+000 4.400000e+000 4.682290e+000

vertex 1.499459e+000 3.000000e+000 4.682290e+000

vertex 1.549449e+000 3.000000e+000 4.610896e+000

endloop

endfacet

facet normal 8.355240e-001 0.000000e+000 5.494540e-001

outer loop

vertex 1.499459e+000 4.400000e+000 4.682290e+000

vertex 1.549449e+000 3.000000e+000 4.610896e+000

vertex 1.549449e+000 4.400000e+000 4.610896e+000

endloop

endfacet

facet normal 8.936031e-001 0.000000e+000 4.488580e-001

outer loop

vertex 1.549449e+000 4.400000e+000 4.610896e+000

vertex 1.549449e+000 3.000000e+000 4.610896e+000

vertex 1.586283e+000 3.000000e+000 4.531906e+000

endloop

endfacet

facet normal 9.182422e-001 0.000000e+000 3.960194e-001

outer loop

vertex 1.549449e+000 4.400000e+000 4.610896e+000

vertex 1.586283e+000 3.000000e+000 4.531906e+000

vertex 1.586283e+000 4.400000e+000 4.531906e+000

endloop

endfacet

facet normal 9.579706e-001 0.000000e+000 2.868663e-001

outer loop

vertex 1.586283e+000 4.400000e+000 4.531906e+000

vertex 1.586283e+000 3.000000e+000 4.531906e+000

vertex 1.608840e+000 3.000000e+000 4.447720e+000

endloop

endfacet

facet normal 9.730600e-001 0.000000e+000 2.305519e-001

outer loop

vertex 1.586283e+000 4.400000e+000 4.531906e+000

vertex 1.608840e+000 3.000000e+000 4.447720e+000

vertex 1.608840e+000 4.400000e+000 4.447720e+000

endloop

endfacet

facet normal 9.932307e-001 0.000000e+000 1.161583e-001

outer loop

vertex 1.608840e+000 4.400000e+000 4.447720e+000

vertex 1.608840e+000 3.000000e+000 4.447720e+000

vertex 1.616436e+000 3.000000e+000 4.360896e+000

endloop

endfacet

facet normal 9.983119e-001 0.000000e+000 5.807913e-002

outer loop

vertex 1.608840e+000 4.400000e+000 4.447720e+000

vertex 1.616436e+000 3.000000e+000 4.360896e+000

vertex 1.616436e+000 4.400000e+000 4.360896e+000

endloop

endfacet

facet normal 9.983119e-001 0.000000e+000 -5.807913e-002

outer loop

vertex 1.616436e+000 4.400000e+000 4.360896e+000

vertex 1.616436e+000 3.000000e+000 4.360896e+000

vertex 1.608840e+000 3.000000e+000 4.274072e+000

endloop

endfacet

facet normal 9.932307e-001 0.000000e+000 -1.161583e-001

outer loop

vertex 1.616436e+000 4.400000e+000 4.360896e+000

vertex 1.608840e+000 3.000000e+000 4.274072e+000

vertex 1.608840e+000 4.400000e+000 4.274072e+000

endloop

endfacet

facet normal 9.730600e-001 0.000000e+000 -2.305519e-001

outer loop

vertex 1.608840e+000 4.400000e+000 4.274072e+000

vertex 1.608840e+000 3.000000e+000 4.274072e+000

vertex 1.586283e+000 3.000000e+000 4.189886e+000

endloop

endfacet

facet normal 9.579706e-001 0.000000e+000 -2.868663e-001

outer loop

vertex 1.608840e+000 4.400000e+000 4.274072e+000

vertex 1.586283e+000 3.000000e+000 4.189886e+000

vertex 1.586283e+000 4.400000e+000 4.189886e+000

endloop

endfacet

facet normal 9.182422e-001 0.000000e+000 -3.960194e-001

outer loop

vertex 1.586283e+000 4.400000e+000 4.189886e+000

vertex 1.586283e+000 3.000000e+000 4.189886e+000

vertex 1.549449e+000 3.000000e+000 4.110896e+000

endloop

endfacet

facet normal 8.936031e-001 0.000000e+000 -4.488580e-001

outer loop

vertex 1.586283e+000 4.400000e+000 4.189886e+000

vertex 1.549449e+000 3.000000e+000 4.110896e+000

vertex 1.549449e+000 4.400000e+000 4.110896e+000

endloop

endfacet

facet normal 8.355240e-001 0.000000e+000 -5.494540e-001

outer loop

vertex 1.549449e+000 4.400000e+000 4.110896e+000

vertex 1.549449e+000 3.000000e+000 4.110896e+000

vertex 1.499459e+000 3.000000e+000 4.039502e+000

endloop

endfacet

facet normal 8.020838e-001 0.000000e+000 -5.972114e-001

outer loop

vertex 1.549449e+000 4.400000e+000 4.110896e+000

vertex 1.499459e+000 3.000000e+000 4.039502e+000

vertex 1.499459e+000 4.400000e+000 4.039502e+000

endloop

endfacet

facet normal 7.274188e-001 0.000000e+000 -6.861938e-001

outer loop

vertex 1.499459e+000 4.400000e+000 4.039502e+000

vertex 1.499459e+000 3.000000e+000 4.039502e+000

vertex 1.437830e+000 3.000000e+000 3.977873e+000

endloop

endfacet

facet normal 6.861938e-001 0.000000e+000 -7.274188e-001

outer loop

vertex 1.499459e+000 4.400000e+000 4.039502e+000

vertex 1.437830e+000 3.000000e+000 3.977873e+000

vertex 1.437830e+000 4.400000e+000 3.977873e+000

endloop

endfacet

facet normal 5.972114e-001 0.000000e+000 -8.020838e-001

outer loop

vertex 1.437830e+000 4.400000e+000 3.977873e+000

vertex 1.437830e+000 3.000000e+000 3.977873e+000

vertex 1.366436e+000 3.000000e+000 3.927883e+000

endloop

endfacet

facet normal 5.494540e-001 0.000000e+000 -8.355240e-001

outer loop

vertex 1.437830e+000 4.400000e+000 3.977873e+000

vertex 1.366436e+000 3.000000e+000 3.927883e+000

vertex 1.366436e+000 4.400000e+000 3.927883e+000

endloop

endfacet

facet normal 4.488580e-001 0.000000e+000 -8.936031e-001

outer loop

vertex 1.366436e+000 4.400000e+000 3.927883e+000

vertex 1.366436e+000 3.000000e+000 3.927883e+000

vertex 1.287446e+000 3.000000e+000 3.891049e+000

endloop

endfacet

facet normal 3.960194e-001 0.000000e+000 -9.182422e-001

outer loop

vertex 1.366436e+000 4.400000e+000 3.927883e+000

vertex 1.287446e+000 3.000000e+000 3.891049e+000

vertex 1.287446e+000 4.400000e+000 3.891049e+000

endloop

endfacet

facet normal 2.868663e-001 0.000000e+000 -9.579706e-001

outer loop

vertex 1.287446e+000 4.400000e+000 3.891049e+000

vertex 1.287446e+000 3.000000e+000 3.891049e+000

vertex 1.203260e+000 3.000000e+000 3.868492e+000

endloop

endfacet

facet normal 2.305519e-001 0.000000e+000 -9.730600e-001

outer loop

vertex 1.287446e+000 4.400000e+000 3.891049e+000

vertex 1.203260e+000 3.000000e+000 3.868492e+000

vertex 1.203260e+000 4.400000e+000 3.868492e+000

endloop

endfacet

facet normal 1.161583e-001 0.000000e+000 -9.932307e-001

outer loop

vertex 1.203260e+000 4.400000e+000 3.868492e+000

vertex 1.203260e+000 3.000000e+000 3.868492e+000

vertex 1.116436e+000 3.000000e+000 3.860896e+000

endloop

endfacet

facet normal 5.807913e-002 0.000000e+000 -9.983119e-001

outer loop

vertex 1.203260e+000 4.400000e+000 3.868492e+000

vertex 1.116436e+000 3.000000e+000 3.860896e+000

vertex 1.116436e+000 4.400000e+000 3.860896e+000

endloop

endfacet

facet normal -5.807913e-002 0.000000e+000 -9.983119e-001

outer loop

vertex 1.116436e+000 4.400000e+000 3.860896e+000

vertex 1.116436e+000 3.000000e+000 3.860896e+000

vertex 1.029613e+000 3.000000e+000 3.868492e+000

endloop

endfacet

facet normal -1.161583e-001 0.000000e+000 -9.932307e-001

outer loop

vertex 1.116436e+000 4.400000e+000 3.860896e+000

vertex 1.029613e+000 3.000000e+000 3.868492e+000

vertex 1.029613e+000 4.400000e+000 3.868492e+000

endloop

endfacet

facet normal -2.305519e-001 0.000000e+000 -9.730600e-001

outer loop

vertex 1.029613e+000 4.400000e+000 3.868492e+000

vertex 1.029613e+000 3.000000e+000 3.868492e+000

vertex 9.454265e-001 3.000000e+000 3.891049e+000

endloop

endfacet

facet normal -2.868663e-001 0.000000e+000 -9.579706e-001

outer loop

vertex 1.029613e+000 4.400000e+000 3.868492e+000

vertex 9.454265e-001 3.000000e+000 3.891049e+000

vertex 9.454265e-001 4.400000e+000 3.891049e+000

endloop

endfacet

facet normal -3.960194e-001 0.000000e+000 -9.182422e-001

outer loop

vertex 9.454265e-001 4.400000e+000 3.891049e+000

vertex 9.454265e-001 3.000000e+000 3.891049e+000

vertex 8.664364e-001 3.000000e+000 3.927883e+000

endloop

endfacet

facet normal -4.488580e-001 0.000000e+000 -8.936031e-001

outer loop

vertex 9.454265e-001 4.400000e+000 3.891049e+000

vertex 8.664364e-001 3.000000e+000 3.927883e+000

vertex 8.664364e-001 4.400000e+000 3.927883e+000

endloop

endfacet

facet normal -5.494540e-001 0.000000e+000 -8.355240e-001

outer loop

vertex 8.664364e-001 4.400000e+000 3.927883e+000

vertex 8.664364e-001 3.000000e+000 3.927883e+000

vertex 7.950426e-001 3.000000e+000 3.977873e+000

endloop

endfacet

facet normal -5.972114e-001 0.000000e+000 -8.020838e-001

outer loop

vertex 8.664364e-001 4.400000e+000 3.927883e+000

vertex 7.950426e-001 3.000000e+000 3.977873e+000

vertex 7.950426e-001 4.400000e+000 3.977873e+000

endloop

endfacet

facet normal -6.861938e-001 0.000000e+000 -7.274188e-001

outer loop

vertex 7.950426e-001 4.400000e+000 3.977873e+000

vertex 7.950426e-001 3.000000e+000 3.977873e+000

vertex 7.334142e-001 3.000000e+000 4.039502e+000

endloop

endfacet

facet normal -7.274188e-001 0.000000e+000 -6.861938e-001

outer loop

vertex 7.950426e-001 4.400000e+000 3.977873e+000

vertex 7.334142e-001 3.000000e+000 4.039502e+000

vertex 7.334142e-001 4.400000e+000 4.039502e+000

endloop

endfacet

facet normal -8.020838e-001 0.000000e+000 -5.972114e-001

outer loop

vertex 7.334142e-001 4.400000e+000 4.039502e+000

vertex 7.334142e-001 3.000000e+000 4.039502e+000

vertex 6.834236e-001 3.000000e+000 4.110896e+000

endloop

endfacet

facet normal -8.355240e-001 0.000000e+000 -5.494540e-001

outer loop

vertex 7.334142e-001 4.400000e+000 4.039502e+000

vertex 6.834236e-001 3.000000e+000 4.110896e+000

vertex 6.834236e-001 4.400000e+000 4.110896e+000

endloop

endfacet

facet normal -8.936031e-001 0.000000e+000 -4.488580e-001

outer loop

vertex 6.834236e-001 4.400000e+000 4.110896e+000

vertex 6.834236e-001 3.000000e+000 4.110896e+000

vertex 6.465902e-001 3.000000e+000 4.189886e+000

endloop

endfacet

facet normal -9.182422e-001 0.000000e+000 -3.960194e-001

outer loop

vertex 6.834236e-001 4.400000e+000 4.110896e+000

vertex 6.465902e-001 3.000000e+000 4.189886e+000

vertex 6.465902e-001 4.400000e+000 4.189886e+000

endloop

endfacet

facet normal -9.579706e-001 0.000000e+000 -2.868663e-001

outer loop

vertex 6.465902e-001 4.400000e+000 4.189886e+000

vertex 6.465902e-001 3.000000e+000 4.189886e+000

vertex 6.240327e-001 3.000000e+000 4.274072e+000

endloop

endfacet

facet normal -9.730600e-001 0.000000e+000 -2.305519e-001

outer loop

vertex 6.465902e-001 4.400000e+000 4.189886e+000

vertex 6.240327e-001 3.000000e+000 4.274072e+000

vertex 6.240327e-001 4.400000e+000 4.274072e+000

endloop

endfacet

facet normal -9.932307e-001 0.000000e+000 -1.161583e-001

outer loop

vertex 6.240327e-001 4.400000e+000 4.274072e+000

vertex 6.240327e-001 3.000000e+000 4.274072e+000

vertex 6.164364e-001 3.000000e+000 4.360896e+000

endloop

endfacet

facet normal -9.983119e-001 0.000000e+000 -5.807913e-002

outer loop

vertex 6.240327e-001 4.400000e+000 4.274072e+000

vertex 6.164364e-001 3.000000e+000 4.360896e+000

vertex 6.164364e-001 4.400000e+000 4.360896e+000

endloop

endfacet

facet normal -9.983119e-001 0.000000e+000 5.807913e-002

outer loop

vertex 6.164364e-001 4.400000e+000 4.360896e+000

vertex 6.164364e-001 3.000000e+000 4.360896e+000

vertex 6.240327e-001 3.000000e+000 4.447720e+000

endloop

endfacet

facet normal -9.932307e-001 0.000000e+000 1.161583e-001

outer loop

vertex 6.164364e-001 4.400000e+000 4.360896e+000

vertex 6.240327e-001 3.000000e+000 4.447720e+000

vertex 6.240327e-001 4.400000e+000 4.447720e+000

endloop

endfacet

facet normal -9.730600e-001 0.000000e+000 2.305519e-001

outer loop

vertex 6.240327e-001 4.400000e+000 4.447720e+000

vertex 6.240327e-001 3.000000e+000 4.447720e+000

vertex 6.465902e-001 3.000000e+000 4.531906e+000

endloop

endfacet

facet normal -9.579706e-001 0.000000e+000 2.868663e-001

outer loop

vertex 6.240327e-001 4.400000e+000 4.447720e+000

vertex 6.465902e-001 3.000000e+000 4.531906e+000

vertex 6.465902e-001 4.400000e+000 4.531906e+000

endloop

endfacet

facet normal -9.182422e-001 0.000000e+000 3.960194e-001

outer loop

vertex 6.465902e-001 4.400000e+000 4.531906e+000

vertex 6.465902e-001 3.000000e+000 4.531906e+000

vertex 6.834236e-001 3.000000e+000 4.610896e+000

endloop

endfacet

facet normal -8.936031e-001 0.000000e+000 4.488580e-001

outer loop

vertex 6.465902e-001 4.400000e+000 4.531906e+000

vertex 6.834236e-001 3.000000e+000 4.610896e+000

vertex 6.834236e-001 4.400000e+000 4.610896e+000

endloop

endfacet

facet normal -8.355240e-001 0.000000e+000 5.494540e-001

outer loop

vertex 6.834236e-001 4.400000e+000 4.610896e+000

vertex 6.834236e-001 3.000000e+000 4.610896e+000

vertex 7.334142e-001 3.000000e+000 4.682290e+000

endloop

endfacet

facet normal -8.020838e-001 0.000000e+000 5.972114e-001

outer loop

vertex 6.834236e-001 4.400000e+000 4.610896e+000

vertex 7.334142e-001 3.000000e+000 4.682290e+000

vertex 7.334142e-001 4.400000e+000 4.682290e+000

endloop

endfacet

facet normal -7.274188e-001 0.000000e+000 6.861938e-001

outer loop

vertex 7.334142e-001 4.400000e+000 4.682290e+000

vertex 7.334142e-001 3.000000e+000 4.682290e+000

vertex 7.950426e-001 3.000000e+000 4.743918e+000

endloop

endfacet

facet normal -6.861938e-001 0.000000e+000 7.274188e-001

outer loop

vertex 7.334142e-001 4.400000e+000 4.682290e+000

vertex 7.950426e-001 3.000000e+000 4.743918e+000

vertex 7.950426e-001 4.400000e+000 4.743918e+000

endloop

endfacet

facet normal -5.972114e-001 0.000000e+000 8.020838e-001

outer loop

vertex 7.950426e-001 4.400000e+000 4.743918e+000

vertex 7.950426e-001 3.000000e+000 4.743918e+000

vertex 8.664364e-001 3.000000e+000 4.793909e+000

endloop

endfacet

facet normal -5.494540e-001 0.000000e+000 8.355240e-001

outer loop

vertex 7.950426e-001 4.400000e+000 4.743918e+000

vertex 8.664364e-001 3.000000e+000 4.793909e+000

vertex 8.664364e-001 4.400000e+000 4.793909e+000

endloop

endfacet

facet normal -4.488580e-001 0.000000e+000 8.936031e-001

outer loop

vertex 8.664364e-001 4.400000e+000 4.793909e+000

vertex 8.664364e-001 3.000000e+000 4.793909e+000

vertex 9.454265e-001 3.000000e+000 4.830742e+000

endloop

endfacet

facet normal -3.960194e-001 0.000000e+000 9.182422e-001

outer loop

vertex 8.664364e-001 4.400000e+000 4.793909e+000

vertex 9.454265e-001 3.000000e+000 4.830742e+000

vertex 9.454265e-001 4.400000e+000 4.830742e+000

endloop

endfacet

facet normal -2.868663e-001 0.000000e+000 9.579706e-001

outer loop

vertex 9.454265e-001 4.400000e+000 4.830742e+000

vertex 9.454265e-001 3.000000e+000 4.830742e+000

vertex 1.029613e+000 3.000000e+000 4.853300e+000

endloop

endfacet

facet normal -2.305519e-001 0.000000e+000 9.730600e-001

outer loop

vertex 9.454265e-001 4.400000e+000 4.830742e+000

vertex 1.029613e+000 3.000000e+000 4.853300e+000

vertex 1.029613e+000 4.400000e+000 4.853300e+000

endloop

endfacet

facet normal -1.161583e-001 0.000000e+000 9.932307e-001

outer loop

vertex 1.029613e+000 4.400000e+000 4.853300e+000

vertex 1.029613e+000 3.000000e+000 4.853300e+000

vertex 1.116436e+000 3.000000e+000 4.860896e+000

endloop

endfacet

facet normal -5.807913e-002 0.000000e+000 9.983119e-001

outer loop

vertex 1.029613e+000 4.400000e+000 4.853300e+000

vertex 1.116436e+000 3.000000e+000 4.860896e+000

vertex 1.116436e+000 4.400000e+000 4.860896e+000

endloop

endfacet

facet normal 5.807913e-002 0.000000e+000 9.983119e-001

outer loop

vertex 1.116436e+000 4.400000e+000 4.860896e+000

vertex 1.116436e+000 3.000000e+000 4.860896e+000

vertex 1.203260e+000 3.000000e+000 4.853300e+000

endloop

endfacet

facet normal 1.161583e-001 0.000000e+000 9.932307e-001

outer loop

vertex 1.078841e+001 4.400000e+000 8.628060e+000

vertex 1.087523e+001 3.000000e+000 8.620463e+000

vertex 1.087523e+001 4.400000e+000 8.620463e+000

endloop

endfacet

facet normal 2.305519e-001 0.000000e+000 9.730600e-001

outer loop

vertex 1.087523e+001 4.400000e+000 8.620463e+000

vertex 1.087523e+001 3.000000e+000 8.620463e+000

vertex 1.095942e+001 3.000000e+000 8.597907e+000

endloop

endfacet

facet normal 2.868663e-001 0.000000e+000 9.579706e-001

outer loop

vertex 1.087523e+001 4.400000e+000 8.620463e+000

vertex 1.095942e+001 3.000000e+000 8.597907e+000

vertex 1.095942e+001 4.400000e+000 8.597907e+000

endloop

endfacet

facet normal 3.960194e-001 0.000000e+000 9.182422e-001

outer loop

vertex 1.095942e+001 4.400000e+000 8.597907e+000

vertex 1.095942e+001 3.000000e+000 8.597907e+000

vertex 1.103841e+001 3.000000e+000 8.561073e+000

endloop

endfacet

facet normal 4.488580e-001 0.000000e+000 8.936031e-001

outer loop

vertex 1.095942e+001 4.400000e+000 8.597907e+000

vertex 1.103841e+001 3.000000e+000 8.561073e+000

vertex 1.103841e+001 4.400000e+000 8.561073e+000

endloop

endfacet

facet normal 5.494540e-001 0.000000e+000 8.355240e-001

outer loop

vertex 1.103841e+001 4.400000e+000 8.561073e+000

vertex 1.103841e+001 3.000000e+000 8.561073e+000

vertex 1.110980e+001 3.000000e+000 8.511083e+000

endloop

endfacet

facet normal 5.972114e-001 0.000000e+000 8.020838e-001

outer loop

vertex 1.103841e+001 4.400000e+000 8.561073e+000

vertex 1.110980e+001 3.000000e+000 8.511083e+000

vertex 1.110980e+001 4.400000e+000 8.511083e+000

endloop

endfacet

facet normal 6.861938e-001 0.000000e+000 7.274188e-001

outer loop

vertex 1.110980e+001 4.400000e+000 8.511083e+000

vertex 1.110980e+001 3.000000e+000 8.511083e+000

vertex 1.117143e+001 3.000000e+000 8.449454e+000

endloop

endfacet

facet normal 7.274188e-001 0.000000e+000 6.861938e-001

outer loop

vertex 1.110980e+001 4.400000e+000 8.511083e+000

vertex 1.117143e+001 3.000000e+000 8.449454e+000

vertex 1.117143e+001 4.400000e+000 8.449454e+000

endloop

endfacet

facet normal 8.020838e-001 0.000000e+000 5.972114e-001

outer loop

vertex 1.117143e+001 4.400000e+000 8.449454e+000

vertex 1.117143e+001 3.000000e+000 8.449454e+000

vertex 1.122142e+001 3.000000e+000 8.378060e+000

endloop

endfacet

facet normal 8.355240e-001 0.000000e+000 5.494540e-001

outer loop

vertex 1.117143e+001 4.400000e+000 8.449454e+000

vertex 1.122142e+001 3.000000e+000 8.378060e+000

vertex 1.122142e+001 4.400000e+000 8.378060e+000

endloop

endfacet

facet normal 8.936031e-001 0.000000e+000 4.488580e-001

outer loop

vertex 1.122142e+001 4.400000e+000 8.378060e+000

vertex 1.122142e+001 3.000000e+000 8.378060e+000

vertex 1.125826e+001 3.000000e+000 8.299070e+000

endloop

endfacet

facet normal 9.182422e-001 0.000000e+000 3.960194e-001

outer loop

vertex 1.122142e+001 4.400000e+000 8.378060e+000

vertex 1.125826e+001 3.000000e+000 8.299070e+000

vertex 1.125826e+001 4.400000e+000 8.299070e+000

endloop

endfacet

facet normal 9.579706e-001 0.000000e+000 2.868663e-001

outer loop

vertex 1.125826e+001 4.400000e+000 8.299070e+000

vertex 1.125826e+001 3.000000e+000 8.299070e+000

vertex 1.128081e+001 3.000000e+000 8.214885e+000

endloop

endfacet

facet normal 9.730600e-001 0.000000e+000 2.305519e-001

outer loop

vertex 1.125826e+001 4.400000e+000 8.299070e+000

vertex 1.128081e+001 3.000000e+000 8.214885e+000

vertex 1.128081e+001 4.400000e+000 8.214885e+000

endloop

endfacet

facet normal 9.932307e-001 0.000000e+000 1.161583e-001

outer loop

vertex 1.128081e+001 4.400000e+000 8.214885e+000

vertex 1.128081e+001 3.000000e+000 8.214885e+000

vertex 1.128841e+001 3.000000e+000 8.128060e+000

endloop

endfacet

facet normal 9.983119e-001 0.000000e+000 5.807913e-002

outer loop

vertex 1.128081e+001 4.400000e+000 8.214885e+000

vertex 1.128841e+001 3.000000e+000 8.128060e+000

vertex 1.128841e+001 4.400000e+000 8.128060e+000

endloop

endfacet

facet normal 9.983119e-001 0.000000e+000 -5.807913e-002

outer loop

vertex 1.128841e+001 4.400000e+000 8.128060e+000

vertex 1.128841e+001 3.000000e+000 8.128060e+000

vertex 1.128081e+001 3.000000e+000 8.041236e+000

endloop

endfacet

facet normal 9.932307e-001 0.000000e+000 -1.161583e-001

outer loop

vertex 1.128841e+001 4.400000e+000 8.128060e+000

vertex 1.128081e+001 3.000000e+000 8.041236e+000

vertex 1.128081e+001 4.400000e+000 8.041236e+000

endloop

endfacet

facet normal 9.730600e-001 0.000000e+000 -2.305519e-001

outer loop

vertex 1.128081e+001 4.400000e+000 8.041236e+000

vertex 1.128081e+001 3.000000e+000 8.041236e+000

vertex 1.125826e+001 3.000000e+000 7.957050e+000

endloop

endfacet

facet normal 9.579706e-001 0.000000e+000 -2.868663e-001

outer loop

vertex 1.128081e+001 4.400000e+000 8.041236e+000

vertex 1.125826e+001 3.000000e+000 7.957050e+000

vertex 1.125826e+001 4.400000e+000 7.957050e+000

endloop

endfacet

facet normal 9.182422e-001 0.000000e+000 -3.960194e-001

outer loop

vertex 1.125826e+001 4.400000e+000 7.957050e+000

vertex 1.125826e+001 3.000000e+000 7.957050e+000

vertex 1.122142e+001 3.000000e+000 7.878059e+000

endloop

endfacet

facet normal 8.936031e-001 0.000000e+000 -4.488580e-001

outer loop

vertex 1.125826e+001 4.400000e+000 7.957050e+000

vertex 1.122142e+001 3.000000e+000 7.878059e+000

vertex 1.122142e+001 4.400000e+000 7.878059e+000

endloop

endfacet

facet normal 8.355240e-001 0.000000e+000 -5.494540e-001

outer loop

vertex 1.122142e+001 4.400000e+000 7.878059e+000

vertex 1.122142e+001 3.000000e+000 7.878059e+000

vertex 1.117143e+001 3.000000e+000 7.806666e+000

endloop

endfacet

facet normal 8.020838e-001 0.000000e+000 -5.972114e-001

outer loop

vertex 1.122142e+001 4.400000e+000 7.878059e+000

vertex 1.117143e+001 3.000000e+000 7.806666e+000

vertex 1.117143e+001 4.400000e+000 7.806666e+000

endloop

endfacet

facet normal 7.274188e-001 0.000000e+000 -6.861938e-001

outer loop

vertex 1.117143e+001 4.400000e+000 7.806666e+000

vertex 1.117143e+001 3.000000e+000 7.806666e+000

vertex 1.110980e+001 3.000000e+000 7.745038e+000

endloop

endfacet

facet normal 6.861938e-001 0.000000e+000 -7.274188e-001

outer loop

vertex 1.117143e+001 4.400000e+000 7.806666e+000

vertex 1.110980e+001 3.000000e+000 7.745038e+000

vertex 1.110980e+001 4.400000e+000 7.745038e+000

endloop

endfacet

facet normal 5.972114e-001 0.000000e+000 -8.020838e-001

outer loop

vertex 1.110980e+001 4.400000e+000 7.745038e+000

vertex 1.110980e+001 3.000000e+000 7.745038e+000

vertex 1.103841e+001 3.000000e+000 7.695047e+000

endloop

endfacet

facet normal 5.494540e-001 0.000000e+000 -8.355240e-001

outer loop

vertex 1.110980e+001 4.400000e+000 7.745038e+000

vertex 1.103841e+001 3.000000e+000 7.695047e+000

vertex 1.103841e+001 4.400000e+000 7.695047e+000

endloop

endfacet

facet normal 4.488580e-001 0.000000e+000 -8.936031e-001

outer loop

vertex 1.103841e+001 4.400000e+000 7.695047e+000

vertex 1.103841e+001 3.000000e+000 7.695047e+000

vertex 1.095942e+001 3.000000e+000 7.658214e+000

endloop

endfacet

facet normal 3.960194e-001 0.000000e+000 -9.182422e-001

outer loop

vertex 1.103841e+001 4.400000e+000 7.695047e+000

vertex 1.095942e+001 3.000000e+000 7.658214e+000

vertex 1.095942e+001 4.400000e+000 7.658214e+000

endloop

endfacet

facet normal 2.868663e-001 0.000000e+000 -9.579706e-001

outer loop

vertex 1.095942e+001 4.400000e+000 7.658214e+000

vertex 1.095942e+001 3.000000e+000 7.658214e+000

vertex 1.087523e+001 3.000000e+000 7.635656e+000

endloop

endfacet

facet normal 2.305519e-001 0.000000e+000 -9.730600e-001

outer loop

vertex 1.095942e+001 4.400000e+000 7.658214e+000

vertex 1.087523e+001 3.000000e+000 7.635656e+000

vertex 1.087523e+001 4.400000e+000 7.635656e+000

endloop

endfacet

facet normal 1.161583e-001 0.000000e+000 -9.932307e-001

outer loop

vertex 1.087523e+001 4.400000e+000 7.635656e+000

vertex 1.087523e+001 3.000000e+000 7.635656e+000

vertex 1.078841e+001 3.000000e+000 7.628060e+000

endloop

endfacet

facet normal 5.807913e-002 0.000000e+000 -9.983119e-001

outer loop

vertex 1.087523e+001 4.400000e+000 7.635656e+000

vertex 1.078841e+001 3.000000e+000 7.628060e+000

vertex 1.078841e+001 4.400000e+000 7.628060e+000

endloop

endfacet

facet normal -5.807913e-002 0.000000e+000 -9.983119e-001

outer loop

vertex 1.078841e+001 4.400000e+000 7.628060e+000

vertex 1.078841e+001 3.000000e+000 7.628060e+000

vertex 1.070159e+001 3.000000e+000 7.635656e+000

endloop

endfacet

facet normal -1.161583e-001 0.000000e+000 -9.932307e-001

outer loop

vertex 1.078841e+001 4.400000e+000 7.628060e+000

vertex 1.070159e+001 3.000000e+000 7.635656e+000

vertex 1.070159e+001 4.400000e+000 7.635656e+000

endloop

endfacet

facet normal -2.305519e-001 0.000000e+000 -9.730600e-001

outer loop

vertex 1.070159e+001 4.400000e+000 7.635656e+000

vertex 1.070159e+001 3.000000e+000 7.635656e+000

vertex 1.061740e+001 3.000000e+000 7.658214e+000

endloop

endfacet

facet normal -2.868663e-001 0.000000e+000 -9.579706e-001

outer loop

vertex 1.070159e+001 4.400000e+000 7.635656e+000

vertex 1.061740e+001 3.000000e+000 7.658214e+000

vertex 1.061740e+001 4.400000e+000 7.658214e+000

endloop

endfacet

facet normal -3.960194e-001 0.000000e+000 -9.182422e-001

outer loop

vertex 1.061740e+001 4.400000e+000 7.658214e+000

vertex 1.061740e+001 3.000000e+000 7.658214e+000

vertex 1.053841e+001 3.000000e+000 7.695047e+000

endloop

endfacet

facet normal -4.488580e-001 0.000000e+000 -8.936031e-001

outer loop

vertex 1.061740e+001 4.400000e+000 7.658214e+000

vertex 1.053841e+001 3.000000e+000 7.695047e+000

vertex 1.053841e+001 4.400000e+000 7.695047e+000

endloop

endfacet

facet normal -5.494540e-001 0.000000e+000 -8.355240e-001

outer loop

vertex 1.053841e+001 4.400000e+000 7.695047e+000

vertex 1.053841e+001 3.000000e+000 7.695047e+000

vertex 1.046702e+001 3.000000e+000 7.745038e+000

endloop

endfacet

facet normal -5.972114e-001 0.000000e+000 -8.020838e-001

outer loop

vertex 1.053841e+001 4.400000e+000 7.695047e+000

vertex 1.046702e+001 3.000000e+000 7.745038e+000

vertex 1.046702e+001 4.400000e+000 7.745038e+000

endloop

endfacet

facet normal -6.861938e-001 0.000000e+000 -7.274188e-001

outer loop

vertex 1.046702e+001 4.400000e+000 7.745038e+000

vertex 1.046702e+001 3.000000e+000 7.745038e+000

vertex 1.040539e+001 3.000000e+000 7.806666e+000

endloop

endfacet

facet normal -7.274188e-001 0.000000e+000 -6.861938e-001

outer loop

vertex 1.046702e+001 4.400000e+000 7.745038e+000

vertex 1.040539e+001 3.000000e+000 7.806666e+000

vertex 1.040539e+001 4.400000e+000 7.806666e+000

endloop

endfacet

facet normal -8.020838e-001 0.000000e+000 -5.972114e-001

outer loop

vertex 1.040539e+001 4.400000e+000 7.806666e+000

vertex 1.040539e+001 3.000000e+000 7.806666e+000

vertex 1.035540e+001 3.000000e+000 7.878059e+000

endloop

endfacet

facet normal -8.355240e-001 0.000000e+000 -5.494540e-001

outer loop

vertex 1.040539e+001 4.400000e+000 7.806666e+000

vertex 1.035540e+001 3.000000e+000 7.878059e+000

vertex 1.035540e+001 4.400000e+000 7.878059e+000

endloop

endfacet

facet normal -8.936031e-001 0.000000e+000 -4.488580e-001

outer loop

vertex 1.035540e+001 4.400000e+000 7.878059e+000

vertex 1.035540e+001 3.000000e+000 7.878059e+000

vertex 1.031856e+001 3.000000e+000 7.957050e+000

endloop

endfacet

facet normal -9.182422e-001 0.000000e+000 -3.960194e-001

outer loop

vertex 1.035540e+001 4.400000e+000 7.878059e+000

vertex 1.031856e+001 3.000000e+000 7.957050e+000

vertex 1.031856e+001 4.400000e+000 7.957050e+000

endloop

endfacet

facet normal -9.579706e-001 0.000000e+000 -2.868663e-001

outer loop

vertex 1.031856e+001 4.400000e+000 7.957050e+000

vertex 1.031856e+001 3.000000e+000 7.957050e+000

vertex 1.029601e+001 3.000000e+000 8.041236e+000

endloop

endfacet

facet normal -9.730600e-001 0.000000e+000 -2.305519e-001

outer loop

vertex 1.031856e+001 4.400000e+000 7.957050e+000

vertex 1.029601e+001 3.000000e+000 8.041236e+000

vertex 1.029601e+001 4.400000e+000 8.041236e+000

endloop

endfacet

facet normal -9.932307e-001 0.000000e+000 -1.161583e-001

outer loop

vertex 1.029601e+001 4.400000e+000 8.041236e+000

vertex 1.029601e+001 3.000000e+000 8.041236e+000

vertex 1.028841e+001 3.000000e+000 8.128060e+000

endloop

endfacet

facet normal -9.983119e-001 0.000000e+000 -5.807913e-002

outer loop

vertex 1.029601e+001 4.400000e+000 8.041236e+000

vertex 1.028841e+001 3.000000e+000 8.128060e+000

vertex 1.028841e+001 4.400000e+000 8.128060e+000

endloop

endfacet

facet normal -9.983119e-001 0.000000e+000 5.807913e-002

outer loop

vertex 1.028841e+001 4.400000e+000 8.128060e+000

vertex 1.028841e+001 3.000000e+000 8.128060e+000

vertex 1.029601e+001 3.000000e+000 8.214885e+000

endloop

endfacet

facet normal -9.932307e-001 0.000000e+000 1.161583e-001

outer loop

vertex 1.028841e+001 4.400000e+000 8.128060e+000

vertex 1.029601e+001 3.000000e+000 8.214885e+000

vertex 1.029601e+001 4.400000e+000 8.214885e+000

endloop

endfacet

facet normal -9.730600e-001 0.000000e+000 2.305519e-001

outer loop

vertex 1.029601e+001 4.400000e+000 8.214885e+000

vertex 1.029601e+001 3.000000e+000 8.214885e+000

vertex 1.031856e+001 3.000000e+000 8.299070e+000

endloop

endfacet

facet normal -9.579706e-001 0.000000e+000 2.868663e-001

outer loop

vertex 1.029601e+001 4.400000e+000 8.214885e+000

vertex 1.031856e+001 3.000000e+000 8.299070e+000

vertex 1.031856e+001 4.400000e+000 8.299070e+000

endloop

endfacet

facet normal -9.182422e-001 0.000000e+000 3.960194e-001

outer loop

vertex 1.031856e+001 4.400000e+000 8.299070e+000

vertex 1.031856e+001 3.000000e+000 8.299070e+000

vertex 1.035540e+001 3.000000e+000 8.378060e+000

endloop

endfacet

facet normal -8.936031e-001 0.000000e+000 4.488580e-001

outer loop

vertex 1.031856e+001 4.400000e+000 8.299070e+000

vertex 1.035540e+001 3.000000e+000 8.378060e+000

vertex 1.035540e+001 4.400000e+000 8.378060e+000

endloop

endfacet

facet normal -8.355240e-001 0.000000e+000 5.494540e-001

outer loop

vertex 1.035540e+001 4.400000e+000 8.378060e+000

vertex 1.035540e+001 3.000000e+000 8.378060e+000

vertex 1.040539e+001 3.000000e+000 8.449454e+000

endloop

endfacet

facet normal -8.020838e-001 0.000000e+000 5.972114e-001

outer loop

vertex 1.035540e+001 4.400000e+000 8.378060e+000

vertex 1.040539e+001 3.000000e+000 8.449454e+000

vertex 1.040539e+001 4.400000e+000 8.449454e+000

endloop

endfacet

facet normal -7.274188e-001 0.000000e+000 6.861938e-001

outer loop

vertex 1.040539e+001 4.400000e+000 8.449454e+000

vertex 1.040539e+001 3.000000e+000 8.449454e+000

vertex 1.046702e+001 3.000000e+000 8.511083e+000

endloop

endfacet

facet normal -6.861938e-001 0.000000e+000 7.274188e-001

outer loop

vertex 1.040539e+001 4.400000e+000 8.449454e+000

vertex 1.046702e+001 3.000000e+000 8.511083e+000

vertex 1.046702e+001 4.400000e+000 8.511083e+000

endloop

endfacet

facet normal -5.972114e-001 0.000000e+000 8.020838e-001

outer loop

vertex 1.046702e+001 4.400000e+000 8.511083e+000

vertex 1.046702e+001 3.000000e+000 8.511083e+000

vertex 1.053841e+001 3.000000e+000 8.561073e+000

endloop

endfacet

facet normal -5.494540e-001 0.000000e+000 8.355240e-001

outer loop

vertex 1.046702e+001 4.400000e+000 8.511083e+000

vertex 1.053841e+001 3.000000e+000 8.561073e+000

vertex 1.053841e+001 4.400000e+000 8.561073e+000

endloop

endfacet

facet normal -4.488580e-001 0.000000e+000 8.936031e-001

outer loop

vertex 1.053841e+001 4.400000e+000 8.561073e+000

vertex 1.053841e+001 3.000000e+000 8.561073e+000

vertex 1.061740e+001 3.000000e+000 8.597907e+000

endloop

endfacet

facet normal -3.960194e-001 0.000000e+000 9.182422e-001

outer loop

vertex 1.053841e+001 4.400000e+000 8.561073e+000

vertex 1.061740e+001 3.000000e+000 8.597907e+000

vertex 1.061740e+001 4.400000e+000 8.597907e+000

endloop

endfacet

facet normal -2.868663e-001 0.000000e+000 9.579706e-001

outer loop

vertex 1.061740e+001 4.400000e+000 8.597907e+000

vertex 1.061740e+001 3.000000e+000 8.597907e+000

vertex 1.070159e+001 3.000000e+000 8.620463e+000

endloop

endfacet

facet normal -2.305519e-001 0.000000e+000 9.730600e-001

outer loop

vertex 1.061740e+001 4.400000e+000 8.597907e+000

vertex 1.070159e+001 3.000000e+000 8.620463e+000

vertex 1.070159e+001 4.400000e+000 8.620463e+000

endloop

endfacet

facet normal -1.161583e-001 0.000000e+000 9.932307e-001

outer loop

vertex 1.070159e+001 4.400000e+000 8.620463e+000

vertex 1.070159e+001 3.000000e+000 8.620463e+000

vertex 1.078841e+001 3.000000e+000 8.628060e+000

endloop

endfacet

facet normal -5.807913e-002 0.000000e+000 9.983119e-001

outer loop

vertex 1.070159e+001 4.400000e+000 8.620463e+000

vertex 1.078841e+001 3.000000e+000 8.628060e+000

vertex 1.078841e+001 4.400000e+000 8.628060e+000

endloop

endfacet

facet normal 5.807913e-002 0.000000e+000 9.983119e-001

outer loop

vertex 1.078841e+001 4.400000e+000 8.628060e+000

vertex 1.078841e+001 3.000000e+000 8.628060e+000

vertex 1.087523e+001 3.000000e+000 8.620463e+000

endloop

endfacet

facet normal -9.983119e-001 0.000000e+000 -5.807913e-002

outer loop

vertex 7.579920e+000 4.400000e+000 8.041236e+000

vertex 7.572323e+000 3.000000e+000 8.128060e+000

vertex 7.572323e+000 4.400000e+000 8.128060e+000

endloop

endfacet

facet normal -9.983119e-001 0.000000e+000 5.807913e-002

outer loop

vertex 7.572323e+000 4.400000e+000 8.128060e+000

vertex 7.572323e+000 3.000000e+000 8.128060e+000

vertex 7.579920e+000 3.000000e+000 8.214885e+000

endloop

endfacet

facet normal -9.932307e-001 0.000000e+000 1.161583e-001

outer loop

vertex 7.572323e+000 4.400000e+000 8.128060e+000

vertex 7.579920e+000 3.000000e+000 8.214885e+000

vertex 7.579920e+000 4.400000e+000 8.214885e+000

endloop

endfacet

facet normal -9.776480e-001 0.000000e+000 2.102483e-001

outer loop

vertex 7.579920e+000 4.400000e+000 8.214885e+000

vertex 7.579920e+000 3.000000e+000 8.214885e+000

vertex 7.594380e+000 3.000000e+000 8.268851e+000

endloop

endfacet

facet normal -9.637589e-001 0.000000e+000 2.667744e-001

outer loop

vertex 7.579920e+000 4.400000e+000 8.214885e+000

vertex 7.594380e+000 3.000000e+000 8.268851e+000

vertex 7.602478e+000 4.400000e+000 8.299070e+000

endloop

endfacet

facet normal -9.466226e-001 0.000000e+000 3.223439e-001

outer loop

vertex 7.594380e+000 3.000000e+000 8.268851e+000

vertex 7.602478e+000 3.000000e+000 8.299070e+000

vertex 7.602478e+000 4.400000e+000 8.299070e+000

endloop

endfacet

facet normal -9.182422e-001 0.000000e+000 3.960194e-001

outer loop

vertex 7.602478e+000 4.400000e+000 8.299070e+000

vertex 7.602478e+000 3.000000e+000 8.299070e+000

vertex 7.639311e+000 3.000000e+000 8.378060e+000

endloop

endfacet

facet normal -8.936031e-001 0.000000e+000 4.488580e-001

outer loop

vertex 7.602478e+000 4.400000e+000 8.299070e+000

vertex 7.639311e+000 3.000000e+000 8.378060e+000

vertex 7.639311e+000 4.400000e+000 8.378060e+000

endloop

endfacet

facet normal -8.355240e-001 0.000000e+000 5.494540e-001

outer loop

vertex 7.639311e+000 4.400000e+000 8.378060e+000

vertex 7.639311e+000 3.000000e+000 8.378060e+000

vertex 7.689301e+000 3.000000e+000 8.449454e+000

endloop

endfacet

facet normal -8.020838e-001 0.000000e+000 5.972114e-001

outer loop

vertex 7.639311e+000 4.400000e+000 8.378060e+000

vertex 7.689301e+000 3.000000e+000 8.449454e+000

vertex 7.689301e+000 4.400000e+000 8.449454e+000

endloop

endfacet

facet normal -7.274188e-001 0.000000e+000 6.861938e-001

outer loop

vertex 7.689301e+000 4.400000e+000 8.449454e+000

vertex 7.689301e+000 3.000000e+000 8.449454e+000

vertex 7.750930e+000 3.000000e+000 8.511083e+000

endloop

endfacet

facet normal -6.861938e-001 0.000000e+000 7.274188e-001

outer loop

vertex 7.689301e+000 4.400000e+000 8.449454e+000

vertex 7.750930e+000 3.000000e+000 8.511083e+000

vertex 7.750930e+000 4.400000e+000 8.511083e+000

endloop

endfacet

facet normal -5.972114e-001 0.000000e+000 8.020838e-001

outer loop

vertex 7.750930e+000 4.400000e+000 8.511083e+000

vertex 7.750930e+000 3.000000e+000 8.511083e+000

vertex 7.822324e+000 3.000000e+000 8.561073e+000

endloop

endfacet

facet normal -5.494540e-001 0.000000e+000 8.355240e-001

outer loop

vertex 7.750930e+000 4.400000e+000 8.511083e+000

vertex 7.822324e+000 3.000000e+000 8.561073e+000

vertex 7.822324e+000 4.400000e+000 8.561073e+000

endloop

endfacet

facet normal -4.488580e-001 0.000000e+000 8.936031e-001

outer loop

vertex 7.822324e+000 4.400000e+000 8.561073e+000

vertex 7.822324e+000 3.000000e+000 8.561073e+000

vertex 7.901314e+000 3.000000e+000 8.597907e+000

endloop

endfacet

facet normal -3.960194e-001 0.000000e+000 9.182422e-001

outer loop

vertex 7.822324e+000 4.400000e+000 8.561073e+000

vertex 7.901314e+000 3.000000e+000 8.597907e+000

vertex 7.901314e+000 4.400000e+000 8.597907e+000

endloop

endfacet

facet normal -2.868663e-001 0.000000e+000 9.579706e-001

outer loop

vertex 7.901314e+000 4.400000e+000 8.597907e+000

vertex 7.901314e+000 3.000000e+000 8.597907e+000

vertex 7.985500e+000 3.000000e+000 8.620463e+000

endloop

endfacet

facet normal -2.305519e-001 0.000000e+000 9.730600e-001

outer loop

vertex 7.901314e+000 4.400000e+000 8.597907e+000

vertex 7.985500e+000 3.000000e+000 8.620463e+000

vertex 7.985500e+000 4.400000e+000 8.620463e+000

endloop

endfacet

facet normal -1.161583e-001 0.000000e+000 9.932307e-001

outer loop

vertex 7.985500e+000 4.400000e+000 8.620463e+000

vertex 7.985500e+000 3.000000e+000 8.620463e+000

vertex 8.072324e+000 3.000000e+000 8.628060e+000

endloop

endfacet

facet normal -5.807913e-002 0.000000e+000 9.983119e-001

outer loop

vertex 7.985500e+000 4.400000e+000 8.620463e+000

vertex 8.072324e+000 3.000000e+000 8.628060e+000

vertex 8.072324e+000 4.400000e+000 8.628060e+000

endloop

endfacet

facet normal 5.807913e-002 0.000000e+000 9.983119e-001

outer loop

vertex 8.072324e+000 4.400000e+000 8.628060e+000

vertex 8.072324e+000 3.000000e+000 8.628060e+000

vertex 8.159147e+000 3.000000e+000 8.620463e+000

endloop

endfacet

facet normal 1.161583e-001 0.000000e+000 9.932307e-001

outer loop

vertex 8.072324e+000 4.400000e+000 8.628060e+000

vertex 8.159147e+000 3.000000e+000 8.620463e+000

vertex 8.159147e+000 4.400000e+000 8.620463e+000

endloop

endfacet

facet normal 2.305519e-001 0.000000e+000 9.730600e-001

outer loop

vertex 8.159147e+000 4.400000e+000 8.620463e+000

vertex 8.159147e+000 3.000000e+000 8.620463e+000

vertex 8.243334e+000 3.000000e+000 8.597907e+000

endloop

endfacet

facet normal 2.868663e-001 0.000000e+000 9.579706e-001

outer loop

vertex 8.159147e+000 4.400000e+000 8.620463e+000

vertex 8.243334e+000 3.000000e+000 8.597907e+000

vertex 8.243334e+000 4.400000e+000 8.597907e+000

endloop

endfacet

facet normal 3.960194e-001 0.000000e+000 9.182422e-001

outer loop

vertex 8.243334e+000 4.400000e+000 8.597907e+000

vertex 8.243334e+000 3.000000e+000 8.597907e+000

vertex 8.322324e+000 3.000000e+000 8.561073e+000

endloop

endfacet

facet normal 4.488580e-001 0.000000e+000 8.936031e-001

outer loop

vertex 8.243334e+000 4.400000e+000 8.597907e+000

vertex 8.322324e+000 3.000000e+000 8.561073e+000

vertex 8.322324e+000 4.400000e+000 8.561073e+000

endloop

endfacet

facet normal 5.494540e-001 0.000000e+000 8.355240e-001

outer loop

vertex 8.322324e+000 4.400000e+000 8.561073e+000

vertex 8.322324e+000 3.000000e+000 8.561073e+000

vertex 8.393718e+000 3.000000e+000 8.511083e+000

endloop

endfacet

facet normal 5.972114e-001 0.000000e+000 8.020838e-001

outer loop

vertex 8.322324e+000 4.400000e+000 8.561073e+000

vertex 8.393718e+000 3.000000e+000 8.511083e+000

vertex 8.393718e+000 4.400000e+000 8.511083e+000

endloop

endfacet

facet normal 6.861938e-001 0.000000e+000 7.274188e-001

outer loop

vertex 8.393718e+000 4.400000e+000 8.511083e+000

vertex 8.393718e+000 3.000000e+000 8.511083e+000

vertex 8.455345e+000 3.000000e+000 8.449454e+000

endloop

endfacet

facet normal 7.274188e-001 0.000000e+000 6.861938e-001

outer loop

vertex 8.393718e+000 4.400000e+000 8.511083e+000

vertex 8.455345e+000 3.000000e+000 8.449454e+000

vertex 8.455345e+000 4.400000e+000 8.449454e+000

endloop

endfacet

facet normal 8.020838e-001 0.000000e+000 5.972114e-001

outer loop

vertex 8.455345e+000 4.400000e+000 8.449454e+000

vertex 8.455345e+000 3.000000e+000 8.449454e+000

vertex 8.505336e+000 3.000000e+000 8.378060e+000

endloop

endfacet

facet normal 8.355240e-001 0.000000e+000 5.494540e-001

outer loop

vertex 8.455345e+000 4.400000e+000 8.449454e+000

vertex 8.505336e+000 3.000000e+000 8.378060e+000

vertex 8.505336e+000 4.400000e+000 8.378060e+000

endloop

endfacet

facet normal 8.936031e-001 0.000000e+000 4.488580e-001

outer loop

vertex 8.505336e+000 4.400000e+000 8.378060e+000

vertex 8.505336e+000 3.000000e+000 8.378060e+000

vertex 8.542170e+000 3.000000e+000 8.299070e+000

endloop

endfacet

facet normal 9.182422e-001 0.000000e+000 3.960194e-001

outer loop

vertex 8.505336e+000 4.400000e+000 8.378060e+000

vertex 8.542170e+000 3.000000e+000 8.299070e+000

vertex 8.542170e+000 4.400000e+000 8.299070e+000

endloop

endfacet

facet normal 9.579706e-001 0.000000e+000 2.868663e-001

outer loop

vertex 8.542170e+000 4.400000e+000 8.299070e+000

vertex 8.542170e+000 3.000000e+000 8.299070e+000

vertex 8.564728e+000 3.000000e+000 8.214885e+000

endloop

endfacet

facet normal 9.730600e-001 0.000000e+000 2.305519e-001

outer loop

vertex 8.542170e+000 4.400000e+000 8.299070e+000

vertex 8.564728e+000 3.000000e+000 8.214885e+000

vertex 8.564728e+000 4.400000e+000 8.214885e+000

endloop

endfacet

facet normal 9.932307e-001 0.000000e+000 1.161583e-001

outer loop

vertex 8.564728e+000 4.400000e+000 8.214885e+000

vertex 8.564728e+000 3.000000e+000 8.214885e+000

vertex 8.572323e+000 3.000000e+000 8.128060e+000

endloop

endfacet

facet normal 9.983119e-001 0.000000e+000 5.807913e-002

outer loop

vertex 8.564728e+000 4.400000e+000 8.214885e+000

vertex 8.572323e+000 3.000000e+000 8.128060e+000

vertex 8.572323e+000 4.400000e+000 8.128060e+000

endloop

endfacet

facet normal 9.983119e-001 0.000000e+000 -5.807913e-002

outer loop

vertex 8.572323e+000 4.400000e+000 8.128060e+000

vertex 8.572323e+000 3.000000e+000 8.128060e+000

vertex 8.564728e+000 3.000000e+000 8.041236e+000

endloop

endfacet

facet normal 9.932307e-001 0.000000e+000 -1.161583e-001

outer loop

vertex 8.572323e+000 4.400000e+000 8.128060e+000

vertex 8.564728e+000 3.000000e+000 8.041236e+000

vertex 8.564728e+000 4.400000e+000 8.041236e+000

endloop

endfacet

facet normal 9.730600e-001 0.000000e+000 -2.305519e-001

outer loop

vertex 8.564728e+000 4.400000e+000 8.041236e+000

vertex 8.564728e+000 3.000000e+000 8.041236e+000

vertex 8.542170e+000 3.000000e+000 7.957050e+000

endloop

endfacet

facet normal 9.579706e-001 0.000000e+000 -2.868663e-001

outer loop

vertex 8.564728e+000 4.400000e+000 8.041236e+000

vertex 8.542170e+000 3.000000e+000 7.957050e+000

vertex 8.542170e+000 4.400000e+000 7.957050e+000

endloop

endfacet

facet normal 9.182422e-001 0.000000e+000 -3.960194e-001

outer loop

vertex 8.542170e+000 4.400000e+000 7.957050e+000

vertex 8.542170e+000 3.000000e+000 7.957050e+000

vertex 8.505336e+000 3.000000e+000 7.878059e+000

endloop

endfacet

facet normal 8.936031e-001 0.000000e+000 -4.488580e-001

outer loop

vertex 8.542170e+000 4.400000e+000 7.957050e+000

vertex 8.505336e+000 3.000000e+000 7.878059e+000

vertex 8.505336e+000 4.400000e+000 7.878059e+000

endloop

endfacet

facet normal 8.355240e-001 0.000000e+000 -5.494540e-001

outer loop

vertex 8.505336e+000 4.400000e+000 7.878059e+000

vertex 8.505336e+000 3.000000e+000 7.878059e+000

vertex 8.455345e+000 3.000000e+000 7.806666e+000

endloop

endfacet

facet normal 8.020838e-001 0.000000e+000 -5.972114e-001

outer loop

vertex 8.505336e+000 4.400000e+000 7.878059e+000

vertex 8.455345e+000 3.000000e+000 7.806666e+000

vertex 8.455345e+000 4.400000e+000 7.806666e+000

endloop

endfacet

facet normal 7.274188e-001 0.000000e+000 -6.861938e-001

outer loop

vertex 8.455345e+000 4.400000e+000 7.806666e+000

vertex 8.455345e+000 3.000000e+000 7.806666e+000

vertex 8.393718e+000 3.000000e+000 7.745038e+000

endloop

endfacet

facet normal 6.861938e-001 0.000000e+000 -7.274188e-001

outer loop

vertex 8.455345e+000 4.400000e+000 7.806666e+000

vertex 8.393718e+000 3.000000e+000 7.745038e+000

vertex 8.393718e+000 4.400000e+000 7.745038e+000

endloop

endfacet

facet normal 5.972114e-001 0.000000e+000 -8.020838e-001

outer loop

vertex 8.393718e+000 4.400000e+000 7.745038e+000

vertex 8.393718e+000 3.000000e+000 7.745038e+000

vertex 8.322324e+000 3.000000e+000 7.695047e+000

endloop

endfacet

facet normal 5.494540e-001 0.000000e+000 -8.355240e-001

outer loop

vertex 8.393718e+000 4.400000e+000 7.745038e+000

vertex 8.322324e+000 3.000000e+000 7.695047e+000

vertex 8.322324e+000 4.400000e+000 7.695047e+000

endloop

endfacet

facet normal 4.488580e-001 0.000000e+000 -8.936031e-001

outer loop

vertex 8.322324e+000 4.400000e+000 7.695047e+000

vertex 8.322324e+000 3.000000e+000 7.695047e+000

vertex 8.243334e+000 3.000000e+000 7.658214e+000

endloop

endfacet

facet normal 3.960194e-001 0.000000e+000 -9.182422e-001

outer loop

vertex 8.322324e+000 4.400000e+000 7.695047e+000

vertex 8.243334e+000 3.000000e+000 7.658214e+000

vertex 8.243334e+000 4.400000e+000 7.658214e+000

endloop

endfacet

facet normal 2.868663e-001 0.000000e+000 -9.579706e-001

outer loop

vertex 8.243334e+000 4.400000e+000 7.658214e+000

vertex 8.243334e+000 3.000000e+000 7.658214e+000

vertex 8.159147e+000 3.000000e+000 7.635656e+000

endloop

endfacet

facet normal 2.305519e-001 0.000000e+000 -9.730600e-001

outer loop

vertex 8.243334e+000 4.400000e+000 7.658214e+000

vertex 8.159147e+000 3.000000e+000 7.635656e+000

vertex 8.159147e+000 4.400000e+000 7.635656e+000

endloop

endfacet

facet normal 1.161583e-001 0.000000e+000 -9.932307e-001

outer loop

vertex 8.159147e+000 4.400000e+000 7.635656e+000

vertex 8.159147e+000 3.000000e+000 7.635656e+000

vertex 8.072324e+000 3.000000e+000 7.628060e+000

endloop

endfacet

facet normal 5.807913e-002 0.000000e+000 -9.983119e-001

outer loop

vertex 8.159147e+000 4.400000e+000 7.635656e+000

vertex 8.072324e+000 3.000000e+000 7.628060e+000

vertex 8.072324e+000 4.400000e+000 7.628060e+000

endloop

endfacet

facet normal -5.807913e-002 0.000000e+000 -9.983119e-001

outer loop

vertex 8.072324e+000 4.400000e+000 7.628060e+000

vertex 8.072324e+000 3.000000e+000 7.628060e+000

vertex 7.985500e+000 3.000000e+000 7.635656e+000

endloop

endfacet

facet normal -1.161583e-001 0.000000e+000 -9.932307e-001

outer loop

vertex 8.072324e+000 4.400000e+000 7.628060e+000

vertex 7.985500e+000 3.000000e+000 7.635656e+000

vertex 7.985500e+000 4.400000e+000 7.635656e+000

endloop

endfacet

facet normal -2.305519e-001 0.000000e+000 -9.730600e-001

outer loop

vertex 7.985500e+000 4.400000e+000 7.635656e+000

vertex 7.985500e+000 3.000000e+000 7.635656e+000

vertex 7.901314e+000 3.000000e+000 7.658214e+000

endloop

endfacet

facet normal -2.868663e-001 0.000000e+000 -9.579706e-001

outer loop

vertex 7.985500e+000 4.400000e+000 7.635656e+000

vertex 7.901314e+000 3.000000e+000 7.658214e+000

vertex 7.901314e+000 4.400000e+000 7.658214e+000

endloop

endfacet

facet normal -3.960194e-001 0.000000e+000 -9.182422e-001

outer loop

vertex 7.901314e+000 4.400000e+000 7.658214e+000

vertex 7.901314e+000 3.000000e+000 7.658214e+000

vertex 7.822324e+000 3.000000e+000 7.695047e+000

endloop

endfacet

facet normal -4.488580e-001 0.000000e+000 -8.936031e-001

outer loop

vertex 7.901314e+000 4.400000e+000 7.658214e+000

vertex 7.822324e+000 3.000000e+000 7.695047e+000

vertex 7.822324e+000 4.400000e+000 7.695047e+000

endloop

endfacet

facet normal -5.494540e-001 0.000000e+000 -8.355240e-001

outer loop

vertex 7.822324e+000 4.400000e+000 7.695047e+000

vertex 7.822324e+000 3.000000e+000 7.695047e+000

vertex 7.750930e+000 3.000000e+000 7.745038e+000

endloop

endfacet

facet normal -5.972114e-001 0.000000e+000 -8.020838e-001

outer loop

vertex 7.822324e+000 4.400000e+000 7.695047e+000

vertex 7.750930e+000 3.000000e+000 7.745038e+000

vertex 7.750930e+000 4.400000e+000 7.745038e+000

endloop

endfacet

facet normal -6.861938e-001 0.000000e+000 -7.274188e-001

outer loop

vertex 7.750930e+000 4.400000e+000 7.745038e+000

vertex 7.750930e+000 3.000000e+000 7.745038e+000

vertex 7.689301e+000 3.000000e+000 7.806666e+000

endloop

endfacet

facet normal -7.274188e-001 0.000000e+000 -6.861938e-001

outer loop

vertex 7.750930e+000 4.400000e+000 7.745038e+000

vertex 7.689301e+000 3.000000e+000 7.806666e+000

vertex 7.689301e+000 4.400000e+000 7.806666e+000

endloop

endfacet

facet normal -8.020838e-001 0.000000e+000 -5.972114e-001

outer loop

vertex 7.689301e+000 4.400000e+000 7.806666e+000

vertex 7.689301e+000 3.000000e+000 7.806666e+000

vertex 7.639311e+000 3.000000e+000 7.878059e+000

endloop

endfacet

facet normal -8.355240e-001 0.000000e+000 -5.494540e-001

outer loop

vertex 7.689301e+000 4.400000e+000 7.806666e+000

vertex 7.639311e+000 3.000000e+000 7.878059e+000

vertex 7.639311e+000 4.400000e+000 7.878059e+000

endloop

endfacet

facet normal -8.936031e-001 0.000000e+000 -4.488580e-001

outer loop

vertex 7.639311e+000 4.400000e+000 7.878059e+000

vertex 7.639311e+000 3.000000e+000 7.878059e+000

vertex 7.602478e+000 3.000000e+000 7.957050e+000

endloop

endfacet

facet normal -9.182422e-001 0.000000e+000 -3.960194e-001

outer loop

vertex 7.639311e+000 4.400000e+000 7.878059e+000

vertex 7.602478e+000 3.000000e+000 7.957050e+000

vertex 7.602478e+000 4.400000e+000 7.957050e+000

endloop

endfacet

facet normal -9.466226e-001 0.000000e+000 -3.223439e-001

outer loop

vertex 7.602478e+000 4.400000e+000 7.957050e+000

vertex 7.602478e+000 3.000000e+000 7.957050e+000

vertex 7.594380e+000 3.000000e+000 7.987269e+000

endloop

endfacet

facet normal -9.637589e-001 0.000000e+000 -2.667744e-001

outer loop

vertex 7.602478e+000 4.400000e+000 7.957050e+000

vertex 7.594380e+000 3.000000e+000 7.987269e+000

vertex 7.579920e+000 4.400000e+000 8.041236e+000

endloop

endfacet

facet normal -9.776480e-001 0.000000e+000 -2.102483e-001

outer loop

vertex 7.579920e+000 4.400000e+000 8.041236e+000

vertex 7.594380e+000 3.000000e+000 7.987269e+000

vertex 7.579920e+000 3.000000e+000 8.041236e+000

endloop

endfacet

facet normal -9.932307e-001 0.000000e+000 -1.161583e-001

outer loop

vertex 7.579920e+000 4.400000e+000 8.041236e+000

vertex 7.579920e+000 3.000000e+000 8.041236e+000

vertex 7.572323e+000 3.000000e+000 8.128060e+000

endloop

endfacet

facet normal 1.161583e-001 0.000000e+000 9.932307e-001

outer loop

vertex 1.078841e+001 4.400000e+000 1.186090e+001

vertex 1.087523e+001 3.000000e+000 1.185330e+001

vertex 1.087523e+001 4.400000e+000 1.185330e+001

endloop

endfacet

facet normal 2.305519e-001 0.000000e+000 9.730600e-001

outer loop

vertex 1.087523e+001 4.400000e+000 1.185330e+001

vertex 1.087523e+001 3.000000e+000 1.185330e+001

vertex 1.095942e+001 3.000000e+000 1.183074e+001

endloop

endfacet

facet normal 2.868663e-001 0.000000e+000 9.579706e-001

outer loop

vertex 1.087523e+001 4.400000e+000 1.185330e+001

vertex 1.095942e+001 3.000000e+000 1.183074e+001

vertex 1.095942e+001 4.400000e+000 1.183074e+001

endloop

endfacet

facet normal 3.960194e-001 0.000000e+000 9.182422e-001

outer loop

vertex 1.095942e+001 4.400000e+000 1.183074e+001

vertex 1.095942e+001 3.000000e+000 1.183074e+001

vertex 1.103841e+001 3.000000e+000 1.179391e+001

endloop

endfacet

facet normal 4.488580e-001 0.000000e+000 8.936031e-001

outer loop

vertex 1.095942e+001 4.400000e+000 1.183074e+001

vertex 1.103841e+001 3.000000e+000 1.179391e+001

vertex 1.103841e+001 4.400000e+000 1.179391e+001

endloop

endfacet

facet normal 5.494540e-001 0.000000e+000 8.355240e-001

outer loop

vertex 1.103841e+001 4.400000e+000 1.179391e+001

vertex 1.103841e+001 3.000000e+000 1.179391e+001

vertex 1.110980e+001 3.000000e+000 1.174392e+001

endloop

endfacet

facet normal 5.972114e-001 0.000000e+000 8.020838e-001

outer loop

vertex 1.103841e+001 4.400000e+000 1.179391e+001

vertex 1.110980e+001 3.000000e+000 1.174392e+001

vertex 1.110980e+001 4.400000e+000 1.174392e+001

endloop

endfacet

facet normal 6.861938e-001 0.000000e+000 7.274188e-001

outer loop

vertex 1.110980e+001 4.400000e+000 1.174392e+001

vertex 1.110980e+001 3.000000e+000 1.174392e+001

vertex 1.117143e+001 3.000000e+000 1.168229e+001

endloop

endfacet

facet normal 7.274188e-001 0.000000e+000 6.861938e-001

outer loop

vertex 1.110980e+001 4.400000e+000 1.174392e+001

vertex 1.117143e+001 3.000000e+000 1.168229e+001

vertex 1.117143e+001 4.400000e+000 1.168229e+001

endloop

endfacet

facet normal 8.020838e-001 0.000000e+000 5.972114e-001

outer loop

vertex 1.117143e+001 4.400000e+000 1.168229e+001

vertex 1.117143e+001 3.000000e+000 1.168229e+001

vertex 1.122142e+001 3.000000e+000 1.161090e+001

endloop

endfacet

facet normal 8.355240e-001 0.000000e+000 5.494540e-001

outer loop

vertex 1.117143e+001 4.400000e+000 1.168229e+001

vertex 1.122142e+001 3.000000e+000 1.161090e+001

vertex 1.122142e+001 4.400000e+000 1.161090e+001

endloop

endfacet

facet normal 8.936031e-001 0.000000e+000 4.488580e-001

outer loop

vertex 1.122142e+001 4.400000e+000 1.161090e+001

vertex 1.122142e+001 3.000000e+000 1.161090e+001

vertex 1.125826e+001 3.000000e+000 1.153191e+001

endloop

endfacet

facet normal 9.182422e-001 0.000000e+000 3.960194e-001

outer loop

vertex 1.122142e+001 4.400000e+000 1.161090e+001

vertex 1.125826e+001 3.000000e+000 1.153191e+001

vertex 1.125826e+001 4.400000e+000 1.153191e+001

endloop

endfacet

facet normal 9.579706e-001 0.000000e+000 2.868663e-001

outer loop

vertex 1.125826e+001 4.400000e+000 1.153191e+001

vertex 1.125826e+001 3.000000e+000 1.153191e+001

vertex 1.128081e+001 3.000000e+000 1.144772e+001

endloop

endfacet

facet normal 9.730600e-001 0.000000e+000 2.305519e-001

outer loop

vertex 1.125826e+001 4.400000e+000 1.153191e+001

vertex 1.128081e+001 3.000000e+000 1.144772e+001

vertex 1.128081e+001 4.400000e+000 1.144772e+001

endloop

endfacet

facet normal 9.932307e-001 0.000000e+000 1.161583e-001

outer loop

vertex 1.128081e+001 4.400000e+000 1.144772e+001

vertex 1.128081e+001 3.000000e+000 1.144772e+001

vertex 1.128841e+001 3.000000e+000 1.136090e+001

endloop

endfacet

facet normal 9.983119e-001 0.000000e+000 5.807913e-002

outer loop

vertex 1.128081e+001 4.400000e+000 1.144772e+001

vertex 1.128841e+001 3.000000e+000 1.136090e+001

vertex 1.128841e+001 4.400000e+000 1.136090e+001

endloop

endfacet

facet normal 9.983119e-001 0.000000e+000 -5.807913e-002

outer loop

vertex 1.128841e+001 4.400000e+000 1.136090e+001

vertex 1.128841e+001 3.000000e+000 1.136090e+001

vertex 1.128081e+001 3.000000e+000 1.127407e+001

endloop

endfacet

facet normal 9.932307e-001 0.000000e+000 -1.161583e-001

outer loop

vertex 1.128841e+001 4.400000e+000 1.136090e+001

vertex 1.128081e+001 3.000000e+000 1.127407e+001

vertex 1.128081e+001 4.400000e+000 1.127407e+001

endloop

endfacet

facet normal 9.730600e-001 0.000000e+000 -2.305519e-001

outer loop

vertex 1.128081e+001 4.400000e+000 1.127407e+001

vertex 1.128081e+001 3.000000e+000 1.127407e+001

vertex 1.125826e+001 3.000000e+000 1.118989e+001

endloop

endfacet

facet normal 9.579706e-001 0.000000e+000 -2.868663e-001

outer loop

vertex 1.128081e+001 4.400000e+000 1.127407e+001

vertex 1.125826e+001 3.000000e+000 1.118989e+001

vertex 1.125826e+001 4.400000e+000 1.118989e+001

endloop

endfacet

facet normal 9.182422e-001 0.000000e+000 -3.960194e-001

outer loop

vertex 1.125826e+001 4.400000e+000 1.118989e+001

vertex 1.125826e+001 3.000000e+000 1.118989e+001

vertex 1.122142e+001 3.000000e+000 1.111090e+001

endloop

endfacet

facet normal 8.936031e-001 0.000000e+000 -4.488580e-001

outer loop

vertex 1.125826e+001 4.400000e+000 1.118989e+001

vertex 1.122142e+001 3.000000e+000 1.111090e+001

vertex 1.122142e+001 4.400000e+000 1.111090e+001

endloop

endfacet

facet normal 8.355240e-001 0.000000e+000 -5.494540e-001

outer loop

vertex 1.122142e+001 4.400000e+000 1.111090e+001

vertex 1.122142e+001 3.000000e+000 1.111090e+001

vertex 1.117143e+001 3.000000e+000 1.103950e+001

endloop

endfacet

facet normal 8.020838e-001 0.000000e+000 -5.972114e-001

outer loop

vertex 1.122142e+001 4.400000e+000 1.111090e+001

vertex 1.117143e+001 3.000000e+000 1.103950e+001

vertex 1.117143e+001 4.400000e+000 1.103950e+001

endloop

endfacet

facet normal 7.274188e-001 0.000000e+000 -6.861938e-001

outer loop

vertex 1.117143e+001 4.400000e+000 1.103950e+001

vertex 1.117143e+001 3.000000e+000 1.103950e+001

vertex 1.110980e+001 3.000000e+000 1.097787e+001

endloop

endfacet

facet normal 6.861938e-001 0.000000e+000 -7.274188e-001

outer loop

vertex 1.117143e+001 4.400000e+000 1.103950e+001

vertex 1.110980e+001 3.000000e+000 1.097787e+001

vertex 1.110980e+001 4.400000e+000 1.097787e+001

endloop

endfacet

facet normal 5.972114e-001 0.000000e+000 -8.020838e-001

outer loop

vertex 1.110980e+001 4.400000e+000 1.097787e+001

vertex 1.110980e+001 3.000000e+000 1.097787e+001

vertex 1.103841e+001 3.000000e+000 1.092788e+001

endloop

endfacet

facet normal 5.494540e-001 0.000000e+000 -8.355240e-001

outer loop

vertex 1.110980e+001 4.400000e+000 1.097787e+001

vertex 1.103841e+001 3.000000e+000 1.092788e+001

vertex 1.103841e+001 4.400000e+000 1.092788e+001

endloop

endfacet

facet normal 4.488580e-001 0.000000e+000 -8.936031e-001

outer loop

vertex 1.103841e+001 4.400000e+000 1.092788e+001

vertex 1.103841e+001 3.000000e+000 1.092788e+001

vertex 1.095942e+001 3.000000e+000 1.089105e+001

endloop

endfacet

facet normal 3.960194e-001 0.000000e+000 -9.182422e-001

outer loop

vertex 1.103841e+001 4.400000e+000 1.092788e+001

vertex 1.095942e+001 3.000000e+000 1.089105e+001

vertex 1.095942e+001 4.400000e+000 1.089105e+001

endloop

endfacet

facet normal 2.868663e-001 0.000000e+000 -9.579706e-001

outer loop

vertex 1.095942e+001 4.400000e+000 1.089105e+001

vertex 1.095942e+001 3.000000e+000 1.089105e+001

vertex 1.087523e+001 3.000000e+000 1.086849e+001

endloop

endfacet

facet normal 2.305519e-001 0.000000e+000 -9.730600e-001

outer loop

vertex 1.095942e+001 4.400000e+000 1.089105e+001

vertex 1.087523e+001 3.000000e+000 1.086849e+001

vertex 1.087523e+001 4.400000e+000 1.086849e+001

endloop

endfacet

facet normal 1.161583e-001 0.000000e+000 -9.932307e-001

outer loop

vertex 1.087523e+001 4.400000e+000 1.086849e+001

vertex 1.087523e+001 3.000000e+000 1.086849e+001

vertex 1.078841e+001 3.000000e+000 1.086090e+001

endloop

endfacet

facet normal 5.807913e-002 0.000000e+000 -9.983119e-001

outer loop

vertex 1.087523e+001 4.400000e+000 1.086849e+001

vertex 1.078841e+001 3.000000e+000 1.086090e+001

vertex 1.078841e+001 4.400000e+000 1.086090e+001

endloop

endfacet

facet normal -5.807913e-002 0.000000e+000 -9.983119e-001

outer loop

vertex 1.078841e+001 4.400000e+000 1.086090e+001

vertex 1.078841e+001 3.000000e+000 1.086090e+001

vertex 1.070159e+001 3.000000e+000 1.086849e+001

endloop

endfacet

facet normal -1.161583e-001 0.000000e+000 -9.932307e-001

outer loop

vertex 1.078841e+001 4.400000e+000 1.086090e+001

vertex 1.070159e+001 3.000000e+000 1.086849e+001

vertex 1.070159e+001 4.400000e+000 1.086849e+001

endloop

endfacet

facet normal -2.305519e-001 0.000000e+000 -9.730600e-001

outer loop

vertex 1.070159e+001 4.400000e+000 1.086849e+001

vertex 1.070159e+001 3.000000e+000 1.086849e+001

vertex 1.061740e+001 3.000000e+000 1.089105e+001

endloop

endfacet

facet normal -2.868663e-001 0.000000e+000 -9.579706e-001

outer loop

vertex 1.070159e+001 4.400000e+000 1.086849e+001

vertex 1.061740e+001 3.000000e+000 1.089105e+001

vertex 1.061740e+001 4.400000e+000 1.089105e+001

endloop

endfacet

facet normal -3.960194e-001 0.000000e+000 -9.182422e-001

outer loop

vertex 1.061740e+001 4.400000e+000 1.089105e+001

vertex 1.061740e+001 3.000000e+000 1.089105e+001

vertex 1.053841e+001 3.000000e+000 1.092788e+001

endloop

endfacet

facet normal -4.488580e-001 0.000000e+000 -8.936031e-001

outer loop

vertex 1.061740e+001 4.400000e+000 1.089105e+001

vertex 1.053841e+001 3.000000e+000 1.092788e+001

vertex 1.053841e+001 4.400000e+000 1.092788e+001

endloop

endfacet

facet normal -5.494540e-001 0.000000e+000 -8.355240e-001

outer loop

vertex 1.053841e+001 4.400000e+000 1.092788e+001

vertex 1.053841e+001 3.000000e+000 1.092788e+001

vertex 1.046702e+001 3.000000e+000 1.097787e+001

endloop

endfacet

facet normal -5.972114e-001 0.000000e+000 -8.020838e-001

outer loop

vertex 1.053841e+001 4.400000e+000 1.092788e+001

vertex 1.046702e+001 3.000000e+000 1.097787e+001

vertex 1.046702e+001 4.400000e+000 1.097787e+001

endloop

endfacet

facet normal -6.861938e-001 0.000000e+000 -7.274188e-001

outer loop

vertex 1.046702e+001 4.400000e+000 1.097787e+001

vertex 1.046702e+001 3.000000e+000 1.097787e+001

vertex 1.040539e+001 3.000000e+000 1.103950e+001

endloop

endfacet

facet normal -7.274188e-001 0.000000e+000 -6.861938e-001

outer loop

vertex 1.046702e+001 4.400000e+000 1.097787e+001

vertex 1.040539e+001 3.000000e+000 1.103950e+001

vertex 1.040539e+001 4.400000e+000 1.103950e+001

endloop

endfacet

facet normal -8.020838e-001 0.000000e+000 -5.972114e-001

outer loop

vertex 1.040539e+001 4.400000e+000 1.103950e+001

vertex 1.040539e+001 3.000000e+000 1.103950e+001

vertex 1.035540e+001 3.000000e+000 1.111090e+001

endloop

endfacet

facet normal -8.355240e-001 0.000000e+000 -5.494540e-001

outer loop

vertex 1.040539e+001 4.400000e+000 1.103950e+001

vertex 1.035540e+001 3.000000e+000 1.111090e+001

vertex 1.035540e+001 4.400000e+000 1.111090e+001

endloop

endfacet

facet normal -8.936031e-001 0.000000e+000 -4.488580e-001

outer loop

vertex 1.035540e+001 4.400000e+000 1.111090e+001

vertex 1.035540e+001 3.000000e+000 1.111090e+001

vertex 1.031856e+001 3.000000e+000 1.118989e+001

endloop

endfacet

facet normal -9.182422e-001 0.000000e+000 -3.960194e-001

outer loop

vertex 1.035540e+001 4.400000e+000 1.111090e+001

vertex 1.031856e+001 3.000000e+000 1.118989e+001

vertex 1.031856e+001 4.400000e+000 1.118989e+001

endloop

endfacet

facet normal -9.579706e-001 0.000000e+000 -2.868663e-001

outer loop

vertex 1.031856e+001 4.400000e+000 1.118989e+001

vertex 1.031856e+001 3.000000e+000 1.118989e+001

vertex 1.029601e+001 3.000000e+000 1.127407e+001

endloop

endfacet

facet normal -9.730600e-001 0.000000e+000 -2.305519e-001

outer loop

vertex 1.031856e+001 4.400000e+000 1.118989e+001

vertex 1.029601e+001 3.000000e+000 1.127407e+001

vertex 1.029601e+001 4.400000e+000 1.127407e+001

endloop

endfacet

facet normal -9.932307e-001 0.000000e+000 -1.161583e-001

outer loop

vertex 1.029601e+001 4.400000e+000 1.127407e+001

vertex 1.029601e+001 3.000000e+000 1.127407e+001

vertex 1.028841e+001 3.000000e+000 1.136090e+001

endloop

endfacet

facet normal -9.983119e-001 0.000000e+000 -5.807913e-002

outer loop

vertex 1.029601e+001 4.400000e+000 1.127407e+001

vertex 1.028841e+001 3.000000e+000 1.136090e+001

vertex 1.028841e+001 4.400000e+000 1.136090e+001

endloop

endfacet

facet normal -9.983119e-001 0.000000e+000 5.807913e-002

outer loop

vertex 1.028841e+001 4.400000e+000 1.136090e+001

vertex 1.028841e+001 3.000000e+000 1.136090e+001

vertex 1.029601e+001 3.000000e+000 1.144772e+001

endloop

endfacet

facet normal -9.932307e-001 0.000000e+000 1.161583e-001

outer loop

vertex 1.028841e+001 4.400000e+000 1.136090e+001

vertex 1.029601e+001 3.000000e+000 1.144772e+001

vertex 1.029601e+001 4.400000e+000 1.144772e+001

endloop

endfacet

facet normal -9.730600e-001 0.000000e+000 2.305519e-001

outer loop

vertex 1.029601e+001 4.400000e+000 1.144772e+001

vertex 1.029601e+001 3.000000e+000 1.144772e+001

vertex 1.031856e+001 3.000000e+000 1.153191e+001

endloop

endfacet

facet normal -9.579706e-001 0.000000e+000 2.868663e-001

outer loop

vertex 1.029601e+001 4.400000e+000 1.144772e+001

vertex 1.031856e+001 3.000000e+000 1.153191e+001

vertex 1.031856e+001 4.400000e+000 1.153191e+001

endloop

endfacet

facet normal -9.182422e-001 0.000000e+000 3.960194e-001

outer loop

vertex 1.031856e+001 4.400000e+000 1.153191e+001

vertex 1.031856e+001 3.000000e+000 1.153191e+001

vertex 1.035540e+001 3.000000e+000 1.161090e+001

endloop

endfacet

facet normal -8.936031e-001 0.000000e+000 4.488580e-001

outer loop

vertex 1.031856e+001 4.400000e+000 1.153191e+001

vertex 1.035540e+001 3.000000e+000 1.161090e+001

vertex 1.035540e+001 4.400000e+000 1.161090e+001

endloop

endfacet

facet normal -8.355240e-001 0.000000e+000 5.494540e-001

outer loop

vertex 1.035540e+001 4.400000e+000 1.161090e+001

vertex 1.035540e+001 3.000000e+000 1.161090e+001

vertex 1.040539e+001 3.000000e+000 1.168229e+001

endloop

endfacet

facet normal -8.020838e-001 0.000000e+000 5.972114e-001

outer loop

vertex 1.035540e+001 4.400000e+000 1.161090e+001

vertex 1.040539e+001 3.000000e+000 1.168229e+001

vertex 1.040539e+001 4.400000e+000 1.168229e+001

endloop

endfacet

facet normal -7.274188e-001 0.000000e+000 6.861938e-001

outer loop

vertex 1.040539e+001 4.400000e+000 1.168229e+001

vertex 1.040539e+001 3.000000e+000 1.168229e+001

vertex 1.046702e+001 3.000000e+000 1.174392e+001

endloop

endfacet

facet normal -6.861938e-001 0.000000e+000 7.274188e-001

outer loop

vertex 1.040539e+001 4.400000e+000 1.168229e+001

vertex 1.046702e+001 3.000000e+000 1.174392e+001

vertex 1.046702e+001 4.400000e+000 1.174392e+001

endloop

endfacet

facet normal -5.972114e-001 0.000000e+000 8.020838e-001

outer loop

vertex 1.046702e+001 4.400000e+000 1.174392e+001

vertex 1.046702e+001 3.000000e+000 1.174392e+001

vertex 1.053841e+001 3.000000e+000 1.179391e+001

endloop

endfacet

facet normal -5.494540e-001 0.000000e+000 8.355240e-001

outer loop

vertex 1.046702e+001 4.400000e+000 1.174392e+001

vertex 1.053841e+001 3.000000e+000 1.179391e+001

vertex 1.053841e+001 4.400000e+000 1.179391e+001

endloop

endfacet

facet normal -4.488580e-001 0.000000e+000 8.936031e-001

outer loop

vertex 1.053841e+001 4.400000e+000 1.179391e+001

vertex 1.053841e+001 3.000000e+000 1.179391e+001

vertex 1.061740e+001 3.000000e+000 1.183074e+001

endloop

endfacet

facet normal -3.960194e-001 0.000000e+000 9.182422e-001

outer loop

vertex 1.053841e+001 4.400000e+000 1.179391e+001

vertex 1.061740e+001 3.000000e+000 1.183074e+001

vertex 1.061740e+001 4.400000e+000 1.183074e+001

endloop

endfacet

facet normal -2.868663e-001 0.000000e+000 9.579706e-001

outer loop

vertex 1.061740e+001 4.400000e+000 1.183074e+001

vertex 1.061740e+001 3.000000e+000 1.183074e+001

vertex 1.070159e+001 3.000000e+000 1.185330e+001

endloop

endfacet

facet normal -2.305519e-001 0.000000e+000 9.730600e-001

outer loop

vertex 1.061740e+001 4.400000e+000 1.183074e+001

vertex 1.070159e+001 3.000000e+000 1.185330e+001

vertex 1.070159e+001 4.400000e+000 1.185330e+001

endloop

endfacet

facet normal -1.161583e-001 0.000000e+000 9.932307e-001

outer loop

vertex 1.070159e+001 4.400000e+000 1.185330e+001

vertex 1.070159e+001 3.000000e+000 1.185330e+001

vertex 1.078841e+001 3.000000e+000 1.186090e+001

endloop

endfacet

facet normal -5.807913e-002 0.000000e+000 9.983119e-001

outer loop

vertex 1.070159e+001 4.400000e+000 1.185330e+001

vertex 1.078841e+001 3.000000e+000 1.186090e+001

vertex 1.078841e+001 4.400000e+000 1.186090e+001

endloop

endfacet

facet normal 5.807913e-002 0.000000e+000 9.983119e-001

outer loop

vertex 1.078841e+001 4.400000e+000 1.186090e+001

vertex 1.078841e+001 3.000000e+000 1.186090e+001

vertex 1.087523e+001 3.000000e+000 1.185330e+001

endloop

endfacet

facet normal 1.161583e-001 0.000000e+000 9.932307e-001

outer loop

vertex 8.116436e+000 4.400000e+000 1.186090e+001

vertex 8.203260e+000 3.000000e+000 1.185330e+001

vertex 8.203260e+000 4.400000e+000 1.185330e+001

endloop

endfacet

facet normal 2.305519e-001 0.000000e+000 9.730600e-001

outer loop

vertex 8.203260e+000 4.400000e+000 1.185330e+001

vertex 8.203260e+000 3.000000e+000 1.185330e+001

vertex 8.287447e+000 3.000000e+000 1.183074e+001

endloop

endfacet

facet normal 2.868663e-001 0.000000e+000 9.579706e-001

outer loop

vertex 8.203260e+000 4.400000e+000 1.185330e+001

vertex 8.287447e+000 3.000000e+000 1.183074e+001

vertex 8.287447e+000 4.400000e+000 1.183074e+001

endloop

endfacet

facet normal 3.960194e-001 0.000000e+000 9.182422e-001

outer loop

vertex 8.287447e+000 4.400000e+000 1.183074e+001

vertex 8.287447e+000 3.000000e+000 1.183074e+001

vertex 8.366437e+000 3.000000e+000 1.179391e+001

endloop

endfacet

facet normal 4.488580e-001 0.000000e+000 8.936031e-001

outer loop

vertex 8.287447e+000 4.400000e+000 1.183074e+001

vertex 8.366437e+000 3.000000e+000 1.179391e+001

vertex 8.366437e+000 4.400000e+000 1.179391e+001

endloop

endfacet

facet normal 5.494540e-001 0.000000e+000 8.355240e-001

outer loop

vertex 8.366437e+000 4.400000e+000 1.179391e+001

vertex 8.366437e+000 3.000000e+000 1.179391e+001

vertex 8.437830e+000 3.000000e+000 1.174392e+001

endloop

endfacet

facet normal 5.972114e-001 0.000000e+000 8.020838e-001

outer loop

vertex 8.366437e+000 4.400000e+000 1.179391e+001

vertex 8.437830e+000 3.000000e+000 1.174392e+001

vertex 8.437830e+000 4.400000e+000 1.174392e+001

endloop

endfacet

facet normal 6.861938e-001 0.000000e+000 7.274188e-001

outer loop

vertex 8.437830e+000 4.400000e+000 1.174392e+001

vertex 8.437830e+000 3.000000e+000 1.174392e+001

vertex 8.499458e+000 3.000000e+000 1.168229e+001

endloop

endfacet

facet normal 7.274188e-001 0.000000e+000 6.861938e-001

outer loop

vertex 8.437830e+000 4.400000e+000 1.174392e+001

vertex 8.499458e+000 3.000000e+000 1.168229e+001

vertex 8.499458e+000 4.400000e+000 1.168229e+001

endloop

endfacet

facet normal 8.020838e-001 0.000000e+000 5.972114e-001

outer loop

vertex 8.499458e+000 4.400000e+000 1.168229e+001

vertex 8.499458e+000 3.000000e+000 1.168229e+001

vertex 8.549449e+000 3.000000e+000 1.161090e+001

endloop

endfacet

facet normal 8.355240e-001 0.000000e+000 5.494540e-001

outer loop

vertex 8.499458e+000 4.400000e+000 1.168229e+001

vertex 8.549449e+000 3.000000e+000 1.161090e+001

vertex 8.549449e+000 4.400000e+000 1.161090e+001

endloop

endfacet

facet normal 8.936031e-001 0.000000e+000 4.488580e-001

outer loop

vertex 8.549449e+000 4.400000e+000 1.161090e+001

vertex 8.549449e+000 3.000000e+000 1.161090e+001

vertex 8.586283e+000 3.000000e+000 1.153191e+001

endloop

endfacet

facet normal 9.182422e-001 0.000000e+000 3.960194e-001

outer loop

vertex 8.549449e+000 4.400000e+000 1.161090e+001

vertex 8.586283e+000 3.000000e+000 1.153191e+001

vertex 8.586283e+000 4.400000e+000 1.153191e+001

endloop

endfacet

facet normal 9.579706e-001 0.000000e+000 2.868663e-001

outer loop

vertex 8.586283e+000 4.400000e+000 1.153191e+001

vertex 8.586283e+000 3.000000e+000 1.153191e+001

vertex 8.608840e+000 3.000000e+000 1.144772e+001

endloop

endfacet

facet normal 9.730600e-001 0.000000e+000 2.305519e-001

outer loop

vertex 8.586283e+000 4.400000e+000 1.153191e+001

vertex 8.608840e+000 3.000000e+000 1.144772e+001

vertex 8.608840e+000 4.400000e+000 1.144772e+001

endloop

endfacet

facet normal 9.932307e-001 0.000000e+000 1.161583e-001

outer loop

vertex 8.608840e+000 4.400000e+000 1.144772e+001

vertex 8.608840e+000 3.000000e+000 1.144772e+001

vertex 8.616436e+000 3.000000e+000 1.136090e+001

endloop

endfacet

facet normal 9.983119e-001 0.000000e+000 5.807913e-002

outer loop

vertex 8.608840e+000 4.400000e+000 1.144772e+001

vertex 8.616436e+000 3.000000e+000 1.136090e+001

vertex 8.616436e+000 4.400000e+000 1.136090e+001

endloop

endfacet

facet normal 9.983119e-001 0.000000e+000 -5.807913e-002

outer loop

vertex 8.616436e+000 4.400000e+000 1.136090e+001

vertex 8.616436e+000 3.000000e+000 1.136090e+001

vertex 8.608840e+000 3.000000e+000 1.127407e+001

endloop

endfacet

facet normal 9.932307e-001 0.000000e+000 -1.161583e-001

outer loop

vertex 8.616436e+000 4.400000e+000 1.136090e+001

vertex 8.608840e+000 3.000000e+000 1.127407e+001

vertex 8.608840e+000 4.400000e+000 1.127407e+001

endloop

endfacet

facet normal 9.730600e-001 0.000000e+000 -2.305519e-001

outer loop

vertex 8.608840e+000 4.400000e+000 1.127407e+001

vertex 8.608840e+000 3.000000e+000 1.127407e+001

vertex 8.586283e+000 3.000000e+000 1.118989e+001

endloop

endfacet

facet normal 9.579706e-001 0.000000e+000 -2.868663e-001

outer loop

vertex 8.608840e+000 4.400000e+000 1.127407e+001

vertex 8.586283e+000 3.000000e+000 1.118989e+001

vertex 8.586283e+000 4.400000e+000 1.118989e+001

endloop

endfacet

facet normal 9.182422e-001 0.000000e+000 -3.960194e-001

outer loop

vertex 8.586283e+000 4.400000e+000 1.118989e+001

vertex 8.586283e+000 3.000000e+000 1.118989e+001

vertex 8.549449e+000 3.000000e+000 1.111090e+001

endloop

endfacet

facet normal 8.936031e-001 0.000000e+000 -4.488580e-001

outer loop

vertex 8.586283e+000 4.400000e+000 1.118989e+001

vertex 8.549449e+000 3.000000e+000 1.111090e+001

vertex 8.549449e+000 4.400000e+000 1.111090e+001

endloop

endfacet

facet normal 8.355240e-001 0.000000e+000 -5.494540e-001

outer loop

vertex 8.549449e+000 4.400000e+000 1.111090e+001

vertex 8.549449e+000 3.000000e+000 1.111090e+001

vertex 8.499458e+000 3.000000e+000 1.103950e+001

endloop

endfacet

facet normal 8.020838e-001 0.000000e+000 -5.972114e-001

outer loop

vertex 8.549449e+000 4.400000e+000 1.111090e+001

vertex 8.499458e+000 3.000000e+000 1.103950e+001

vertex 8.499458e+000 4.400000e+000 1.103950e+001

endloop

endfacet

facet normal 7.274188e-001 0.000000e+000 -6.861938e-001

outer loop

vertex 8.499458e+000 4.400000e+000 1.103950e+001

vertex 8.499458e+000 3.000000e+000 1.103950e+001

vertex 8.437830e+000 3.000000e+000 1.097787e+001

endloop

endfacet

facet normal 6.861938e-001 0.000000e+000 -7.274188e-001

outer loop

vertex 8.499458e+000 4.400000e+000 1.103950e+001

vertex 8.437830e+000 3.000000e+000 1.097787e+001

vertex 8.437830e+000 4.400000e+000 1.097787e+001

endloop

endfacet

facet normal 5.972114e-001 0.000000e+000 -8.020838e-001

outer loop

vertex 8.437830e+000 4.400000e+000 1.097787e+001

vertex 8.437830e+000 3.000000e+000 1.097787e+001

vertex 8.366437e+000 3.000000e+000 1.092788e+001

endloop

endfacet

facet normal 5.494540e-001 0.000000e+000 -8.355240e-001

outer loop

vertex 8.437830e+000 4.400000e+000 1.097787e+001

vertex 8.366437e+000 3.000000e+000 1.092788e+001

vertex 8.366437e+000 4.400000e+000 1.092788e+001

endloop

endfacet

facet normal 4.488580e-001 0.000000e+000 -8.936031e-001

outer loop

vertex 8.366437e+000 4.400000e+000 1.092788e+001

vertex 8.366437e+000 3.000000e+000 1.092788e+001

vertex 8.287447e+000 3.000000e+000 1.089105e+001

endloop

endfacet

facet normal 3.960194e-001 0.000000e+000 -9.182422e-001

outer loop

vertex 8.366437e+000 4.400000e+000 1.092788e+001

vertex 8.287447e+000 3.000000e+000 1.089105e+001

vertex 8.287447e+000 4.400000e+000 1.089105e+001

endloop

endfacet

facet normal 2.868663e-001 0.000000e+000 -9.579706e-001

outer loop

vertex 8.287447e+000 4.400000e+000 1.089105e+001

vertex 8.287447e+000 3.000000e+000 1.089105e+001

vertex 8.203260e+000 3.000000e+000 1.086849e+001

endloop

endfacet

facet normal 2.305519e-001 0.000000e+000 -9.730600e-001

outer loop

vertex 8.287447e+000 4.400000e+000 1.089105e+001

vertex 8.203260e+000 3.000000e+000 1.086849e+001

vertex 8.203260e+000 4.400000e+000 1.086849e+001

endloop

endfacet

facet normal 1.161583e-001 0.000000e+000 -9.932307e-001

outer loop

vertex 8.203260e+000 4.400000e+000 1.086849e+001

vertex 8.203260e+000 3.000000e+000 1.086849e+001

vertex 8.116436e+000 3.000000e+000 1.086090e+001

endloop

endfacet

facet normal 5.807913e-002 0.000000e+000 -9.983119e-001

outer loop

vertex 8.203260e+000 4.400000e+000 1.086849e+001

vertex 8.116436e+000 3.000000e+000 1.086090e+001

vertex 8.116436e+000 4.400000e+000 1.086090e+001

endloop

endfacet

facet normal -5.807913e-002 0.000000e+000 -9.983119e-001

outer loop

vertex 8.116436e+000 4.400000e+000 1.086090e+001

vertex 8.116436e+000 3.000000e+000 1.086090e+001

vertex 8.029613e+000 3.000000e+000 1.086849e+001

endloop

endfacet

facet normal -1.161583e-001 0.000000e+000 -9.932307e-001

outer loop

vertex 8.116436e+000 4.400000e+000 1.086090e+001

vertex 8.029613e+000 3.000000e+000 1.086849e+001

vertex 8.029613e+000 4.400000e+000 1.086849e+001

endloop

endfacet

facet normal -2.305519e-001 0.000000e+000 -9.730600e-001

outer loop

vertex 8.029613e+000 4.400000e+000 1.086849e+001

vertex 8.029613e+000 3.000000e+000 1.086849e+001

vertex 7.945427e+000 3.000000e+000 1.089105e+001

endloop

endfacet

facet normal -2.868663e-001 0.000000e+000 -9.579706e-001

outer loop

vertex 8.029613e+000 4.400000e+000 1.086849e+001

vertex 7.945427e+000 3.000000e+000 1.089105e+001

vertex 7.945427e+000 4.400000e+000 1.089105e+001

endloop

endfacet

facet normal -3.960194e-001 0.000000e+000 -9.182422e-001

outer loop

vertex 7.945427e+000 4.400000e+000 1.089105e+001

vertex 7.945427e+000 3.000000e+000 1.089105e+001

vertex 7.866436e+000 3.000000e+000 1.092788e+001

endloop

endfacet

facet normal -4.488580e-001 0.000000e+000 -8.936031e-001

outer loop

vertex 7.945427e+000 4.400000e+000 1.089105e+001

vertex 7.866436e+000 3.000000e+000 1.092788e+001

vertex 7.866436e+000 4.400000e+000 1.092788e+001

endloop

endfacet

facet normal -5.494540e-001 0.000000e+000 -8.355240e-001

outer loop

vertex 7.866436e+000 4.400000e+000 1.092788e+001

vertex 7.866436e+000 3.000000e+000 1.092788e+001

vertex 7.795043e+000 3.000000e+000 1.097787e+001

endloop

endfacet

facet normal -5.972114e-001 0.000000e+000 -8.020838e-001

outer loop

vertex 7.866436e+000 4.400000e+000 1.092788e+001

vertex 7.795043e+000 3.000000e+000 1.097787e+001

vertex 7.795043e+000 4.400000e+000 1.097787e+001

endloop

endfacet

facet normal -6.861938e-001 0.000000e+000 -7.274188e-001

outer loop

vertex 7.795043e+000 4.400000e+000 1.097787e+001

vertex 7.795043e+000 3.000000e+000 1.097787e+001

vertex 7.733414e+000 3.000000e+000 1.103950e+001

endloop

endfacet

facet normal -7.274188e-001 0.000000e+000 -6.861938e-001

outer loop

vertex 7.795043e+000 4.400000e+000 1.097787e+001

vertex 7.733414e+000 3.000000e+000 1.103950e+001

vertex 7.733414e+000 4.400000e+000 1.103950e+001

endloop

endfacet

facet normal -8.020838e-001 0.000000e+000 -5.972114e-001

outer loop

vertex 7.733414e+000 4.400000e+000 1.103950e+001

vertex 7.733414e+000 3.000000e+000 1.103950e+001

vertex 7.683424e+000 3.000000e+000 1.111090e+001

endloop

endfacet

facet normal -8.355240e-001 0.000000e+000 -5.494540e-001

outer loop

vertex 7.733414e+000 4.400000e+000 1.103950e+001

vertex 7.683424e+000 3.000000e+000 1.111090e+001

vertex 7.683424e+000 4.400000e+000 1.111090e+001

endloop

endfacet

facet normal -8.936031e-001 0.000000e+000 -4.488580e-001

outer loop

vertex 7.683424e+000 4.400000e+000 1.111090e+001

vertex 7.683424e+000 3.000000e+000 1.111090e+001

vertex 7.646590e+000 3.000000e+000 1.118989e+001

endloop

endfacet

facet normal -9.182422e-001 0.000000e+000 -3.960194e-001

outer loop

vertex 7.683424e+000 4.400000e+000 1.111090e+001

vertex 7.646590e+000 3.000000e+000 1.118989e+001

vertex 7.646590e+000 4.400000e+000 1.118989e+001

endloop

endfacet

facet normal -9.579706e-001 0.000000e+000 -2.868663e-001

outer loop

vertex 7.646590e+000 4.400000e+000 1.118989e+001

vertex 7.646590e+000 3.000000e+000 1.118989e+001

vertex 7.624032e+000 3.000000e+000 1.127407e+001

endloop

endfacet

facet normal -9.730600e-001 0.000000e+000 -2.305519e-001

outer loop

vertex 7.646590e+000 4.400000e+000 1.118989e+001

vertex 7.624032e+000 3.000000e+000 1.127407e+001

vertex 7.624032e+000 4.400000e+000 1.127407e+001

endloop

endfacet

facet normal -9.932307e-001 0.000000e+000 -1.161583e-001

outer loop

vertex 7.624032e+000 4.400000e+000 1.127407e+001

vertex 7.624032e+000 3.000000e+000 1.127407e+001

vertex 7.616436e+000 3.000000e+000 1.136090e+001

endloop

endfacet

facet normal -9.983119e-001 0.000000e+000 -5.807913e-002

outer loop

vertex 7.624032e+000 4.400000e+000 1.127407e+001

vertex 7.616436e+000 3.000000e+000 1.136090e+001

vertex 7.616436e+000 4.400000e+000 1.136090e+001

endloop

endfacet

facet normal -9.983119e-001 0.000000e+000 5.807913e-002

outer loop

vertex 7.616436e+000 4.400000e+000 1.136090e+001

vertex 7.616436e+000 3.000000e+000 1.136090e+001

vertex 7.624032e+000 3.000000e+000 1.144772e+001

endloop

endfacet

facet normal -9.932307e-001 0.000000e+000 1.161583e-001

outer loop

vertex 7.616436e+000 4.400000e+000 1.136090e+001

vertex 7.624032e+000 3.000000e+000 1.144772e+001

vertex 7.624032e+000 4.400000e+000 1.144772e+001

endloop

endfacet

facet normal -9.730600e-001 0.000000e+000 2.305519e-001

outer loop

vertex 7.624032e+000 4.400000e+000 1.144772e+001

vertex 7.624032e+000 3.000000e+000 1.144772e+001

vertex 7.646590e+000 3.000000e+000 1.153191e+001

endloop

endfacet

facet normal -9.579706e-001 0.000000e+000 2.868663e-001

outer loop

vertex 7.624032e+000 4.400000e+000 1.144772e+001

vertex 7.646590e+000 3.000000e+000 1.153191e+001

vertex 7.646590e+000 4.400000e+000 1.153191e+001

endloop

endfacet

facet normal -9.182422e-001 0.000000e+000 3.960194e-001

outer loop

vertex 7.646590e+000 4.400000e+000 1.153191e+001

vertex 7.646590e+000 3.000000e+000 1.153191e+001

vertex 7.683424e+000 3.000000e+000 1.161090e+001

endloop

endfacet

facet normal -8.936031e-001 0.000000e+000 4.488580e-001

outer loop

vertex 7.646590e+000 4.400000e+000 1.153191e+001

vertex 7.683424e+000 3.000000e+000 1.161090e+001

vertex 7.683424e+000 4.400000e+000 1.161090e+001

endloop

endfacet

facet normal -8.355240e-001 0.000000e+000 5.494540e-001

outer loop

vertex 7.683424e+000 4.400000e+000 1.161090e+001

vertex 7.683424e+000 3.000000e+000 1.161090e+001

vertex 7.733414e+000 3.000000e+000 1.168229e+001

endloop

endfacet

facet normal -8.020838e-001 0.000000e+000 5.972114e-001

outer loop

vertex 7.683424e+000 4.400000e+000 1.161090e+001

vertex 7.733414e+000 3.000000e+000 1.168229e+001

vertex 7.733414e+000 4.400000e+000 1.168229e+001

endloop

endfacet

facet normal -7.274188e-001 0.000000e+000 6.861938e-001

outer loop

vertex 7.733414e+000 4.400000e+000 1.168229e+001

vertex 7.733414e+000 3.000000e+000 1.168229e+001

vertex 7.795043e+000 3.000000e+000 1.174392e+001

endloop

endfacet

facet normal -6.861938e-001 0.000000e+000 7.274188e-001

outer loop

vertex 7.733414e+000 4.400000e+000 1.168229e+001

vertex 7.795043e+000 3.000000e+000 1.174392e+001

vertex 7.795043e+000 4.400000e+000 1.174392e+001

endloop

endfacet

facet normal -5.972114e-001 0.000000e+000 8.020838e-001

outer loop

vertex 7.795043e+000 4.400000e+000 1.174392e+001

vertex 7.795043e+000 3.000000e+000 1.174392e+001

vertex 7.866436e+000 3.000000e+000 1.179391e+001

endloop

endfacet

facet normal -5.494540e-001 0.000000e+000 8.355240e-001

outer loop

vertex 7.795043e+000 4.400000e+000 1.174392e+001

vertex 7.866436e+000 3.000000e+000 1.179391e+001

vertex 7.866436e+000 4.400000e+000 1.179391e+001

endloop

endfacet

facet normal -4.488580e-001 0.000000e+000 8.936031e-001

outer loop

vertex 7.866436e+000 4.400000e+000 1.179391e+001

vertex 7.866436e+000 3.000000e+000 1.179391e+001

vertex 7.945427e+000 3.000000e+000 1.183074e+001

endloop

endfacet

facet normal -3.960194e-001 0.000000e+000 9.182422e-001

outer loop

vertex 7.866436e+000 4.400000e+000 1.179391e+001

vertex 7.945427e+000 3.000000e+000 1.183074e+001

vertex 7.945427e+000 4.400000e+000 1.183074e+001

endloop

endfacet

facet normal -2.868663e-001 0.000000e+000 9.579706e-001

outer loop

vertex 7.945427e+000 4.400000e+000 1.183074e+001

vertex 7.945427e+000 3.000000e+000 1.183074e+001

vertex 8.029613e+000 3.000000e+000 1.185330e+001

endloop

endfacet

facet normal -2.305519e-001 0.000000e+000 9.730600e-001

outer loop

vertex 7.945427e+000 4.400000e+000 1.183074e+001

vertex 8.029613e+000 3.000000e+000 1.185330e+001

vertex 8.029613e+000 4.400000e+000 1.185330e+001

endloop

endfacet

facet normal -1.161583e-001 0.000000e+000 9.932307e-001

outer loop

vertex 8.029613e+000 4.400000e+000 1.185330e+001

vertex 8.029613e+000 3.000000e+000 1.185330e+001

vertex 8.116436e+000 3.000000e+000 1.186090e+001

endloop

endfacet

facet normal -5.807913e-002 0.000000e+000 9.983119e-001

outer loop

vertex 8.029613e+000 4.400000e+000 1.185330e+001

vertex 8.116436e+000 3.000000e+000 1.186090e+001

vertex 8.116436e+000 4.400000e+000 1.186090e+001

endloop

endfacet

facet normal 5.807913e-002 0.000000e+000 9.983119e-001

outer loop

vertex 8.116436e+000 4.400000e+000 1.186090e+001

vertex 8.116436e+000 3.000000e+000 1.186090e+001

vertex 8.203260e+000 3.000000e+000 1.185330e+001

endloop

endfacet

facet normal 1.161583e-001 0.000000e+000 9.932307e-001

outer loop

vertex 3.788410e+000 4.400000e+000 8.628060e+000

vertex 3.875234e+000 3.000000e+000 8.620463e+000

vertex 3.875234e+000 4.400000e+000 8.620463e+000

endloop

endfacet

facet normal 2.305519e-001 0.000000e+000 9.730600e-001

outer loop

vertex 3.875234e+000 4.400000e+000 8.620463e+000

vertex 3.875234e+000 3.000000e+000 8.620463e+000

vertex 3.959420e+000 3.000000e+000 8.597907e+000

endloop

endfacet

facet normal 2.868663e-001 0.000000e+000 9.579706e-001

outer loop

vertex 3.875234e+000 4.400000e+000 8.620463e+000

vertex 3.959420e+000 3.000000e+000 8.597907e+000

vertex 3.959420e+000 4.400000e+000 8.597907e+000

endloop

endfacet

facet normal 3.960194e-001 0.000000e+000 9.182422e-001

outer loop

vertex 3.959420e+000 4.400000e+000 8.597907e+000

vertex 3.959420e+000 3.000000e+000 8.597907e+000

vertex 4.038410e+000 3.000000e+000 8.561073e+000

endloop

endfacet

facet normal 4.488580e-001 0.000000e+000 8.936031e-001

outer loop

vertex 3.959420e+000 4.400000e+000 8.597907e+000

vertex 4.038410e+000 3.000000e+000 8.561073e+000

vertex 4.038410e+000 4.400000e+000 8.561073e+000

endloop

endfacet

facet normal 5.494540e-001 0.000000e+000 8.355240e-001

outer loop

vertex 4.038410e+000 4.400000e+000 8.561073e+000

vertex 4.038410e+000 3.000000e+000 8.561073e+000

vertex 4.109804e+000 3.000000e+000 8.511083e+000

endloop

endfacet

facet normal 5.972114e-001 0.000000e+000 8.020838e-001

outer loop

vertex 4.038410e+000 4.400000e+000 8.561073e+000

vertex 4.109804e+000 3.000000e+000 8.511083e+000

vertex 4.109804e+000 4.400000e+000 8.511083e+000

endloop

endfacet

facet normal 6.861938e-001 0.000000e+000 7.274188e-001

outer loop

vertex 4.109804e+000 4.400000e+000 8.511083e+000

vertex 4.109804e+000 3.000000e+000 8.511083e+000

vertex 4.171432e+000 3.000000e+000 8.449454e+000

endloop

endfacet

facet normal 7.274188e-001 0.000000e+000 6.861938e-001

outer loop

vertex 4.109804e+000 4.400000e+000 8.511083e+000

vertex 4.171432e+000 3.000000e+000 8.449454e+000

vertex 4.171432e+000 4.400000e+000 8.449454e+000

endloop

endfacet

facet normal 8.020838e-001 0.000000e+000 5.972114e-001

outer loop

vertex 4.171432e+000 4.400000e+000 8.449454e+000

vertex 4.171432e+000 3.000000e+000 8.449454e+000

vertex 4.221423e+000 3.000000e+000 8.378060e+000

endloop

endfacet

facet normal 8.355240e-001 0.000000e+000 5.494540e-001

outer loop

vertex 4.171432e+000 4.400000e+000 8.449454e+000

vertex 4.221423e+000 3.000000e+000 8.378060e+000

vertex 4.221423e+000 4.400000e+000 8.378060e+000

endloop

endfacet

facet normal 8.936031e-001 0.000000e+000 4.488580e-001

outer loop

vertex 4.221423e+000 4.400000e+000 8.378060e+000

vertex 4.221423e+000 3.000000e+000 8.378060e+000

vertex 4.258256e+000 3.000000e+000 8.299070e+000

endloop

endfacet

facet normal 9.182422e-001 0.000000e+000 3.960194e-001

outer loop

vertex 4.221423e+000 4.400000e+000 8.378060e+000

vertex 4.258256e+000 3.000000e+000 8.299070e+000

vertex 4.258256e+000 4.400000e+000 8.299070e+000

endloop

endfacet

facet normal 9.579706e-001 0.000000e+000 2.868663e-001

outer loop

vertex 4.258256e+000 4.400000e+000 8.299070e+000

vertex 4.258256e+000 3.000000e+000 8.299070e+000

vertex 4.280814e+000 3.000000e+000 8.214885e+000

endloop

endfacet

facet normal 9.730600e-001 0.000000e+000 2.305519e-001

outer loop

vertex 4.258256e+000 4.400000e+000 8.299070e+000

vertex 4.280814e+000 3.000000e+000 8.214885e+000

vertex 4.280814e+000 4.400000e+000 8.214885e+000

endloop

endfacet

facet normal 9.932307e-001 0.000000e+000 1.161583e-001

outer loop

vertex 4.280814e+000 4.400000e+000 8.214885e+000

vertex 4.280814e+000 3.000000e+000 8.214885e+000

vertex 4.288410e+000 3.000000e+000 8.128060e+000

endloop

endfacet

facet normal 9.983119e-001 0.000000e+000 5.807913e-002

outer loop

vertex 4.280814e+000 4.400000e+000 8.214885e+000

vertex 4.288410e+000 3.000000e+000 8.128060e+000

vertex 4.288410e+000 4.400000e+000 8.128060e+000

endloop

endfacet

facet normal 9.983119e-001 0.000000e+000 -5.807913e-002

outer loop

vertex 4.288410e+000 4.400000e+000 8.128060e+000

vertex 4.288410e+000 3.000000e+000 8.128060e+000

vertex 4.280814e+000 3.000000e+000 8.041236e+000

endloop

endfacet

facet normal 9.932307e-001 0.000000e+000 -1.161583e-001

outer loop

vertex 4.288410e+000 4.400000e+000 8.128060e+000

vertex 4.280814e+000 3.000000e+000 8.041236e+000

vertex 4.280814e+000 4.400000e+000 8.041236e+000

endloop

endfacet

facet normal 9.730600e-001 0.000000e+000 -2.305519e-001

outer loop

vertex 4.280814e+000 4.400000e+000 8.041236e+000

vertex 4.280814e+000 3.000000e+000 8.041236e+000

vertex 4.258256e+000 3.000000e+000 7.957050e+000

endloop

endfacet

facet normal 9.579706e-001 0.000000e+000 -2.868663e-001

outer loop

vertex 4.280814e+000 4.400000e+000 8.041236e+000

vertex 4.258256e+000 3.000000e+000 7.957050e+000

vertex 4.258256e+000 4.400000e+000 7.957050e+000

endloop

endfacet

facet normal 9.182422e-001 0.000000e+000 -3.960194e-001

outer loop

vertex 4.258256e+000 4.400000e+000 7.957050e+000

vertex 4.258256e+000 3.000000e+000 7.957050e+000

vertex 4.221423e+000 3.000000e+000 7.878059e+000

endloop

endfacet

facet normal 8.936031e-001 0.000000e+000 -4.488580e-001

outer loop

vertex 4.258256e+000 4.400000e+000 7.957050e+000

vertex 4.221423e+000 3.000000e+000 7.878059e+000

vertex 4.221423e+000 4.400000e+000 7.878059e+000

endloop

endfacet

facet normal 8.355240e-001 0.000000e+000 -5.494540e-001

outer loop

vertex 4.221423e+000 4.400000e+000 7.878059e+000

vertex 4.221423e+000 3.000000e+000 7.878059e+000

vertex 4.171432e+000 3.000000e+000 7.806666e+000

endloop

endfacet

facet normal 8.020838e-001 0.000000e+000 -5.972114e-001

outer loop

vertex 4.221423e+000 4.400000e+000 7.878059e+000

vertex 4.171432e+000 3.000000e+000 7.806666e+000

vertex 4.171432e+000 4.400000e+000 7.806666e+000

endloop

endfacet

facet normal 7.274188e-001 0.000000e+000 -6.861938e-001

outer loop

vertex 4.171432e+000 4.400000e+000 7.806666e+000

vertex 4.171432e+000 3.000000e+000 7.806666e+000

vertex 4.109804e+000 3.000000e+000 7.745038e+000

endloop

endfacet

facet normal 6.861938e-001 0.000000e+000 -7.274188e-001

outer loop

vertex 4.171432e+000 4.400000e+000 7.806666e+000

vertex 4.109804e+000 3.000000e+000 7.745038e+000

vertex 4.109804e+000 4.400000e+000 7.745038e+000

endloop

endfacet

facet normal 5.972114e-001 0.000000e+000 -8.020838e-001

outer loop

vertex 4.109804e+000 4.400000e+000 7.745038e+000

vertex 4.109804e+000 3.000000e+000 7.745038e+000

vertex 4.038410e+000 3.000000e+000 7.695047e+000

endloop

endfacet

facet normal 5.494540e-001 0.000000e+000 -8.355240e-001

outer loop

vertex 4.109804e+000 4.400000e+000 7.745038e+000

vertex 4.038410e+000 3.000000e+000 7.695047e+000

vertex 4.038410e+000 4.400000e+000 7.695047e+000

endloop

endfacet

facet normal 4.488580e-001 0.000000e+000 -8.936031e-001

outer loop

vertex 4.038410e+000 4.400000e+000 7.695047e+000

vertex 4.038410e+000 3.000000e+000 7.695047e+000

vertex 3.959420e+000 3.000000e+000 7.658214e+000

endloop

endfacet

facet normal 3.960194e-001 0.000000e+000 -9.182422e-001

outer loop

vertex 4.038410e+000 4.400000e+000 7.695047e+000

vertex 3.959420e+000 3.000000e+000 7.658214e+000

vertex 3.959420e+000 4.400000e+000 7.658214e+000

endloop

endfacet

facet normal 2.868663e-001 0.000000e+000 -9.579706e-001

outer loop

vertex 3.959420e+000 4.400000e+000 7.658214e+000

vertex 3.959420e+000 3.000000e+000 7.658214e+000

vertex 3.875234e+000 3.000000e+000 7.635656e+000

endloop

endfacet

facet normal 2.305519e-001 0.000000e+000 -9.730600e-001

outer loop

vertex 3.959420e+000 4.400000e+000 7.658214e+000

vertex 3.875234e+000 3.000000e+000 7.635656e+000

vertex 3.875234e+000 4.400000e+000 7.635656e+000

endloop

endfacet

facet normal 1.161583e-001 0.000000e+000 -9.932307e-001

outer loop

vertex 3.875234e+000 4.400000e+000 7.635656e+000

vertex 3.875234e+000 3.000000e+000 7.635656e+000

vertex 3.788410e+000 3.000000e+000 7.628060e+000

endloop

endfacet

facet normal 5.807913e-002 0.000000e+000 -9.983119e-001

outer loop

vertex 3.875234e+000 4.400000e+000 7.635656e+000

vertex 3.788410e+000 3.000000e+000 7.628060e+000

vertex 3.788410e+000 4.400000e+000 7.628060e+000

endloop

endfacet

facet normal -5.807913e-002 0.000000e+000 -9.983119e-001

outer loop

vertex 3.788410e+000 4.400000e+000 7.628060e+000

vertex 3.788410e+000 3.000000e+000 7.628060e+000

vertex 3.701586e+000 3.000000e+000 7.635656e+000

endloop

endfacet

facet normal -1.161583e-001 0.000000e+000 -9.932307e-001

outer loop

vertex 3.788410e+000 4.400000e+000 7.628060e+000

vertex 3.701586e+000 3.000000e+000 7.635656e+000

vertex 3.701586e+000 4.400000e+000 7.635656e+000

endloop

endfacet

facet normal -2.305519e-001 0.000000e+000 -9.730600e-001

outer loop

vertex 3.701586e+000 4.400000e+000 7.635656e+000

vertex 3.701586e+000 3.000000e+000 7.635656e+000

vertex 3.617400e+000 3.000000e+000 7.658214e+000

endloop

endfacet

facet normal -2.868663e-001 0.000000e+000 -9.579706e-001

outer loop

vertex 3.701586e+000 4.400000e+000 7.635656e+000

vertex 3.617400e+000 3.000000e+000 7.658214e+000

vertex 3.617400e+000 4.400000e+000 7.658214e+000

endloop

endfacet

facet normal -3.960194e-001 0.000000e+000 -9.182422e-001

outer loop

vertex 3.617400e+000 4.400000e+000 7.658214e+000

vertex 3.617400e+000 3.000000e+000 7.658214e+000

vertex 3.538410e+000 3.000000e+000 7.695047e+000

endloop

endfacet

facet normal -4.488580e-001 0.000000e+000 -8.936031e-001

outer loop

vertex 3.617400e+000 4.400000e+000 7.658214e+000

vertex 3.538410e+000 3.000000e+000 7.695047e+000

vertex 3.538410e+000 4.400000e+000 7.695047e+000

endloop

endfacet

facet normal -5.494540e-001 0.000000e+000 -8.355240e-001

outer loop

vertex 3.538410e+000 4.400000e+000 7.695047e+000

vertex 3.538410e+000 3.000000e+000 7.695047e+000

vertex 3.467016e+000 3.000000e+000 7.745038e+000

endloop

endfacet

facet normal -5.972114e-001 0.000000e+000 -8.020838e-001

outer loop

vertex 3.538410e+000 4.400000e+000 7.695047e+000

vertex 3.467016e+000 3.000000e+000 7.745038e+000

vertex 3.467016e+000 4.400000e+000 7.745038e+000

endloop

endfacet

facet normal -6.861938e-001 0.000000e+000 -7.274188e-001

outer loop

vertex 3.467016e+000 4.400000e+000 7.745038e+000

vertex 3.467016e+000 3.000000e+000 7.745038e+000

vertex 3.405388e+000 3.000000e+000 7.806666e+000

endloop

endfacet

facet normal -7.274188e-001 0.000000e+000 -6.861938e-001

outer loop

vertex 3.467016e+000 4.400000e+000 7.745038e+000

vertex 3.405388e+000 3.000000e+000 7.806666e+000

vertex 3.405388e+000 4.400000e+000 7.806666e+000

endloop

endfacet

facet normal -8.020838e-001 0.000000e+000 -5.972114e-001

outer loop

vertex 3.405388e+000 4.400000e+000 7.806666e+000

vertex 3.405388e+000 3.000000e+000 7.806666e+000

vertex 3.355397e+000 3.000000e+000 7.878059e+000

endloop

endfacet

facet normal -8.355240e-001 0.000000e+000 -5.494540e-001

outer loop

vertex 3.405388e+000 4.400000e+000 7.806666e+000

vertex 3.355397e+000 3.000000e+000 7.878059e+000

vertex 3.355397e+000 4.400000e+000 7.878059e+000

endloop

endfacet

facet normal -8.936031e-001 0.000000e+000 -4.488580e-001

outer loop

vertex 3.355397e+000 4.400000e+000 7.878059e+000

vertex 3.355397e+000 3.000000e+000 7.878059e+000

vertex 3.318563e+000 3.000000e+000 7.957050e+000

endloop

endfacet

facet normal -9.182422e-001 0.000000e+000 -3.960194e-001

outer loop

vertex 3.355397e+000 4.400000e+000 7.878059e+000

vertex 3.318563e+000 3.000000e+000 7.957050e+000

vertex 3.318563e+000 4.400000e+000 7.957050e+000

endloop

endfacet

facet normal -9.579706e-001 0.000000e+000 -2.868663e-001

outer loop

vertex 3.318563e+000 4.400000e+000 7.957050e+000

vertex 3.318563e+000 3.000000e+000 7.957050e+000

vertex 3.296006e+000 3.000000e+000 8.041236e+000

endloop

endfacet

facet normal -9.730600e-001 0.000000e+000 -2.305519e-001

outer loop

vertex 3.318563e+000 4.400000e+000 7.957050e+000

vertex 3.296006e+000 3.000000e+000 8.041236e+000

vertex 3.296006e+000 4.400000e+000 8.041236e+000

endloop

endfacet

facet normal -9.932307e-001 0.000000e+000 -1.161583e-001

outer loop

vertex 3.296006e+000 4.400000e+000 8.041236e+000

vertex 3.296006e+000 3.000000e+000 8.041236e+000

vertex 3.288410e+000 3.000000e+000 8.128060e+000

endloop

endfacet

facet normal -9.983119e-001 0.000000e+000 -5.807913e-002

outer loop

vertex 3.296006e+000 4.400000e+000 8.041236e+000

vertex 3.288410e+000 3.000000e+000 8.128060e+000

vertex 3.288410e+000 4.400000e+000 8.128060e+000

endloop

endfacet

facet normal -9.983119e-001 0.000000e+000 5.807913e-002

outer loop

vertex 3.288410e+000 4.400000e+000 8.128060e+000

vertex 3.288410e+000 3.000000e+000 8.128060e+000

vertex 3.296006e+000 3.000000e+000 8.214885e+000

endloop

endfacet

facet normal -9.932307e-001 0.000000e+000 1.161583e-001

outer loop

vertex 3.288410e+000 4.400000e+000 8.128060e+000

vertex 3.296006e+000 3.000000e+000 8.214885e+000

vertex 3.296006e+000 4.400000e+000 8.214885e+000

endloop

endfacet

facet normal -9.730600e-001 0.000000e+000 2.305519e-001

outer loop

vertex 3.296006e+000 4.400000e+000 8.214885e+000

vertex 3.296006e+000 3.000000e+000 8.214885e+000

vertex 3.318563e+000 3.000000e+000 8.299070e+000

endloop

endfacet

facet normal -9.579706e-001 0.000000e+000 2.868663e-001

outer loop

vertex 3.296006e+000 4.400000e+000 8.214885e+000

vertex 3.318563e+000 3.000000e+000 8.299070e+000

vertex 3.318563e+000 4.400000e+000 8.299070e+000

endloop

endfacet

facet normal -9.182422e-001 0.000000e+000 3.960194e-001

outer loop

vertex 3.318563e+000 4.400000e+000 8.299070e+000

vertex 3.318563e+000 3.000000e+000 8.299070e+000

vertex 3.355397e+000 3.000000e+000 8.378060e+000

endloop

endfacet

facet normal -8.936031e-001 0.000000e+000 4.488580e-001

outer loop

vertex 3.318563e+000 4.400000e+000 8.299070e+000

vertex 3.355397e+000 3.000000e+000 8.378060e+000

vertex 3.355397e+000 4.400000e+000 8.378060e+000

endloop

endfacet

facet normal -8.355240e-001 0.000000e+000 5.494540e-001

outer loop

vertex 3.355397e+000 4.400000e+000 8.378060e+000

vertex 3.355397e+000 3.000000e+000 8.378060e+000

vertex 3.405388e+000 3.000000e+000 8.449454e+000

endloop

endfacet

facet normal -8.020838e-001 0.000000e+000 5.972114e-001

outer loop

vertex 3.355397e+000 4.400000e+000 8.378060e+000

vertex 3.405388e+000 3.000000e+000 8.449454e+000

vertex 3.405388e+000 4.400000e+000 8.449454e+000

endloop

endfacet

facet normal -7.274188e-001 0.000000e+000 6.861938e-001

outer loop

vertex 3.405388e+000 4.400000e+000 8.449454e+000

vertex 3.405388e+000 3.000000e+000 8.449454e+000

vertex 3.467016e+000 3.000000e+000 8.511083e+000

endloop

endfacet

facet normal -6.861938e-001 0.000000e+000 7.274188e-001

outer loop

vertex 3.405388e+000 4.400000e+000 8.449454e+000

vertex 3.467016e+000 3.000000e+000 8.511083e+000

vertex 3.467016e+000 4.400000e+000 8.511083e+000

endloop

endfacet

facet normal -5.972114e-001 0.000000e+000 8.020838e-001

outer loop

vertex 3.467016e+000 4.400000e+000 8.511083e+000

vertex 3.467016e+000 3.000000e+000 8.511083e+000

vertex 3.538410e+000 3.000000e+000 8.561073e+000

endloop

endfacet

facet normal -5.494540e-001 0.000000e+000 8.355240e-001

outer loop

vertex 3.467016e+000 4.400000e+000 8.511083e+000

vertex 3.538410e+000 3.000000e+000 8.561073e+000

vertex 3.538410e+000 4.400000e+000 8.561073e+000

endloop

endfacet

facet normal -4.488580e-001 0.000000e+000 8.936031e-001

outer loop

vertex 3.538410e+000 4.400000e+000 8.561073e+000

vertex 3.538410e+000 3.000000e+000 8.561073e+000

vertex 3.617400e+000 3.000000e+000 8.597907e+000

endloop

endfacet

facet normal -3.960194e-001 0.000000e+000 9.182422e-001

outer loop

vertex 3.538410e+000 4.400000e+000 8.561073e+000

vertex 3.617400e+000 3.000000e+000 8.597907e+000

vertex 3.617400e+000 4.400000e+000 8.597907e+000

endloop

endfacet

facet normal -2.868663e-001 0.000000e+000 9.579706e-001

outer loop

vertex 3.617400e+000 4.400000e+000 8.597907e+000

vertex 3.617400e+000 3.000000e+000 8.597907e+000

vertex 3.701586e+000 3.000000e+000 8.620463e+000

endloop

endfacet

facet normal -2.305519e-001 0.000000e+000 9.730600e-001

outer loop

vertex 3.617400e+000 4.400000e+000 8.597907e+000

vertex 3.701586e+000 3.000000e+000 8.620463e+000

vertex 3.701586e+000 4.400000e+000 8.620463e+000

endloop

endfacet

facet normal -1.161583e-001 0.000000e+000 9.932307e-001

outer loop

vertex 3.701586e+000 4.400000e+000 8.620463e+000

vertex 3.701586e+000 3.000000e+000 8.620463e+000

vertex 3.788410e+000 3.000000e+000 8.628060e+000

endloop

endfacet

facet normal -5.807913e-002 0.000000e+000 9.983119e-001

outer loop

vertex 3.701586e+000 4.400000e+000 8.620463e+000

vertex 3.788410e+000 3.000000e+000 8.628060e+000

vertex 3.788410e+000 4.400000e+000 8.628060e+000

endloop

endfacet

facet normal 5.807913e-002 0.000000e+000 9.983119e-001

outer loop

vertex 3.788410e+000 4.400000e+000 8.628060e+000

vertex 3.788410e+000 3.000000e+000 8.628060e+000

vertex 3.875234e+000 3.000000e+000 8.620463e+000

endloop

endfacet

facet normal -9.983119e-001 0.000000e+000 -5.807913e-002

outer loop

vertex 5.799197e-001 4.400000e+000 8.041236e+000

vertex 5.723238e-001 3.000000e+000 8.128060e+000

vertex 5.723238e-001 4.400000e+000 8.128060e+000

endloop

endfacet

facet normal -9.983119e-001 0.000000e+000 5.807913e-002

outer loop

vertex 5.723238e-001 4.400000e+000 8.128060e+000

vertex 5.723238e-001 3.000000e+000 8.128060e+000

vertex 5.799197e-001 3.000000e+000 8.214885e+000

endloop

endfacet

facet normal -9.932307e-001 0.000000e+000 1.161583e-001

outer loop

vertex 5.723238e-001 4.400000e+000 8.128060e+000

vertex 5.799197e-001 3.000000e+000 8.214885e+000

vertex 5.799197e-001 4.400000e+000 8.214885e+000

endloop

endfacet

facet normal -9.776480e-001 0.000000e+000 2.102483e-001

outer loop

vertex 5.799197e-001 4.400000e+000 8.214885e+000

vertex 5.799197e-001 3.000000e+000 8.214885e+000

vertex 5.943798e-001 3.000000e+000 8.268851e+000

endloop

endfacet

facet normal -9.637589e-001 0.000000e+000 2.667744e-001

outer loop

vertex 5.799197e-001 4.400000e+000 8.214885e+000

vertex 5.943798e-001 3.000000e+000 8.268851e+000

vertex 6.024773e-001 4.400000e+000 8.299070e+000

endloop

endfacet

facet normal -9.466226e-001 0.000000e+000 3.223439e-001

outer loop

vertex 5.943798e-001 3.000000e+000 8.268851e+000

vertex 6.024773e-001 3.000000e+000 8.299070e+000

vertex 6.024773e-001 4.400000e+000 8.299070e+000

endloop

endfacet

facet normal -9.182422e-001 0.000000e+000 3.960194e-001

outer loop

vertex 6.024773e-001 4.400000e+000 8.299070e+000

vertex 6.024773e-001 3.000000e+000 8.299070e+000

vertex 6.393110e-001 3.000000e+000 8.378060e+000

endloop

endfacet

facet normal -8.936031e-001 0.000000e+000 4.488580e-001

outer loop

vertex 6.024773e-001 4.400000e+000 8.299070e+000

vertex 6.393110e-001 3.000000e+000 8.378060e+000

vertex 6.393110e-001 4.400000e+000 8.378060e+000

endloop

endfacet

facet normal -8.355240e-001 0.000000e+000 5.494540e-001

outer loop

vertex 6.393110e-001 4.400000e+000 8.378060e+000

vertex 6.393110e-001 3.000000e+000 8.378060e+000

vertex 6.893016e-001 3.000000e+000 8.449454e+000

endloop

endfacet

facet normal -8.020838e-001 0.000000e+000 5.972114e-001

outer loop

vertex 6.393110e-001 4.400000e+000 8.378060e+000

vertex 6.893016e-001 3.000000e+000 8.449454e+000

vertex 6.893016e-001 4.400000e+000 8.449454e+000

endloop

endfacet

facet normal -7.274188e-001 0.000000e+000 6.861938e-001

outer loop

vertex 6.893016e-001 4.400000e+000 8.449454e+000

vertex 6.893016e-001 3.000000e+000 8.449454e+000

vertex 7.509301e-001 3.000000e+000 8.511083e+000

endloop

endfacet

facet normal -6.861938e-001 0.000000e+000 7.274188e-001

outer loop

vertex 6.893016e-001 4.400000e+000 8.449454e+000

vertex 7.509301e-001 3.000000e+000 8.511083e+000

vertex 7.509301e-001 4.400000e+000 8.511083e+000

endloop

endfacet

facet normal -5.972114e-001 0.000000e+000 8.020838e-001

outer loop

vertex 7.509301e-001 4.400000e+000 8.511083e+000

vertex 7.509301e-001 3.000000e+000 8.511083e+000

vertex 8.223239e-001 3.000000e+000 8.561073e+000

endloop

endfacet

facet normal -5.494540e-001 0.000000e+000 8.355240e-001

outer loop

vertex 7.509301e-001 4.400000e+000 8.511083e+000

vertex 8.223239e-001 3.000000e+000 8.561073e+000

vertex 8.223239e-001 4.400000e+000 8.561073e+000

endloop

endfacet

facet normal -4.488580e-001 0.000000e+000 8.936031e-001

outer loop

vertex 8.223239e-001 4.400000e+000 8.561073e+000

vertex 8.223239e-001 3.000000e+000 8.561073e+000

vertex 9.013135e-001 3.000000e+000 8.597907e+000

endloop

endfacet

facet normal -3.960194e-001 0.000000e+000 9.182422e-001

outer loop

vertex 8.223239e-001 4.400000e+000 8.561073e+000

vertex 9.013135e-001 3.000000e+000 8.597907e+000

vertex 9.013135e-001 4.400000e+000 8.597907e+000

endloop

endfacet

facet normal -2.868663e-001 0.000000e+000 9.579706e-001

outer loop

vertex 9.013135e-001 4.400000e+000 8.597907e+000

vertex 9.013135e-001 3.000000e+000 8.597907e+000

vertex 9.854995e-001 3.000000e+000 8.620463e+000

endloop

endfacet

facet normal -2.305519e-001 0.000000e+000 9.730600e-001

outer loop

vertex 9.013135e-001 4.400000e+000 8.597907e+000

vertex 9.854995e-001 3.000000e+000 8.620463e+000

vertex 9.854995e-001 4.400000e+000 8.620463e+000

endloop

endfacet

facet normal -1.161583e-001 0.000000e+000 9.932307e-001

outer loop

vertex 9.854995e-001 4.400000e+000 8.620463e+000

vertex 9.854995e-001 3.000000e+000 8.620463e+000

vertex 1.072323e+000 3.000000e+000 8.628060e+000

endloop

endfacet

facet normal -5.807913e-002 0.000000e+000 9.983119e-001

outer loop

vertex 9.854995e-001 4.400000e+000 8.620463e+000

vertex 1.072323e+000 3.000000e+000 8.628060e+000

vertex 1.072323e+000 4.400000e+000 8.628060e+000

endloop

endfacet

facet normal 5.807913e-002 0.000000e+000 9.983119e-001

outer loop

vertex 1.072323e+000 4.400000e+000 8.628060e+000

vertex 1.072323e+000 3.000000e+000 8.628060e+000

vertex 1.159148e+000 3.000000e+000 8.620463e+000

endloop

endfacet

facet normal 1.161583e-001 0.000000e+000 9.932307e-001

outer loop

vertex 1.072323e+000 4.400000e+000 8.628060e+000

vertex 1.159148e+000 3.000000e+000 8.620463e+000

vertex 1.159148e+000 4.400000e+000 8.620463e+000

endloop

endfacet

facet normal 2.305519e-001 0.000000e+000 9.730600e-001

outer loop

vertex 1.159148e+000 4.400000e+000 8.620463e+000

vertex 1.159148e+000 3.000000e+000 8.620463e+000

vertex 1.243334e+000 3.000000e+000 8.597907e+000

endloop

endfacet

facet normal 2.868663e-001 0.000000e+000 9.579706e-001

outer loop

vertex 1.159148e+000 4.400000e+000 8.620463e+000

vertex 1.243334e+000 3.000000e+000 8.597907e+000

vertex 1.243334e+000 4.400000e+000 8.597907e+000

endloop

endfacet

facet normal 3.960194e-001 0.000000e+000 9.182422e-001

outer loop

vertex 1.243334e+000 4.400000e+000 8.597907e+000

vertex 1.243334e+000 3.000000e+000 8.597907e+000

vertex 1.322323e+000 3.000000e+000 8.561073e+000

endloop

endfacet

facet normal 4.488580e-001 0.000000e+000 8.936031e-001

outer loop

vertex 1.243334e+000 4.400000e+000 8.597907e+000

vertex 1.322323e+000 3.000000e+000 8.561073e+000

vertex 1.322323e+000 4.400000e+000 8.561073e+000

endloop

endfacet

facet normal 5.494540e-001 0.000000e+000 8.355240e-001

outer loop

vertex 1.322323e+000 4.400000e+000 8.561073e+000

vertex 1.322323e+000 3.000000e+000 8.561073e+000

vertex 1.393717e+000 3.000000e+000 8.511083e+000

endloop

endfacet

facet normal 5.972114e-001 0.000000e+000 8.020838e-001

outer loop

vertex 1.322323e+000 4.400000e+000 8.561073e+000

vertex 1.393717e+000 3.000000e+000 8.511083e+000

vertex 1.393717e+000 4.400000e+000 8.511083e+000

endloop

endfacet

facet normal 6.861938e-001 0.000000e+000 7.274188e-001

outer loop

vertex 1.393717e+000 4.400000e+000 8.511083e+000

vertex 1.393717e+000 3.000000e+000 8.511083e+000

vertex 1.455346e+000 3.000000e+000 8.449454e+000

endloop

endfacet

facet normal 7.274188e-001 0.000000e+000 6.861938e-001

outer loop

vertex 1.393717e+000 4.400000e+000 8.511083e+000

vertex 1.455346e+000 3.000000e+000 8.449454e+000

vertex 1.455346e+000 4.400000e+000 8.449454e+000

endloop

endfacet

facet normal 8.020838e-001 0.000000e+000 5.972114e-001

outer loop

vertex 1.455346e+000 4.400000e+000 8.449454e+000

vertex 1.455346e+000 3.000000e+000 8.449454e+000

vertex 1.505336e+000 3.000000e+000 8.378060e+000

endloop

endfacet

facet normal 8.355240e-001 0.000000e+000 5.494540e-001

outer loop

vertex 1.455346e+000 4.400000e+000 8.449454e+000

vertex 1.505336e+000 3.000000e+000 8.378060e+000

vertex 1.505336e+000 4.400000e+000 8.378060e+000

endloop

endfacet

facet normal 8.936031e-001 0.000000e+000 4.488580e-001

outer loop

vertex 1.505336e+000 4.400000e+000 8.378060e+000

vertex 1.505336e+000 3.000000e+000 8.378060e+000

vertex 1.542170e+000 3.000000e+000 8.299070e+000

endloop

endfacet

facet normal 9.182422e-001 0.000000e+000 3.960194e-001

outer loop

vertex 1.505336e+000 4.400000e+000 8.378060e+000

vertex 1.542170e+000 3.000000e+000 8.299070e+000

vertex 1.542170e+000 4.400000e+000 8.299070e+000

endloop

endfacet

facet normal 9.579706e-001 0.000000e+000 2.868663e-001

outer loop

vertex 1.542170e+000 4.400000e+000 8.299070e+000

vertex 1.542170e+000 3.000000e+000 8.299070e+000

vertex 1.564728e+000 3.000000e+000 8.214885e+000

endloop

endfacet

facet normal 9.730600e-001 0.000000e+000 2.305519e-001

outer loop

vertex 1.542170e+000 4.400000e+000 8.299070e+000
[truncated: 655,912 more chars]
